# Supplementary material for: Biology-inspired graph neural network encodes reactome and reveals biochemical reactions of disease
Source: Patterns (N Y). 2023 May 22;4(7):100758. doi: 10.1016/j.patter.2023.100758 (PMC10382942; doi:10.1016/j.patter.2023.100758)
Supplement: Document S1. Figures S1–S4 and Tables S1–S3 [file mmc1.pdf]

**Patterns, Volume *4***

## Supplemental information

# Biology-inspired graph neural network encodes reactome and reveals biochemical reactions of disease

**Joshua G. Burkhart, Guanming Wu, Xubo Song, Francesco Raimondi, Shannon McWeeney, Melissa H. Wong, and Youping Deng**

# Supplementary Figures & Figure Legends

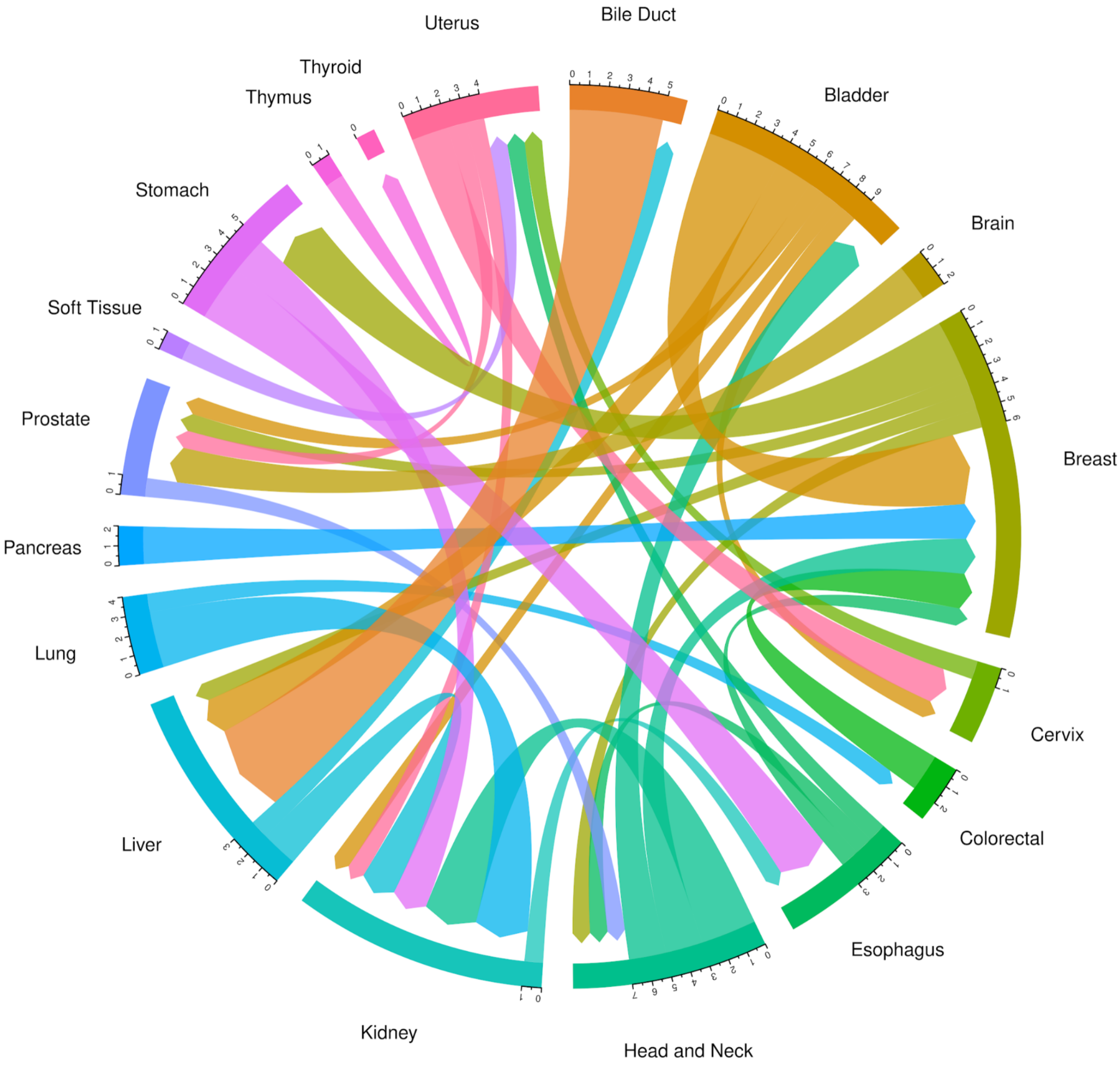


**Supplementary Figure 1: Resnet TCGA Tissue Sample Misclassifications**

Chord diagram showing Resnet TCGA tissue misclassifications from the held-out dataset of 370 samples. True sample labels are numbered on the edge of the diagram and their misclassified label is depicted as the target of the corresponding ribbon. For example, the Resnet model misclassified 1 cervix sample as uterus and it misclassified 2 uterus samples and 1 bladder sample as cervix.

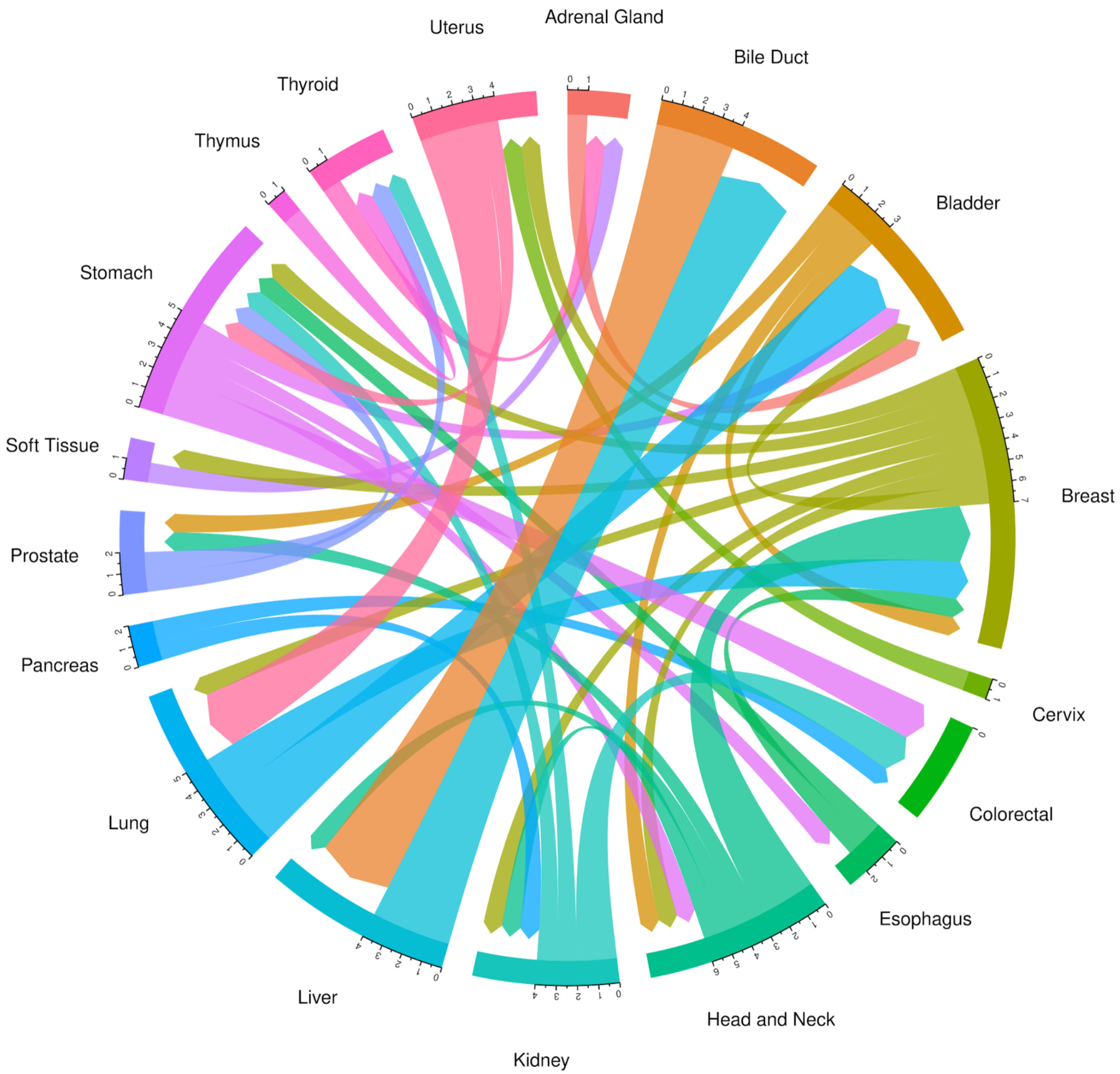


**Supplementary Figure 2: GNN TCGA Tissue Sample Misclassifications**

Chord diagram showing GNN TCGA tissue misclassifications from the held-out dataset of 370 samples. True sample labels are numbered on the edge of the diagram and their misclassified label is depicted as the target of the corresponding ribbon. For example, the GNN model misclassified 0 colorectal samples; however, it misclassified 2 stomach samples, 2 kidney samples and 1 pancreas sample as colorectal.

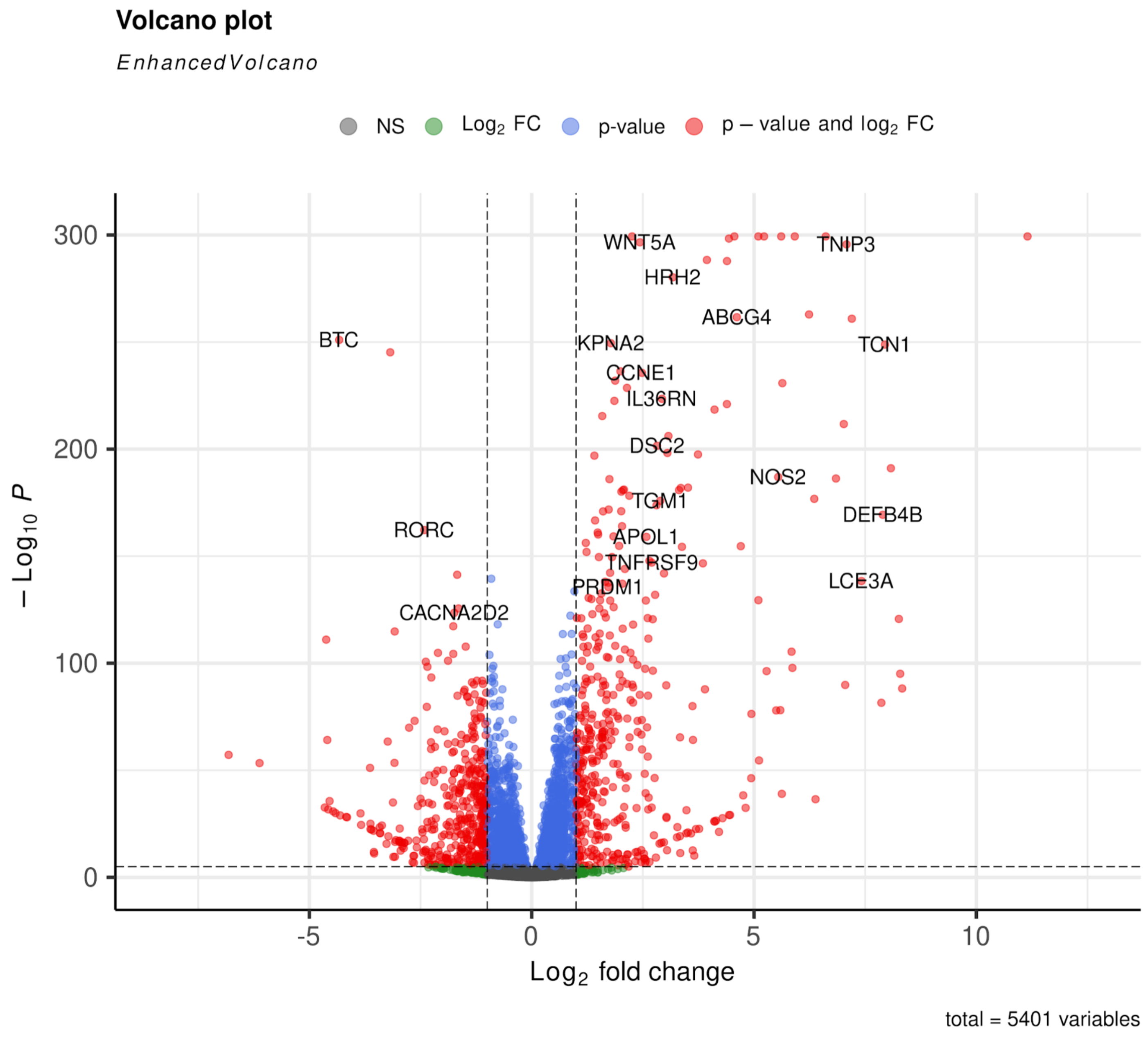


**Supplementary Figure 3: EnhancedVolcano Plot for SRP035988 using Default Parameters**

Volcano plot generated using the EnhancedVolcano package showing significantly differentially expressed genes found in the SRP035988 dataset using default parameters. Genes reported in prior literature as associated with psoriasis (Table S2) are labeled.

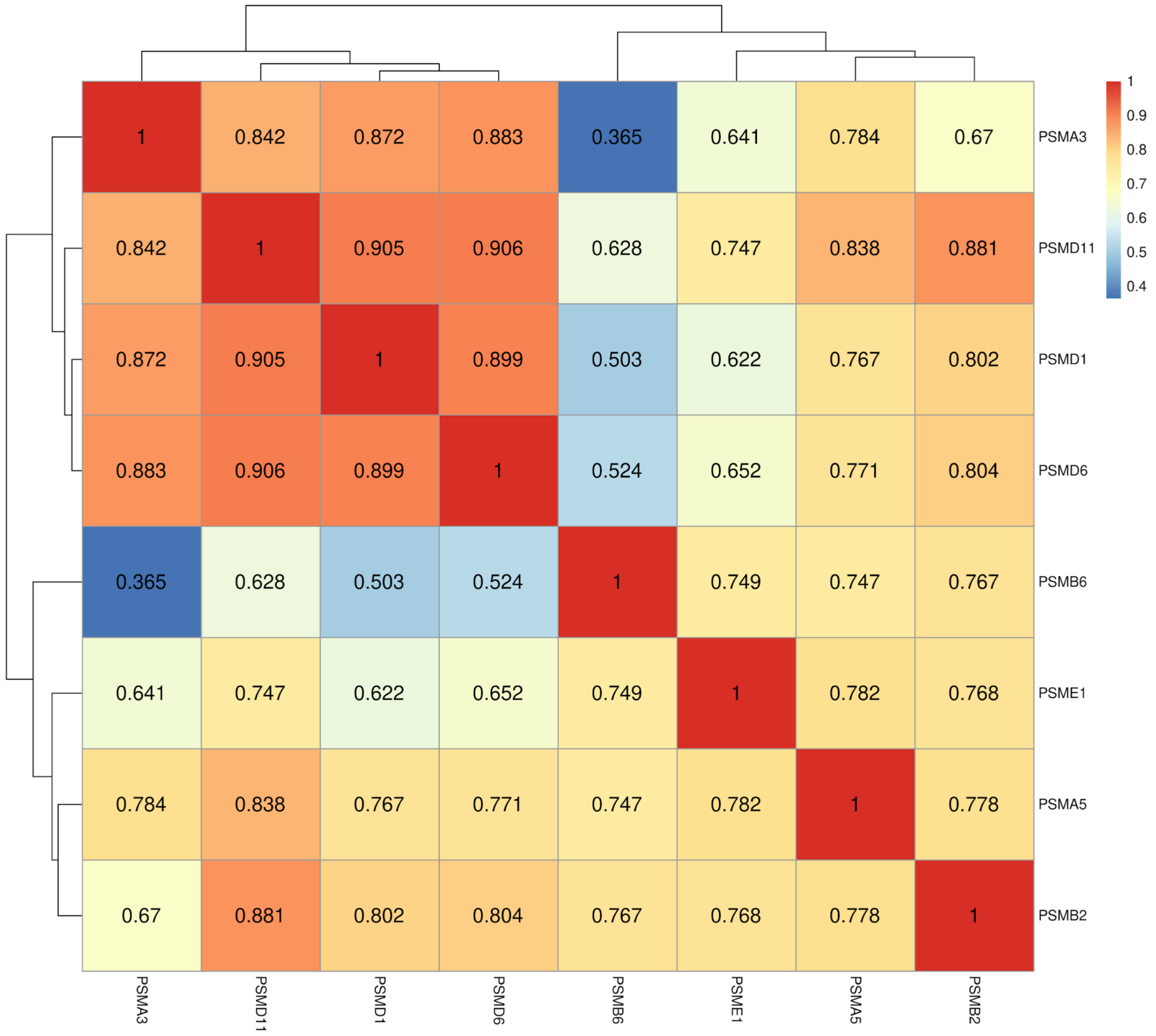


**Supplementary Figure 4: R-HSA-8956184 Differentially Expressed Gene Correlation**

Pearson correlation coefficients of significantly differentially expressed genes of R-HSA-8956184 form two distinct clusters.

# Supplementary Tables

**Supplementary Table 1: TCGA Misclassification Sample Counts**

Table showing TCGA tissue misclassification counts from the held-out dataset of 370 samples for each model.

| TCGA Tissue Label | # Miscalled by both models | # Miscalled by Resnet only | # Miscalled by GNN only |
|---|---|---|---|
| Adrenal Gland | 0 | 0 | 1 |
| Bile Duct | 4 | 1 | 0 |

| | | | |
|---|---|---|---|
| Bladder | 3 | 6 | 0 |
| Brain | 0 | 2 | 0 |
| Breast | 2 | 4 | 5 |
| Cervix | 1 | 0 | 0 |
| Colorectal | 0 | 2 | 0 |
| Esophagus | 1 | 2 | 1 |
| Head and Neck | 3 | 4 | 3 |
| Kidney | 1 | 0 | 3 |
| Liver | 0 | 3 | 4 |
| Lung | 0 | 4 | 5 |
| Pancreas | 2 | 0 | 0 |
| Prostate | 0 | 1 | 2 |
| Soft Tissue | 1 | 0 | 0 |
| Stomach | 2 | 3 | 3 |
| Thymus | 1 | 0 | 0 |
| Thyroid | 0 | 0 | 1 |
| Uterus | 1 | 3 | 3 |
| (Total) | 22 | 35 | 31 |

### Supplementary Table 2: Significant Differentially Expressed Genes of EnhancedVolcano

Significantly differentially expressed genes found in the SRP035988 dataset using default parameters of EnhancedVolcano reported in prior literature as associated with psoriasis.

| Gene Name | Ensembl ID | Overexpression Group | Supporting Literature PMID |
|---|---|---|---|
| WNT5A | ENSG00000114251 | Lesional psoriatic skin | 31313518 |
| TNIP3 | ENSG00000050730 | Lesional psoriatic skin | 25521225 |
| HRH2 | ENSG00000113749 | Lesional psoriatic skin | 29317264 |
| ABCG4 | ENSG00000172350 | Lesional psoriatic skin | 25505559 |
| BTC | ENSG00000174808 | Normal skin | 20220767 |
| KPNA2 | ENSG00000182481 | Lesional psoriatic skin | 24098495 |
| TCN1 | ENSG00000134827 | Lesional psoriatic skin | 23915137 |
| CCNE1 | ENSG00000105173 | Lesional psoriatic skin | 21711342 |
| IL36RN | ENSG00000136695 | Lesional psoriatic skin | 24129779 |
| DSC2 | ENSG00000134755 | Lesional psoriatic skin | 33995349 |
| NOS2 | ENSG00000007171 | Lesional psoriatic skin | 25539641 |
| TGM1 | ENSG00000092295 | Lesional psoriatic skin | 32106600 |
| RORC | ENSG00000143365 | Normal skin | 26149470 |
| APOL1 | ENSG00000100342 | Lesional psoriatic skin | 29204449 |
| DEFB4B | ENSG00000177257 | Lesional psoriatic skin | 32484435 |
| TNFRSF9 | ENSG00000049249 | Lesional psoriatic skin | 24885462 |
| PRDM1 | ENSG00000057657 | Lesional psoriatic skin | 23576729 |
| LCE3A | ENSG00000185962 | Lesional psoriatic skin | 28634035 |
| CACNA2D2 | ENSG00000007402 | Normal skin | 31611939 |

**Supplementary Table 3: Loci and TFs of Differentially Expressed Genes in R-HSA-8956184**

HGNC (Tweedie et al. 2021) bands and top five promoters/enhancers by GeneHancer Score (Fishilevich et al. 2017) of significantly differentially expressed genes of R-HSA-8956184.

| Gene Name | Ensembl ID | HGNC Band | Cluster | Promoter/Enhancer List |
|---|---|---|---|---|
| PSMD11 | ENSG00000108671 | 17q11.2 | 1 | GH17J032348, GH17J032485, GH17J032443, GH17J031548, GH17J032302 |
| PSMA5 | ENSG00000143106 | 1p13.3 | 2 | GH01J109389, GH01J109423, GH01J109438, GH01J109441, GH01J109466 |
| PSME1 | ENSG00000092010 | 14q12 | 2 | GH14J024422, GH14J024240, GH14J024270, GH14J024139, GH14J024135 |
| PSMB2 | ENSG00000126067 | 1p34.3 | 2 | GH01J035717, GH01J035636, GH01J035707, GH01J035715, GH01J035577 |
| PSMA3 | ENSG00000100567 | 14q23.1 | 1 | GH14J058242, GH14J058199, GH14J058238, GH14J058271, GH14J058280 |
| PSMD1 | ENSG00000173692 | 2q37.1 | 1 | GH02J230863, GH02J230711, GH02J231056, GH02J231407, GH02J231052 |
| PSMD6 | ENSG00000163636 | 3p14.1 | 1 | GH03J063910, GH03J064021, GH03J064219, GH03J063966, GH03J064264 |
| PSMB6 | ENSG00000142507 | 17p13.2 | 2 | GH17J004795, GH17J004843, GH17J004842, GH17J004791, GH17J004801 |

## Ancillary R Package References


1. Azzalini A, Menardi G. Clustering via nonparametric density estimation: The R package pdfCluster. arXiv preprint arXiv:1301.6559. 2013 Jan 28. doi: https://doi.org/10.48550/arXiv.1301.6559
2. Bache SM and Wickham H, (2022). magrittr: A Forward-Pipe Operator for R. R package version 2.0.2. https://CRAN.R-project.org/package=magrittr
3. Chen H, (2022). VennDiagram: Generate High-Resolution Venn and Euler Plots. R package version 1.7.3. https://CRAN.R-project.org/package=VennDiagram
4. Dewey M, (2022). metap: meta-analysis of significance values. R package version 1.8.
5. Donaldson J, (2022). tsne: T-Distributed Stochastic Neighbor Embedding for R (t-SNE). R package version 0.1-3.1. https://CRAN.R-project.org/package=tsne
6. Durinck S, Spellman PT, Birney E, Huber W. Mapping identifiers for the integration of genomic datasets with the R/Bioconductor package biomaRt. Nature protocols. 2009 Aug;4(8):1184-91.
7. Eckert A, (2022). parallelDist: Parallel Distance Matrix Computation using Multiple Threads. R package version 0.2.6. https://CRAN.R-project.org/package=parallelDist
8. Fishilevich S, Nudel R, Rappaport N, Hadar R, Plaschkes I, Iny Stein T, Rosen N, Kohn A, Twik M, Safran M, Lancet D. GeneHancer: genome-wide integration of enhancers and target genes in GeneCards. Database. 2017 Jan 1;2017.
9. Flor M, (2022). chorddiag: Interactive Chord Diagrams. R package version 0.1.3. https://github.com/mattflor/chorddiag/
10. Garnier S, Ross N, Rudis R, Camargo AP, Sciaini M, and Scherer C, (2021). Rvision - Colorblind-Friendly Color Maps for R. R package version 0.6.2
11. Gohel D and Skintzos P, (2022). ggiraph: Make 'ggplot2' Graphics Interactive. R package version 0.8.3. https://CRAN.R-project.org/package=ggiraph
12. Gu Z, Gu L, Eils R, Schlesner M, Brors B. Circlize implements and enhances circular visualization in R. Bioinformatics. 2014 Oct 1;30(19):2811-2.

13. Hatje K, Rahman RU, Vidal RO, Simm D, Hammesfahr B, Bansal V, Rajput A, Mickael ME, Sun T, Bonn S, Kollmar M. The landscape of human mutually exclusive splicing. Molecular systems biology. 2017 Dec;13(12):959.
14. Hosmer DW, Lemeshow S. Applied Logistic Regression, 2nd Ed. Chapter 5. New York, NY: John Wiley and Sons, 2000. Pp. 160 –164.
15. Hočevar T, Demšar J. Computation of graphlet orbits for nodes and edges in sparse graphs. Journal of Statistical Software. 2016 Jul 28;71:1-24.
16. Huber W, Carey VJ, Gentleman R, Anders S, Carlson M, Carvalho BS, Bravo HC, Davis S, Gatto L, Girke T, Gottardo R. Orchestrating high-throughput genomic analysis with Bioconductor. Nature methods. 2015 Feb;12(2):115-21.
17. Kassambara A and Mundt F, (2020). factoextra: Extract and Visualize the Results of Multivariate Data Analyses. R package version 1.0.7. https://CRAN.R-project.org/package=factoextra
18. Kolde R, (2019). pheatmap: Pretty Heatmaps. R package version 1.0.12. https://CRAN.R-project.org/package=pheatmap
19. Konopka T, (2022). umap: Uniform Manifold Approximation and Projection. R package version 0.2.9.0. https://CRAN.R-project.org/package=umap
20. Kuhn M. Caret: classification and regression training. Astrophysics Source Code Library. 2015 May:ascl-1505.
21. Mandrekar JN. Receiver operating characteristic curve in diagnostic test assessment. Journal of Thoracic Oncology. 2010 Sep 1;5(9):1315-6.
22. Mecham BH, Nelson PS, Storey JD. Supervised normalization of microarrays. Bioinformatics. 2010 May 15;26(10):1308-15.
23. Microsoft and Weston S, (2022). foreach: Provides Foreach Looping Construct. R package version 1.5.2. https://CRAN.R-project.org/package=foreach
24. Microsoft Corporation and Weston S, (2022). doParallel: Foreach Parallel Adaptor for the 'parallel' Package. R package version 1.0.17. https://CRAN.R-project.org/package=doParallel
25. Morgan M, Obenchain V, Hester J and Pagès H, (2021). SummarizedExperiment: SummarizedExperiment container. R package version 1.24.0. https://bioconductor.org/packages/SummarizedExperiment
26. Neuwirth E, (2022). RColorBrewer: ColorBrewer Palettes. R package version 1.1-3. https://CRAN.R-project.org/package=RColorBrewer
27. Pedersen TL, (2022). patchwork: The Composer of Plots. R package version 1.1.2. https://CRAN.R-project.org/package=patchwork
28. Ritchie ME, Phipson B, Wu DI, Hu Y, Law CW, Shi W, Smyth GK. limma powers differential expression analyses for RNA-sequencing and microarray studies. Nucleic acids research. 2015 Apr 20;43(7):e47-.
29. Rudis B, (2020). hrbrthemes: Additional Themes, Theme Components and Utilities for 'ggplot2'. R package version 0.8.0. https://CRAN.R-project.org/package=hrbrthemes
30. Sievert C. Interactive web-based data visualization with R, plotly, and shiny. CRC Press; 2020 Jan 30.
31. Signorell A, Aho K, Alfons A, Anderegg N, Aragon T, Arppe A, Baddeley A, Barton K, Bolker B, Borchers HW. DescTools: Tools for descriptive statistics. R package version 0.99. 2019;28:17.
32. Tang Y, Horikoshi M, Li W. ggfortify: unified interface to visualize statistical results of popular R packages. R J.. 2016 Dec 1;8(2):474.
33. Tweedie S, Braschi B, Gray K, Jones TE, Seal RL, Yates B, Bruford EA. Genenames. org: the HGNC and VGNC resources in 2021. Nucleic acids research. 2021 Jan 8;49(D1):D939-46.
34. Venables WN, Ripley BD, (2002). Modern Applied Statistics with S, Fourth edition. Springer, New York. ISBN 0-387-95457-0, https://www.stats.ox.ac.uk/pub/MASS4/.
35. Wei R and Wang J, (2018). multiROC: Calculating and Visualizing ROC and PR Curves Across Multi-Class Classifications. R package version 1.1.1. https://CRAN.R-project.org/package=multiROC
36. Wickham H, (2022). stringr: Simple, Consistent Wrappers for Common String Operations. R package version 1.4.1. https://CRAN.R-project.org/package=stringr

37. Wickham H, Averick M, Bryan J, Chang W, McGowan LD, François R, Grolemund G, Hayes A, Henry L, Hester J, Kuhn M. Welcome to the Tidyverse. Journal of open source software. 2019 Nov 21;4(43):1686.
38. Wickham H, François R, Henry L and Müller K, (2022). dplyr: A Grammar of Data Manipulation. R package version 1.0.10. https://CRAN.R-project.org/package=dplyr
39. Wickham H. Package 'ggplot2': elegant graphics for data analysis. Springer-Verlag New York. doi. 2016;10:978-0.
40. Wickham H. Reshaping data with the reshape package. Journal of statistical software. 2007 Nov 13;21:1-20.
41. Wickham H. The split-apply-combine strategy for data analysis. Journal of statistical software. 2011 Apr 7;40:1-29.
42. Wickham H and Girlich M, (2022). tidyr: Tidy Messy Data. R package version 1.2.1. https://CRAN.R-project.org/package=tidyr
43. Wickham H and Seidel D, (2022). scales: Scale Functions for Visualization. R package version 1.2.1. https://CRAN.R-project.org/package=scales
