# Supplementary material for: Biology-inspired graph neural network encodes reactome and reveals biochemical reactions of disease
Source: Patterns (N Y). 2023 May 22;4(7):100758. doi: 10.1016/j.patter.2023.100758 (PMC10382942; doi:10.1016/j.patter.2023.100758)
Supplement: Data S6. Reactome pathway default threshold enrichment report [file mmc7.pdf]

reactome

Pathway Analysis Report

This report contains the pathway analysis results for the submitted sample ''. Analysis was performed against Reactome version 80 on 06/05/2022. The web link to these results is:

https://reactome.org/PathwayBrowser/#/ANALYSIS=MjAyMjA1MDYyMjI5MDFfMzEwMjg%3D

Please keep in mind that analysis results are temporarily stored on our server. The storage period depends on usage of the service but is at least 7 days. As a result, please note that this URL is only valid for a limited time period and it might have expired.

Table of Contents

1. Introduction
2. Properties
3. Genome-wide overview
4. Most significant pathways
5. Pathways details
6. Identifiers found
7. Identifiers not found

1. Introduction

Reactome is a curated database of pathways and reactions in human biology. Reactions can be considered as pathway 'steps'. Reactome defines a 'reaction' as any event in biology that changes the state of a biological molecule. Binding, activation, translocation, degradation and classical biochemical events involving a catalyst are all reactions. Information in the database is authored by expert biologists, entered and maintained by Reactome's team of curators and editorial staff. Reactome content frequently cross-references other resources e.g. NCBI, Ensembl, UniProt, KEGG (Gene and Compound), ChEBI, PubMed and GO. Orthologous reactions inferred from annotation for Homo sapiens are available for 17 non-human species including mouse, rat, chicken, puffer fish, worm, fly, yeast, rice, and Arabidopsis. Pathways are represented by simple diagrams following an SBGN-like format.

Reactome's annotated data describe reactions possible if all annotated proteins and small molecules were present and active simultaneously in a cell. By overlaying an experimental dataset on these annotations, a user can perform a pathway over-representation analysis. By overlaying quantitative expression data or time series, a user can visualize the extent of change in affected pathways and its progression. A binomial test is used to calculate the probability shown for each result, and the p-values are corrected for the multiple testing (Benjamini–Hochberg procedure) that arises from evaluating the submitted list of identifiers against every pathway.

To learn more about our Pathway Analysis, please have a look at our relevant publications:

Fabregat A, Sidiropoulos K, Garapati P, Gillespie M, Hausmann K, Haw R, … D'Eustachio P (2016). The reactome pathway knowledgebase. Nucleic Acids Research, 44(D1), D481–D487. https://doi.org/10.1093/nar/gkv1351.

Fabregat A, Sidiropoulos K, Viteri G, Forner O, Marin-Garcia P, Arnau V, … Hermjakob H (2017). Reactome pathway analysis: a high-performance in-memory approach. BMC Bioinformatics, 18.

2. Properties

- This is an **overrepresentation** analysis: A statistical (hypergeometric distribution) test that determines whether certain Reactome pathways are over-represented (enriched) in the submitted data. It answers the question ‘Does my list contain more proteins for pathway X than would be expected by chance?’ This test produces a probability score, which is corrected for false discovery rate using the Benjamani-Hochberg method.
- 135 out of 135 identifiers in the sample were found in Reactome, where 445 pathways were hit by at least one of them.
- All non-human identifiers have been converted to their human equivalent.
- This report is filtered to show only results for species 'Homo sapiens' and resource 'all resources'.
- The unique ID for this analysis (token) is MjAyMjA1MDYyMjI5MDFfMzEwMjg%3D. This ID is valid for at least 7 days in Reactome’s server. Use it to access Reactome services with your data.

3. Genome-wide overview

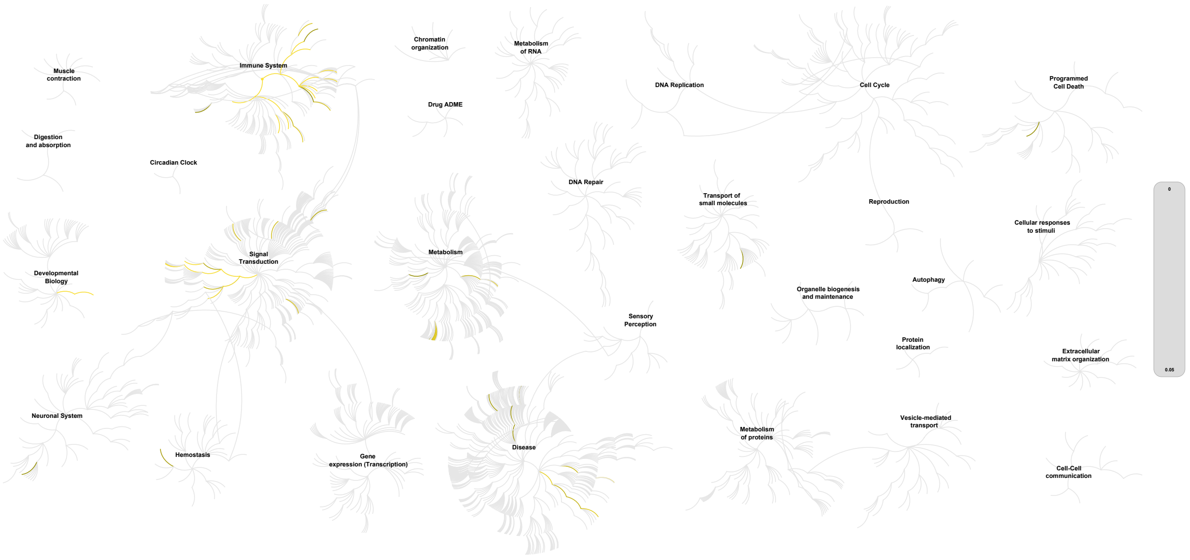

This figure shows a genome-wide overview of the results of your pathway analysis. Reactome pathways are arranged in a hierarchy. The center of each of the circular "bursts" is the root of one top-level pathway, for example "DNA Repair". Each step away from the center represents the next level lower in the pathway hierarchy. The color code denotes over-representation of that pathway in your input dataset. Light grey signifies pathways which are not significantly over-represented.

4. Most significant pathways

The following table shows the 25 most relevant pathways sorted by p-value.

Pathway name	Entities				Reactions	
	found	ratio	p-value	FDR*	found	ratio
Immune System	84 / 2,691	0.178	1.11e-16	5.04e-14	252 / 1,625	0.118
Formation of the cornified envelope	19 / 138	0.009	5.77e-15	1.31e-12	11 / 27	0.002
Cytokine Signaling in Immune system	45 / 1,094	0.072	5.26e-14	7.95e-12	140 / 710	0.051
Keratinization	19 / 226	0.015	2.82e-11	3.19e-09	18 / 34	0.002
Chemokine receptors bind chemokines	11 / 57	0.004	1.07e-10	9.67e-09	5 / 19	0.001
Signaling by Interleukins	29 / 645	0.043	4.53e-10	3.39e-08	101 / 493	0.036
Interleukin-10 signaling	12 / 86	0.006	5.77e-10	3.70e-08	2 / 15	0.001
Peptide ligand-binding receptors	14 / 203	0.013	1.41e-07	7.90e-06	8 / 77	0.006
Innate Immune System	37 / 1,338	0.089	5.63e-07	2.82e-05	87 / 710	0.051
Neutrophil degranulation	20 / 480	0.032	9.37e-07	4.22e-05	10 / 10	7.25e-04
Antimicrobial peptides	10 / 123	0.008	2.16e-06	8.88e-05	15 / 58	0.004
Interferon Signaling	17 / 395	0.026	4.22e-06	1.56e-04	27 / 71	0.005
Class A/1 (Rhodopsin-like receptors)	17 / 412	0.027	7.30e-06	2.39e-04	10 / 160	0.012
Interleukin-4 and Interleukin-13 signaling	12 / 211	0.014	7.92e-06	2.39e-04	9 / 47	0.003
Interferon gamma signaling	13 / 250	0.017	8.51e-06	2.39e-04	9 / 16	0.001
GPCR ligand binding	21 / 606	0.04	8.55e-06	2.39e-04	13 / 187	0.014
Interleukin-36 pathway	3 / 7	4.63e-04	8.23e-05	0.002	3 / 3	2.17e-04
Signaling by GPCR	23 / 871	0.058	2.03e-04	0.005	29 / 360	0.026
Calcitonin-like ligand receptors	3 / 11	7.28e-04	3.09e-04	0.007	2 / 4	2.90e-04
Interleukin-20 family signaling	4 / 29	0.002	3.95e-04	0.009	30 / 56	0.004
Interferon alpha/beta signaling	9 / 190	0.013	4.18e-04	0.009	11 / 24	0.002
Metal sequestration by antimicrobial proteins	3 / 13	8.61e-04	5.01e-04	0.01	3 / 5	3.62e-04
Dectin-2 family	5 / 65	0.004	0.001	0.02	11 / 12	8.70e-04
Inhibition of nitric oxide production	2 / 5	3.31e-04	0.002	0.029	2 / 5	3.62e-04
Acyl chain remodelling of PG	3 / 26	0.002	0.004	0.065	5 / 10	7.25e-04

* False Discovery Rate

5. Pathways details

For every pathway of the most significant pathways, we present its diagram, as well as a short summary, its bibliography and the list of inputs found in it.

1. Immune System (R-HSA-168256)

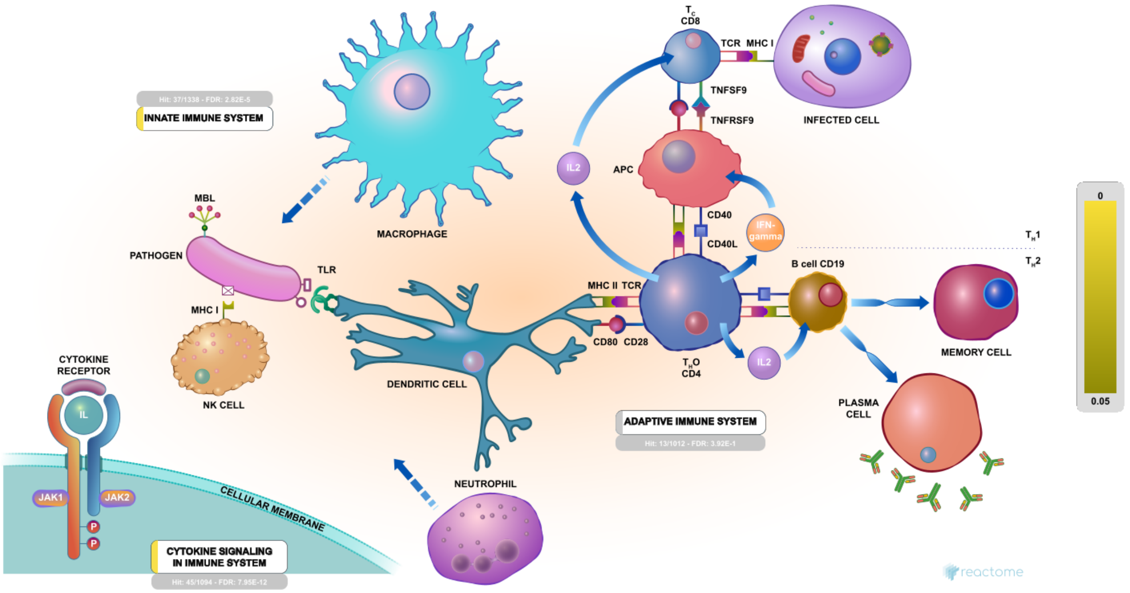

Humans are exposed to millions of potential pathogens daily, through contact, ingestion, and inhalation. Our ability to avoid infection depends on the adaptive immune system and during the first critical hours and days of exposure to a new pathogen, our innate immune system.

References

Edit history

Date	Action	Author
2005-11-11	Created	Gillespie ME
2006-03-30	Authored	Luo F, Ouwehand WH, Gillespie ME, de Bono B
2006-04-19	Reviewed	Zwaginga JJ, D'Eustachio P, Gay NJ, Gale M Jr
2022-03-23	Modified	Weiser JD

63 submitted entities found in this pathway, mapping to 87 Reactome entities

Input	UniProt Id	Input	UniProt Id	Input	UniProt Id
ENSG00000007171	P35228	ENSG00000049249	Q07011	ENSG00000057149	P29508
ENSG00000100453	P08311	ENSG00000103313	O15553	ENSG00000108688	P13500
ENSG00000110944	Q9NPF7	ENSG00000111331	Q9Y6K5	ENSG00000111335	P29728
ENSG00000112115	Q16552	ENSG00000112116	Q96PD4	ENSG00000112299	O95497
ENSG00000113302	P29460	ENSG00000113356	O15318	ENSG00000115415	P42224, P42224-1, P42224-2
ENSG00000120217	Q9NZQ7	ENSG00000124233	P04279	ENSG00000124256	Q9H171
ENSG00000124731	Q9NP99	ENSG00000126709	P09912	ENSG00000134827	P20061

Input	UniProt Id	Input	UniProt Id	Input	UniProt Id
ENSG00000136688	Q9NZH8	ENSG00000136694	Q9UHA7	ENSG00000136695	Q9UBH0
ENSG00000138642	Q8IVU3	ENSG00000139572	Q9NQS5	ENSG00000142224	Q9UHD0
ENSG00000143546	P05109	ENSG00000143556	P31151, Q86SG5	ENSG00000145113	Q99102
ENSG00000145287	Q9NZF1	ENSG00000150244	Q8IWZ4	ENSG00000150337	P12314, Q92637
ENSG00000156234	P02775	ENSG00000158125	P47989	ENSG00000162891	Q9NYY1
ENSG00000162892	Q13007	ENSG00000163568	O14862	ENSG00000163739	P09341
ENSG00000164047	P49913	ENSG00000166523	Q9ULY5	ENSG00000166527	Q8WXI8
ENSG00000167618	Q6ISS4	ENSG00000169245	P02778	ENSG00000171049	P21462, P25090
ENSG00000171051	P21462, P62942	ENSG00000176797	P81534	ENSG00000177243	P81534
ENSG00000177257	O15263	ENSG00000183347	Q6ZN66	ENSG00000186191	P59827
ENSG00000186431	P24071	ENSG00000188389	Q15116	ENSG00000188404	P14151
ENSG00000189013	Q99706	ENSG00000197249	P01009	ENSG00000198019	Q92637
ENSG00000198805	P00491	ENSG00000203747	P08637	ENSG00000205420	P04264
ENSG00000205846	Q6EIG7, Q8WTT0	ENSG00000214643	Q30KQ1	ENSG00000277632	P10147, P16619

Input	Ensembl Id	Input	Ensembl Id	Input	Ensembl Id
ENSG00000007171	ENSG00000007171	ENSG00000111331	ENSG00000111331	ENSG00000111335	ENSG00000111335
ENSG00000112115	ENSG00000112115	ENSG00000112116	ENSG00000112116	ENSG00000113302	ENSG00000113302
ENSG00000115415	ENSG00000115415	ENSG00000126709	ENSG00000126709	ENSG00000150244	ENSG00000150244
ENSG00000150337	ENSG00000150337	ENSG00000163739	ENSG00000163739	ENSG00000169245	ENSG00000169245
ENSG00000171051	ENSG00000171051	ENSG00000183347	ENSG00000183347	ENSG00000198019	ENSG00000198019
ENSG00000277632	ENSG00000277632				

2. Formation of the cornified envelope (R-HSA-6809371)

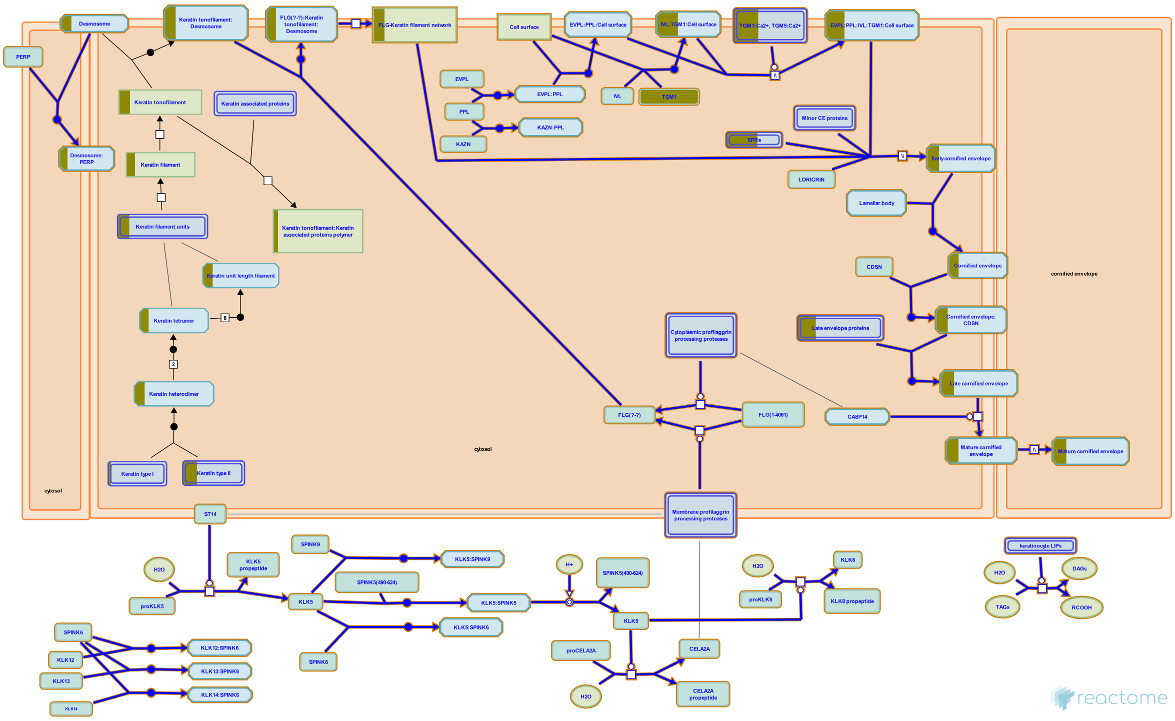

As keratinocytes progress towards the upper epidermis, they undergo a unique process of cell death termed cornification (Eckhart et al. 2013). This involves the crosslinking of keratinocyte proteins such as loricrin and involucrin by transglutaminases and the breakdown of the nucleus and other organelles by intracellular and secreted proteases (Eckhart et al. 2000, Denecker et al. 2008). This process is strictly regulated by the Ca2+ concentration gradient in the epidermis (Esholtz et al. 2014). Loricrin and involucrin are encoded in 'Epidermal Differentiation Complex' linked to a large number of genes encoding nonredundant components of the CE (Kypriotou et al. 2012, Niehues et al. 2016). Keratinocytes produce specialized proteins and lipids which are used to construct the cornified envelope (CE), a heavily crosslinked submembranous layer that confers rigidity to the upper epidermis, allows keratin filaments to attach to any location in the cell membrane (Kirfel et al. 2003) and acts as a water-impermeable barrier. The CE has two functional parts: covalently crosslinked proteins (10 nm thick) that comprise the backbone of the envelope and covalently linked lipids (5 nm thick) that coat the exterior (Eckert et al. 2005). Desmosomal components are crosslinked to the CE to form corneodesmosomes, which bind cornified cells together (Ishida-Yamamoto et al. 2011). Mature terminally differentiated cornified cells consist mostly of keratin filaments covalently attached to the CE embedded in lipid lamellae (Kalinin et al. 2002). The exact composition of the cornified envelope varies between epithelia (Steinert et al. 1998); the relative amino-acid composition of the proteins used may determine differential mechanical properties (Kartasova et al. 1996).

References

Candi E, Melino G & Schmidt R (2005). The cornified envelope: a model of cell death in the skin. Nat. Rev. Mol. Cell Biol., 6, 328-40.

Edit history

Date	Action	Author
2015-11-11	Created	Jupe S
2016-03-10	Authored	Jupe S
2016-08-10	Edited	Jupe S
2016-08-12	Reviewed	Blumenberg M
2022-03-23	Modified	Weiser JD

17 submitted entities found in this pathway, mapping to 20 Reactome entities

Input	UniProt Id	Input	UniProt Id	Input	UniProt Id
ENSG00000092295	P22735	ENSG00000134755	Q02487	ENSG00000134757	P32926
ENSG00000159516	Q9BYE4	ENSG00000170465	P04259, P48668	ENSG00000185069	Q01546
ENSG00000185962	Q5TA76	ENSG00000185966	Q5T5B0	ENSG00000186442	P12035
ENSG00000186832	P08779	ENSG00000187238	Q5TA77	ENSG00000196805	P35325
ENSG00000203785	P22531	ENSG00000205420	P02538, P04264, P48668	ENSG00000241794	P35326
ENSG00000244057	Q5T5A8	ENSG00000244094	Q96RM1		

3. Cytokine Signaling in Immune system (R-HSA-1280215)

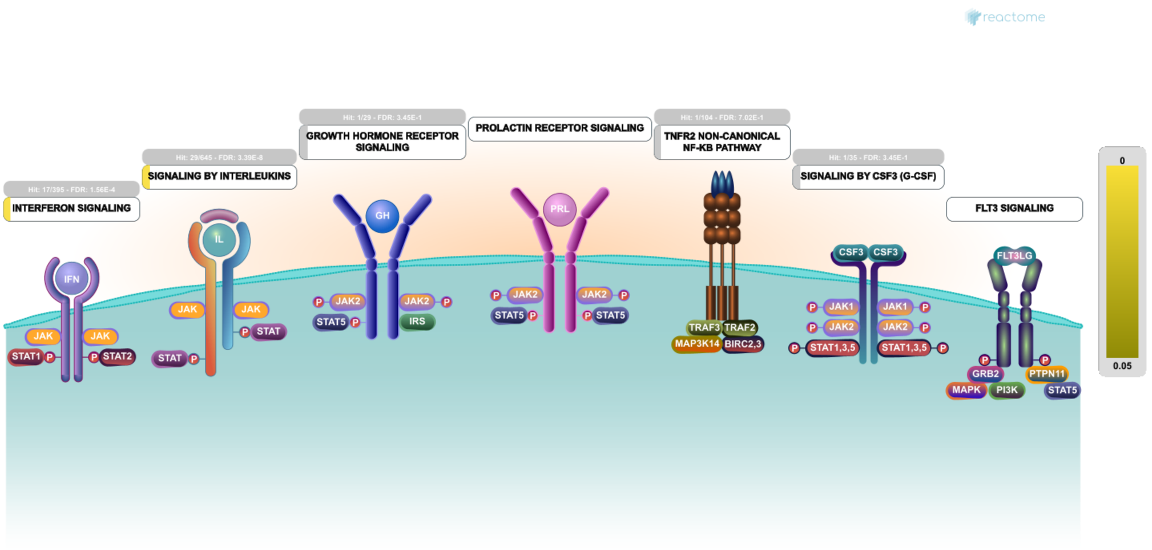

Cytokines are small proteins that regulate and mediate immunity, inflammation, and hematopoiesis. They are secreted in response to immune stimuli, and usually act briefly, locally, at very low concentrations. Cytokines bind to specific membrane receptors, which then signal the cell via second messengers, to regulate cellular activity.

References

Feldmann M & Oppenheim J (2002). *Cytokines and the immune system, Cytokine Reference* .

IMMPORT:Bioinformatics for the future of immunology. Retrieved from https://www.immport.org/immportWeb/queryref/geneListSummary.do

Santamaria P (2003). Cytokines and chemokines in autoimmune disease: an overview. Adv Exp Med Biol, 520, 1-7.

COPE. Retrieved from http://www.copewithcytokines.org/cope.cgi

Edit history

Date	Action	Author
2011-05-12	Created	Garapati P V
2011-05-22	Edited	Ray KP, Jupe S, Garapati P V
2011-05-22	Authored	Ray KP, Jupe S, Garapati P V
2011-05-28	Reviewed	Abdul-Sater AA, Schindler C, Pinteaux E
2022-03-23	Modified	Weiser JD

27 submitted entities found in this pathway, mapping to 47 Reactome entities

Input	UniProt Id	Input	UniProt Id	Input	UniProt Id
ENSG00000007171	P35228	ENSG00000049249	Q07011	ENSG00000100453	P08311
ENSG00000108688	P13500	ENSG00000110944	Q9NPF7	ENSG00000111331	Q9Y6K5
ENSG00000111335	P29728	ENSG00000112115	Q16552	ENSG00000112116	Q96PD4

Input	UniProt Id	Input	UniProt Id	Input	UniProt Id
ENSG00000113302	P29460	ENSG00000115415	P42224, P42224-1, P42224-2	ENSG00000126709	P09912
ENSG00000136688	Q9NZH8	ENSG00000136694	Q9UHA7	ENSG00000136695	Q9UBH0
ENSG00000142224	Q9UHD0	ENSG00000150244	Q8IWZ4	ENSG00000150337	P12314, Q92637
ENSG00000162891	Q9NYY1	ENSG00000162892	Q13007	ENSG00000163739	P09341
ENSG00000169245	P02778	ENSG00000171049	P21462	ENSG00000171051	P21462
ENSG00000183347	Q6ZN66	ENSG00000198019	Q92637	ENSG00000277632	P10147, P16619

Input	Ensembl Id	Input	Ensembl Id	Input	Ensembl Id
ENSG00000007171	ENSG00000007171	ENSG00000111331	ENSG00000111331	ENSG00000111335	ENSG00000111335
ENSG00000112115	ENSG00000112115	ENSG00000112116	ENSG00000112116	ENSG00000113302	ENSG00000113302
ENSG00000115415	ENSG00000115415	ENSG00000126709	ENSG00000126709	ENSG00000150244	ENSG00000150244
ENSG00000150337	ENSG00000150337	ENSG00000163739	ENSG00000163739	ENSG00000169245	ENSG00000169245
ENSG00000171051	ENSG00000171051	ENSG00000183347	ENSG00000183347	ENSG00000198019	ENSG00000198019
ENSG00000277632	ENSG00000277632				

4. Keratinization (R-HSA-6805567)

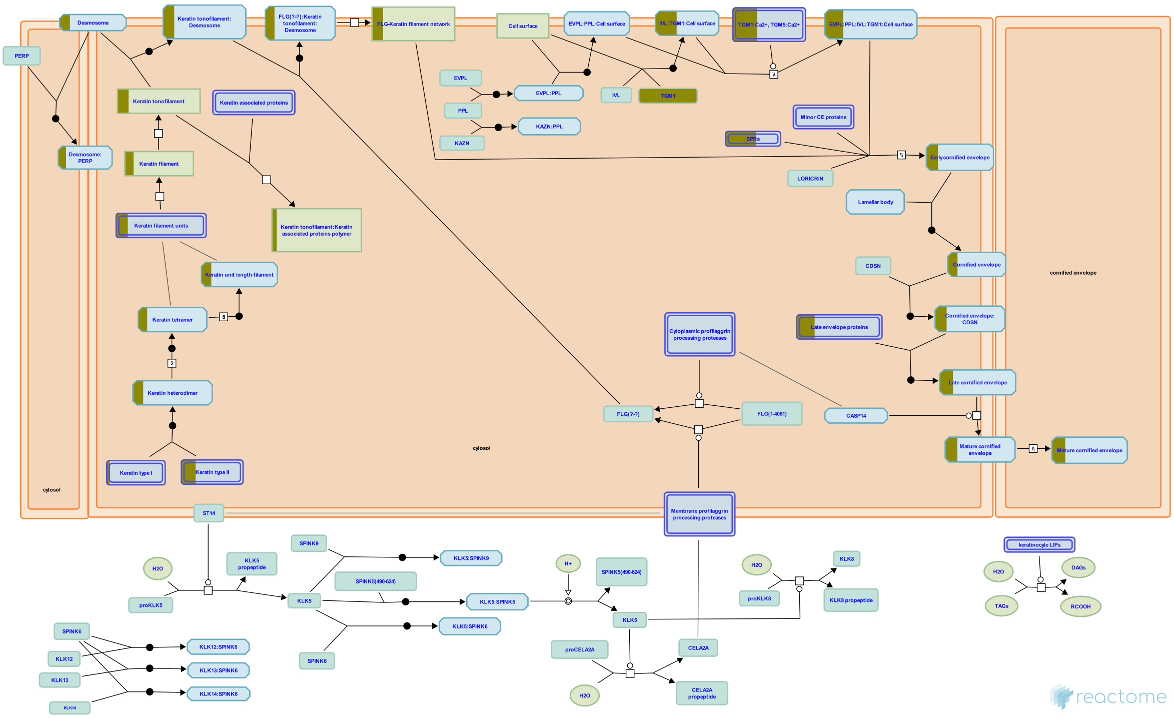

Keratins are the major structural protein of vertebrate epidermis, constituting up to 85% of a fully differentiated keratinocyte (Fuchs 1995). Keratins belong to a superfamily of intermediate filament (IF) proteins that form alpha-helical coiled-coil dimers, which associate laterally and end-to-end to form approximately 10 nm diameter filaments. Keratin filaments are heteropolymeric, formed from equal amounts of acidic type I and basic /neutral type 2 keratins. Humans have 54 keratin genes (Schweitzer et al. 2006). They have highly specific expression patterns, related to the epithelial type and stage of differentiation. Roughly half of human keratins are specific to hair follicles (Langbein & Schweizer 2005). Keratin filaments bundle into tonofilaments that span the cytoplasm and bind to desmosomes and other cell membrane structures (Waschke 2008). This reflects their primary function, maintaining the mechanical stability of individual cells and epithelial tissues (Moll et al. 2008).

References

Divo M, Langbein L & Moll R (2008). The human keratins: biology and pathology. Histochem. Cell Biol., 129, 705-33.

Edit history

Date	Action	Author
2015-10-20	Created	Jupe S
2016-03-10	Authored	Jupe S
2016-08-10	Edited	Jupe S
2016-08-12	Reviewed	Blumenberg M
2022-03-23	Modified	Weiser JD

17 submitted entities found in this pathway, mapping to 20 Reactome entities

Input	UniProt Id	Input	UniProt Id	Input	UniProt Id
ENSG00000092295	P22735	ENSG00000134755	Q02487	ENSG00000134757	P32926
ENSG00000159516	Q9BYE4	ENSG00000170465	P04259, P48668	ENSG00000185069	Q01546
ENSG00000185962	Q5TA76	ENSG00000185966	Q5T5B0	ENSG00000186442	P12035
ENSG00000186832	P08779	ENSG00000187238	Q5TA77	ENSG00000196805	P35325
ENSG00000203785	P22531	ENSG00000205420	P02538, P04264, P48668	ENSG00000241794	P35326
ENSG00000244057	Q5T5A8	ENSG00000244094	Q96RM1		

5. Chemokine receptors bind chemokines (R-HSA-380108)

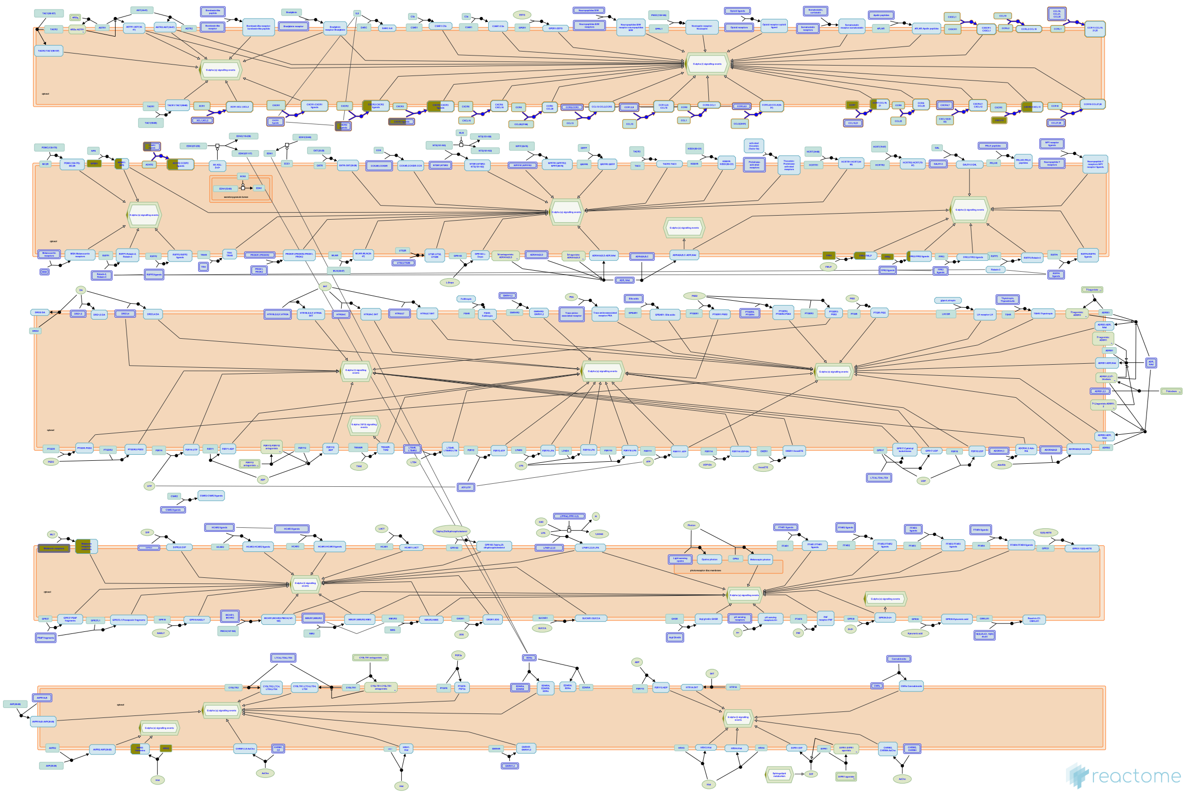

Chemokine receptors are cytokine receptors found on the surface of certain cells, which interact with a type of cytokine called a chemokine. Following interaction, these receptors trigger a flux of intracellular calcium which leads to chemotaxis. Chemokine receptors are divided into different families, CXC chemokine receptors, CC chemokine receptors, CX3C chemokine receptors and XC chemokine receptors that correspond to the 4 distinct subfamilies of chemokines they bind.

References

Finn A & Murdoch C (2000). Chemokine receptors and their role in inflammation and infectious diseases. Blood, 95, 3032-43.

Kim CH (2004). Chemokine-chemokine receptor network in immune cell trafficking. Curr Drug Targets Immune Endocr Metabol Disord, 4, 343-61.

Horuk R (2001). Chemokine receptors. Cytokine Growth Factor Rev, 12, 313-35.

Edit history

Date	Action	Author
2008-11-07	Authored	Jassal B
2008-11-07	Created	Jassal B
2022-03-23	Modified	Weiser JD

8 submitted entities found in this pathway, mapping to 11 Reactome entities

Input	UniProt Id	Input	UniProt Id	Input	UniProt Id
ENSG00000108688	P13500, P80098	ENSG00000126353	P32248	ENSG00000156234	O43927, P02775
ENSG00000163735	P42830	ENSG00000163739	P09341	ENSG00000169245	P02778

Input	UniProt Id	Input	UniProt Id	Input	UniProt Id
ENSG00000169248	O14625	ENSG00000277632	P10147, P16619		

6. Signaling by Interleukins (R-HSA-449147)

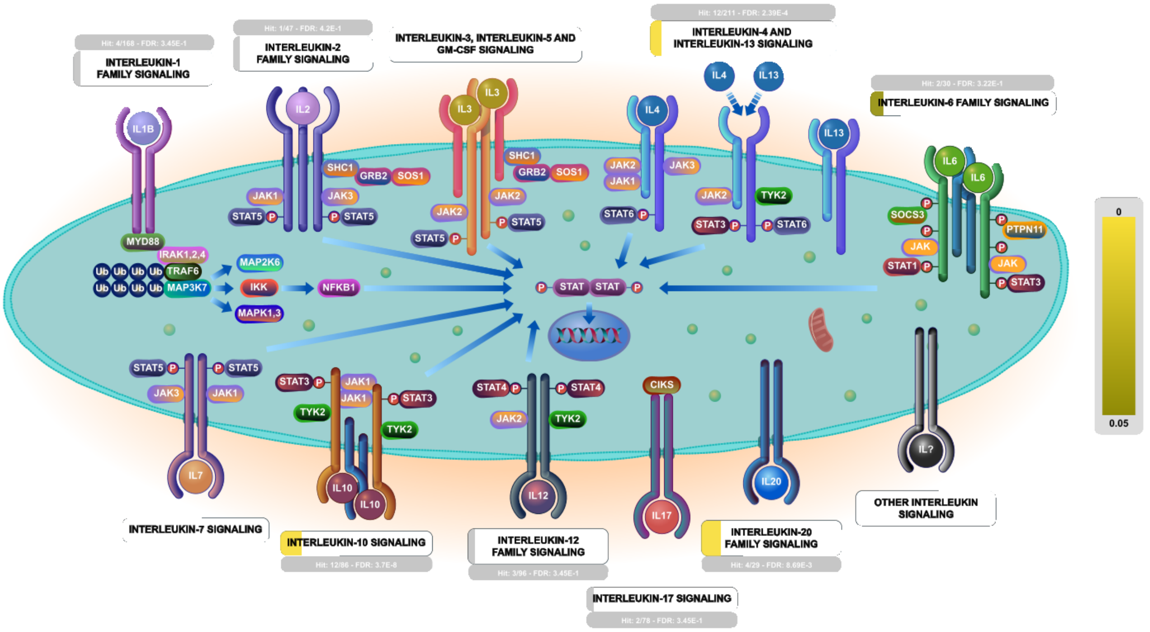

Cellular compartments: plasma membrane.

Interleukins are low molecular weight proteins that bind to cell surface receptors and act in an autocrine and/or paracrine fashion. They were first identified as factors produced by leukocytes but are now known to be produced by many other cells throughout the body. They have pleiotropic effects on cells which bind them, impacting processes such as tissue growth and repair, hematopoietic homeostasis, and multiple levels of the host defense against pathogens where they are an essential part of the immune system.

References

Dinarello CA (2009). Immunological and inflammatory functions of the interleukin-1 family. Annu Rev Immunol, 27, 519-50.

Komlosi Z, Kucuksezer UC, Frei R, Huitema C, Garbani M, Pezer M, ... Eiwegger T (2016). Interleukins (from IL-1 to IL-38), interferons, transforming growth factor , and TNF-: Receptors, functions, and roles in diseases. J. Allergy Clin. Immunol., 138, 984-1010.

Vosshenrich CA & Di Santo JP (2002). Interleukin signaling. Curr Biol, 12, R760-3.

Edit history

Date	Action	Author
2009-11-27	Created	Jupe S
2010-05-17	Reviewed	Pinteaux E
2010-05-17	Authored	Ray KP
2010-05-26	Edited	Jupe S
2022-03-23	Modified	Weiser JD

19 submitted entities found in this pathway, mapping to 30 Reactome entities

Input	UniProt Id	Input	UniProt Id	Input	UniProt Id
ENSG00000007171	P35228	ENSG00000100453	P08311	ENSG00000108688	P13500
ENSG00000110944	Q9NPF7	ENSG00000112115	Q16552	ENSG00000112116	Q96PD4
ENSG00000113302	P29460	ENSG00000115415	P42224, P42224-1	ENSG00000136688	Q9NZH8
ENSG00000136694	Q9UHA7	ENSG00000136695	Q9UBH0	ENSG00000142224	Q9UHD0
ENSG00000162891	Q9NYY1	ENSG00000162892	Q13007	ENSG00000163739	P09341
ENSG00000169245	P02778	ENSG00000171049	P21462	ENSG00000171051	P21462
ENSG00000277632	P10147, P16619				

Input	Ensembl Id	Input	Ensembl Id	Input	Ensembl Id
ENSG00000007171	ENSG00000007171	ENSG00000112115	ENSG00000112115	ENSG00000112116	ENSG00000112116
ENSG00000113302	ENSG00000113302	ENSG00000115415	ENSG00000115415	ENSG00000163739	ENSG00000163739
ENSG00000169245	ENSG00000169245	ENSG00000171051	ENSG00000171051	ENSG00000277632	ENSG00000277632

7. Interleukin-10 signaling (R-HSA-6783783)

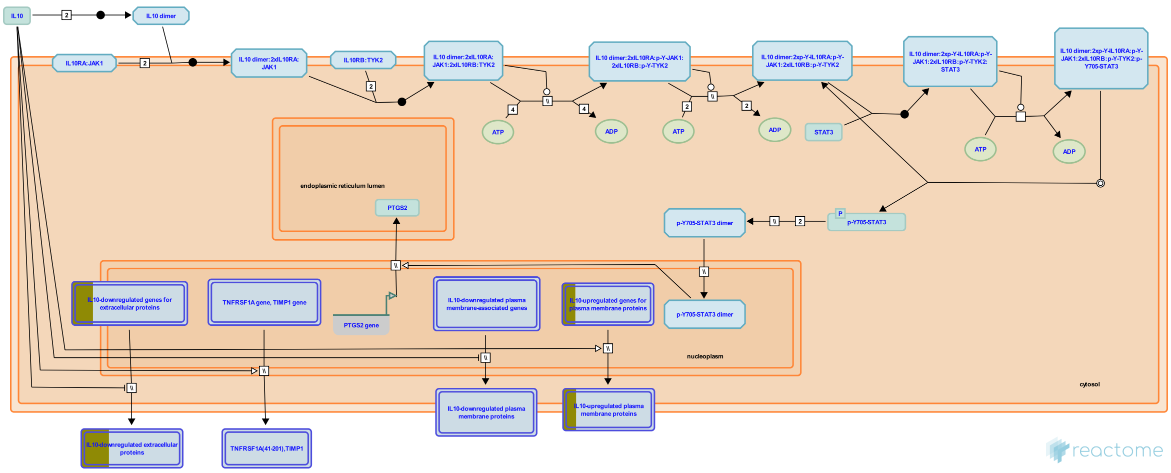

Interleukin-10 (IL10) was originally described as a factor named cytokine synthesis inhibitory factor that inhibited T-helper (Th) 1 activation and Th1 cytokine production (Fiorentino et al. 1989). It was found to be expressed by a variety of cell types including macrophages, dendritic cell subsets, B cells, several T-cell subpopulations including Th2 and T-regulatory cells (Tregs) and Natural Killer (NK) cells (Moore et al. 2001). It is now recognized that the biological effects of IL10 are directed at antigen-presenting cells (APCs) such as macrophages and dendritic cells (DCs), its effects on T-cell development and differentiation are largely indirect via inhibition of macrophage/dendritic cell activation and maturation (Pestka et al. 2004, Mocellin et al. 2004). T cells are thought to be the main source of IL10 (Hedrich & Bream 2010). IL10 inhibits a broad spectrum of activated macrophage/monocyte functions including monokine synthesis, NO production, and expression of class II MHC and costimulatory molecules such as IL12 and CD80/CD86 (de Waal Malefyt et al. 1991, Gazzinelli et al. 1992). Studies with recombinant cytokine and neutralizing antibodies revealed pleiotropic activities of IL10 on B, T, and mast cells (de Waal Malefyt et al. 1993, Rousset et al. 1992, Thompson-Snipes et al. 1991) and provided evidence for the in vivo significance of IL10 activities (Ishida et al. 1992, 1993). IL10 antagonizes the expression of MHC class II and the co-stimulatory molecules CD80/CD86 as well as the pro-inflammatory cytokines IL1Beta, IL6, IL8, TNFalpha and especially IL12 (Fiorentino et al. 1991, D'Andrea et al. 1993). The biological role of IL10 is not limited to inactivation of APCs, it also enhances B cell, granulocyte, mast cell, and keratinocyte growth/differentiation, as well as NK-cell and CD8+ cytotoxic T-cell activation (Moore et al. 2001, Hedrich & Bream 2010). IL10 also enhances NK-cell proliferation and/or production of IFN-gamma (Cai et al. 1999).

IL10-deficient mice exhibited inflammatory bowel disease (IBD) and other exaggerated inflammatory responses (Kuhn et al. 1993, Berg et al. 1995) indicating a critical role for IL10 in limiting inflammatory responses. Dysregulation of IL10 is linked with susceptibility to numerous infectious and autoimmune diseases in humans and mouse models (Hedrich & Bream 2010).

IL10 signaling is initiated by binding of homodimeric IL10 to the extracellular domains of two adjoining IL10RA molecules. This tetramer then binds two IL10RB chains. IL10RB cannot bind to IL10 unless bound to IL10RA (Ding et al. 2001, Yoon et al. 2006); binding of IL10 to IL10RA without the co-presence of IL10RB fails to initiate signal transduction (Kotenko et al. 1997).

IL10 binding activates the receptor-associated Janus tyrosine kinases, JAK1 and TYK2, which are constitutively bound to IL10R1 and IL10R2 respectively. In the classic model of receptor activation assembly of the receptor complex is believed to enable JAK1/TYK2 to phosphorylate and activate each other. Alternatively the binding of IL10 may cause conformational changes that allow the pseudokinase inhibitory domain of one JAK kinase to move away from the kinase domain of the other JAK within the receptor dimer-JAK complex, allowing the two kinase domains to interact and trans-activate (Waters & Brooks 2015).

The activated JAK kinases phosphorylate the intracellular domains of the IL10R1 chains on specific tyrosine residues. These phosphorylated tyrosine residues and their flanking peptide sequences serve as temporary docking sites for the latent, cytosolic, transcription factor, STAT3. STAT3 transiently docks on the IL10R1 chain via its SH2 domain, and is in turn tyrosine phosphorylated by the receptor-associated JAKs. Once activated, it dissociates from the receptor, dimerizes with other STAT3 molecules, and translocates to the nucleus where it binds with high affinity to STAT-binding elements (SBEs) in the promoters of IL-10-inducible genes (Donnelly et al. 1999).

References

Moore KW, O'Garra A, Coffman RL & de Waal Malefyt R (2001). Interleukin-10 and the interleukin-10 receptor. Annu. Rev. Immunol., 19, 683-765.

Edit history

Date	Action	Author
2015-06-17	Authored	Jupe S
2015-06-17	Created	Jupe S
2016-09-05	Reviewed	Meldal BH
2016-11-14	Edited	Jupe S
2022-03-30	Modified	Weiser JD

7 submitted entities found in this pathway, mapping to 13 Reactome entities

Input	UniProt Id
ENSG00000108688	P13500
ENSG00000169245	P02778
ENSG00000277632	P10147, P16619

Input	UniProt Id
ENSG00000113302	P29460
ENSG00000171049	P21462

Input	UniProt Id
ENSG00000163739	P09341
ENSG00000171051	P21462

Input	Ensembl Id
ENSG00000113302	ENSG00000113302
ENSG00000171051	ENSG00000171051

Input	Ensembl Id
ENSG00000163739	ENSG00000163739
ENSG00000277632	ENSG00000277632

Input	Ensembl Id
ENSG00000169245	ENSG00000169245

8. Peptide ligand-binding receptors (R-HSA-375276)

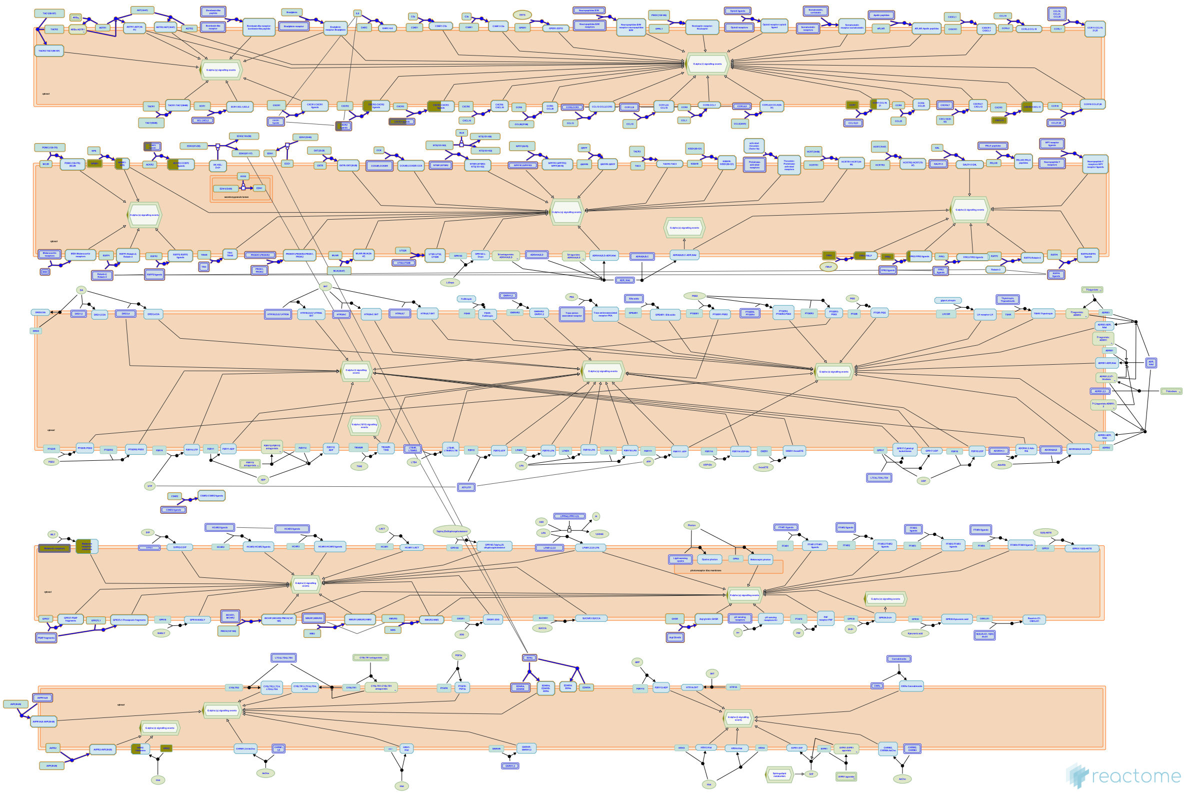

These receptors, a subset of the Class A/1 (Rhodopsin-like) family, all bind peptide ligands which include the chemokines, opioids and somatostatins.

References

Møller LN, Hartmann B, Stidsen CE & Holst JJ (2003). Somatostatin receptors. Biochim Biophys Acta, 1616, 1-84.

Gabrilovac J, Balog T & Martin-Kleiner I (2006). Signal transduction induced by opioids in immune cells: a review. Neuroimmunomodulation, 13, 1-7.

Satake H & Kawada T (2006). Overview of the primary structure, tissue-distribution, and functions of tachykinins and their receptors. Curr Drug Targets, 7, 963-74.

van der Westhuizen ET, Halls ML, Summers RJ & Bathgate RA (2007). Relaxin family peptide receptors--former orphans reunite with their parent ligands to activate multiple signalling pathways. Br J Pharmacol, 150, 677-91.

Evans JF (2002). Cysteinyl leukotriene receptors. Prostaglandins Other Lipid Mediat, 68, 587-97.

Edit history

Date	Action	Author
2008-08-21	Authored	Jassal B
2008-08-21	Created	Jassal B
2008-09-01	Edited	D'Eustachio P
2008-09-01	Reviewed	Bockaert J
2016-11-17	Revised	D'Eustachio P

Date	Action	Author
2022-03-23	Modified	Weiser JD

11 submitted entities found in this pathway, mapping to 15 Reactome entities

Input	UniProt Id	Input	UniProt Id	Input	UniProt Id
ENSG00000108688	P13500, P80098	ENSG00000126353	P32248	ENSG00000156234	O43927, P02775
ENSG00000163735	P42830	ENSG00000163739	P09341	ENSG00000169245	P02778
ENSG00000169248	O14625	ENSG00000171049	P21462, P25090	ENSG00000171051	P21462
ENSG00000187258	Q6W5P4	ENSG00000277632	P10147, P16619		

9. Innate Immune System (R-HSA-168249)

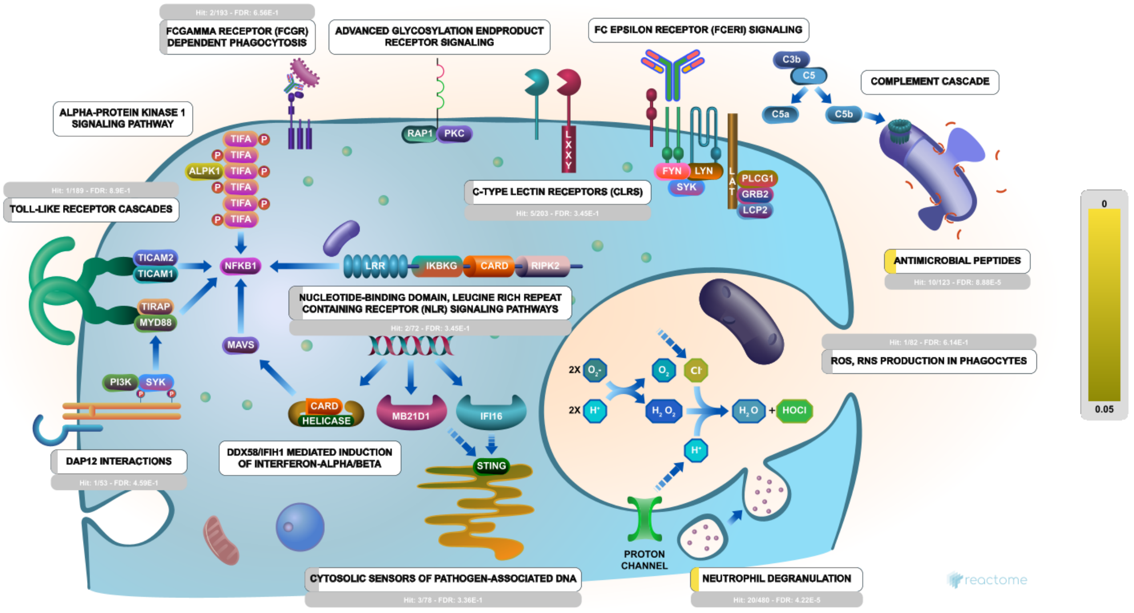

Innate immunity encompases the nonspecific part of immunity tha are part of an individual's natural biologic makeup

References

Edit history

Date	Action	Author
2005-11-11	Created	Gillespie ME
2022-03-23	Modified	Weiser JD

36 submitted entities found in this pathway, mapping to 39 Reactome entities

Input	UniProt Id	Input	UniProt Id	Input	UniProt Id
ENSG00000007171	P35228	ENSG00000057149	P29508	ENSG00000100453	P08311
ENSG00000103313	O15553	ENSG00000112299	O95497	ENSG00000113356	O15318
ENSG00000124233	P04279	ENSG00000124256	Q9H171	ENSG00000124731	Q9NP99
ENSG00000134827	P20061	ENSG00000139572	Q9NQS5	ENSG00000143546	P05109
ENSG00000143556	P31151, Q86SG5	ENSG00000145113	Q99102	ENSG00000145287	Q9NZF1
ENSG00000150337	P12314	ENSG00000156234	P02775	ENSG00000163568	O14862
ENSG00000163739	P09341	ENSG00000164047	P49913	ENSG00000166523	Q9ULY5
ENSG00000166527	Q8WXI8	ENSG00000171049	P21462, P25090	ENSG00000171051	P21462
ENSG00000176797	P81534	ENSG00000177243	P81534	ENSG00000177257	O15263
ENSG00000186191	P59827	ENSG00000186431	P24071	ENSG00000188404	P14151
ENSG00000197249	P01009	ENSG00000198805	P00491	ENSG00000203747	P08637
ENSG00000205420	P04264	ENSG00000205846	Q6EIG7, Q8WTT0	ENSG00000214643	Q30KQ1

10. Neutrophil degranulation (R-HSA-6798695)

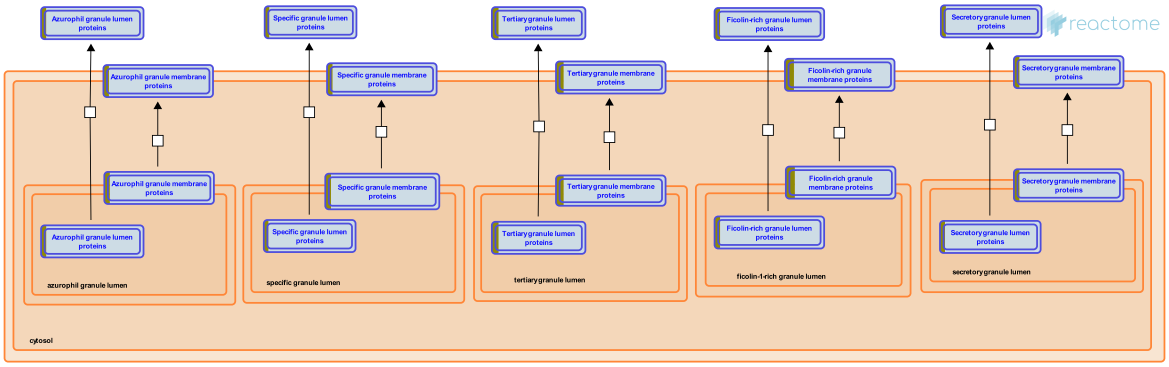

Neutrophils are the most abundant leukocytes (white blood cells), indispensable in defending the body against invading microorganisms. In response to infection, neutrophils leave the circulation and migrate towards the inflammatory focus. They contain several subsets of granules that are mobilized to fuse with the cell membrane or phagosomal membrane, resulting in the exocytosis or exposure of membrane proteins. Traditionally, neutrophil granule constituents are described as antimicrobial or proteolytic, but granules also introduce membrane proteins to the cell surface, changing how the neutrophil responds to its environment (Borregaard et al. 2007). Primed neutrophils actively secrete cytokines and other inflammatory mediators and can present antigens via MHC II, stimulating T-cells (Wright et al. 2010).

Granules form during neutrophil differentiation. Granule subtypes can be distinguished by their content but overlap in structure and composition. The differences are believed to be a consequence of changing protein expression and differential timing of granule formation during the terminal processes of neutrophil differentiation, rather than sorting (Le Cabec et al. 1996).

The classical granule subsets are Azurophil or primary granules (AG), secondary granules (SG) and gelatinase granules (GG). Neutrophils also contain exocytosable storage cell organelles, storage vesicles (SV), formed by endocytosis they contain many cell-surface markers and extracellular, plasma proteins (Borregaard et al. 1992). Ficolin-1-rich granules (FG) are like GGs highly exocytosable but gelatinase-poor (Rorvig et al. 2009).

References

Heegaard NH, Rørvig S, Borregaard N & Østergaard O (2013). Proteome profiling of human neutrophil granule subsets, secretory vesicles, and cell membrane: correlation with transcriptome profiling of neutrophil precursors. J. Leukoc. Biol., 94, 711-21.

Sørensen OE, Borregaard N & Theilgaard-Mönch K (2007). Neutrophil granules: a library of innate immunity proteins. Trends Immunol., 28, 340-5.

Nielsen MH, Johnsen AH, Bjerrum OW, Borregaard N, Kjeldsen L, Rygaard K, ... Bastholm L (1992). Stimulus-dependent secretion of plasma proteins from human neutrophils. J. Clin. Invest., 90, 86-96.

Bucknall RC, Wright HL, Edwards SW & Moots RJ (2010). Neutrophil function in inflammation and inflammatory diseases. Rheumatology (Oxford), 49, 1618-31.

Le Cabec V, Borregaard N, Calafat J & Cowland JB (1996). Targeting of proteins to granule subsets is determined by timing and not by sorting: The specific granule protein NGAL is localized to azurophil granules when expressed in HL-60 cells. Proc. Natl. Acad. Sci. U.S.A., 93, 6454-7.

Edit history

Date	Action	Author
2015-09-21	Authored	Jupe S
2015-09-21	Created	Jupe S
2016-06-13	Edited	Jupe S
2016-06-13	Reviewed	Heegaard N
2022-03-23	Modified	Weiser JD

20 submitted entities found in this pathway, mapping to 21 Reactome entities

Input	UniProt Id	Input	UniProt Id	Input	UniProt Id
ENSG00000057149	P29508	ENSG00000100453	P08311	ENSG00000112299	O95497
ENSG00000134827	P20061	ENSG00000139572	Q9NQS5	ENSG00000143546	P05109
ENSG00000143556	P31151	ENSG00000145287	Q9NZF1	ENSG00000156234	P02775
ENSG00000163739	P09341	ENSG00000164047	P49913	ENSG00000166527	Q8WXI8
ENSG00000171049	P21462, P25090	ENSG00000171051	P21462	ENSG00000186431	P24071
ENSG00000188404	P14151	ENSG00000197249	P01009	ENSG00000198805	P00491
ENSG00000205420	P04264	ENSG00000205846	Q8WTT0		

11. Antimicrobial peptides (R-HSA-6803157)

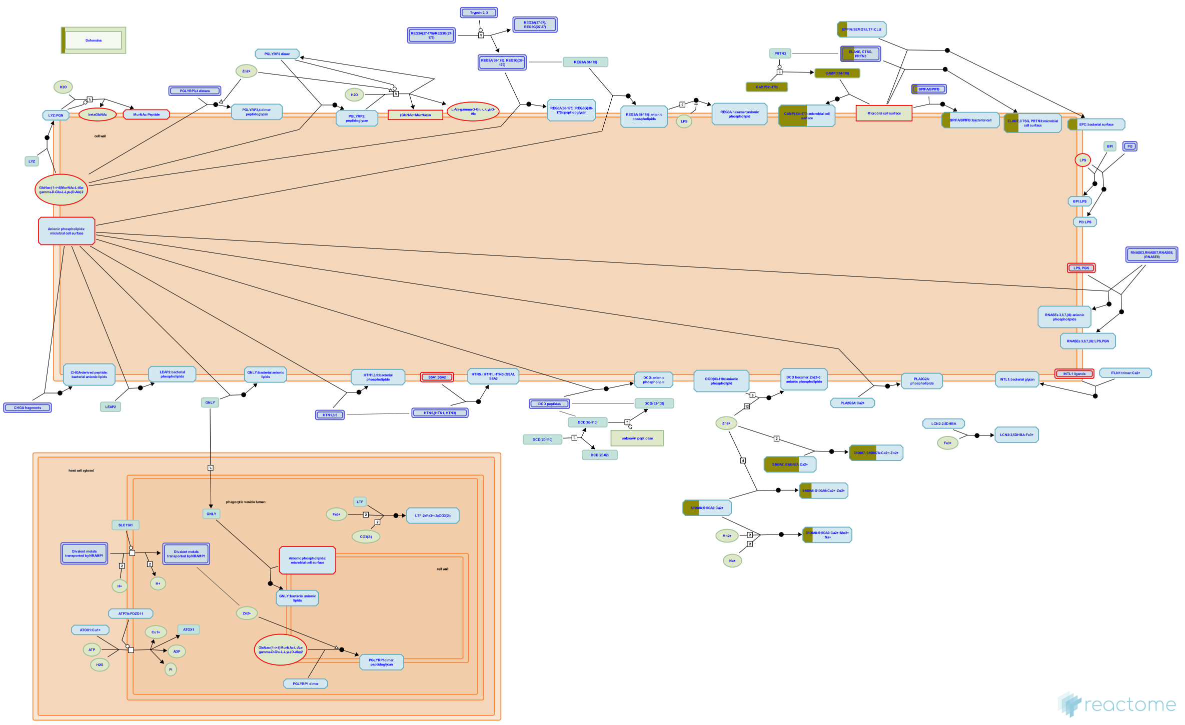

Antimicrobial peptides (AMPs) are small molecular weight proteins with broad spectrum of antimicrobial activity against bacteria, viruses, and fungi (Zasloff M 2002; Radek K & Gallo R 2007). The majority of known AMPs are cationic peptides with common structural characteristics where domains of hydrophobic and cationic amino acids are spatially arranged into an amphipathic design, which facilitates their interaction with bacterial membranes (Shai Y 2002; Yeaman MR & Yount NY 2003; Brown KL & Hancock RE 2006; Dennison SR et al. 2005; Zelezetsky I & Tossi A 2006). It is generally excepted that the electrostatic interaction facilitates the initial binding of the positively charged peptides to the negatively charged bacterial membrane. Moreover, the structural amphiphilicity of AMPs is thought to promote their integration into lipid bilayers of pathogenic cells, leading to membrane disintegration and finally to the microbial cell death. In addition to cationic AMPs a few anionic antimicrobial peptides have been found in humans, however their mechanism of action remains to be clarified (Lai Y et al. 2007; Harris F et al. 2009; Paulmann M et al. 2012). Besides the direct neutralizing effects on bacteria AMPs may modulate cells of the adaptive immunity (neutrophils, T-cells, macrophages) to control inflammation and/or to increase bacterial clearance.

AMPs have also been referred to as cationic host defense peptides, anionic antimicrobial peptides/proteins, cationic amphipathic peptides, cationic AMPs, host defense peptides and alpha-helical antimicrobial peptides (Brown KL & Hancock RE 2006; Harris F et al. 2009; Groenink J et al. 1999; Bradshaw J 2003; Riedl S et al. 2011; Huang Y et al. 2010).

The Reactome module describes the interaction events of various types of human AMPs, such as cathelicidin, histatins and neutrophil serine proteases, with conserved patterns of microbial membranes at the host-pathogen interface. The module includes also proteolytic processing events for dermcidin (DCD) and cathelicidin (CAMP) that become functional upon cleavage. In addition, the module highlights an AMP-associated ability of the host to control metal quota at inflammation sites to influence host-pathogen interactions.

References

Yeaman MR & Yount NY (2003). Mechanisms of antimicrobial peptide action and resistance. Pharmacol. Rev., 55, 27-55.

Ren D & Bahar AA (2013). Antimicrobial peptides. Pharmaceuticals (Basel), 6, 1543-75.

Edit history

Date	Action	Author
2015-10-05	Authored	Shamovsky V
2015-10-05	Created	Shamovsky V
2016-04-15	Reviewed	Jupe S
2016-08-02	Reviewed	Hains DS
2016-08-15	Edited	Shamovsky V
2022-03-23	Modified	Weiser JD

10 submitted entities found in this pathway, mapping to 11 Reactome entities

Input	UniProt Id	Input	UniProt Id	Input	UniProt Id
ENSG00000100453	P08311	ENSG00000124233	P04279	ENSG00000143546	P05109
ENSG00000143556	P31151, Q86SG5	ENSG00000164047	P49913	ENSG00000176797	P81534
ENSG00000177243	P81534	ENSG00000177257	O15263	ENSG00000186191	P59827
ENSG00000214643	Q30KQ1				

12. Interferon Signaling (R-HSA-913531)

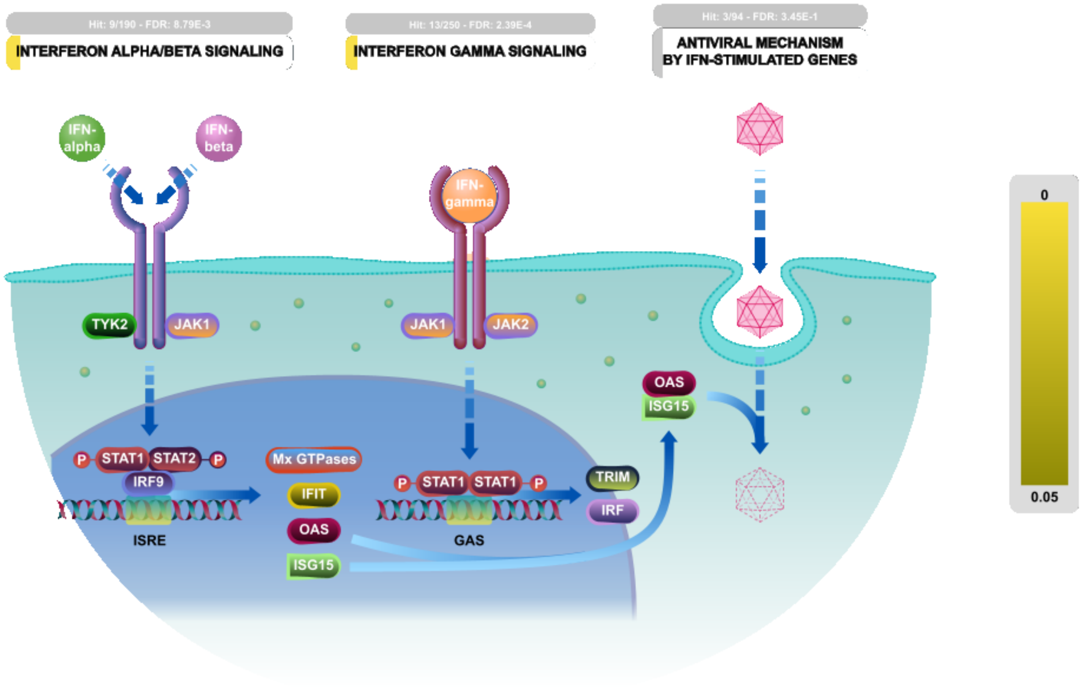

Interferons (IFNs) are cytokines that play a central role in initiating immune responses, especially antiviral and antitumor effects. There are three types of IFNs:Type I (IFN-alpha, -beta and others, such as omega, epsilon, and kappa), Type II (IFN-gamma) and Type III (IFN-lamda). In this module we are mainly focusing on type I IFNs alpha and beta and type II IFN-gamma. Both type I and type II IFNs exert their actions through cognate receptor complexes, IFNAR and IFNGR respectively, present on cell surface membranes. Type I IFNs are broadly expressed heterodimeric receptors composed of the IFNAR1 and IFNAR2 subunits, while the type II IFN receptor consists of IFNGR1 and IFNGR2. Type III interferon lambda has three members: lamda1 (IL-29), lambda2 (IL-28A), and lambda3 (IL-28B) respectively. IFN-lambda signaling is initiated through unique heterodimeric receptor composed of IFN-LR1/IF-28Ralpha and IL10R2 chains.

Type I IFNs typically recruit JAK1 and TYK2 proteins to transduce their signals to STAT1 and 2; in combination with IRF9 (IFN-regulatory factor 9), these proteins form the heterotrimeric complex ISGF3. In nucleus ISGF3 binds to IFN-stimulated response elements (ISRE) to promote gene induction.

Type II IFNs in turn rely upon the activation of JAKs 1 and 2 and STAT1. Once activated, STAT1 dimerizes to form the transcriptional regulator GAF (IFNG activated factor) and this binds to the IFNG activated sequence (GAS) elements and initiate the transcription of IFNG-responsive genes.

Like type I IFNs, IFN-lambda recruits TYK2 and JAK1 kinases and then promote the phosphorylation of STAT1/2, and induce the ISRE3 complex formation.

References

Schroder K, Ravasi T, Hume DA & Hertzog PJ (2004). Interferon-gamma: an overview of signals, mechanisms and functions. J Leukoc Biol, 75, 163-89.

Platanias LC (2005). Mechanisms of type-I- and type-II-interferon-mediated signalling. Nat Rev Immunol, 5, 375-86.

Gough DJ, Levy DE, Clarke CJ & Johnstone RW (2008). IFNgamma signaling-does it mean JAK-STAT?. Cytokine Growth Factor Rev, 19, 383-94.

Ferreira PC, Bonjardim CA & Kroon EG (2009). Interferons: signaling, antiviral and viral evasion. Immunol Lett, 122, 1-11.

Platanias LC & Uddin S (2004). Mechanisms of type-I interferon signal transduction. J Biochem Mol Biol, 37, 635-41.

Edit history

Date	Action	Author
2010-07-07	Edited	Garapati P V
2010-07-07	Authored	Garapati P V
2010-07-16	Created	Garapati P V
2010-08-17	Reviewed	Abdul-Sater AA, Schindler C
2022-03-23	Modified	Weiser JD

8 submitted entities found in this pathway, mapping to 18 Reactome entities

Input	UniProt Id	Input	UniProt Id	Input	UniProt Id
ENSG00000111331	Q9Y6K5	ENSG00000111335	P29728	ENSG00000115415	P42224, P42224-1, P42224-2
ENSG00000126709	P09912	ENSG00000150244	Q8IWZ4	ENSG00000150337	P12314, Q92637
ENSG00000183347	Q6ZN66	ENSG00000198019	Q92637		

Input	Ensembl Id	Input	Ensembl Id	Input	Ensembl Id
ENSG00000111331	ENSG00000111331	ENSG00000111335	ENSG00000111335	ENSG00000126709	ENSG00000126709
ENSG00000150244	ENSG00000150244	ENSG00000150337	ENSG00000150337	ENSG00000183347	ENSG00000183347
ENSG00000198019	ENSG00000198019				

13. Class A/1 (Rhodopsin-like receptors) (R-HSA-373076)

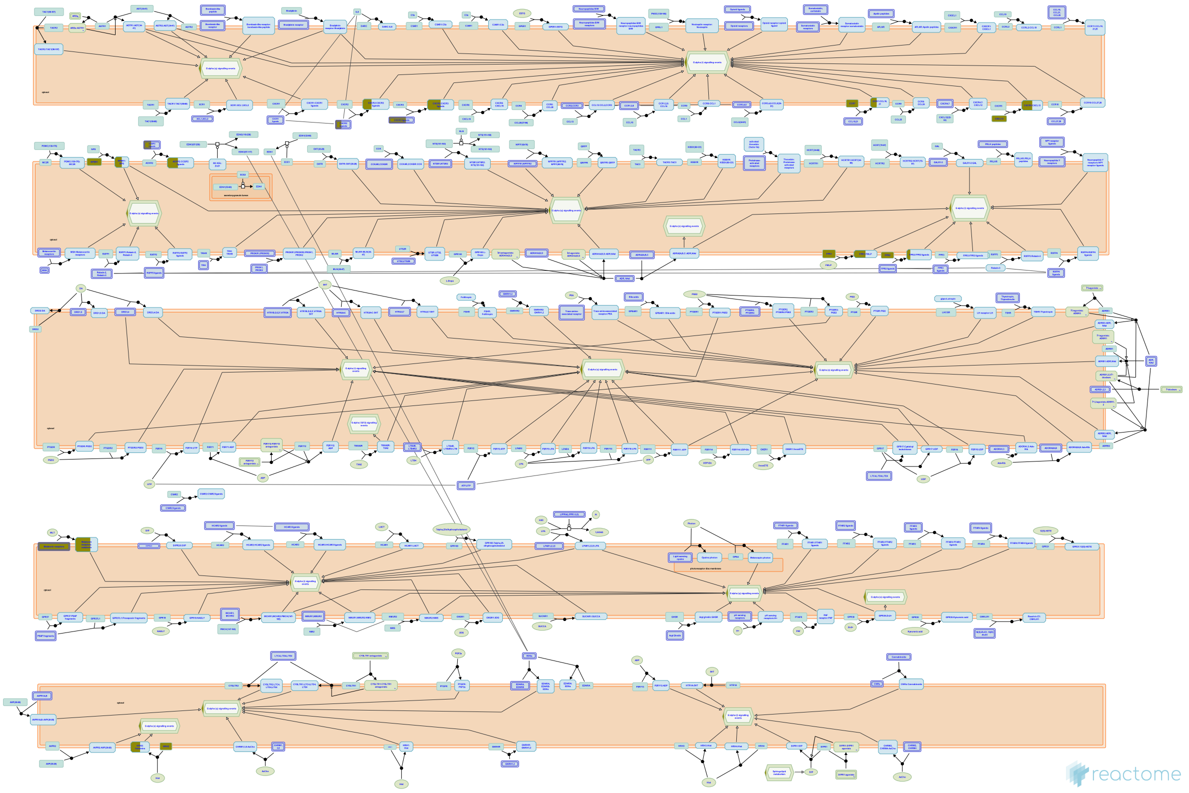

Rhodopsin-like receptors (class A/1) are the largest group of GPCRs and are the best studied group from a functional and structural point of view. They show great diversity at the sequence level and thus, can be subdivided into 19 subfamilies (Subfamily A1-19) based on a phylogenetic analysis (Joost P and Methner A, 2002). They represent members which include hormone, light and neurotransmitter receptors and encompass a wide range of functions including many autocrine, paracrine and endocrine processes.

References

Bouhelal R, Jacoby E, Gerspacher M & Seuwen K (2006). The 7 TM G-protein-coupled receptor target family. ChemMedChem, 1, 761-82.

Edit history

Date	Action	Author
2008-07-03	Authored	Jassal B
2008-07-14	Created	Jassal B
2008-09-01	Edited	D'Eustachio P
2008-09-01	Reviewed	Bockaert J
2016-11-17	Revised	D'Eustachio P
2022-03-23	Modified	Weiser JD

14 submitted entities found in this pathway, mapping to 18 Reactome entities

Input	UniProt Id	Input	UniProt Id	Input	UniProt Id
ENSG00000108688	P13500, P80098	ENSG00000113749	P25021	ENSG00000126353	P32248

Input	UniProt Id	Input	UniProt Id	Input	UniProt Id
ENSG00000134640	P49286	ENSG00000156234	O43927, P02775	ENSG00000163735	P42830
ENSG00000163739	P09341	ENSG00000168412	P48039	ENSG00000169245	P02778
ENSG00000169248	O14625	ENSG00000171049	P21462, P25090	ENSG00000171051	P21462
ENSG00000187258	Q6W5P4	ENSG00000277632	P10147, P16619		

14. Interleukin-4 and Interleukin-13 signaling (R-HSA-6785807)

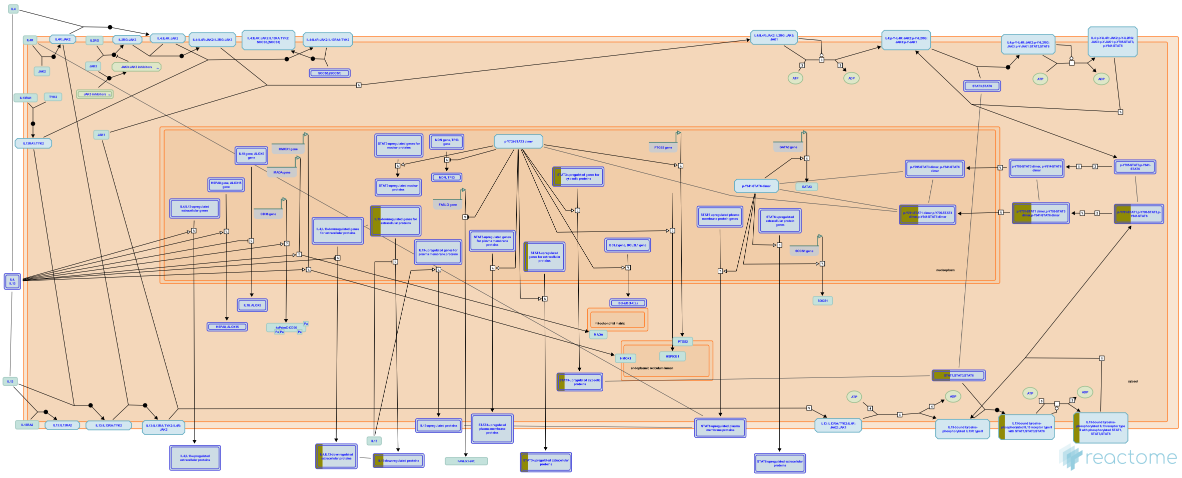

Interleukin-4 (IL4) is a principal regulatory cytokine during the immune response, crucially important in allergy and asthma (Nelms et al. 1999). When resting T cells are antigen-activated and expand in response to Interleukin-2 (IL2), they can differentiate as Type 1 (Th1) or Type 2 (Th2) T helper cells. The outcome is influenced by IL4. Th2 cells secrete IL4, which both stimulates Th2 in an autocrine fashion and acts as a potent B cell growth factor to promote humoral immunity (Nelms et al. 1999).

Interleukin-13 (IL13) is an immunoregulatory cytokine secreted predominantly by activated Th2 cells. It is a key mediator in the pathogenesis of allergic inflammation. IL13 shares many functional properties with IL4, stemming from the fact that they share a common receptor subunit. IL13 receptors are expressed on human B cells, basophils, eosinophils, mast cells, endothelial cells, fibroblasts, monocytes, macrophages, respiratory epithelial cells, and smooth muscle cells, but unlike IL4, not T cells. Thus IL13 does not appear to be important in the initial differentiation of CD4 T cells into Th2 cells, rather it is important in the effector phase of allergic inflammation (Hershey et al. 2003).

IL4 and IL13 induce “alternative activation” of macrophages, inducing an anti-inflammatory phenotype by signaling through IL4R alpha in a STAT6 dependent manner. This signaling plays an important role in the Th2 response, mediating anti-parasitic effects and aiding wound healing (Gordon & Martinez 2010, Loke et al. 2002)

There are two types of IL4 receptor complex (Andrews et al. 2006). Type I IL4R (IL4R1) is predominantly expressed on the surface of hematopoietic cells and consists of IL4R and IL2RG, the common gamma chain. Type II IL4R (IL4R2) is predominantly expressed on the surface of nonhematopoietic cells, it consists of IL4R and IL13RA1 and is also the type II receptor for IL13. (Obiri et al. 1995, Aman et al. 1996, Hilton et al. 1996, Miloux et al. 1997, Zhang et al. 1997). The second receptor for IL13 consists of IL4R and Interleukin-13 receptor alpha 2 (IL13RA2), sometimes called Interleukin-13 binding protein (IL13BP). It has a high affinity receptor for IL13 (Kd = 250 pmol/L) but is not sufficient to render cells responsive to IL13, even in the presence of IL4R (Donaldson et al. 1998). It is reported to exist in soluble form (Zhang et al. 1997) and when overexpressed reduces JAK-STAT signaling (Kawakami et al. 2001). It's function may be to prevent IL13 signalling via the functional IL4R:IL13RA1 receptor. IL13RA2 is overexpressed and enhances cell invasion in some human cancers (Joshi & Puri 2012).

The first step in the formation of IL4R1 (IL4:IL4R:IL2RB) is the binding of IL4 with IL4R (Hoffman et al. 1995, Shen et al. 1996, Hage et al. 1999). This is also the first step in formation of IL4R2 (IL4:IL4R:IL13RA1). After the initial binding of IL4 and IL4R, IL2RB binds (LaPorte et al. 2008), to form IL4R1. Alternatively, IL13RA1 binds, forming IL4R2. In contrast, the type II IL13 complex (IL13R2) forms with IL13 first binding to IL13RA1 followed by recruitment of IL4R (Wang et al. 2009).

Crystal structures of the IL4:IL4R:IL2RG, IL4:IL4R:IL13RA1 and IL13:IL4R:IL13RA1 complexes have been determined (LaPorte et al. 2008). Consistent with these structures, in monocytes IL4R is tyrosine phosphorylated in response to both IL4 and IL13 (Roy et al. 2002, Gordon & Martinez 2010) while IL13RA1 phosphorylation is induced only by IL13 (Roy et al. 2002, LaPorte et al. 2008) and IL2RG phosphorylation is induced only by IL4 (Roy et al. 2002).

Both IL4 receptor complexes signal through Jak/STAT cascades. IL4R is constitutively-associated with JAK2 (Roy et al. 2002) and associates with JAK1 following binding of IL4 (Yin et al. 1994) or IL13 (Roy et al. 2002). IL2RG constitutively associates with JAK3 (Boussiotis et al. 1994, Russell et al. 1994). IL13RA1 constitutively associates with TYK2 (Umeshita-Suyama et al. 2000, Roy et al. 2002, LaPorte et al. 2008, Bhattacharjee et al. 2013).

IL4 binding to IL4R1 leads to phosphorylation of JAK1 (but not JAK2) and STAT6 activation (Takeda et al. 1994, Ratthe et al. 2007, Bhattacharjee et al. 2013).

IL13 binding increases activating tyrosine-99 phosphorylation of IL13RA1 but not that of IL2RG. IL4 binding to IL2RG leads to its tyrosine phosphorylation (Roy et al. 2002). IL13 binding to IL4R2 leads to TYK2 and JAK2 (but not JAK1) phosphorylation (Roy & Cathcart 1998, Roy et al. 2002).

Phosphorylated TYK2 binds and phosphorylates STAT6 and possibly STAT1 (Bhattacharjee et al. 2013).

A second mechanism of signal transduction activated by IL4 and IL13 leads to the insulin receptor substrate (IRS) family (Kelly-Welch et al. 2003). IL4R1 associates with insulin receptor substrate 2 and activates the PI3K/Akt and Ras/MEK/Erk pathways involved in cell proliferation, survival and translational control. IL4R2 does not associate with insulin receptor substrate 2 and consequently the PI3K/Akt and Ras/MEK/Erk pathways are not activated (Busch-Dienstfertig & González-Rodríguez 2013).

References

Ryan JJ, Nelms K, Paul WE, Zamorano J & Keegan AD (1999). The IL-4 receptor: signaling mechanisms and biologic functions. Annu. Rev. Immunol., 17, 701-38.

Hershey GK (2003). IL-13 receptors and signaling pathways: an evolving web. J. Allergy Clin. Immunol., 111, 677-90; quiz 691.

Edit history

Date	Action	Author
2015-07-01	Authored	Jupe S
2015-07-01	Created	Jupe S
2016-09-02	Edited	Jupe S
2016-09-02	Reviewed	Leibovich SJ

Date	Action	Author
2022-03-30	Modified	Weiser JD

7 submitted entities found in this pathway, mapping to 12 Reactome entities

Input	UniProt Id	Input	UniProt Id	Input	UniProt Id
ENSG00000007171	P35228	ENSG00000108688	P13500	ENSG00000110944	Q9NPF7
ENSG00000112115	Q16552	ENSG00000112116	Q96PD4	ENSG00000113302	P29460
ENSG00000115415	P42224				

Input	Ensembl Id	Input	Ensembl Id	Input	Ensembl Id
ENSG00000007171	ENSG00000007171	ENSG00000112115	ENSG00000112115	ENSG00000112116	ENSG00000112116
ENSG00000113302	ENSG00000113302	ENSG00000115415	ENSG00000115415		

15. Interferon gamma signaling (R-HSA-877300)

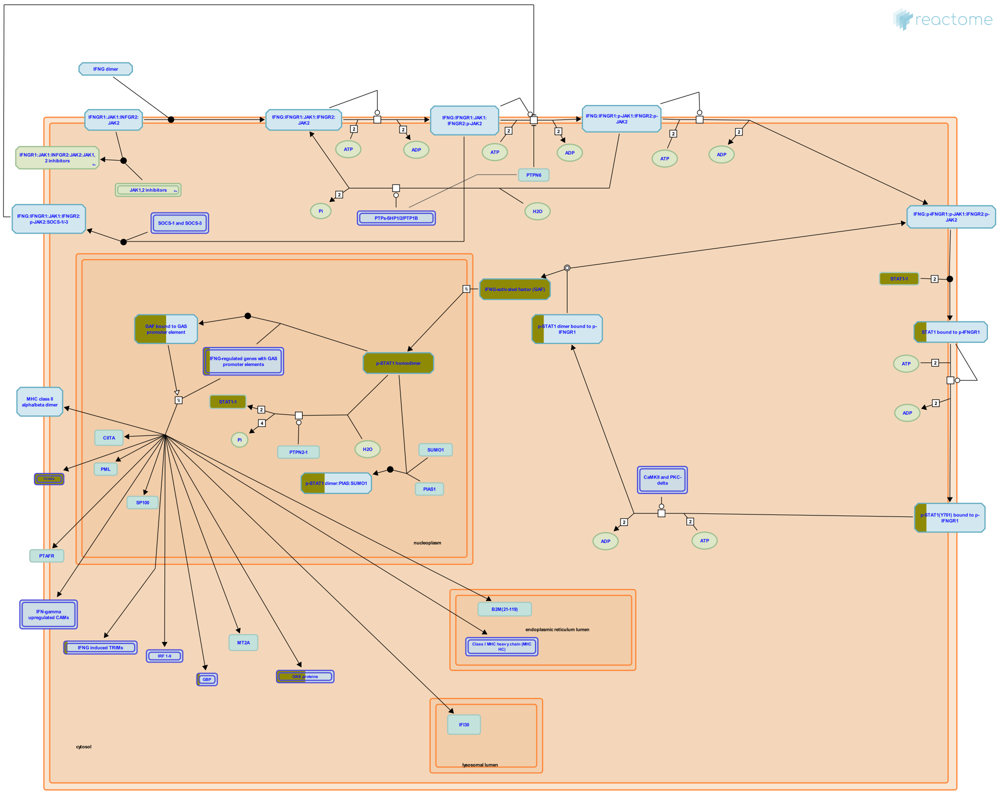

Interferon-gamma (IFN-gamma) belongs to the type II interferon family and is secreted by activated immune cells-primarily T and NK cells, but also B-cells and APC. INFG exerts its effect on cells by interacting with the specific IFN-gamma receptor (IFNGR). IFNGR consists of two chains, namely IFNGR1 (also known as the IFNGR alpha chain) and IFNGR2 (also known as the IFNGR beta chain). IFNGR1 is the ligand binding receptor and is required but not sufficient for signal transduction, whereas IFNGR2 do not bind IFNG independently but mainly plays a role in IFNG signaling and is generally the limiting factor in IFNG responsiveness. Both IFNGR chains lack intrinsic kinase/phosphatase activity and thus rely on other signaling proteins like Janus-activated kinase 1 (JAK1), JAK2 and Signal transducer and activator of transcription 1 (STAT-1) for signal transduction. IFNGR complex in its resting state is a preformed tetramer and upon IFNG association undergoes a conformational change. This conformational change induces the phosphorylation and activation of JAK1, JAK2, and STAT1 which in turn induces genes containing the gamma-interferon activation sequence (GAS) in the promoter.

References

Schroder K, Ravasi T, Hume DA & Hertzog PJ (2004). Interferon-gamma: an overview of signals, mechanisms and functions. J Leukoc Biol, 75, 163-89.

Aguet M, Bach EA & Schreiber RD (1997). The IFN gamma receptor: a paradigm for cytokine receptor signaling. Annu Rev Immunol, 15, 563-91.

Gough DJ, Levy DE, Clarke CJ & Johnstone RW (2008). IFNgamma signaling-does it mean JAK-STAT?. Cytokine Growth Factor Rev, 19, 383-94.

Izotova LS, Garotta G, Muthukumaran G, Kotenko SV, Cook JR & Pestka S (1997). The interferon gamma (IFN-gamma) receptor: a paradigm for the multichain cytokine receptor. Cytokine Growth Factor Rev, 8, 189-206.

Edit history

Date	Action	Author
2010-06-08	Edited	Garapati P V
2010-06-08	Authored	Garapati P V
2010-06-11	Created	Garapati P V
2010-08-17	Reviewed	Abdul-Sater AA, Schindler C
2022-03-30	Modified	Weiser JD

7 submitted entities found in this pathway, mapping to 14 Reactome entities

Input	UniProt Id	Input	UniProt Id	Input	UniProt Id
ENSG00000111331	Q9Y6K5	ENSG00000111335	P29728	ENSG00000115415	P42224-1
ENSG00000150244	Q8IWZ4	ENSG00000150337	P12314, Q92637	ENSG00000183347	Q6ZN66
ENSG00000198019	Q92637				

Input	Ensembl Id	Input	Ensembl Id	Input	Ensembl Id
ENSG00000111331	ENSG00000111331	ENSG00000111335	ENSG00000111335	ENSG00000150244	ENSG00000150244
ENSG00000150337	ENSG00000150337	ENSG00000183347	ENSG00000183347	ENSG00000198019	ENSG00000198019

16. GPCR ligand binding (R-HSA-500792)

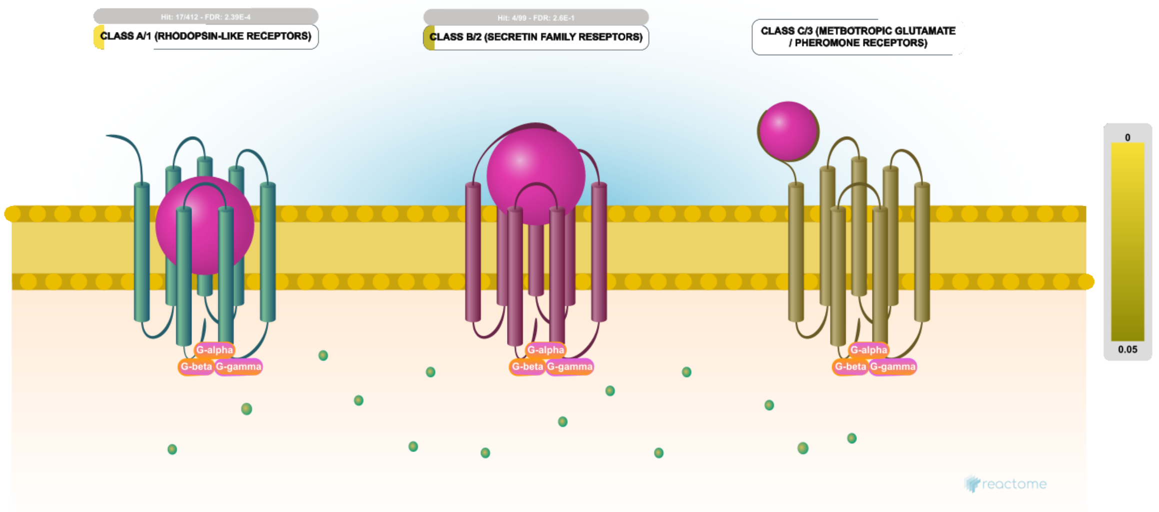

Cellular compartments: plasma membrane.

There are more than 800 G-protein coupled receptor (GPCRs) in the human genome, making it the largest receptor superfamily. GPCRs are also the largest class of drug targets, involved in virtually all physiological processes (Frederiksson 2003). GPCRs are receptors for a diverse range of ligands from large proteins to photons (Kristiansen et al. 2004) and have an equal diversity of ligand-binding mechanisms (Gether et al. 2002). Classical GPCR signaling involves signal transduction via heterotrimeric G-proteins, though G-protein independent mechanisms have been reported.

Rhodopsin-like receptors (class A/1) are by far the largest group of GPCRs and the best studied, though a large proportion of the functional and structural studies have focused on a very few members; many remain functionally uncharacterized. This large family can be subdivided into at least 19 subfamilies (Subfamily A1-19) based on phylogenetic analysis (Joost & Methner 2002). Family A includes receptors for a wide variety of ligands including hormones, light and neurotransmitters, encompassing a wide range of functions including many autocrine, paracrine and endocrine processes.

The secretin-like family B/2 GPCRs includes receptors for many hormone-like peptides, such as secretin, calcitonin, parathyroid hormone/parathyroid hormone-related peptides and vasoactive intestinal peptide, which activate adenylyl cyclase and the phosphatidyl-inositol-calcium pathway (Harmar 2001).

The class C/3 GPCRs include the metabotropic glutamate receptors and taste receptors (Brauner-Osborne et al. 2007). All have a large extracellular N-terminus that structurally resembles a clamshell and has an important role in ligand binding.

References

Kristiansen K (2004). Molecular mechanisms of ligand binding, signaling, and regulation within the superfamily of G-protein-coupled receptors: molecular modeling and mutagenesis approaches to receptor structure and function. Pharmacol Ther, 103, 21-80.

Edit history

Date	Action	Author
2009-12-11	Reviewed	D'Eustachio P
2010-02-05	Authored	Jassal B
2010-02-05	Created	Jassal B
2010-02-10	Edited	Jupe S
2022-03-23	Modified	Weiser JD

16 submitted entities found in this pathway, mapping to 22 Reactome entities

Input	UniProt Id	Input	UniProt Id	Input	UniProt Id
ENSG00000108688	P13500, P80098	ENSG00000110680	P01258, P06881, P10092	ENSG00000113749	P25021
ENSG00000114251	P41221	ENSG00000126353	P32248	ENSG00000134640	P49286
ENSG00000156234	O43927, P02775	ENSG00000163735	P42830	ENSG00000163739	P09341
ENSG00000168412	P48039	ENSG00000169245	P02778	ENSG00000169248	O14625
ENSG00000171049	P21462, P25090	ENSG00000171051	P21462	ENSG00000187258	Q6W5P4
ENSG00000277632	P10147, P16619				

17. Interleukin-36 pathway (R-HSA-9014826)

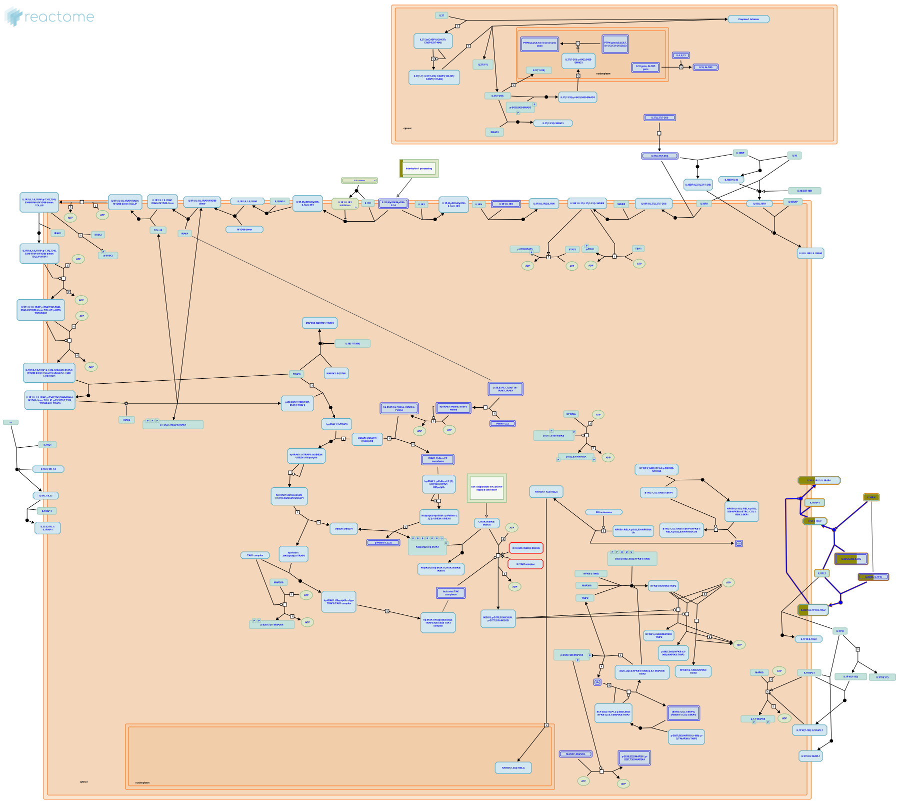

Interleukin-36 alpha (IL36A), IL36B and IL36G are collectively known as IL36. They are members of the Interlukin-1 family that signal through a receptor composed of Interleukin-1 receptor-like 2 (IL1RL2, IL36R) and Interleukin-1 receptor accessory protein (IL1RAP, IL-1R/AcP) to promote inflammatory responses. Interleukin-36 receptor antagonist protein (IL36RN, IL36Ra) is a natural antagonist. IL36 is expressed predominantly by epithelial cells and is implicated strongly through functional and genetic evidence in the pathology of psoriatic disorders.

References

Towne JE & Gabay C (2015). Regulation and function of interleukin-36 cytokines in homeostasis and pathological conditions. J. Leukoc. Biol., 97, 645-52.

Edit history

Date	Action	Author
2014-06-04	Authored	Jupe S
2016-01-28	Edited	Jupe S
2016-01-28	Reviewed	Meldal BH
2017-08-04	Created	Duenas C
2022-03-22	Modified	Weiser JD

3 submitted entities found in this pathway, mapping to 3 Reactome entities

Input	UniProt Id	Input	UniProt Id	Input	UniProt Id
ENSG00000136688	Q9NZH8	ENSG00000136694	Q9UHA7	ENSG00000136695	Q9UBH0

18. Signaling by GPCR (R-HSA-372790)

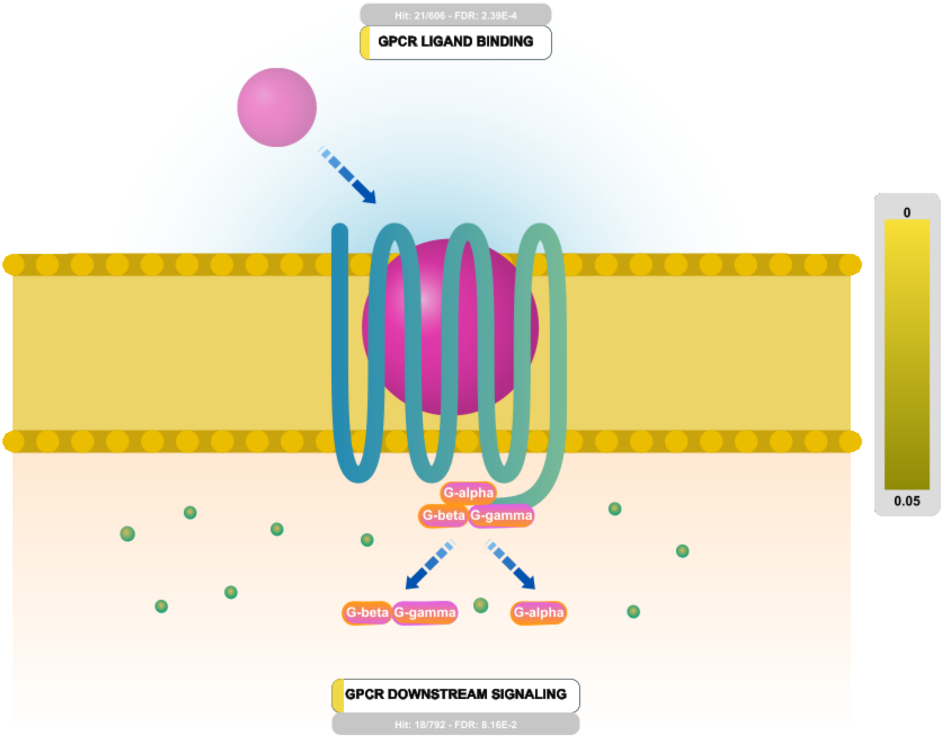

G protein-coupled receptors (GPCRs; 7TM receptors; seven transmembrane domain receptors; heptahelical receptors; G protein-linked receptors [GPLR]) are the largest family of transmembrane receptors in humans, accounting for more than 1% of the protein-coding capacity of the human genome. All known GPCRs share a common architecture of seven membrane-spanning helices connected by intra- and extracellular loops. The extracellular loops contain two highly-conserved cysteine residues that form disulphide bonds to stabilize the structure of the receptor. They recognize diverse messengers such as light, odorants, small molecules, hormones and neurotransmitters. Most GPCRs act as guanine nucleotide exchange factors; activated by ligand binding, they promote GDP-GTP exchange on associated heterotrimeric guanine nucleotide-binding (G) proteins. There are two models for GPCR-G Protein interactions: 1) ligand-GPCR binding first, then binding to G Proteins; 2) "Pre-coupling" of GPCRs and G Proteins before ligand binding (review Oldham WM and Hamm HE, 2008). These in turn activate effector enzymes or ion channels. GPCRs are involved in a range of physiological roles which include the visual sense, smell, behavioural regulation, functions of the autonomic nervous system and regulation of the immune system and inflammation.

GPCRs are divided into classes based on sequence homology and functional similarity. The main mammalian classes, in order of size, are the Rhodopsin-like family A, the Secretin receptor family B, and the Metabotropic glutamate/pheromone receptor family C.

References

Bockaert J & Pin JP (1999). Molecular tinkering of G protein-coupled receptors: an evolutionary success. EMBO J, 18, 1723-9.

Oldham WM & Hamm HE (2008). Heterotrimeric G protein activation by G-protein-coupled receptors. Nat Rev Mol Cell Biol, 9, 60-71.

Bouhelal R, Jacoby E, Gerspacher M & Seuwen K (2006). The 7 TM G-protein-coupled receptor target family. ChemMedChem, 1, 761-82.

Edit history

Date	Action	Author
2008-07-02	Authored	Jassal B
2008-07-02	Created	Jassal B
2008-09-01	Edited	D'Eustachio P
2008-09-01	Reviewed	Bockaert J
2022-03-23	Modified	Weiser JD

18 submitted entities found in this pathway, mapping to 24 Reactome entities

Input	UniProt Id	Input	UniProt Id	Input	UniProt Id
ENSG00000090104	Q08116	ENSG00000108688	P13500, P80098	ENSG00000110680	P01258, P06881, P10092
ENSG00000113749	P25021	ENSG00000114251	P41221	ENSG00000126353	P32248
ENSG00000134640	P49286	ENSG00000139572	Q9NQS5	ENSG00000156234	O43927, P02775
ENSG00000163735	P42830	ENSG00000163739	P09341	ENSG00000168412	P48039
ENSG00000169245	P02778	ENSG00000169248	O14625	ENSG00000171049	P21462, P25090
ENSG00000171051	P21462	ENSG00000187258	Q6W5P4	ENSG00000277632	P10147, P16619

19. Calcitonin-like ligand receptors (R-HSA-419812)

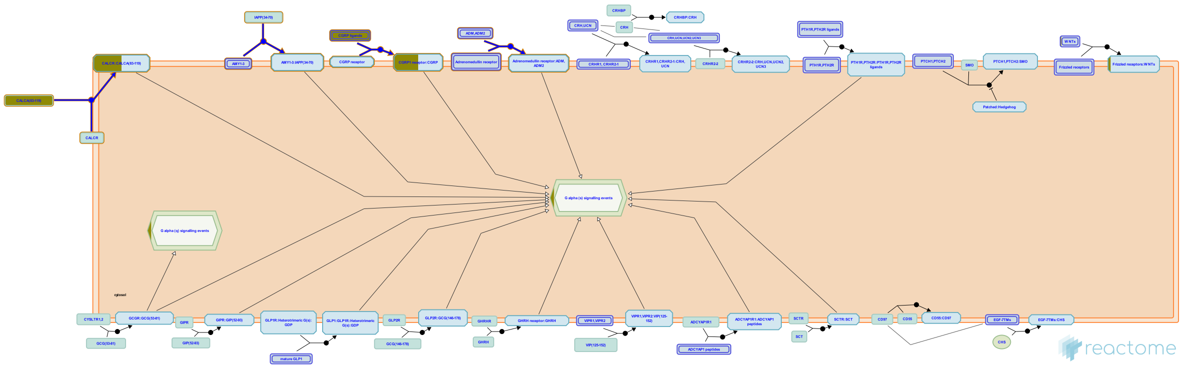

The calcitonin peptide family comprises calcitonin, amylin, calcitonin gene-related peptide (CGRP), adrenomedullin (AM) and intermedin (AM2). Calcitonin is a 32 amino acid peptide, involved in bone homeostasis (Sexton PM et al, 1999). Amylin is a product of the islet beta-cell (Cooper GJ et al, 1987), along with insulin and probably has a hormonal role in the regulation of nutrient intake (Young A and Denaro M, 1998). Adrenomedullin (AM) is a ubiquitously expressed peptide initially isolated from phaechromocytoma (a tumour of the adrenal medulla) (Kitamura K et al, 1993). Both AM and AM2 (Takei Y et al, 2004) belong to a family of calcitonin-related peptide hormones important for regulating diverse physiologic functions and the chemical composition of fluids and tissues.

The receptor family for these peptides consists of two class B GPCRs, the calcitonin receptor (CT) and calcitonin receptor-like receptor (CL) (Poyner DR er al, 2002). Whilst the receptor for calcitonin is a conventional class B GPCR, the receptors for CGRP, AM and amylin require additional proteins, called the receptor activity modifying proteins (RAMPs). There are three RAMPs in mammals; they interact with the CT receptor to convert it to receptors for amylin. For CGRP and AM, the related CL interacts with RAMP1 to give a CGRP receptor and RAMP2 or 3 to give AM receptors. CL by itself will bind no known endogenous ligand.

References

Findlay DM, Martin TJ & Sexton PM (1999). Calcitonin. Curr Med Chem, 6, 1067-93.

Denaro M & Young A (1998). Roles of amylin in diabetes and in regulation of nutrient load. Nutrition, 14, 524-7.

Eto T, Kitamura K, Kangawa K, Kawamoto M, Matsuo H, Ichiki Y & Nakamura S (1993). Adrenomedullin: a novel hypotensive peptide isolated from human pheochromocytoma. Biochem Biophys Res Commun, 192, 553-60.

Miyano S, Ogoshi M, Bannai H, Kawahara T, Inoue K & Takei Y (2004). Identification of novel adrenomedullin in mammals: a potent cardiovascular and renal regulator. FEBS Lett, 556, 53-8.

Fischer JA, Smith DM, Poyner DR, Foord SM, Quirion R, Muff R, ... Sexton PM (2002). International Union of Pharmacology. XXXII. The mammalian calcitonin gene-related peptides, adrenomedullin, amylin, and calcitonin receptors. Pharmacol Rev, 54, 233-46.

Edit history

Date	Action	Author
2009-05-07	Edited	Jassal B

Date	Action	Author
2009-05-07	Authored	Jassal B
2009-05-07	Created	Jassal B
2009-05-29	Reviewed	D'Eustachio P
2022-03-23	Modified	Weiser JD

1 submitted entities found in this pathway, mapping to 3 Reactome entities

Input	UniProt Id
ENSG00000110680	P01258, P06881, P10092

20. Interleukin-20 family signaling (R-HSA-8854691)

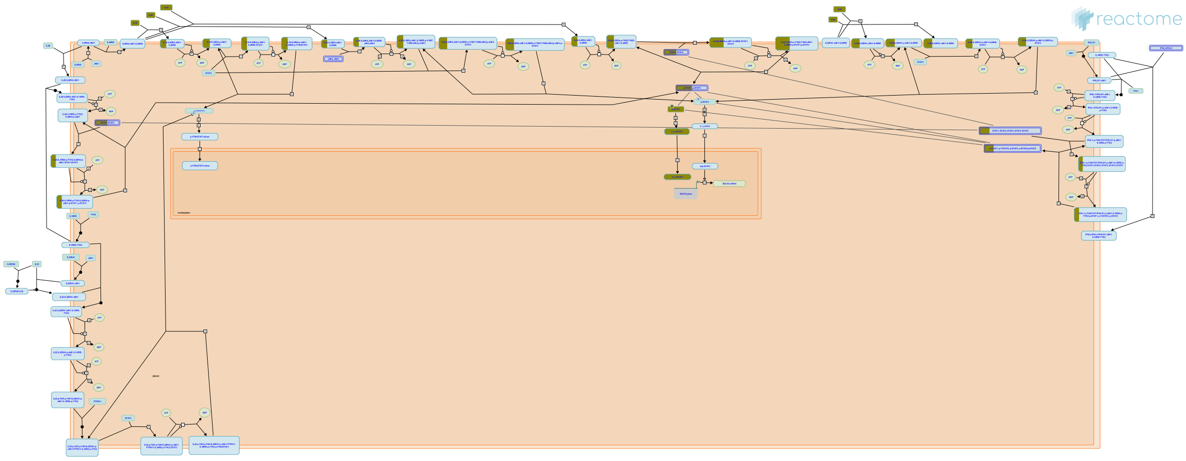

The interleukin 20 (IL20) subfamily comprises IL19, IL20, IL22, IL24 and IL26. They are members of the larger IL10 family, but have been grouped together based on their usage of common receptor subunits and similarities in their target cell profiles and biological functions. Members of the IL20 subfamily facilitate the communication between leukocytes and epithelial cells, thereby enhancing innate defence mechanisms and tissue repair processes at epithelial surfaces. Much of the understanding of this group of cytokines is based on IL22, which is the most studied member (Rutz et al. 2014, Akdis M et al. 2016, Longsdon et al. 2012).

References

Logsdon NJ, Rajashankar KR, Harris BD, Deshpande A & Walter MR (2012). Structural basis for receptor sharing and activation by interleukin-20 receptor-2 (IL-20R2) binding cytokines. Proc. Natl. Acad. Sci. U.S.A., 109, 12704-9.

Komlosi Z, Kucuksezer UC, Frei R, Huitema C, Garbani M, Pezer M, ... Eiwegger T (2016). Interleukins (from IL-1 to IL-38), interferons, transforming growth factor , and TNF-: Receptors, functions, and roles in diseases. J. Allergy Clin. Immunol., 138, 984-1010.

Wang X, Rutz S & Ouyang W (2014). The IL-20 subfamily of cytokines--from host defence to tissue homeostasis. Nat. Rev. Immunol., 14, 783-95.

Edit history

Date	Action	Author
2014-06-04	Authored	Jupe S
2016-01-28	Edited	Jupe S
2016-01-28	Reviewed	Meldal BH
2016-01-28	Created	Jupe S
2017-11-15	Reviewed	Datta SK
2022-03-30	Modified	Weiser JD

4 submitted entities found in this pathway, mapping to 4 Reactome entities

Input	UniProt Id	Input	UniProt Id
ENSG00000115415	P42224	ENSG00000142224	Q9UHD0
ENSG00000162891	Q9NYY1	ENSG00000162892	Q13007

Input	UniProt Id	Input	UniProt Id

21. Interferon alpha/beta signaling (R-HSA-909733)

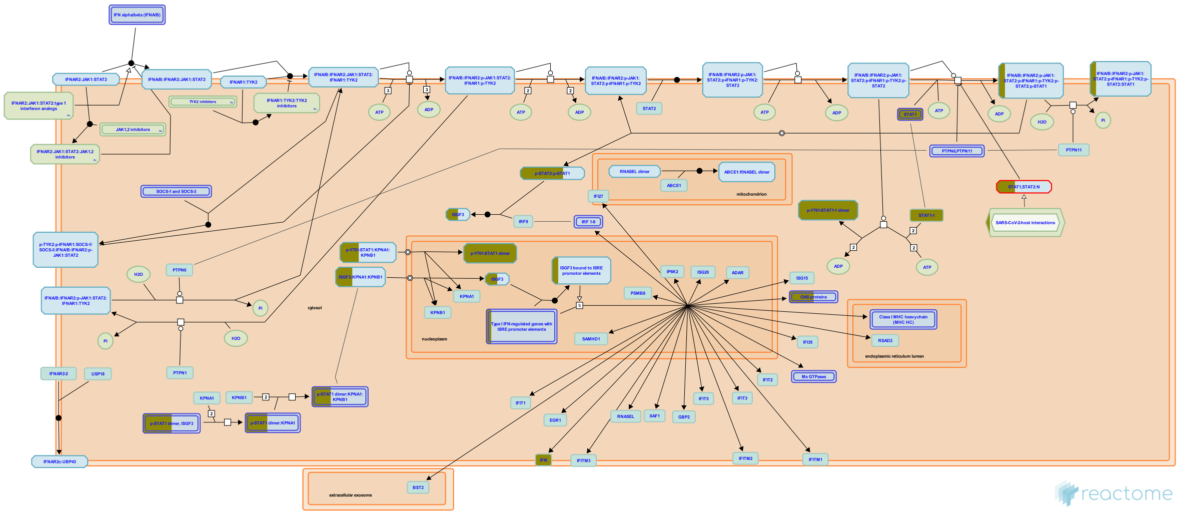

Type I interferons (IFNs) are composed of various genes including IFN alpha (IFNA), beta (IFNB), omega, epsilon, and kappa. In humans the IFNA genes are composed of more than 13 subfamily genes, whereas there is only one IFNB gene. The large family of IFNA/B proteins all bind to a single receptor which is composed of two distinct chains: IFNAR1 and IFNAR2. The IFNA/B stimulation of the IFNA receptor complex leads to the formation of two transcriptional activator complexes: IFNA-activated-factor (AAF), which is a homodimer of STAT1 and IFN-stimulated gene factor 3 (ISGF3), which comprises STAT1, STAT2 and a member of the IRF family, IRF9/P48. AAF mediates activation of the IRF-1 gene by binding to GAS (IFNG-activated site), whereas ISGF3 activates several IFN-inducible genes including IRF3 and IRF7.

References

Stark GR, Darnell JE Jr, Qureshi S, Li X & Leung S (1996). Formation of STAT1-STAT2 heterodimers and their role in the activation of IRF-1 gene transcription by interferon-alpha. J Biol Chem, 271, 5790-4.

Gauzzi MC, Pellegrini S, Velazquez L, McKendry R, Fellous M & Mogensen KE (1996). Interferon-alpha-dependent activation of Tyk2 requires phosphorylation of positive regulatory tyrosines by another kinase. J Biol Chem, 271, 20494-500.

Pellegrini S, Piehler J, Schreiber G & Uzé G (2007). The receptor of the type I interferon family. Curr Top Microbiol Immunol, 316, 71-95.

Gupta S, Greenlund AC, Krolewski JJ, Yan H, Schreiber RD, Schindler CW, ... Krishnan K (1996). Phosphorylated interferon-alpha receptor 1 subunit (IFNaR1) acts as a docking site for the latent form of the 113 kDa STAT2 protein. EMBO J, 15, 1064-74.

Edit history

Date	Action	Author
2010-07-07	Edited	Garapati P V
2010-07-07	Authored	Garapati P V
2010-07-07	Created	Garapati P V
2010-08-17	Reviewed	Abdul-Sater AA, Schindler C
2022-03-30	Modified	Weiser JD

4 submitted entities found in this pathway, mapping to 9 Reactome entities

Input	UniProt Id
ENSG00000111331	Q9Y6K5
ENSG00000115415	P42224, P42224-1, P42224-2

Input	UniProt Id
ENSG00000111335	P29728
ENSG00000126709	P09912

Input	Ensembl Id
ENSG00000111331	ENSG00000111331

Input	Ensembl Id
ENSG00000111335	ENSG00000111335

Input	Ensembl Id
ENSG00000126709	ENSG00000126709

22. Metal sequestration by antimicrobial proteins (R-HSA-6799990)

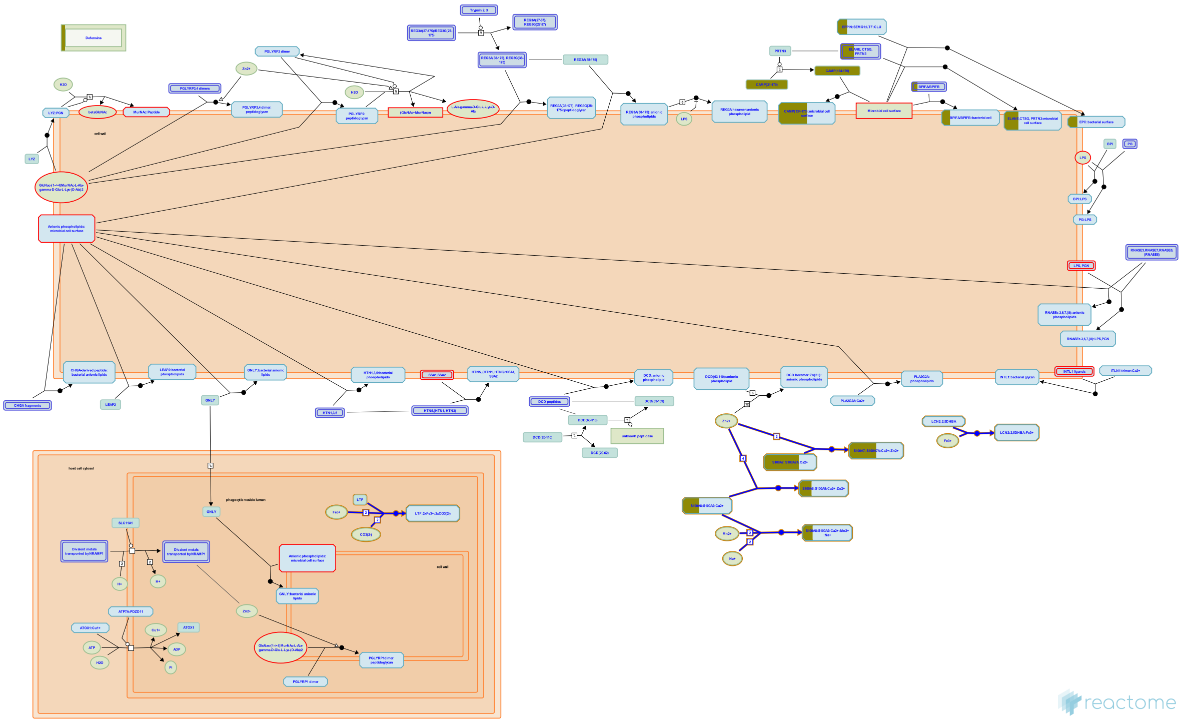

Metals are necessary for all forms of life including microorganisms, evidenced by the fact that metal cations are constituents of approximately 40% of all proteins crystallized to date (Waldron KJ et al. 2009; Foster AW et al. 2014; Guengerich FP 2014, 2015). The ability of microorganisms to maintain the intracellular metal quota is essential and allows microorganisms to adapt to a variety of environments. Accordingly, the ability of the host to control metal quota at inflammation sites can influence host-pathogen interactions. The host may restrict microbial growth either by excluding essential metals from the microbes, by delivery of excess metals to cause toxicity, or by complexing metals in microorganisms (Becker KW & Skaar EP 2014).

References

Guengerich FP (2015). Introduction: Metals in Biology: METALS AT THE HOST-PATHOGEN INTERFACE. J. Biol. Chem., 290, 18943-4.

Skaar EP & Becker KW (2014). Metal limitation and toxicity at the interface between host and pathogen. FEMS Microbiol. Rev., 38, 1235-49.

Edit history

Date	Action	Author
2015-09-26	Created	Shamovsky V
2015-10-05	Authored	Shamovsky V
2016-04-15	Reviewed	Jupe S
2016-08-02	Reviewed	Hains DS
2016-08-15	Edited	Shamovsky V
2022-03-23	Modified	Weiser JD

2 submitted entities found in this pathway, mapping to 3 Reactome entities

Input	UniProt Id	Input	UniProt Id
ENSG00000143546	P05109	ENSG00000143556	P31151, Q86SG5

23. Dectin-2 family (R-HSA-5621480)

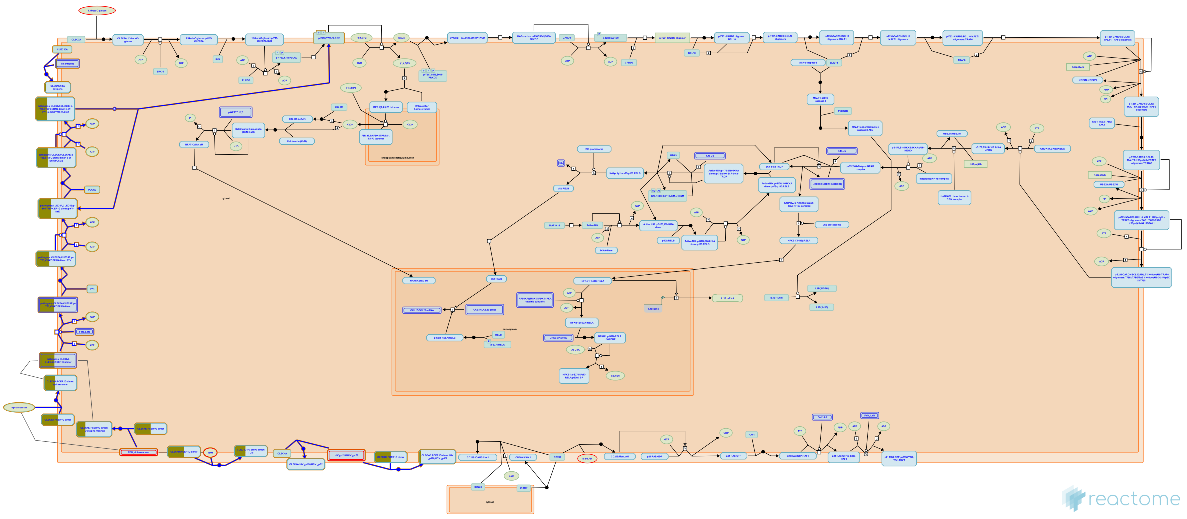

Cellular compartments: plasma membrane.

Dendritic cell-associated C-type lectin-2 (Dectin-2) family of C-type lectin receptors (CLRs) includes Dectin-2 (CLEC6A), blood dendritic antigen 2 (BDCA2/CLEC4C), macrophage C-type lectin (MCL/CLEC4D), Dendritic cell immunoreceptor (DCIR/CLEC4A) and macrophage inducible C-type lectin (Mincle/CLEC4E). These receptors possesses a single extracellular conserved C-type lectin domain (CTLD) with a short cytoplasmic tail that induces intracellular signalling indirectly by binding with the FCERG (High affinity immunoglobulin epsilon receptor subunit gamma) except for DCIR that has a longer cytoplasmic tail with an integral inhibitory signalling motif (Graham & Brown. 2009, Kerschera et al. 2013). CLEC6A (Dectin-2) binds to high mannose containing pathogen-associated molecular patterns (PAMPs) expressed by fungal hyphae, and CLEC4E (mincle) binds to alpha-mannaosyl PAMPs on fungal, mycobacterial and necrotic cell ligands. Both signaling pathways lead to Toll-like receptor (TLR)-independent production of cytokines such as tumor necrosis factor (TNF) and interleukin 6 (IL6). Similarities with Dectin-1 (CLC7A) signaling pathway suggests that both these CLRs couple SYK activation to NF-kB activation using a complex involving CARD9, BCL10 and MALT1 (Geijtenbeek & Gringhuis 2009).

References

Geijtenbeek TB & Gringhuis SI (2009). Signalling through C-type lectin receptors: shaping immune responses. Nat. Rev. Immunol., 9, 465-79.

Kerscher B, Willment JA & Brown GD (2013). The Dectin-2 family of C-type lectin-like receptors: an update. Int. Immunol., 25, 271-7.

Graham LM & Brown GD (2009). The Dectin-2 family of C-type lectins in immunity and homeostasis . Cytokine, 48, 148-55.

Edit history

Date	Action	Author
2014-08-29	Edited	Garapati P V
2014-08-29	Authored	Garapati P V
2014-08-29	Created	Garapati P V

Date	Action	Author
2014-09-02	Reviewed	Geijtenbeek TB
2022-03-23	Modified	Weiser JD

4 submitted entities found in this pathway, mapping to 5 Reactome entities

Input	UniProt Id	Input	UniProt Id
ENSG00000145113	Q99102	ENSG00000166523	Q9ULY5
ENSG00000166527	Q8WXI8	ENSG00000205846	Q6EIG7, Q8WTT0

24. Inhibition of nitric oxide production (R-HSA-9636249)

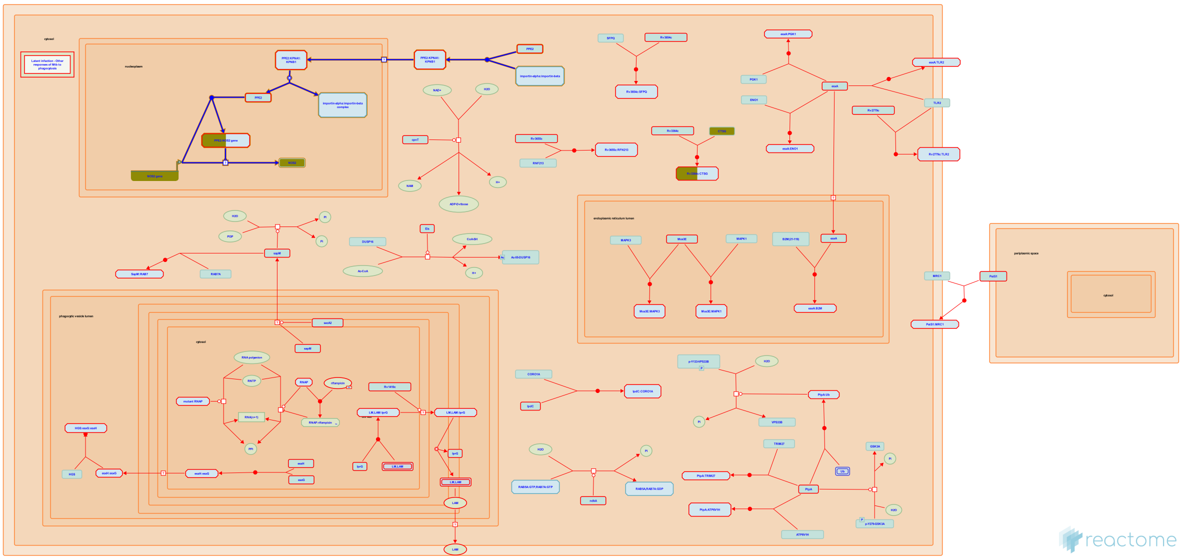

Diseases: tuberculosis.

Phagocytes produce nitric oxide to damage interned bacteria before fusion of the phagosome with lysosomes. While Mtb has several pathways to neutralize NO it also attempts to block the host enzymes used for NO production (Fang 2004, Bhat 2017).

References

Fang FC (2004). Antimicrobial reactive oxygen and nitrogen species: concepts and controversies. Nat Rev Microbiol, 2, 820-32.

Ghosh S, Mukhopadhyay S, Kotturu SK, Bhat KH & Srivastava S (2017). The PPE2 protein of Mycobacterium tuberculosis translocates to host nucleus and inhibits nitric oxide production. Sci Rep , 7, 39706.

Edit history

Date	Action	Author
2019-02-06	Authored	Stephan R
2019-02-12	Edited	Koile I
2019-02-12	Created	Koile I
2019-10-23	Reviewed	Wilkinson RJ, Deffur A
2019-10-31	Modified	Matthews L

1 submitted entities found in this pathway, mapping to 2 Reactome entities

Input	UniProt Id
ENSG00000007171	P35228

Input	Ensembl Id
ENSG00000007171	ENSG00000007171

25. Acyl chain remodelling of PG (R-HSA-1482925)

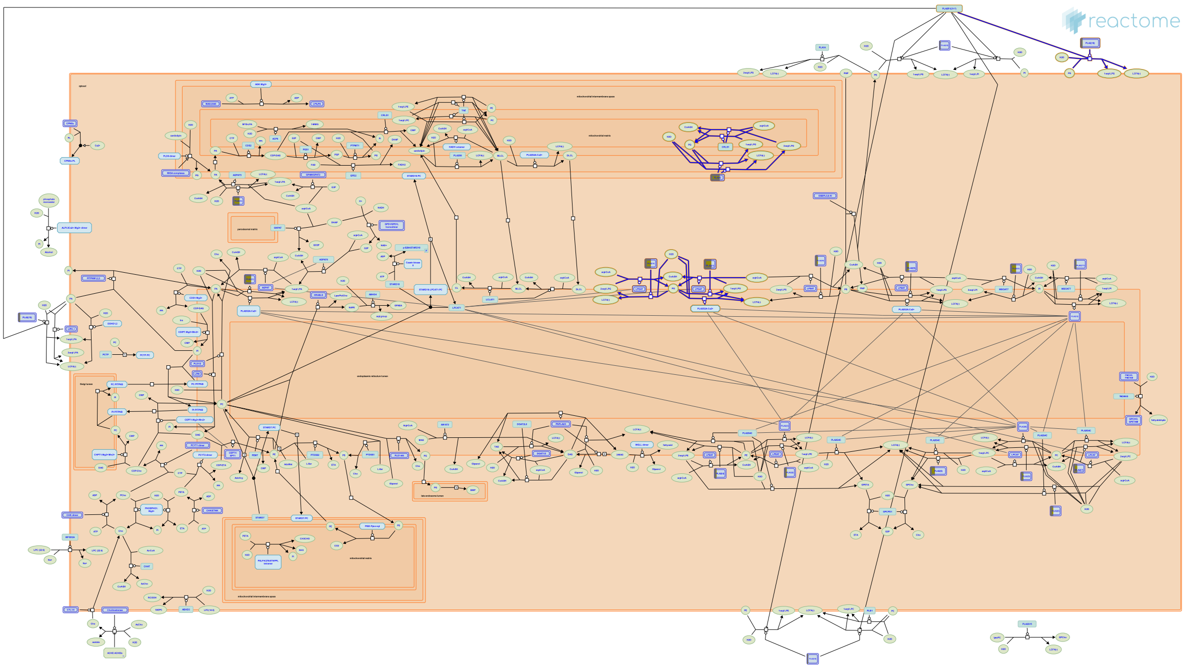

In the acyl chain remodelling pathway (Lands cycle), phosphatidylglycerol (PG) is hydrolyzed by phopholipases and subsequently reacylated by acyltransferases. These cycles modify the fatty acid composition of glycerophospholipids to generate diverse molecules asymmetrically distributed in the cell membrane. The events occur additionally in the inner mitochondria membranes (IM) as well as in the endoplasmic reticulum (ER) membrane (Ghomashchi et al. 2010, Singer et al. 2002, Cao et al. 2008, Yang et al. 2004, Nie et al. 2010).

References

Shi Y, Yang Y & Cao J (2004). Identification and characterization of a gene encoding human LPGAT1, an endoplasmic reticulum-associated lysophosphatidylglycerol acyltransferase. J Biol Chem, 279, 55866-74.

Hao X, Shi Y, Chen D, Chang Z, Nie J & Han X (2010). A novel function of the human CLS1 in phosphatidylglycerol synthesis and remodeling. Biochim Biophys Acta, 1801, 438-45.

Lehr M, Gelb MH, Aloulou A, Naika GS, Bollinger JG, Ghomashchi F & Leslie CC (2010). Interfacial kinetic and binding properties of mammalian group IVB phospholipase A2 (cPLA2beta) and comparison with the other cPLA2 isoforms. J Biol Chem, 285, 36100-11.

Revett T, Tobin JF, Cao J, Li D, Shan D, Gimeno RE, ... Liu W (2008). Molecular identification of a novel mammalian brain isoform of acyl-CoA:lysophospholipid acyltransferase with prominent ethanolamine lysophospholipid acylating activity, LPEAT2. J Biol Chem, 283, 19049-57.

Gelb MH, Le Calvez C, Ghomashchi F, Rouault M, Singer AG, Sadilek M, ... Nguyen E (2002). Interfacial kinetic and binding properties of the complete set of human and mouse groups I, II, V, X, and XII secreted phospholipases A2. J Biol Chem, 277, 48535-49.

Edit history

Date	Action	Author
2011-08-12	Edited	Williams MG
2011-08-12	Created	Williams MG

Date	Action	Author
2011-09-14	Authored	Williams MG
2022-03-23	Modified	Weiser JD

3 submitted entities found in this pathway, mapping to 4 Reactome entities

Input	UniProt Id	Input	UniProt Id	Input	UniProt Id
ENSG00000158786	Q9BZM2	ENSG00000159337	Q86XP0	ENSG00000243708	P0C869, Q86XP0

6. Identifiers found

Below is a list of the input identifiers that have been found or mapped to an equivalent element in Reactome, classified by resource.

135 of the submitted entities were found, mapping to 175 Reactome entities

Input	UniProt Id	Input	UniProt Id	Input	UniProt Id
ENSG00000004468	P28907	ENSG00000007171	P35228	ENSG00000049249	Q07011
ENSG00000050730	Q96KP6	ENSG00000057149	P29508	ENSG00000090104	Q08116
ENSG00000092295	P22735	ENSG00000093134	Q9NY84	ENSG00000100342	O14791
ENSG00000100433	P57789	ENSG00000100453	P08311	ENSG00000101670	Q9Y5X9
ENSG00000103257	Q01650	ENSG00000103313	O15553	ENSG00000105173	P24864
ENSG00000108688	P13500	ENSG00000110680	P01258, P06881, P10092	ENSG00000110944	Q9NPF7
ENSG00000111331	Q9Y6K5	ENSG00000111335	P29728	ENSG00000112115	Q16552
ENSG00000112116	Q96PD4	ENSG00000112299	O95497	ENSG00000113302	P29460
ENSG00000113356	O15318	ENSG00000113749	P25021	ENSG00000114251	P41221
ENSG00000115415	P42224, P42224-1, P42224-2	ENSG00000115488	Q9Y3R4	ENSG00000119508	Q92570
ENSG00000120217	Q9NZQ7	ENSG00000124233	P04279	ENSG00000124256	Q9H171
ENSG00000124731	Q9NP99	ENSG00000126353	P32248	ENSG00000126709	P09912
ENSG00000130829	Q99956	ENSG00000134640	P49286	ENSG00000134755	Q02487
ENSG00000134757	P32926	ENSG00000134827	P20061	ENSG00000136688	Q9NZH8
ENSG00000136694	Q9UHA7	ENSG00000136695	Q9UBH0	ENSG00000137440	Q14512
ENSG00000137757	P51878	ENSG00000138496	Q8IXQ6	ENSG00000138642	Q8IVU3
ENSG00000139572	Q9NQS5	ENSG00000139629	Q8NCL4	ENSG00000140379	Q16548
ENSG00000140519	Q9UBD6	ENSG00000142224	Q9UHD0	ENSG00000143320	P29373
ENSG00000143546	P05109	ENSG00000143556	P31151, Q86SG5	ENSG00000145113	Q99102
ENSG00000145287	Q9NZF1	ENSG00000150244	Q8IWZ4	ENSG00000150337	P12314, Q92637
ENSG00000150551	Q8N2G4	ENSG00000151012	Q9UPY5	ENSG00000151790	P48775
ENSG00000153976	Q9Y663	ENSG00000156234	P02775	ENSG00000156282	P56750
ENSG00000156413	P51993, Q11128	ENSG00000158125	P47989	ENSG00000158786	Q9BZM2
ENSG00000158859	O75173	ENSG00000159337	Q86XP0	ENSG00000159516	Q9BYE4
ENSG00000162572	P51172	ENSG00000162891	Q9NYY1	ENSG00000162892	Q13007
ENSG00000163568	O14862	ENSG00000163735	P42830	ENSG00000163739	P09341
ENSG00000164047	P49913	ENSG00000166523	Q9ULY5	ENSG00000166527	Q8WXI8
ENSG00000167618	Q6ISS4	ENSG00000167941	Q9BQB4	ENSG00000168412	P48039
ENSG00000169174	Q8NBP7	ENSG00000169245	P02778	ENSG00000169248	O14625
ENSG00000170465	P04259, P48668	ENSG00000171049	P21462, P25090	ENSG00000171051	P21462, P62942
ENSG00000172350	Q9H172	ENSG00000172602	Q92730	ENSG00000174502	Q7LBE3
ENSG00000175426	P29120	ENSG00000175592	P15407	ENSG00000176749	Q15078
ENSG00000176797	P81534	ENSG00000176920	Q10981	ENSG00000176928	Q9P109
ENSG00000177243	P81534	ENSG00000177257	O15263	ENSG00000182585	Q6UW88
ENSG00000183347	Q6ZN66	ENSG00000183696	Q16831	ENSG00000185069	Q01546
ENSG00000185962	Q5TA76	ENSG00000185966	Q5T5B0	ENSG00000186191	P59827
ENSG00000186431	P24071	ENSG00000186442	P12035	ENSG00000186832	P08779
ENSG00000186871	Q2NKX8	ENSG00000187238	Q5TA77	ENSG00000187258	Q6W5P4
ENSG00000188037	P35523	ENSG00000188389	Q15116	ENSG00000188404	P14151

Input	UniProt Id	Input	UniProt Id	Input	UniProt Id
ENSG00000189013	Q99706	ENSG00000196805	P35325	ENSG00000197249	P01009
ENSG00000198019	Q92637	ENSG00000198805	P00491	ENSG00000203747	P08637
ENSG00000203785	P22531	ENSG00000205420	P04264	ENSG00000205846	Q6EIG7, Q8WTT0
ENSG00000214643	Q30KQ1	ENSG00000227471	C9JRZ8	ENSG00000241794	P35326
ENSG00000243708	P0C869, Q86XP0	ENSG00000244057	Q5T5A8	ENSG00000244094	Q96RM1
ENSG00000262406	P39900	ENSG00000268104	Q9UN76	ENSG00000277632	P10147, P16619

Input	Ensembl Id	Input	Ensembl Id	Input	Ensembl Id
ENSG00000007171	ENSG00000007171	ENSG00000100453	ENSG00000100453	ENSG00000105173	ENSG00000105173
ENSG00000111331	ENSG00000111331	ENSG00000111335	ENSG00000111335	ENSG00000112115	ENSG00000112115
ENSG00000112116	ENSG00000112116	ENSG00000113302	ENSG00000113302	ENSG00000115415	ENSG00000115415
ENSG00000119508	ENSG00000119508	ENSG00000120217	ENSG00000120217	ENSG00000126709	ENSG00000126709
ENSG00000140379	ENSG00000140379	ENSG00000150244	ENSG00000150244	ENSG00000150337	ENSG00000150337
ENSG00000151012	ENSG00000151012	ENSG00000163739	ENSG00000163739	ENSG00000169245	ENSG00000169245
ENSG00000171051	ENSG00000171051	ENSG00000176749	ENSG00000176749	ENSG00000183347	ENSG00000183347
ENSG00000198019	ENSG00000198019	ENSG00000243708	ENSG00000243708	ENSG00000277632	ENSG00000277632
