# Supplementary material for: Biology-inspired graph neural network encodes reactome and reveals biochemical reactions of disease
Source: Patterns (N Y). 2023 May 22;4(7):100758. doi: 10.1016/j.patter.2023.100758 (PMC10382942; doi:10.1016/j.patter.2023.100758)
Supplement: Data S7. Reactome pathway relaxed threshold enrichment report [file mmc8.pdf]

Pathway Analysis Report

This report contains the pathway analysis results for the submitted sample ''. Analysis was performed against Reactome version 80 on 06/05/2022. The web link to these results is:

https://reactome.org/PathwayBrowser/#/ANALYSIS=MjAyMjA1MDYyMjMyMjNfMzEwMzA%3D

Please keep in mind that analysis results are temporarily stored on our server. The storage period depends on usage of the service but is at least 7 days. As a result, please note that this URL is only valid for a limited time period and it might have expired.

Table of Contents

1. Introduction
2. Properties
3. Genome-wide overview
4. Most significant pathways
5. Pathways details
6. Identifiers found
7. Identifiers not found

1. Introduction

Reactome is a curated database of pathways and reactions in human biology. Reactions can be considered as pathway 'steps'. Reactome defines a 'reaction' as any event in biology that changes the state of a biological molecule. Binding, activation, translocation, degradation and classical biochemical events involving a catalyst are all reactions. Information in the database is authored by expert biologists, entered and maintained by Reactome's team of curators and editorial staff. Reactome content frequently cross-references other resources e.g. NCBI, Ensembl, UniProt, KEGG (Gene and Compound), ChEBI, PubMed and GO. Orthologous reactions inferred from annotation for Homo sapiens are available for 17 non-human species including mouse, rat, chicken, puffer fish, worm, fly, yeast, rice, and Arabidopsis. Pathways are represented by simple diagrams following an SBGN-like format.

Reactome's annotated data describe reactions possible if all annotated proteins and small molecules were present and active simultaneously in a cell. By overlaying an experimental dataset on these annotations, a user can perform a pathway over-representation analysis. By overlaying quantitative expression data or time series, a user can visualize the extent of change in affected pathways and its progression. A binomial test is used to calculate the probability shown for each result, and the p-values are corrected for the multiple testing (Benjamini–Hochberg procedure) that arises from evaluating the submitted list of identifiers against every pathway.

To learn more about our Pathway Analysis, please have a look at our relevant publications:

Fabregat A, Sidiropoulos K, Garapati P, Gillespie M, Hausmann K, Haw R, … D'Eustachio P (2016). The reactome pathway knowledgebase. Nucleic Acids Research, 44(D1), D481–D487. https://doi.org/10.1093/nar/gkv1351.

Fabregat A, Sidiropoulos K, Viteri G, Forner O, Marin-Garcia P, Arnau V, … Hermjakob H (2017). Reactome pathway analysis: a high-performance in-memory approach. BMC Bioinformatics, 18.

2. Properties

- This is an **overrepresentation** analysis: A statistical (hypergeometric distribution) test that determines whether certain Reactome pathways are over-represented (enriched) in the submitted data. It answers the question ‘Does my list contain more proteins for pathway X than would be expected by chance?’ This test produces a probability score, which is corrected for false discovery rate using the Benjamani-Hochberg method.
- 903 out of 904 identifiers in the sample were found in Reactome, where 1617 pathways were hit by at least one of them.
- All non-human identifiers have been converted to their human equivalent.
- This report is filtered to show only results for species 'Homo sapiens' and resource 'all resources'.
- The unique ID for this analysis (token) is MjAyMjA1MDYyMjMyMjNfMzEwMzA%3D. This ID is valid for at least 7 days in Reactome’s server. Use it to access Reactome services with your data.

3. Genome-wide overview

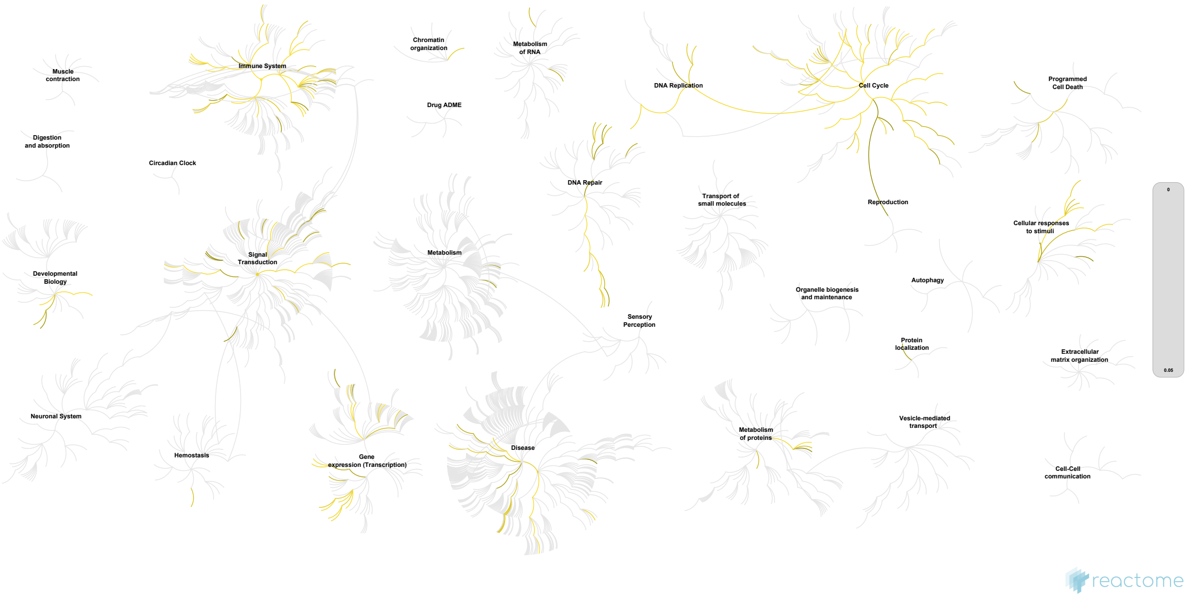

This figure shows a genome-wide overview of the results of your pathway analysis. Reactome pathways are arranged in a hierarchy. The center of each of the circular "bursts" is the root of one top-level pathway, for example "DNA Repair". Each step away from the center represents the next level lower in the pathway hierarchy. The color code denotes over-representation of that pathway in your input dataset. Light grey signifies pathways which are not significantly over-represented.

4. Most significant pathways

The following table shows the 25 most relevant pathways sorted by p-value.

Pathway name	Entities				Reactions	
	found	ratio	p-value	FDR*	found	ratio
Endosomal/Vacuolar pathway	36 / 82	0.005	1.11e-16	3.23e-14	4 / 4	2.90e-04
Interferon alpha/beta signaling	67 / 190	0.013	1.11e-16	3.23e-14	23 / 24	0.002
Interferon Signaling	97 / 395	0.026	1.11e-16	3.23e-14	51 / 71	0.005
Interferon gamma signaling	64 / 250	0.017	1.11e-16	3.23e-14	9 / 16	0.001
Immune System	409 / 2,691	0.178	1.11e-16	3.23e-14	874 / 1,625	0.118
Cytokine Signaling in Immune system	222 / 1,094	0.072	1.11e-16	3.23e-14	378 / 710	0.051
Signaling by Interleukins	112 / 645	0.043	4.44e-16	1.11e-13	274 / 493	0.036
Immunoregulatory interactions between a Lymphoid and a non-Lymphoid cell	69 / 316	0.021	1.09e-14	2.37e-12	29 / 44	0.003
Antigen processing-Cross presentation	52 / 195	0.013	1.38e-14	2.60e-12	19 / 23	0.002
Antigen Presentation: Folding, assembly and peptide loading of class I MHC	38 / 108	0.007	1.49e-14	2.60e-12	16 / 16	0.001
Neutrophil degranulation	87 / 480	0.032	1.17e-13	1.86e-11	10 / 10	7.25e-04
ER-Phagosome pathway	46 / 173	0.011	5.09e-13	7.39e-11	9 / 10	7.25e-04
Adaptive Immune System	142 / 1,012	0.067	7.94e-13	1.06e-10	188 / 264	0.019
SARS-CoV-2 activates/modulates innate and adaptive immune responses	51 / 226	0.015	1.07e-11	1.34e-09	16 / 47	0.003
Cell Cycle	109 / 734	0.049	1.95e-11	2.27e-09	315 / 451	0.033
Innate Immune System	167 / 1,338	0.089	6.49e-11	7.08e-09	335 / 710	0.051
Mitotic G1 phase and G1/S transition	41 / 174	0.012	3.28e-10	3.34e-08	71 / 101	0.007
Cell Cycle, Mitotic	90 / 596	0.039	5.57e-10	5.41e-08	252 / 352	0.026
Cell Cycle Checkpoints	53 / 279	0.018	1.73e-09	1.59e-07	32 / 56	0.004
Interleukin-10 signaling	26 / 86	0.006	4.55e-09	3.96e-07	15 / 15	0.001
Chemokine receptors bind chemokines	21 / 57	0.004	5.02e-09	4.17e-07	10 / 19	0.001
SARS-CoV-2-host interactions	56 / 314	0.021	5.30e-09	4.18e-07	18 / 67	0.005
G1/S Transition	35 / 150	0.01	8.08e-09	6.14e-07	46 / 61	0.004
Formation of the cornified envelope	33 / 138	0.009	1.21e-08	8.72e-07	16 / 27	0.002
G1/S-Specific Transcription	17 / 43	0.003	5.28e-08	3.70e-06	16 / 28	0.002

* False Discovery Rate

5. Pathways details

For every pathway of the most significant pathways, we present its diagram, as well as a short summary, its bibliography and the list of inputs found in it.

1. Endosomal/Vacuolar pathway (R-HSA-1236977)

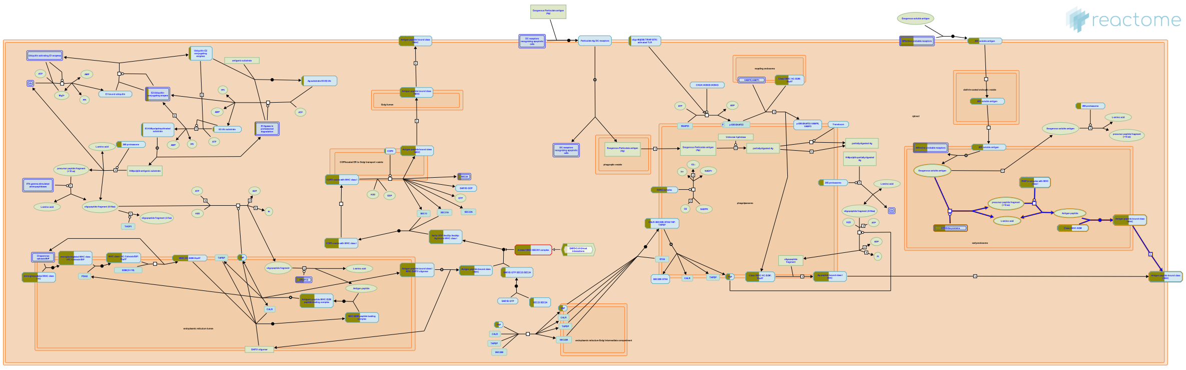

Cellular compartments: early endosome.

Some antigens are cross-presented through a vacuolar mechanism that involves generation of antigenic peptides and their loading on to MHC-I molecules within the endosomal compartment in a proteasome and TAP-independent manner. Antigens within the endosome are processed by cathepsin S and other proteases into antigenic peptides. Loading of these peptides onto MHC-I molecules occurs directly within early and late endosomal compartments. Why certain antigens are cross-presented exclusively by the cytosolic pathway while others use the vacuolar pathway is unknown. It may be because some epitopes cannot be generated by endosomal proteolysis, or are completely destroyed. Alternatively, the physical form of the antigen may influence its accessibility to the endosomal or vacuolar pathways (Shen et al. 2004).

References

Banchereau J, Montes M, Mellman I, Xue Y, Connolly JE, Clayton S, ... Di Pucchio T (2008). Direct proteasome-independent cross-presentation of viral antigen by plasmacytoid dendritic cells on major histocompatibility complex class I. Nat Immunol, 9, 551-7.

Edit history

Date	Action	Author
2011-03-28	Edited	Garapati P V
2011-03-28	Authored	Garapati P V
2011-03-28	Created	Garapati P V
2011-05-13	Reviewed	Desjardins M, English L
2022-03-23	Modified	Weiser JD

2 submitted entities found in this pathway, mapping to 36 Reactome entities

Input	UniProt Id	Input	UniProt Id
ENSG00000163131	P25774	ENSG00000234745	P01889, P03989, P10319, P18463, P18464, P18465, P30460, P30461, P30462, P30464, P30466, P30475, P30479, P30480, P30481, P30483, P30484, P30485, P30486, P30487, P30488, P30490, P30491, P30492, P30493, P30495, P30498, P30685, Q04826, Q29718, Q29836, Q29940, Q31610, Q31612, Q95365

2. Interferon alpha/beta signaling (R-HSA-909733)

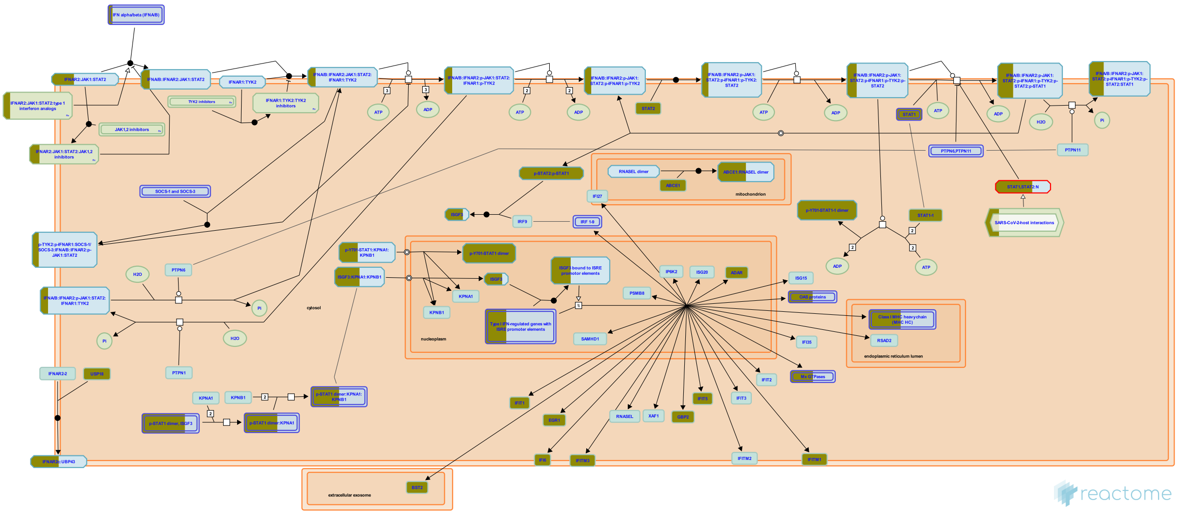

Type I interferons (IFNs) are composed of various genes including IFN alpha (IFNA), beta (IFNB), omega, epsilon, and kappa. In humans the IFNA genes are composed of more than 13 subfamily genes, whereas there is only one IFNB gene. The large family of IFNA/B proteins all bind to a single receptor which is composed of two distinct chains: IFNAR1 and IFNAR2. The IFNA/B stimulation of the IFNA receptor complex leads to the formation of two transcriptional activator complexes: IFNA-activated-factor (AAF), which is a homodimer of STAT1 and IFN-stimulated gene factor 3 (ISGF3), which comprises STAT1, STAT2 and a member of the IRF family, IRF9/P48. AAF mediates activation of the IRF-1 gene by binding to GAS (IFNG-activated site), whereas ISGF3 activates several IFN-inducible genes including IRF3 and IRF7.

References

Stark GR, Darnell JE Jr, Qureshi S, Li X & Leung S (1996). Formation of STAT1-STAT2 heterodimers and their role in the activation of IRF-1 gene transcription by interferon-alpha. J Biol Chem, 271, 5790-4.

Gauzzi MC, Pellegrini S, Velazquez L, McKendry R, Fellous M & Mogensen KE (1996). Interferon-alpha-dependent activation of Tyk2 requires phosphorylation of positive regulatory tyrosines by another kinase. J Biol Chem, 271, 20494-500.

Pellegrini S, Piehler J, Schreiber G & Uzé G (2007). The receptor of the type I interferon family. Curr Top Microbiol Immunol, 316, 71-95.

Gupta S, Greenlund AC, Krolewski JJ, Yan H, Schreiber RD, Schindler CW, ... Krishnan K (1996). Phosphorylated interferon-alpha receptor 1 subunit (IFNaR1) acts as a docking site for the latent form of the 113 kDa STAT2 protein. EMBO J, 15, 1064-74.

Edit history

Date	Action	Author
2010-07-07	Edited	Garapati P V
2010-07-07	Authored	Garapati P V
2010-07-07	Created	Garapati P V
2010-08-17	Reviewed	Abdul-Sater AA, Schindler C
2022-03-30	Modified	Weiser JD

18 submitted entities found in this pathway, mapping to 67 Reactome entities

Input	UniProt Id	Input	UniProt Id	Input	UniProt Id
ENSG00000111331	Q9Y6K5	ENSG00000111335	P29728	ENSG00000115415	P42224, P42224-1, P42224-2
ENSG00000120738	P18146	ENSG00000126709	P09912	ENSG00000130303	Q10589
ENSG00000142089	Q01628	ENSG00000152778	Q13325	ENSG00000160710	P55265
ENSG00000162645	P32456	ENSG00000164163	P61221	ENSG00000170581	P52630
ENSG00000183486	P20592	ENSG00000184979	Q9UMW8	ENSG00000185745	P09914
ENSG00000185885	P13164	ENSG00000233816	P01562	ENSG00000234745	P01889, P03989, P10319, P18463, P18464, P18465, P30460, P30461, P30462, P30464, P30466, P30475, P30479, P30480, P30481, P30483, P30484, P30485, P30486, P30487, P30488, P30490, P30491, P30492, P30493, P30495, P30498, P30685, Q04826, Q29718, Q29836, Q29940, Q31610, Q31612, Q95365

Input	Ensembl Id	Input	Ensembl Id	Input	Ensembl Id
ENSG00000111331	ENSG00000111331	ENSG00000111335	ENSG00000111335	ENSG00000120738	ENSG00000120738
ENSG00000126709	ENSG00000126709	ENSG00000130303	ENSG00000130303	ENSG00000142089	ENSG00000142089
ENSG00000152778	ENSG00000152778	ENSG00000160710	ENSG00000160710	ENSG00000162645	ENSG00000162645
ENSG00000183486	ENSG00000183486	ENSG00000185745	ENSG00000185745	ENSG00000185885	ENSG00000185885
ENSG00000234745	ENSG00000234745				

3. Interferon Signaling (R-HSA-913531)

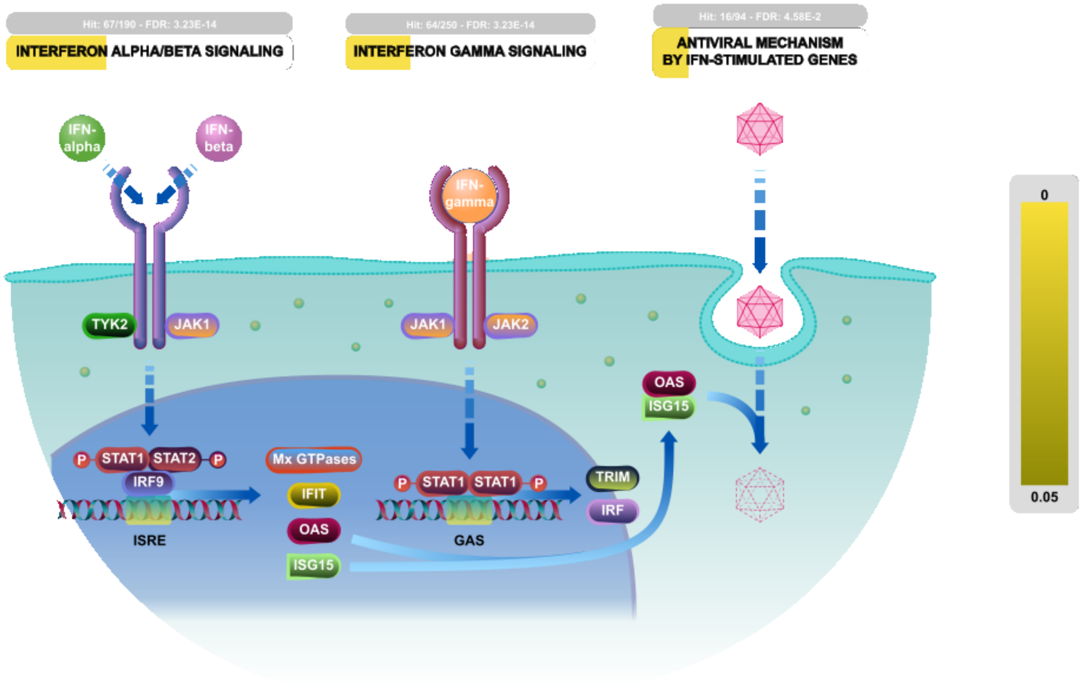

Interferons (IFNs) are cytokines that play a central role in initiating immune responses, especially antiviral and antitumor effects. There are three types of IFNs:Type I (IFN-alpha, -beta and others, such as omega, epsilon, and kappa), Type II (IFN-gamma) and Type III (IFN-lamda). In this module we are mainly focusing on type I IFNs alpha and beta and type II IFN-gamma. Both type I and type II IFNs exert their actions through cognate receptor complexes, IFNAR and IFNGR respectively, present on cell surface membranes. Type I IFNs are broadly expressed heterodimeric receptors composed of the IFNAR1 and IFNAR2 subunits, while the type II IFN receptor consists of IFNGR1 and IFNGR2. Type III interferon lambda has three members: lamda1 (IL-29), lambda2 (IL-28A), and lambda3 (IL-28B) respectively. IFN-lambda signaling is initiated through unique heterodimeric receptor composed of IFN-LR1/IF-28Ralpha and IL10R2 chains.

Type I IFNs typically recruit JAK1 and TYK2 proteins to transduce their signals to STAT1 and 2; in combination with IRF9 (IFN-regulatory factor 9), these proteins form the heterotrimeric complex ISGF3. In nucleus ISGF3 binds to IFN-stimulated response elements (ISRE) to promote gene induction.

Type II IFNs in turn rely upon the activation of JAKs 1 and 2 and STAT1. Once activated, STAT1 dimerizes to form the transcriptional regulator GAF (IFNG activated factor) and this binds to the IFNG activated sequence (GAS) elements and initiate the transcription of IFNG-responsive genes.

Like type I IFNs, IFN-lambda recruits TYK2 and JAK1 kinases and then promote the phosphorylation of STAT1/2, and induce the ISRE3 complex formation.

References

Schroder K, Ravasi T, Hume DA & Hertzog PJ (2004). Interferon-gamma: an overview of signals, mechanisms and functions. J Leukoc Biol, 75, 163-89.

Platanias LC (2005). Mechanisms of type-I- and type-II-interferon-mediated signalling. Nat Rev Immunol, 5, 375-86.

Gough DJ, Levy DE, Clarke CJ & Johnstone RW (2008). IFNgamma signaling-does it mean JAK-STAT?. Cytokine Growth Factor Rev, 19, 383-94.

Ferreira PC, Bonjardim CA & Kroon EG (2009). Interferons: signaling, antiviral and viral evasion. Immunol Lett, 122, 1-11.

Platanias LC & Uddin S (2004). Mechanisms of type-I interferon signal transduction. J Biochem Mol Biol, 37, 635-41.

Edit history

Date	Action	Author
2010-07-07	Edited	Garapati P V
2010-07-07	Authored	Garapati P V
2010-07-16	Created	Garapati P V
2010-08-17	Reviewed	Abdul-Sater AA, Schindler C
2022-03-23	Modified	Weiser JD

37 submitted entities found in this pathway, mapping to 98 Reactome entities

Input	UniProt Id	Input	UniProt Id	Input	UniProt Id
ENSG00000055332	P19525	ENSG00000093000	Q9UKX7	ENSG00000101871	O15344
ENSG00000111331	Q9Y6K5	ENSG00000111335	P29728	ENSG00000115415	P42224, P42224-1, P42224-2
ENSG00000120738	P18146	ENSG00000126709	P09912	ENSG00000130303	Q10589
ENSG00000132109	P19474	ENSG00000138646	Q9UII4	ENSG00000140464	P29590
ENSG00000141543	P38919	ENSG00000142089	Q01628	ENSG00000150244	Q8IWZ4
ENSG00000150337	P12314, Q92637	ENSG00000151247	P06730	ENSG00000152778	Q13325
ENSG00000156587	O14933	ENSG00000160710	P55265	ENSG00000162645	P32456
ENSG00000162654	Q96PP9	ENSG00000164163	P61221	ENSG00000170581	P52630
ENSG00000175354	P17706-2	ENSG00000177889	P61088	ENSG00000182481	P52292
ENSG00000183347	Q6ZN66	ENSG00000183486	P20592	ENSG00000184979	Q3LFD5, Q9UMW8
ENSG00000185745	P09914	ENSG00000185885	P13164	ENSG00000198019	Q92637
ENSG00000213512	Q8N8V2	ENSG00000233816	P01562	ENSG00000234745	P01889, P03989, P10319, P18463, P18464, P18465, P30460, P30461, P30462, P30464, P30466, P30475, P30479, P30480, P30481, P30483, P30484, P30485, P30486, P30487, P30488, P30490, P30491, P30492, P30493, P30495, P30498, P30685, Q04826, Q29718, Q29836, Q29940, Q31610, Q31612, Q95365
ENSG00000237541	P01906				

Input	Ensembl Id	Input	Ensembl Id	Input	Ensembl Id
ENSG00000101871	ENSG00000101871	ENSG00000111331	ENSG00000111331	ENSG00000111335	ENSG00000111335
ENSG00000120738	ENSG00000120738	ENSG00000126709	ENSG00000126709	ENSG00000130303	ENSG00000130303
ENSG00000132109	ENSG00000132109	ENSG00000140464	ENSG00000140464	ENSG00000142089	ENSG00000142089
ENSG00000150244	ENSG00000150244	ENSG00000150337	ENSG00000150337	ENSG00000152778	ENSG00000152778
ENSG00000160710	ENSG00000160710	ENSG00000162645	ENSG00000162645	ENSG00000162654	ENSG00000162654
ENSG00000183347	ENSG00000183347	ENSG00000183486	ENSG00000183486	ENSG00000185745	ENSG00000185745

Input	Ensembl Id	Input	Ensembl Id	Input	Ensembl Id
ENSG00000185885	ENSG00000185885	ENSG00000198019	ENSG00000198019	ENSG00000213512	ENSG00000213512
ENSG00000234745	ENSG00000234745	ENSG00000237541	ENSG00000237541		

4. Interferon gamma signaling (R-HSA-877300)

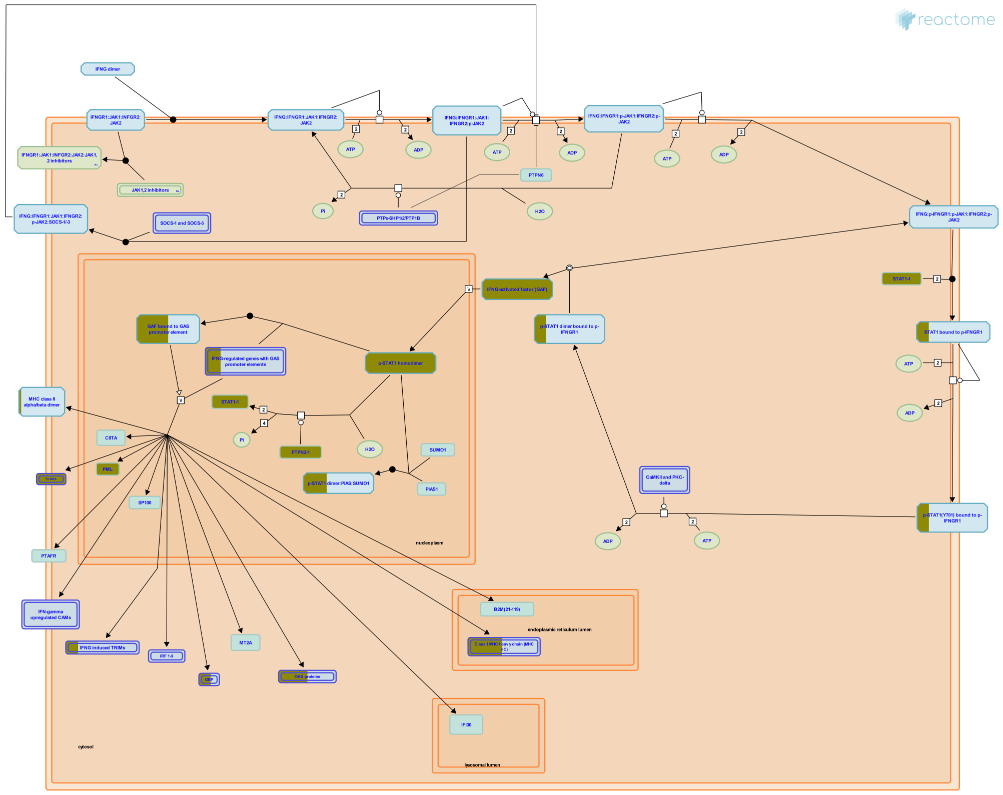

Interferon-gamma (IFN-gamma) belongs to the type II interferon family and is secreted by activated immune cells-primarily T and NK cells, but also B-cells and APC. INFG exerts its effect on cells by interacting with the specific IFN-gamma receptor (IFNGR). IFNGR consists of two chains, namely IFNGR1 (also known as the IFNGR alpha chain) and IFNGR2 (also known as the IFNGR beta chain). IFNGR1 is the ligand binding receptor and is required but not sufficient for signal transduction, whereas IFNGR2 do not bind IFNG independently but mainly plays a role in IFNG signaling and is generally the limiting factor in IFNG responsiveness. Both IFNGR chains lack intrinsic kinase/phosphatase activity and thus rely on other signaling proteins like Janus-activated kinase 1 (JAK1), JAK2 and Signal transducer and activator of transcription 1 (STAT-1) for signal transduction. IFNGR complex in its resting state is a preformed tetramer and upon IFNG association undergoes a conformational change. This conformational change induces the phosphorylation and activation of JAK1, JAK2, and STAT1 which in turn induces genes containing the gamma-interferon activation sequence (GAS) in the promoter.

References

Schroder K, Ravasi T, Hume DA & Hertzog PJ (2004). Interferon-gamma: an overview of signals, mechanisms and functions. J Leukoc Biol, 75, 163-89.

Aguet M, Bach EA & Schreiber RD (1997). The IFN gamma receptor: a paradigm for cytokine receptor signaling. Annu Rev Immunol, 15, 563-91.

Gough DJ, Levy DE, Clarke CJ & Johnstone RW (2008). IFNgamma signaling-does it mean JAK-STAT?. Cytokine Growth Factor Rev, 19, 383-94.

Izotova LS, Garotta G, Muthukumaran G, Kotenko SV, Cook JR & Pestka S (1997). The interferon gamma (IFN-gamma) receptor: a paradigm for the multichain cytokine receptor. Cytokine Growth Factor Rev, 8, 189-206.

Edit history

Date	Action	Author
2010-06-08	Edited	Garapati P V
2010-06-08	Authored	Garapati P V
2010-06-11	Created	Garapati P V
2010-08-17	Reviewed	Abdul-Sater AA, Schindler C
2022-03-30	Modified	Weiser JD

16 submitted entities found in this pathway, mapping to 65 Reactome entities

Input	UniProt Id	Input	UniProt Id	Input	UniProt Id
ENSG00000101871	O15344	ENSG00000111331	Q9Y6K5	ENSG00000111335	P29728
ENSG00000115415	P42224-1	ENSG00000132109	P19474	ENSG00000140464	P29590
ENSG00000150244	Q8IWZ4	ENSG00000150337	P12314, Q92637	ENSG00000162645	P32456
ENSG00000162654	Q96PP9	ENSG00000175354	P17706-2	ENSG00000183347	Q6ZN66
ENSG00000198019	Q92637	ENSG00000213512	Q8N8V2	ENSG00000234745	P01889, P03989, P10319, P18463, P18464, P18465, P30460, P30461, P30462, P30464, P30466, P30475, P30479, P30480, P30481, P30483, P30484, P30485, P30486, P30487, P30488, P30490, P30491, P30492, P30493, P30495, P30498, P30685, Q04826, Q29718, Q29836, Q29940, Q31610, Q31612, Q95365
ENSG00000237541	P01906				

Input	Ensembl Id	Input	Ensembl Id	Input	Ensembl Id
ENSG00000101871	ENSG00000101871	ENSG00000111331	ENSG00000111331	ENSG00000111335	ENSG00000111335
ENSG00000132109	ENSG00000132109	ENSG00000140464	ENSG00000140464	ENSG00000150244	ENSG00000150244
ENSG00000150337	ENSG00000150337	ENSG00000162645	ENSG00000162645	ENSG00000162654	ENSG00000162654
ENSG00000183347	ENSG00000183347	ENSG00000198019	ENSG00000198019	ENSG00000213512	ENSG00000213512
ENSG00000234745	ENSG00000234745	ENSG00000237541	ENSG00000237541		

5. Immune System (R-HSA-168256)

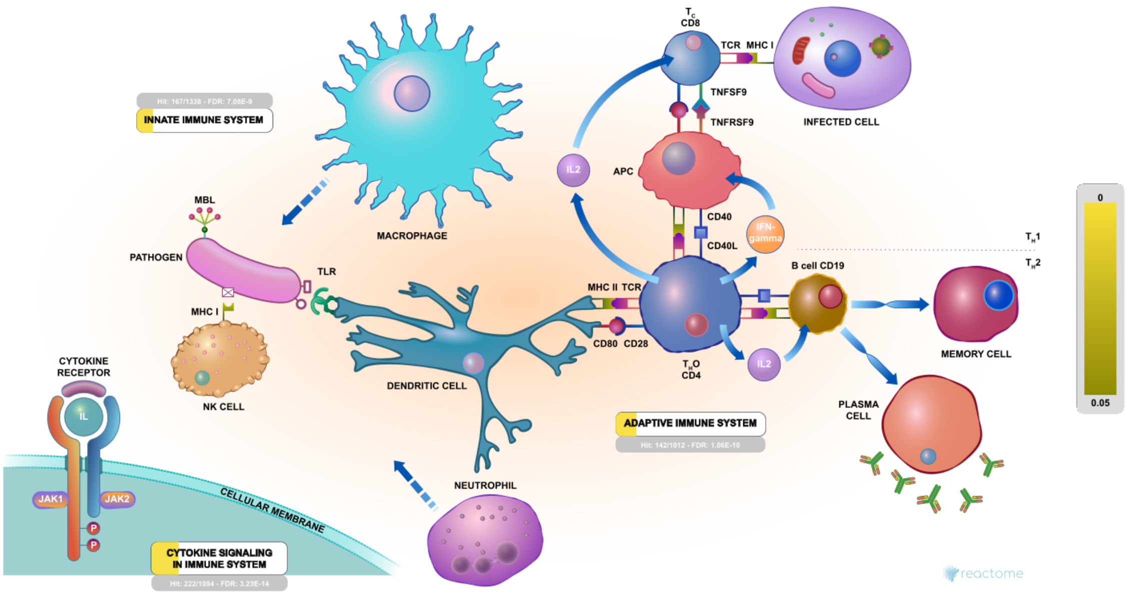

Humans are exposed to millions of potential pathogens daily, through contact, ingestion, and inhalation. Our ability to avoid infection depends on the adaptive immune system and during the first critical hours and days of exposure to a new pathogen, our innate immune system.

References

Edit history

Date	Action	Author
2005-11-11	Created	Gillespie ME
2006-03-30	Authored	Luo F, Ouwehand WH, Gillespie ME, de Bono B
2006-04-19	Reviewed	Zwaginga JJ, D'Eustachio P, Gay NJ, Gale M Jr
2022-03-23	Modified	Weiser JD

308 submitted entities found in this pathway, mapping to 417 Reactome entities

Input	UniProt Id	Input	UniProt Id	Input	UniProt Id
ENSG00000003400	Q92851	ENSG00000005844	P20701	ENSG00000006327	Q9NP84
ENSG00000007171	P35228	ENSG00000008516	Q9NPA2	ENSG00000008517	P24001
ENSG00000021355	P30740	ENSG00000023445	Q13489, Q13490	ENSG00000049249	Q07011
ENSG00000055332	P19525	ENSG00000057149	P29508	ENSG00000058063	Q9Y2G3
ENSG00000072401	P51668	ENSG00000072803	Q9UKB1	ENSG00000088986	P63167
ENSG00000089685	O15392	ENSG00000089692	P18627	ENSG00000090376	Q9Y616
ENSG00000090382	P61626	ENSG00000092010	Q06323	ENSG00000092929	Q70J99
ENSG00000093000	Q9UKX7	ENSG00000095585	Q8WV28	ENSG00000100280	Q10567
ENSG00000100292	P09601	ENSG00000100368	P32927	ENSG00000100453	P08311
ENSG00000100504	P06737	ENSG00000100554	Q9Y5K8	ENSG00000100567	P25788
ENSG00000100644	Q16665	ENSG00000101871	O15344	ENSG00000103187	Q14019
ENSG00000103313	O15553	ENSG00000103415	P30519	ENSG00000103490	Q9ULZ3

Input	UniProt Id	Input	UniProt Id	Input	UniProt Id
ENSG00000103522	Q9HBE5	ENSG00000103653	P41240	ENSG00000104972	Q8NHL6
ENSG00000104974	O75019	ENSG00000105246	Q14213	ENSG00000105376	Q9UMF0
ENSG00000108671	O00231	ENSG00000108688	P13500	ENSG00000108691	P13500
ENSG00000108771	Q96C10	ENSG00000109320	P19838	ENSG00000109971	P11142
ENSG00000110934	Q9UBW5	ENSG00000110944	Q9NPF7	ENSG00000111229	O15145
ENSG00000111321	P36941	ENSG00000111331	Q9Y6K5	ENSG00000111335	P29728
ENSG00000111796	Q12918	ENSG00000111816	P42685	ENSG00000112096	P04179
ENSG00000112115	Q16552	ENSG00000112116	Q96PD4	ENSG00000112146	Q9UK97
ENSG00000112195	Q5T2D2	ENSG00000112299	O95497	ENSG00000113302	P29460
ENSG00000113356	O15318	ENSG00000113368	P20700	ENSG00000113575	P67775
ENSG00000113615	O95486	ENSG00000114013	P33681, P42081	ENSG00000114738	Q16644
ENSG00000115085	P43403	ENSG00000115091	P61158	ENSG00000115232	P13612
ENSG00000115267	Q9BYX4	ENSG00000115415	P42224, P42224-1, P42224-2	ENSG00000115523	P22749
ENSG00000115607	O95256	ENSG00000116260	O00391	ENSG00000116663	Q9NRD1
ENSG00000116701	P19878	ENSG00000117971	P30926	ENSG00000117984	P07339
ENSG00000119535	Q99062	ENSG00000120217	Q9NZQ7	ENSG00000120337	Q9UNG2
ENSG00000120738	P18146	ENSG00000122257	Q7Z6E9	ENSG00000122861	P00749
ENSG00000124233	P04279	ENSG00000124256	Q9H171	ENSG00000124731	Q9NP99
ENSG00000124762	P38936	ENSG00000125657	P41273	ENSG00000125735	O43557
ENSG00000126067	P49721	ENSG00000126709	P09912	ENSG00000130303	Q10589
ENSG00000130429	O15143	ENSG00000132109	P19474	ENSG00000132388	P62253
ENSG00000133661	P35247	ENSG00000134061	Q99467	ENSG00000134070	O43187
ENSG00000134242	Q9Y2R2	ENSG00000134539	Q13241	ENSG00000134545	P26715, P26717
ENSG00000134827	P20061	ENSG00000136634	P22301	ENSG00000136688	Q9NZH8
ENSG00000136689	P18510	ENSG00000136694	Q9UHA7	ENSG00000136695	Q9UBH0
ENSG00000136696	Q9NZH7	ENSG00000137462	O60603	ENSG00000137563	Q92820
ENSG00000137752	P29466	ENSG00000137807	Q02241	ENSG00000138642	Q8IVU3
ENSG00000138646	Q9UII4	ENSG00000139572	Q9NQS5	ENSG00000140368	O43586
ENSG00000140464	P29590	ENSG00000140678	P20702	ENSG00000141543	P38919
ENSG00000142089	Q01628	ENSG00000142185	O94759	ENSG00000142224	Q9UHD0
ENSG00000142507	P28072	ENSG00000142512	A6NMB1, Q96LC7	ENSG00000142583	P22732
ENSG00000142945	Q99661	ENSG00000143106	P28066	ENSG00000143119	P19397
ENSG00000143226	P12318	ENSG00000143384	Q07820	ENSG00000143546	P05109
ENSG00000143556	P31151, Q86SG5	ENSG00000143621	Q12905	ENSG00000145113	Q99102
ENSG00000145287	Q9NZF1	ENSG00000145604	Q13309	ENSG00000145623	Q99650
ENSG00000147614	Q8N8Y2	ENSG00000147813	Q6XQN6	ENSG00000147912	Q9UK96
ENSG00000149925	P04075	ENSG00000149968	P08254	ENSG00000150244	Q8IWZ4
ENSG00000150337	P12314, Q92637	ENSG00000150637	Q15762	ENSG00000151247	P06730
ENSG00000151418	Q96LB4	ENSG00000151651	P78325	ENSG00000151694	P78536
ENSG00000152056	Q96PC3	ENSG00000152778	Q13325	ENSG00000153283	P40200
ENSG00000153395	Q8NF37	ENSG00000153563	P01732	ENSG00000154380	Q8N8S7
ENSG00000156234	P02775	ENSG00000156575	Q9Y2Y8	ENSG00000156587	O14933
ENSG00000158125	P47989	ENSG00000158485	P29016	ENSG00000158517	P14598
ENSG00000159527	Q96LB9	ENSG00000160691	P29353, P29353-2	ENSG00000160703	Q86UT6

Input	UniProt Id	Input	UniProt Id	Input	UniProt Id
ENSG00000160710	P55265	ENSG00000160791	P51681	ENSG00000160796	Q6ZNJ1
ENSG00000160883	P52790	ENSG00000162063	P41002	ENSG00000162594	Q5VWK5
ENSG00000162645	P32456	ENSG00000162654	Q96PP9	ENSG00000162739	Q96DU3
ENSG00000162747	O75015	ENSG00000162891	Q9NYY1	ENSG00000162892	Q13007
ENSG00000162924	Q04864	ENSG00000163131	P25774	ENSG00000163519	Q6PIZ9
ENSG00000163563	P41218	ENSG00000163565	Q16666	ENSG00000163568	O14862
ENSG00000163636	Q15008	ENSG00000163661	P26022	ENSG00000163734	P09341
ENSG00000163736	P02775	ENSG00000163739	P09341	ENSG00000163808	Q9NS87
ENSG00000163882	P52434	ENSG00000163993	P25815	ENSG00000164047	P49913
ENSG00000164062	P13798	ENSG00000164086	Q16829	ENSG00000164163	P61221
ENSG00000164308	Q6P179	ENSG00000164924	P63104	ENSG00000165025	P43405
ENSG00000165168	P04839	ENSG00000166226	P78371	ENSG00000166278	P06681
ENSG00000166523	Q9ULY5	ENSG00000166527	Q8WXI8	ENSG00000167207	Q9HC29
ENSG00000167553	Q9BQE3	ENSG00000167618	Q6ISS4	ENSG00000167984	Q7RTR2
ENSG00000168394	Q03518	ENSG00000168610	P40763	ENSG00000168685	P16871
ENSG00000168884	Q8NFZ5	ENSG00000168961	O00182	ENSG00000168995	Q9Y286
ENSG00000169228	Q969Q5	ENSG00000169245	P02778	ENSG00000169299	Q96G03
ENSG00000169385	P10153	ENSG00000170515	Q9UQ80	ENSG00000170581	P52630
ENSG00000170956	P40198	ENSG00000171049	P21462, P25090	ENSG00000171051	P21462, P62942
ENSG00000172575	O95267	ENSG00000172724	Q99731	ENSG00000172757	P23528
ENSG00000173692	Q99460	ENSG00000173801	P14923	ENSG00000174775	P01112
ENSG00000174842	Q92990	ENSG00000175354	P17706, P17706-2	ENSG00000175463	Q8IV04
ENSG00000176014	Q9BUF5	ENSG00000176797	P81534	ENSG00000177105	P84095
ENSG00000177243	P81534	ENSG00000177257	O15263	ENSG00000177556	O00244
ENSG00000177889	P61088	ENSG00000178372	Q9NZT1	ENSG00000178562	P10747
ENSG00000181449	P48431	ENSG00000181467	P61225	ENSG00000181634	O95150
ENSG00000182481	P52292	ENSG00000182566	Q6UXB4	ENSG00000183019	Q8IX19
ENSG00000183336	Q9H3K6	ENSG00000183347	Q6ZN66	ENSG00000183486	P20592
ENSG00000184979	Q3LFD5, Q9UMW8	ENSG00000185745	P09914	ENSG00000185880	Q86WT6
ENSG00000185885	P13164	ENSG00000186074	Q8TDQ1	ENSG00000186191	P59827
ENSG00000186197	Q8WWZ3	ENSG00000186265	Q7Z6A9	ENSG00000186407	Q496F6
ENSG00000186431	P24071	ENSG00000186818	Q8NHJ6	ENSG00000186827	P43489
ENSG00000187116	A6NI73	ENSG00000188389	Q15116	ENSG00000188404	P14151
ENSG00000189013	Q99706	ENSG00000196743	P17900	ENSG00000196776	Q08722
ENSG00000196954	P49662	ENSG00000197249	P01009	ENSG00000198019	Q92637
ENSG00000198286	Q9BXL7	ENSG00000198805	P00491	ENSG00000198821	P20963-1
ENSG00000198851	P07766	ENSG00000203747	P08637	ENSG00000205420	P04264
ENSG00000205755	Q9HC73	ENSG00000205809	P26717	ENSG00000205846	Q6EIG7, Q8WTT0
ENSG00000205882	Q4QY38	ENSG00000213512	Q8N8V2	ENSG00000213658	O43561, O43561-2
ENSG00000214643	Q30KQ1	ENSG00000226650	O95239, Q2VIQ3	ENSG00000227507	Q06643

Input	UniProt Id	Input	UniProt Id	Input	UniProt Id
ENSG00000228278	P19652, Q8N138	ENSG00000233816	P01562	ENSG00000234745	P01889, P03989, P10319, P18463, P18464, P18465, P30460, P30461, P30462, P30464, P30466, P30475, P30479, P30480, P30481, P30483, P30484, P30485, P30486, P30487, P30488, P30490, P30491, P30492, P30493, P30495, P30498, P30685, Q04826, Q29718, Q29836, Q29940, Q31610, Q31612, Q95365
ENSG00000237541	P01906	ENSG00000239732	Q9NR96	ENSG00000239839	P59666
ENSG00000242550	P48595	ENSG00000243509	O95407	ENSG00000243649	P00751
ENSG00000244482	Q6PI73	ENSG00000254087	P07948	ENSG00000254415	Q08ET2, Q96PQ1
ENSG00000254521	Q96PQ1	ENSG00000257017	P00738	ENSG00000258947	Q13509
ENSG00000261456	Q3ZCM7	ENSG00000271503	P13501	ENSG00000277632	P10147, P16619
ENSG00000277775	P68431	ENSG00000278030	P04435		

Input	Ensembl Id	Input	Ensembl Id	Input	Ensembl Id
ENSG00000007171	ENSG00000007171	ENSG00000089685	ENSG00000089685	ENSG00000100292	ENSG00000100292
ENSG00000100644	ENSG00000100644	ENSG00000101871	ENSG00000101871	ENSG00000105246	ENSG00000105246
ENSG00000108691	ENSG00000108691	ENSG00000109971	ENSG00000109971	ENSG00000111331	ENSG00000111331
ENSG00000111335	ENSG00000111335	ENSG00000112096	ENSG00000112096	ENSG00000112115	ENSG00000112115
ENSG00000112116	ENSG00000112116	ENSG00000113302	ENSG00000113302	ENSG00000113368	ENSG00000113368
ENSG00000114013	ENSG00000114013	ENSG00000115415	ENSG00000115415	ENSG00000120738	ENSG00000120738
ENSG00000124762	ENSG00000124762	ENSG00000126709	ENSG00000126709	ENSG00000130303	ENSG00000130303
ENSG00000132109	ENSG00000132109	ENSG00000136634	ENSG00000136634	ENSG00000136689	ENSG00000136689
ENSG00000140464	ENSG00000140464	ENSG00000140678	ENSG00000140678	ENSG00000142089	ENSG00000142089
ENSG00000143384	ENSG00000143384	ENSG00000149968	ENSG00000149968	ENSG00000150244	ENSG00000150244
ENSG00000150337	ENSG00000150337	ENSG00000152778	ENSG00000152778	ENSG00000160710	ENSG00000160710
ENSG00000160791	ENSG00000160791	ENSG00000162594	ENSG00000162594	ENSG00000162645	ENSG00000162645
ENSG00000162654	ENSG00000162654	ENSG00000163739	ENSG00000163739	ENSG00000169245	ENSG00000169245
ENSG00000171051	ENSG00000171051	ENSG00000172724	ENSG00000172724	ENSG00000172757	ENSG00000172757
ENSG00000175354	ENSG00000175354	ENSG00000181449	ENSG00000181449	ENSG00000183336	ENSG00000183336
ENSG00000183347	ENSG00000183347	ENSG00000183486	ENSG00000183486	ENSG00000185745	ENSG00000185745
ENSG00000185885	ENSG00000185885	ENSG00000198019	ENSG00000198019	ENSG00000213512	ENSG00000213512
ENSG00000233816	ENSG00000233816	ENSG00000234745	ENSG00000234745	ENSG00000237541	ENSG00000237541
ENSG00000271503	ENSG00000271503	ENSG00000277632	ENSG00000277632		

6. Cytokine Signaling in Immune system (R-HSA-1280215)

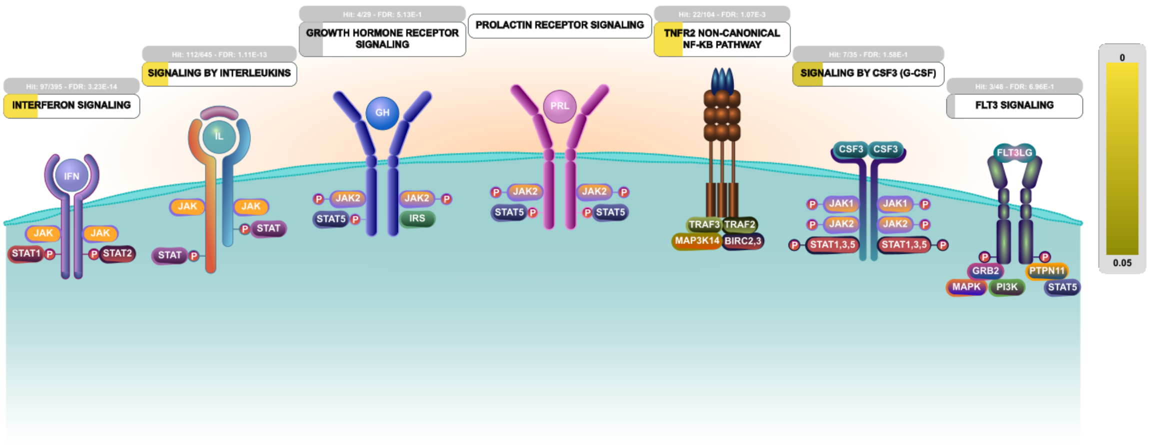

Cytokines are small proteins that regulate and mediate immunity, inflammation, and hematopoiesis. They are secreted in response to immune stimuli, and usually act briefly, locally, at very low concentrations. Cytokines bind to specific membrane receptors, which then signal the cell via second messengers, to regulate cellular activity.

References

Feldmann M & Oppenheim J (2002). *Cytokines and the immune system, Cytokine Reference* .

IMMPORT:Bioinformatics for the future of immunology. Retrieved from https://www.immport.org/immportWeb/queryref/geneListSummary.do

Santamaria P (2003). Cytokines and chemokines in autoimmune disease: an overview. Adv Exp Med Biol, 520, 1-7.

COPE. Retrieved from http://www.copewithcytokines.org/cope.cgi

Edit history

Date	Action	Author
2011-05-12	Created	Garapati P V
2011-05-22	Edited	Ray KP, Jupe S, Garapati P V
2011-05-22	Authored	Ray KP, Jupe S, Garapati P V
2011-05-28	Reviewed	Abdul-Sater AA, Schindler C, Pinteaux E
2022-03-23	Modified	Weiser JD

129 submitted entities found in this pathway, mapping to 226 Reactome entities

Input	UniProt Id	Input	UniProt Id	Input	UniProt Id
ENSG00000006327	Q9NP84	ENSG00000007171	P35228	ENSG00000008517	P24001

Input	UniProt Id	Input	UniProt Id	Input	UniProt Id
ENSG00000023445	Q13489, Q13490	ENSG00000049249	Q07011	ENSG00000055332	P19525
ENSG00000072401	P51668	ENSG00000072803	Q9UKB1	ENSG00000089685	O15392
ENSG00000090376	Q9Y616	ENSG00000092010	Q06323	ENSG00000093000	Q9UKX7
ENSG00000095585	Q8WV28	ENSG00000100292	P09601	ENSG00000100368	P32927
ENSG00000100453	P08311	ENSG00000100567	P25788	ENSG00000100644	Q16665
ENSG00000101871	O15344	ENSG00000103522	Q9HBE5	ENSG00000103653	P41240
ENSG00000105246	Q14213	ENSG00000108671	O00231	ENSG00000108688	P13500
ENSG00000108691	P13500	ENSG00000109320	P19838	ENSG00000109971	P11142
ENSG00000110944	Q9NPF7	ENSG00000111321	P36941	ENSG00000111331	Q9Y6K5
ENSG00000111335	P29728	ENSG00000112096	P04179	ENSG00000112115	Q16552
ENSG00000112116	Q96PD4	ENSG00000113302	P29460	ENSG00000113368	P20700
ENSG00000113575	P67775	ENSG00000114013	P33681, P42081	ENSG00000114738	Q16644
ENSG00000115415	P42224, P42224-1, P42224-2	ENSG00000115607	O95256	ENSG00000119535	Q99062
ENSG00000120337	Q9UNG2	ENSG00000120738	P18146	ENSG00000124762	P38936
ENSG00000125657	P41273	ENSG00000125735	O43557	ENSG00000126067	P49721
ENSG00000126709	P09912	ENSG00000130303	Q10589	ENSG00000132109	P19474
ENSG00000134070	O43187	ENSG00000136634	P22301	ENSG00000136688	Q9NZH8
ENSG00000136689	P18510	ENSG00000136694	Q9UHA7	ENSG00000136695	Q9UBH0
ENSG00000136696	Q9NZH7	ENSG00000137752	P29466	ENSG00000138646	Q9UII4
ENSG00000140464	P29590	ENSG00000140678	P20702	ENSG00000141543	P38919
ENSG00000142089	Q01628	ENSG00000142224	Q9UHD0	ENSG00000142507	P28072
ENSG00000143106	P28066	ENSG00000143384	Q07820	ENSG00000145623	Q99650
ENSG00000149968	P08254	ENSG00000150244	Q8IWZ4	ENSG00000150337	P12314, Q92637
ENSG00000151247	P06730	ENSG00000151694	P78536	ENSG00000152778	Q13325
ENSG00000156587	O14933	ENSG00000160691	P29353	ENSG00000160710	P55265
ENSG00000160791	P51681	ENSG00000162594	Q5VWK5	ENSG00000162645	P32456
ENSG00000162654	Q96PP9	ENSG00000162891	Q9NYY1	ENSG00000162892	Q13007
ENSG00000163636	Q15008	ENSG00000163734	P09341	ENSG00000163739	P09341
ENSG00000164086	Q16829	ENSG00000164163	P61221	ENSG00000164924	P63104
ENSG00000165025	P43405	ENSG00000167207	Q9HC29	ENSG00000168610	P40763
ENSG00000168685	P16871	ENSG00000168884	Q8NFZ5	ENSG00000168961	O00182
ENSG00000169245	P02778	ENSG00000170581	P52630	ENSG00000171049	P21462
ENSG00000171051	P21462	ENSG00000172724	Q99731	ENSG00000172757	P23528
ENSG00000173692	Q99460	ENSG00000174775	P01112	ENSG00000175354	P17706, P17706-2
ENSG00000177889	P61088	ENSG00000181449	P48431	ENSG00000181634	O95150
ENSG00000182481	P52292	ENSG00000183336	Q9H3K6	ENSG00000183347	Q6ZN66
ENSG00000183486	P20592	ENSG00000184979	Q3LFD5, Q9UMW8	ENSG00000185745	P09914
ENSG00000185885	P13164	ENSG00000186197	Q8WWZ3	ENSG00000186827	P43489
ENSG00000198019	Q92637	ENSG00000205755	Q9HC73	ENSG00000213512	Q8N8V2

Input	UniProt Id	Input	UniProt Id	Input	UniProt Id
ENSG00000227507	Q06643	ENSG00000233816	P01562	ENSG00000234745	P01889, P03989, P10319, P18463, P18464, P18465, P30460, P30461, P30462, P30464, P30466, P30475, P30479, P30480, P30481, P30483, P30484, P30485, P30486, P30487, P30488, P30490, P30491, P30492, P30493, P30495, P30498, P30685, Q04826, Q29718, Q29836, Q29940, Q31610, Q31612, Q95365
ENSG00000237541	P01906	ENSG00000243509	O95407	ENSG00000254087	P07948
ENSG00000271503	P13501	ENSG00000277632	P10147, P16619	ENSG00000277775	P68431

Input	Ensembl Id	Input	Ensembl Id	Input	Ensembl Id
ENSG00000007171	ENSG00000007171	ENSG00000089685	ENSG00000089685	ENSG00000100292	ENSG00000100292
ENSG00000100644	ENSG00000100644	ENSG00000101871	ENSG00000101871	ENSG00000105246	ENSG00000105246
ENSG00000108691	ENSG00000108691	ENSG00000109971	ENSG00000109971	ENSG00000111331	ENSG00000111331
ENSG00000111335	ENSG00000111335	ENSG00000112096	ENSG00000112096	ENSG00000112115	ENSG00000112115
ENSG00000112116	ENSG00000112116	ENSG00000113302	ENSG00000113302	ENSG00000113368	ENSG00000113368
ENSG00000114013	ENSG00000114013	ENSG00000115415	ENSG00000115415	ENSG00000120738	ENSG00000120738
ENSG00000124762	ENSG00000124762	ENSG00000126709	ENSG00000126709	ENSG00000130303	ENSG00000130303
ENSG00000132109	ENSG00000132109	ENSG00000136634	ENSG00000136634	ENSG00000136689	ENSG00000136689
ENSG00000140464	ENSG00000140464	ENSG00000140678	ENSG00000140678	ENSG00000142089	ENSG00000142089
ENSG00000143384	ENSG00000143384	ENSG00000149968	ENSG00000149968	ENSG00000150244	ENSG00000150244
ENSG00000150337	ENSG00000150337	ENSG00000152778	ENSG00000152778	ENSG00000160710	ENSG00000160710
ENSG00000160791	ENSG00000160791	ENSG00000162594	ENSG00000162594	ENSG00000162645	ENSG00000162645
ENSG00000162654	ENSG00000162654	ENSG00000163739	ENSG00000163739	ENSG00000169245	ENSG00000169245
ENSG00000171051	ENSG00000171051	ENSG00000172724	ENSG00000172724	ENSG00000172757	ENSG00000172757
ENSG00000175354	ENSG00000175354	ENSG00000181449	ENSG00000181449	ENSG00000183336	ENSG00000183336
ENSG00000183347	ENSG00000183347	ENSG00000183486	ENSG00000183486	ENSG00000185745	ENSG00000185745
ENSG00000185885	ENSG00000185885	ENSG00000198019	ENSG00000198019	ENSG00000213512	ENSG00000213512
ENSG00000234745	ENSG00000234745	ENSG00000237541	ENSG00000237541	ENSG00000271503	ENSG00000271503
ENSG00000277632	ENSG00000277632				

7. Signaling by Interleukins (R-HSA-449147)

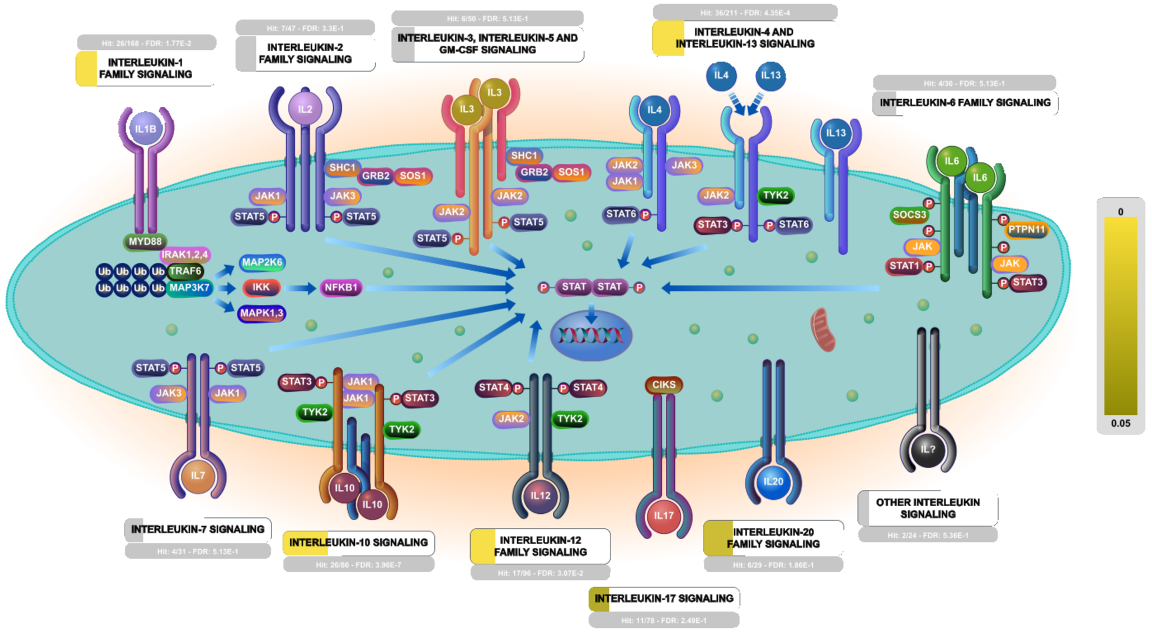

Cellular compartments: plasma membrane.

Interleukins are low molecular weight proteins that bind to cell surface receptors and act in an autocrine and/or paracrine fashion. They were first identified as factors produced by leukocytes but are now known to be produced by many other cells throughout the body. They have pleiotropic effects on cells which bind them, impacting processes such as tissue growth and repair, hematopoietic homeostasis, and multiple levels of the host defense against pathogens where they are an essential part of the immune system.

References

Dinarello CA (2009). Immunological and inflammatory functions of the interleukin-1 family. Annu Rev Immunol, 27, 519-50.

Komlosi Z, Kucuksezer UC, Frei R, Huitema C, Garbani M, Pezer M, ... Eiwegger T (2016). Interleukins (from IL-1 to IL-38), interferons, transforming growth factor , and TNF-: Receptors, functions, and roles in diseases. J. Allergy Clin. Immunol., 138, 984-1010.

Vosshenrich CA & Di Santo JP (2002). Interleukin signaling. Curr Biol, 12, R760-3.

Edit history

Date	Action	Author
2009-11-27	Created	Jupe S
2010-05-17	Reviewed	Pinteaux E
2010-05-17	Authored	Ray KP
2010-05-26	Edited	Jupe S
2022-03-23	Modified	Weiser JD

80 submitted entities found in this pathway, mapping to 115 Reactome entities

Input	UniProt Id	Input	UniProt Id	Input	UniProt Id
ENSG00000007171	P35228	ENSG00000008517	P24001	ENSG00000072803	Q9UKB1
ENSG00000089685	O15392	ENSG00000090376	Q9Y616	ENSG00000092010	Q06323
ENSG00000095585	Q8WV28	ENSG00000100292	P09601	ENSG00000100368	P32927
ENSG00000100453	P08311	ENSG00000100567	P25788	ENSG00000100644	Q16665
ENSG00000103522	Q9HBE5	ENSG00000105246	Q14213	ENSG00000108671	O00231
ENSG00000108688	P13500	ENSG00000108691	P13500	ENSG00000109320	P19838
ENSG00000109971	P11142	ENSG00000110944	Q9NPF7	ENSG00000112096	P04179
ENSG00000112115	Q16552	ENSG00000112116	Q96PD4	ENSG00000113302	P29460
ENSG00000113368	P20700	ENSG00000113575	P67775	ENSG00000114013	P33681, P42081
ENSG00000114738	Q16644	ENSG00000115415	P42224, P42224-1	ENSG00000115607	O95256
ENSG00000119535	Q99062	ENSG00000124762	P38936	ENSG00000126067	P49721
ENSG00000134070	O43187	ENSG00000136634	P22301	ENSG00000136688	Q9NZH8
ENSG00000136689	P18510	ENSG00000136694	Q9UHA7	ENSG00000136695	Q9UBH0
ENSG00000136696	Q9NZH7	ENSG00000137752	P29466	ENSG00000140678	P20702
ENSG00000142224	Q9UHD0	ENSG00000142507	P28072	ENSG00000143106	P28066
ENSG00000143384	Q07820	ENSG00000145623	Q99650	ENSG00000149968	P08254
ENSG00000160691	P29353	ENSG00000160791	P51681	ENSG00000162594	Q5VWK5
ENSG00000162891	Q9NYY1	ENSG00000162892	Q13007	ENSG00000163636	Q15008
ENSG00000163734	P09341	ENSG00000163739	P09341	ENSG00000164086	Q16829
ENSG00000164924	P63104	ENSG00000165025	P43405	ENSG00000167207	Q9HC29
ENSG00000168610	P40763	ENSG00000168685	P16871	ENSG00000168884	Q8NFZ5
ENSG00000168961	O00182	ENSG00000169245	P02778	ENSG00000170581	P52630
ENSG00000171049	P21462	ENSG00000171051	P21462	ENSG00000172724	Q99731
ENSG00000172757	P23528	ENSG00000173692	Q99460	ENSG00000175354	P17706
ENSG00000177889	P61088	ENSG00000181449	P48431	ENSG00000183336	Q9H3K6
ENSG00000205755	Q9HC73	ENSG00000254087	P07948	ENSG00000271503	P13501
ENSG00000277632	P10147, P16619	ENSG00000277775	P68431		

Input	Ensembl Id	Input	Ensembl Id	Input	Ensembl Id
ENSG00000007171	ENSG00000007171	ENSG00000089685	ENSG00000089685	ENSG00000100292	ENSG00000100292
ENSG00000100644	ENSG00000100644	ENSG00000105246	ENSG00000105246	ENSG00000108691	ENSG00000108691
ENSG00000109971	ENSG00000109971	ENSG00000112096	ENSG00000112096	ENSG00000112115	ENSG00000112115
ENSG00000112116	ENSG00000112116	ENSG00000113302	ENSG00000113302	ENSG00000113368	ENSG00000113368
ENSG00000114013	ENSG00000114013	ENSG00000115415	ENSG00000115415	ENSG00000124762	ENSG00000124762
ENSG00000136634	ENSG00000136634	ENSG00000136689	ENSG00000136689	ENSG00000140678	ENSG00000140678
ENSG00000143384	ENSG00000143384	ENSG00000149968	ENSG00000149968	ENSG00000160791	ENSG00000160791
ENSG00000162594	ENSG00000162594	ENSG00000163739	ENSG00000163739	ENSG00000169245	ENSG00000169245
ENSG00000171051	ENSG00000171051	ENSG00000172724	ENSG00000172724	ENSG00000172757	ENSG00000172757
ENSG00000175354	ENSG00000175354	ENSG00000181449	ENSG00000181449	ENSG00000183336	ENSG00000183336
ENSG00000271503	ENSG00000271503	ENSG00000277632	ENSG00000277632		

8. Immunoregulatory interactions between a Lymphoid and a non-Lymphoid cell (R-HSA-198933)

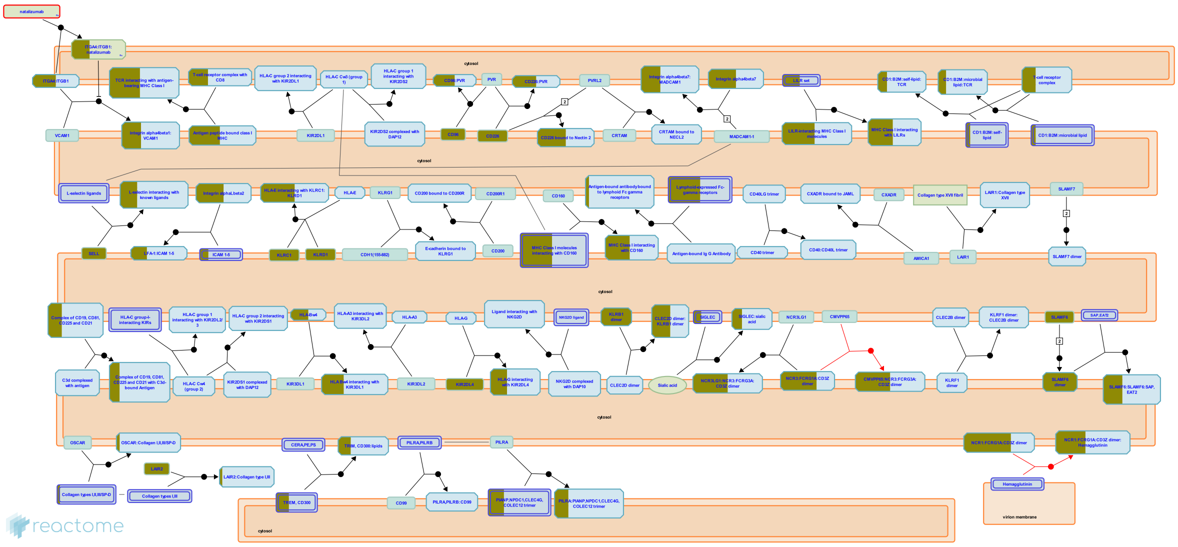

A number of receptors and cell adhesion molecules play a key role in modifying the response of cells of lymphoid origin (such as B-, T- and NK cells) to self and tumor antigens, as well as to pathogenic organisms.

Molecules such as KIRs and LILRs form part of a crucial surveillance system that looks out for any derangement, usually caused by cancer or viral infection, in MHC Class I presentation. Somatic cells are also able to report internal functional impairment by displaying surface stress markers such as MICA. The presence of these molecules on somatic cells is picked up by C-lectin NK immune receptors.

Lymphoid cells are able to regulate their location and movement in accordance to their state of activation, and home in on tissues expressing the appropriate complementary ligands. For example, lymphoid cells may fine tune the presence and concentration of adhesion molecules belonging to the IgSF, Selectin and Integrin class that interact with a number of vascular markers of inflammation.

Furthermore, there are a number of avenues through which lymphoid cells may interact with antigen. This may be presented directly to a specific T-cell receptor in the context of an MHC molecule. Antigen-antibody complexes may anchor to the cell via a small number of lymphoid-specific Fc receptors that may, in turn, influence cell function further. Activated complement factor C3d binds to both antigen and to cell surface receptor CD21. In such cases, the far-reaching influence of CD19 on B-lymphocyte function is tempered by its interaction with CD21.

References

Kelley J, Trowsdale J & Walter L (2005). Comparative genomics of natural killer cell receptor gene clusters. PLoS Genet., 1, 129-39.

Tomasello E, Walzer T, Vivier E, Baratin M & Ugolini S (2008). Functions of natural killer cells. Nat. Immunol., 9, 503-10.

Vivier E, Harris J, Trowsdale J, Vely F, Nedvetzki S, Davis DM, ... Pende D (2007). Reciprocal regulation of human natural killer cells and macrophages associated with distinct immune synapses. Blood, 109, 3776-85.

Batista FD & Carrasco YR (2006). B cell recognition of membrane-bound antigen: an exquisite way of sensing ligands. Curr Opin Immunol, 18, 286-91.

Bromley SK, Shaw AS, Somersalo K, Allen PM, Sims TN, Dustin ML, ... Sumen C (2001). The immunological synapse. Annu Rev Immunol, 19, 375-96.

Edit history

Date	Action	Author
2007-07-08	Authored	de Bono B
2007-07-08	Created	de Bono B
2007-08-06	Reviewed	Trowsdale J
2015-03-27	Authored	Garapati P V
2015-05-12	Reviewed	Barrow AD
2022-03-23	Modified	Weiser JD

36 submitted entities found in this pathway, mapping to 70 Reactome entities

Input	UniProt Id	Input	UniProt Id	Input	UniProt Id
ENSG00000005844	P20701	ENSG00000104972	Q8NHL6	ENSG00000104974	O75019
ENSG00000105376	Q9UMF0	ENSG00000111796	Q12918	ENSG00000112195	Q5T2D2
ENSG00000115232	P13612	ENSG00000124731	Q9NP99	ENSG00000133661	P35247
ENSG00000134539	Q13241	ENSG00000134545	P26715	ENSG00000142512	Q96LC7
ENSG00000150337	P12314	ENSG00000150637	Q15762	ENSG00000153283	P40200
ENSG00000153563	P01732	ENSG00000158485	P29016	ENSG00000162739	Q96DU3
ENSG00000167618	Q6ISS4	ENSG00000168995	Q9Y286	ENSG00000182566	Q6UXB4
ENSG00000185885	P13164	ENSG00000186074	Q8TDQ1	ENSG00000186407	Q496F6
ENSG00000186818	Q8NHJ6	ENSG00000187116	A6NI73	ENSG00000188404	P14151
ENSG00000189013	Q99706	ENSG00000198821	P20963-1	ENSG00000198851	P07766
ENSG00000203747	P08637	ENSG00000234745	P01889, P03989, P10319, P18463, P18464, P18465, P30460, P30461, P30462, P30464, P30466, P30475, P30479, P30480, P30481, P30483, P30484, P30485, P30486, P30487, P30488, P30490, P30491, P30492, P30493, P30495, P30498, P30685, Q04826, Q29718, Q29836, Q29940, Q31610, Q31612, Q95365	ENSG00000244482	Q6PI73
ENSG00000254415	Q96PQ1	ENSG00000254521	Q96PQ1	ENSG00000278030	P04435

9. Antigen processing-Cross presentation (R-HSA-1236975)

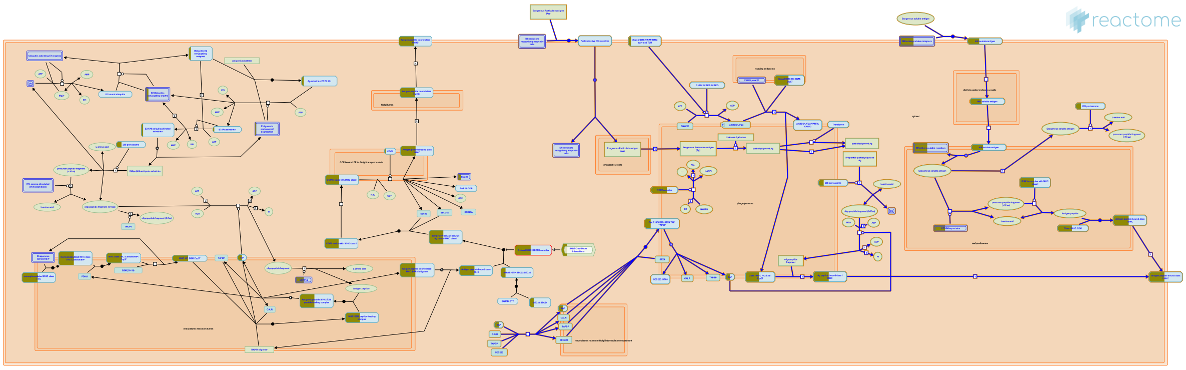

MHC class I molecules generally present peptide antigens derived from proteins synthesized by the cell itself to CD8+ T cells. However, in some circumstances, antigens from extracellular environment can be presented on MHC class I to stimulate CD8+ T cell immunity, a process termed cross-presentation (Rock & Shen. 2005). Cross-presentation/cross-priming is the ability of antigen presenting cells (APCs) to present exogenous antigens on MHC class I molecules to CD8+ T lymphocytes. Among all the APCs, Dendritic cells (DC) are the dominant antigen cross presenting cell types in vivo, although macrophages and B cells appear to cross present model antigens in vitro with a low degree of efficiency (Amigorena & Savina. 2010, Ackermann & Peter Cresswell. 2004). Compared to macrophages, DCs have low levels of lysosomal proteases and exhibit limited lysosomal degradation (Delamarre et al. 2005). This limited proteolysis of internalized antigens by DCs might contribute to their high efficiency for cross presentation (Monua & Trombetta. 2007). APCs acquire the exogenous antigens through endocytic mechanisms, especially phagosomes for particulate/cell-associated antigens and endosomes for soluble protein antigens. There does not seem to be a unique pathway for cross-presentation but rather different potential mechanisms of cross-presentation have been proposed. These proposed pathways can be classified according to the location where two key events occur: 1) processing of the antigenic protein and 2) loading of the processed peptide on to MHC I molecule (Blanchard & Shastri. 2010). Based on the requirement for TAP and cytosolic proteases two mechanisms have been described, a cytosolic pathway (TAP-dependent and proteasome-dependent) or a vacuolar pathway (TAP- and proteasome-independent) (Blanchard & Shastri. 2010, Amigorena & Savina. 2010). Regarding peptide-loading, MHC I could be loaded in the ER or in the phagosome and recycled to cell surface (Blanchard & Shastri. 2010). Exogenous soluble antigens are cross-presented by dendritic cells, albeit with lower efficiency than for particulate substrates. Soluble antigens destined for cross-presentation are taken up by distinct endocytosis mechanisms which route them into stable early endosomes and then to the cytoplasm for proteasomal degradation and peptide loading. The outcome of the cross presentation can be either tolerance or immunity (Rock & Shen. 2005).

References

Rock KL & Shen L (2005). Cross-presentation: underlying mechanisms and role in immune surveillance. Immunol Rev, 207, 166-83.

Savina A & Amigorena S (2010). Intracellular mechanisms of antigen cross presentation in dendritic cells. Curr Opin Immunol, 22, 109-17.

Trombetta ES & Monu N (2007). Cross-talk between the endocytic pathway and the endoplasmic reticulum in cross-presentation by MHC class I molecules. Curr Opin Immunol, 19, 66-72.

Rahner C, Giodini A & Cresswell P (2009). Receptor-mediated phagocytosis elicits cross-presentation in nonprofessional antigen-presenting cells. Proc Natl Acad Sci U S A, 106, 3324-9.

Trombetta ES, Pack M, Mellman I, Chang H & Delamarre L (2005). Differential lysosomal proteolysis in antigen-presenting cells determines antigen fate. Science, 307, 1630-4.

Edit history

Date	Action	Author
2011-03-28	Edited	Garapati P V
2011-03-28	Authored	Garapati P V
2011-03-28	Created	Garapati P V
2011-05-13	Reviewed	Desjardins M, English L
2022-03-23	Modified	Weiser JD

18 submitted entities found in this pathway, mapping to 53 Reactome entities

Input	UniProt Id	Input	UniProt Id	Input	UniProt Id
ENSG00000092010	Q06323	ENSG00000100567	P25788	ENSG00000108671	O00231
ENSG00000116701	P19878	ENSG00000126067	P49721	ENSG00000137462	O60603
ENSG00000142507	P28072	ENSG00000143106	P28066	ENSG00000143546	P05109
ENSG00000150337	P12314, Q92637	ENSG00000158517	P14598	ENSG00000163131	P25774
ENSG00000163636	Q15008	ENSG00000165168	P04839	ENSG00000168394	Q03518
ENSG00000173692	Q99460	ENSG00000198019	Q92637	ENSG00000234745	P01889, P03989, P10319, P18463, P18464, P18465, P30460, P30461, P30462, P30464, P30466, P30475, P30479, P30480, P30481, P30483, P30484, P30485, P30486, P30487, P30488, P30490, P30491, P30492, P30493, P30495, P30498, P30685, Q04826, Q29718, Q29836, Q29940, Q31610, Q31612, Q95365

10. Antigen Presentation: Folding, assembly and peptide loading of class I MHC (R-HSA-983170)

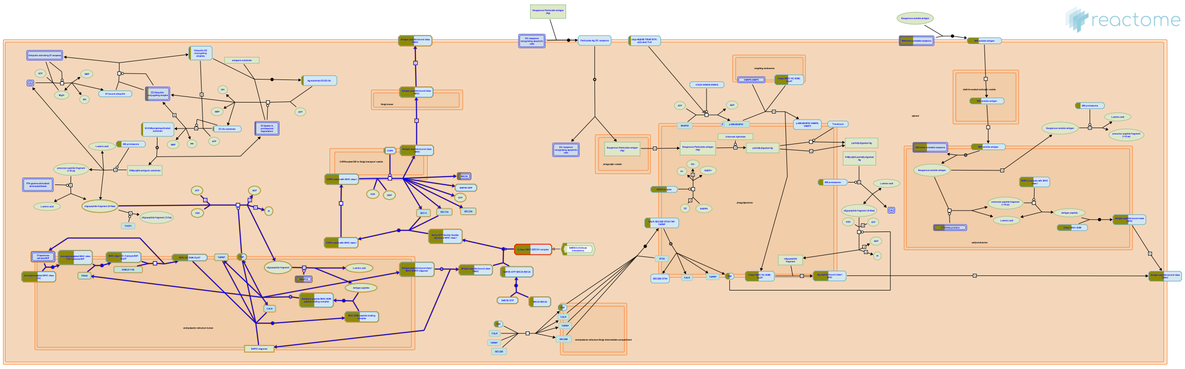

Unlike other glycoproteins, correct folding of MHC class I molecules is not sufficient to trigger their exit from the ER, they exit only after peptide loading. Described here is the process of antigen presentation which consists of the folding, assembly, and peptide loading of MHC class I molecules. The newly synthesized MHC class I Heavy Chain (HC) is initially folded with the help of several chaperones (calnexin, BiP, ERp57) and then binds with Beta-2-microglobulin (B2M). This MHC:B2M heterodimer enters the peptide loading complex (PLC), a multiprotein complex that includes calreticulin, endoplasmic reticulum resident protein 57 (ERp57), transporter associated with antigen processing (TAP) and tapasin. Peptides generated from Ub-proteolysis are transported into the ER through TAP. These peptides are further trimmed by ER-associated aminopeptidase (ERAP) and loaded on to MHC class I molecules. Stable MHC class I trimers with high-affinity peptide are transported from the ER to the cell surface by the Golgi apparatus.

References

Cresswell P & Wearsch PA (2008). The quality control of MHC class I peptide loading. Curr Opin Cell Biol, 20, 624-31.

Ploegh HL, Van der Veen AG & Vyas JM (2008). The known unknowns of antigen processing and presentation. Nat Rev Immunol, 8, 607-18.

Jeong E, Kim Y, Ahn K, Lee YJ, Kim I, Kang K, ... Oh C (2009). Molecular mechanisms of MHC class I-antigen processing: redox considerations. Antioxid Redox Signal, 11, 907-36.

Powis SJ, Elliott T & Antoniou AN (2003). Assembly and export of MHC class I peptide ligands. Curr Opin Immunol, 15, 75-81.

Rock KL, Goldberg AL & York IA (2004). Post-proteasomal antigen processing for major histocompatibility complex class I presentation. Nat Immunol, 5, 670-7.

Edit history

Date	Action	Author
2010-10-29	Edited	Garapati P V
2010-10-29	Authored	Garapati P V
2010-10-29	Created	Garapati P V
2011-02-10	Reviewed	Elliott T
2022-03-23	Modified	Weiser JD

4 submitted entities found in this pathway, mapping to 38 Reactome entities

Input	UniProt Id
ENSG00000113615	O95486
ENSG00000168394	Q03518

Input	UniProt Id
ENSG00000164308	Q6P179
ENSG00000234745	P01889, P03989, P10319, P18463, P18464, P18465, P30460, P30461, P30462, P30464, P30466, P30475, P30479, P30480, P30481, P30483, P30484, P30485, P30486, P30487, P30488, P30490, P30491, P30492, P30493, P30495, P30498, P30685, Q04826, Q29718, Q29836, Q29940, Q31610, Q31612, Q95365

11. Neutrophil degranulation (R-HSA-6798695)

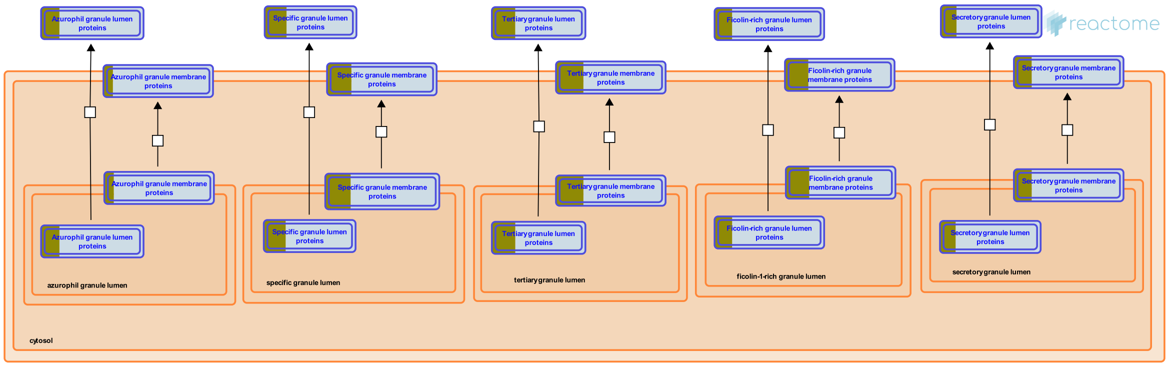

Neutrophils are the most abundant leukocytes (white blood cells), indispensable in defending the body against invading microorganisms. In response to infection, neutrophils leave the circulation and migrate towards the inflammatory focus. They contain several subsets of granules that are mobilized to fuse with the cell membrane or phagosomal membrane, resulting in the exocytosis or exposure of membrane proteins. Traditionally, neutrophil granule constituents are described as antimicrobial or proteolytic, but granules also introduce membrane proteins to the cell surface, changing how the neutrophil responds to its environment (Borregaard et al. 2007). Primed neutrophils actively secrete cytokines and other inflammatory mediators and can present antigens via MHC II, stimulating T-cells (Wright et al. 2010).

Granules form during neutrophil differentiation. Granule subtypes can be distinguished by their content but overlap in structure and composition. The differences are believed to be a consequence of changing protein expression and differential timing of granule formation during the terminal processes of neutrophil differentiation, rather than sorting (Le Cabec et al. 1996).

The classical granule subsets are Azurophil or primary granules (AG), secondary granules (SG) and gelatinase granules (GG). Neutrophils also contain exocytosable storage cell organelles, storage vesicles (SV), formed by endocytosis they contain many cell-surface markers and extracellular, plasma proteins (Borregaard et al. 1992). Ficolin-1-rich granules (FG) are like GGs highly exocytosable but gelatinase-poor (Rorvig et al. 2009).

References

Heegaard NH, Rørvig S, Borregaard N & Østergaard O (2013). Proteome profiling of human neutrophil granule subsets, secretory vesicles, and cell membrane: correlation with transcriptome profiling of neutrophil precursors. J. Leukoc. Biol., 94, 711-21.

Sørensen OE, Borregaard N & Theilgaard-Mönch K (2007). Neutrophil granules: a library of innate immunity proteins. Trends Immunol., 28, 340-5.

Nielsen MH, Johnsen AH, Bjerrum OW, Borregaard N, Kjeldsen L, Rygaard K, ... Bastholm L (1992). Stimulus-dependent secretion of plasma proteins from human neutrophils. J. Clin. Invest., 90, 86-96.

Bucknall RC, Wright HL, Edwards SW & Moots RJ (2010). Neutrophil function in inflammation and inflammatory diseases. Rheumatology (Oxford), 49, 1618-31.

Le Cabec V, Borregaard N, Calafat J & Cowland JB (1996). Targeting of proteins to granule subsets is determined by timing and not by sorting: The specific granule protein NGAL is localized to azurophil granules when expressed in HL-60 cells. Proc. Natl. Acad. Sci. U.S.A., 93, 6454-7.

Edit history

Date	Action	Author
2015-09-21	Authored	Jupe S
2015-09-21	Created	Jupe S
2016-06-13	Edited	Jupe S
2016-06-13	Reviewed	Heegaard N
2022-03-23	Modified	Weiser JD

88 submitted entities found in this pathway, mapping to 90 Reactome entities

Input	UniProt Id	Input	UniProt Id	Input	UniProt Id
ENSG00000005844	P20701	ENSG00000008516	Q9NPA2	ENSG00000021355	P30740
ENSG00000057149	P29508	ENSG00000058063	Q9Y2G3	ENSG00000088986	P63167
ENSG00000090382	P61626	ENSG00000092929	Q70J99	ENSG00000100453	P08311
ENSG00000100504	P06737	ENSG00000100554	Q9Y5K8	ENSG00000103187	Q14019
ENSG00000103415	P30519	ENSG00000103490	Q9ULZ3	ENSG00000108671	O00231
ENSG00000109320	P19838	ENSG00000109971	P11142	ENSG00000110934	Q9UBW5
ENSG00000111816	P42685	ENSG00000112299	O95497	ENSG00000116260	O00391
ENSG00000117971	P30926	ENSG00000117984	P07339	ENSG00000122861	P00749
ENSG00000130303	Q10589	ENSG00000134827	P20061	ENSG00000137462	O60603
ENSG00000137563	Q92820	ENSG00000139572	Q9NQS5	ENSG00000140678	P20702
ENSG00000142185	O94759	ENSG00000142583	P22732	ENSG00000143106	P28066
ENSG00000143119	P19397	ENSG00000143226	P12318	ENSG00000143546	P05109
ENSG00000143556	P31151	ENSG00000143621	Q12905	ENSG00000145287	Q9NZF1
ENSG00000147813	Q6XQN6	ENSG00000149925	P04075	ENSG00000151651	P78325
ENSG00000153395	Q8NF37	ENSG00000156234	P02775	ENSG00000156575	Q9Y2Y8
ENSG00000160796	Q6ZNJ1	ENSG00000160883	P52790	ENSG00000162747	O75015
ENSG00000163131	P25774	ENSG00000163563	P41218	ENSG00000163636	Q15008
ENSG00000163661	P26022	ENSG00000163734	P09341	ENSG00000163736	P02775
ENSG00000163739	P09341	ENSG00000163993	P25815	ENSG00000164047	P49913
ENSG00000164062	P13798	ENSG00000165168	P04839	ENSG00000166226	P78371
ENSG00000166527	Q8WXI8	ENSG00000169228	Q969Q5	ENSG00000169299	Q96G03
ENSG00000169385	P10153	ENSG00000170515	Q9UQ80	ENSG00000170956	P40198
ENSG00000171049	P21462, P25090	ENSG00000171051	P21462	ENSG00000173692	Q99460
ENSG00000173801	P14923	ENSG00000175463	Q8IV04	ENSG00000177105	P84095
ENSG00000178372	Q9NZT1	ENSG00000181467	P61225	ENSG00000183019	Q8IX19
ENSG00000186431	P24071	ENSG00000188404	P14151	ENSG00000196743	P17900
ENSG00000196776	Q08722	ENSG00000197249	P01009	ENSG00000198805	P00491
ENSG00000205420	P04264	ENSG00000205846	Q8WTT0	ENSG00000228278	P19652, Q8N138
ENSG00000234745	P01889	ENSG00000242550	P48595	ENSG00000254415	Q08ET2
ENSG00000257017	P00738				

12. ER-Phagosome pathway (R-HSA-1236974)

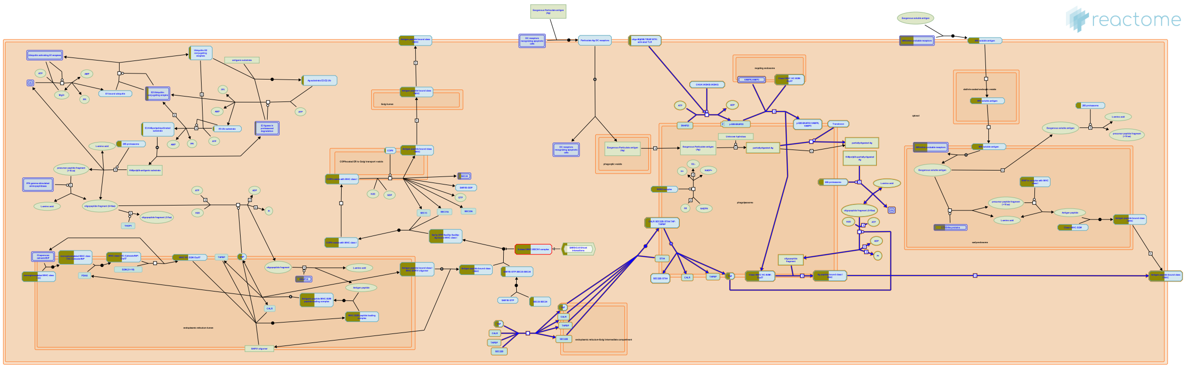

The other TAP-dependent cross-presentation mechanism in phagocytes is the endoplasmic reticulum (ER)-phagosome model. Desjardins proposed that ER is recruited to the cell surface, where it fuses with the plasma membrane, underneath phagocytic cups, to supply membrane for the formation of nascent phagosomes (Gagnon et al. 2002). Three independent studies simultaneously showed that ER contributes to the vast majority of phagosome membrane (Guermonprez et al. 2003, Houde et al. 2003, Ackerman et al. 2003). The composition of early phagosome membrane contains ER-resident proteins, the components required for cross-presentation. This model is similar to the phagosome-to-cytosol model in that Ag is translocated to cytosol for proteasomal degradation, but differs in that antigenic peptides are translocated back into the phagosome (instead of ER) for peptide:MHC-I complexes. ER fusion with phagosome introduces molecules that are involved in Ag transport to cytosol (Sec61) and proteasome-generated peptides back into the phagosome (TAP) for loading onto MHC-I.

Although the ER-phagosome pathway is controversial, the concept remains attractive as it explains how peptide-receptive MHC-I molecules could intersect with a relatively high concentration of exogenous antigens, presumably a crucial prerequisite for efficient cross-presentation (Basha et al. 2008).

References

Paroutis P, Grinstein S & Touret N (2005). The nature of the phagosomal membrane: endoplasmic reticulum versus plasmalemma. J Leukoc Biol, 77, 878-85.

Savina A & Amigorena S (2010). Intracellular mechanisms of antigen cross presentation in dendritic cells. Curr Opin Immunol, 22, 109-17.

Barreiro L, Carruthers NJ, LaBoissière S, Goyette G, Dermine JF, Desjardins M, ... Duclos S (2012). Proteomic characterization of phagosomal membrane microdomains during phagolysosome biogenesis and evolution. Mol. Cell Proteomics, 11, 1365-77.

Houde M, Brunet S, Laplante A, Goyette G, Bertholet S, Desjardins M, ... Princiotta MF (2003). Phagosomes are competent organelles for antigen cross-presentation. Nature, 425, 402-6.

Bergeron JJ, Desjardins M & Gagnon E (2005). ER-mediated phagocytosis: myth or reality?. J Leukoc Biol, 77, 843-5.

Edit history

Date	Action	Author
2011-03-28	Edited	Garapati P V

Date	Action	Author
2011-03-28	Authored	Garapati P V
2011-03-28	Created	Garapati P V
2011-05-13	Reviewed	Desjardins M, English L
2016-05-16	Reviewed	Bergeron JJ
2022-03-23	Modified	Weiser JD

12 submitted entities found in this pathway, mapping to 46 Reactome entities

Input	UniProt Id	Input	UniProt Id	Input	UniProt Id
ENSG00000092010	Q06323	ENSG00000100567	P25788	ENSG00000108671	O00231
ENSG00000126067	P49721	ENSG00000137462	O60603	ENSG00000142507	P28072
ENSG00000143106	P28066	ENSG00000143546	P05109	ENSG00000163636	Q15008
ENSG00000168394	Q03518	ENSG00000173692	Q99460	ENSG00000234745	P01889, P03989, P10319, P18463, P18464, P18465, P30460, P30461, P30462, P30464, P30466, P30475, P30479, P30480, P30481, P30483, P30484, P30485, P30486, P30487, P30488, P30490, P30491, P30492, P30493, P30495, P30498, P30685, Q04826, Q29718, Q29836, Q29940, Q31610, Q31612, Q95365

13. Adaptive Immune System (R-HSA-1280218)

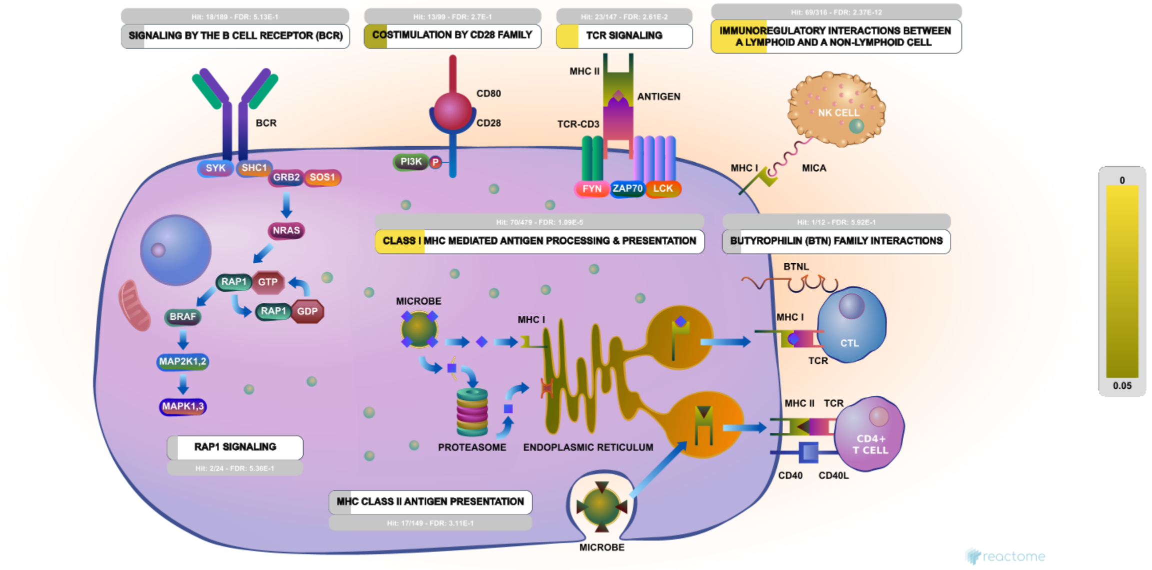

Adaptive immunity refers to antigen-specific immune response efficiently involved in clearing the pathogens. The adaptive immune system is comprised of B and T lymphocytes that express receptors with remarkable diversity tailored to recognize aspects of particular pathogens or antigens. During infection, dendritic cells (DC) which act as sentinels in the peripheral tissues recognize and pick up the pathogen in the form of antigenic determinants and then process these antigens and present them to T cells. These T cells of appropriate specificity respond to the antigen, and either kill the pathogen directly or secrete cytokines that will stimulate B lymphocyte response. B cells provide humoral immunity by secreting antibodies specific for the pathogen or antigen.

References

Minnicozzi M, Sawyer RT & Fenton MJ (2011). Innate immunity in allergic disease. Immunol Rev, 242, 106-27.

Cooper MD & Pancer Z (2006). The evolution of adaptive immunity. Annu Rev Immunol, 24, 497-518 .

Janeway CA Jr & Medzhitov R (2002). Innate immune recognition. Annu Rev Immunol, 20, 197-216.

Edit history

Date	Action	Author
2011-05-12	Created	Garapati P V
2011-05-22	Edited	May B, Jupe S, Garapati P V, de Bono B
2011-05-22	Authored	May B, Jupe S, Garapati P V, de Bono B
2011-05-28	Reviewed	Heemskerk JW, Bluestone JA, Elliott T, Trowsdale J, Esensten J
2022-03-23	Modified	Weiser JD

107 submitted entities found in this pathway, mapping to 144 Reactome entities

Input	UniProt Id	Input	UniProt Id	Input	UniProt Id
ENSG00000005844	P20701	ENSG00000072401	P51668	ENSG00000072803	Q9UKB1
ENSG00000088986	P63167	ENSG00000089692	P18627	ENSG00000092010	Q06323
ENSG00000095585	Q8WV28	ENSG00000100280	Q10567	ENSG00000100567	P25788
ENSG00000103653	P41240	ENSG00000104972	Q8NHL6	ENSG00000104974	O75019
ENSG00000105376	Q9UMF0	ENSG00000108671	O00231	ENSG00000109320	P19838
ENSG00000111796	Q12918	ENSG00000112146	Q9UK97	ENSG00000112195	Q5T2D2
ENSG00000113575	P67775	ENSG00000113615	O95486	ENSG00000114013	P33681, P42081
ENSG00000115085	P43403	ENSG00000115232	P13612	ENSG00000116663	Q9NRD1
ENSG00000116701	P19878	ENSG00000117984	P07339	ENSG00000120217	Q9NZQ7
ENSG00000122257	Q7Z6E9	ENSG00000124731	Q9NP99	ENSG00000126067	P49721
ENSG00000132109	P19474	ENSG00000132388	P62253	ENSG00000133661	P35247
ENSG00000134242	Q9Y2R2	ENSG00000134539	Q13241	ENSG00000134545	P26715
ENSG00000137462	O60603	ENSG00000137807	Q02241	ENSG00000138642	Q8IVU3
ENSG00000138646	Q9UII4	ENSG00000142507	P28072	ENSG00000142512	Q96LC7
ENSG00000142945	Q99661	ENSG00000143106	P28066	ENSG00000143546	P05109
ENSG00000145604	Q13309	ENSG00000147912	Q9UK96	ENSG00000150337	P12314, Q92637
ENSG00000150637	Q15762	ENSG00000152056	Q96PC3	ENSG00000153283	P40200
ENSG00000153563	P01732	ENSG00000154380	Q8N8S7	ENSG00000156587	O14933
ENSG00000158125	P47989	ENSG00000158485	P29016	ENSG00000158517	P14598
ENSG00000162063	P41002	ENSG00000162739	Q96DU3	ENSG00000162924	Q04864
ENSG00000163131	P25774	ENSG00000163519	Q6PIZ9	ENSG00000163636	Q15008
ENSG00000163808	Q9NS87	ENSG00000164308	Q6P179	ENSG00000164924	P63104
ENSG00000165025	P43405	ENSG00000165168	P04839	ENSG00000167553	Q9BQE3
ENSG00000167618	Q6ISS4	ENSG00000168394	Q03518	ENSG00000168995	Q9Y286
ENSG00000171051	P62942	ENSG00000172575	O95267	ENSG00000173692	Q99460
ENSG00000174775	P01112	ENSG00000174842	Q92990	ENSG00000176014	Q9BUF5
ENSG00000177889	P61088	ENSG00000178562	P10747	ENSG00000182566	Q6UXB4
ENSG00000185880	Q86WT6	ENSG00000185885	P13164	ENSG00000186074	Q8TDQ1
ENSG00000186265	Q7Z6A9	ENSG00000186407	Q496F6	ENSG00000186818	Q8NHJ6
ENSG00000187116	A6NI73	ENSG00000188389	Q15116	ENSG00000188404	P14151
ENSG00000189013	Q99706	ENSG00000198019	Q92637	ENSG00000198286	Q9BXL7
ENSG00000198821	P20963-1	ENSG00000198851	P07766	ENSG00000203747	P08637
ENSG00000213658	O43561	ENSG00000226650	O95239, Q2VIQ3	ENSG00000234745	P01889, P03989, P10319, P18463, P18464, P18465, P30460, P30461, P30462, P30464, P30466, P30475, P30479, P30480, P30481, P30483, P30484, P30485, P30486, P30487, P30488, P30490, P30491, P30492, P30493, P30495, P30498, P30685, Q04826, Q29718, Q29836, Q29940, Q31610, Q31612, Q95365
ENSG00000237541	P01906	ENSG00000244482	Q6PI73	ENSG00000254087	P07948
ENSG00000254415	Q96PQ1	ENSG00000254521	Q96PQ1	ENSG00000258947	Q13509
ENSG00000261456	Q3ZCM7	ENSG00000278030	P04435		

14. SARS-CoV-2 activates/modulates innate and adaptive immune responses (R-HSA-9705671)

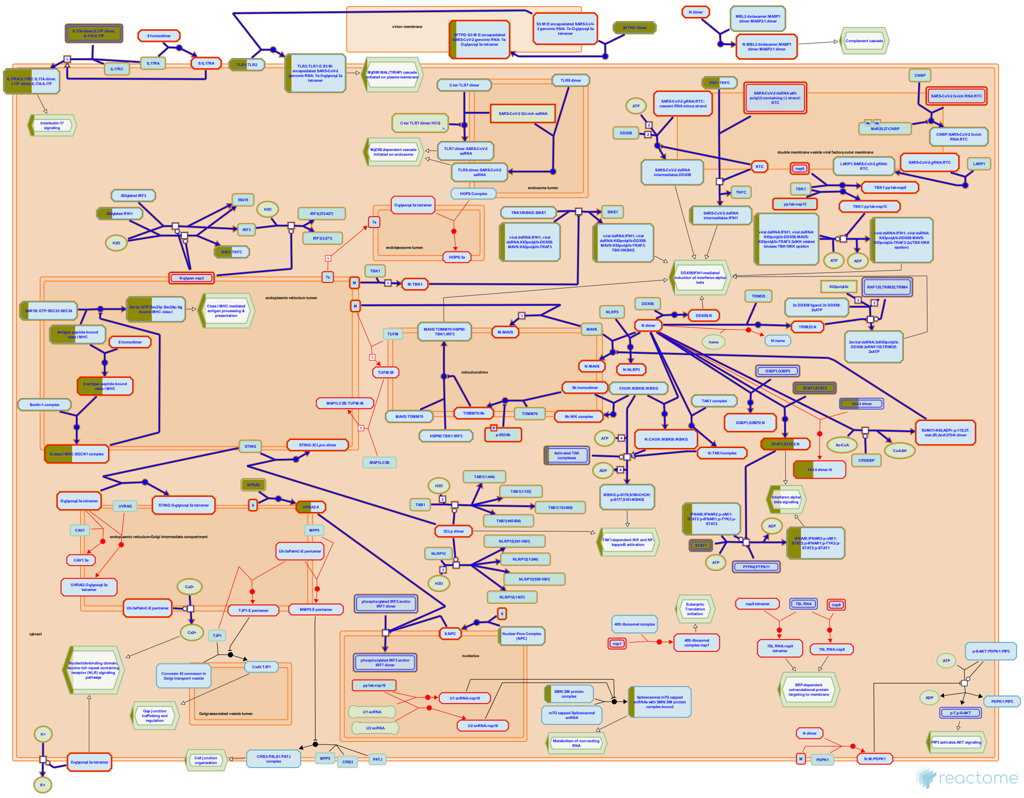

Diseases: COVID-19.

Coronaviruses (CoVs) are positive-sense RNA viruses that replicate in the interior of double membrane vesicles (DMV) in the cytoplasm of infected cells (Stertz S et al. 2007; Knoops K et al. 2008; V'kovski P et al. 2021). The viral replication and transcription are facilitated by virus-encoded non-structural proteins (SARS-CoV-2 nsp1–nsp16) that assemble to form a DMV-bound replication-transcription complex (RTC) (V'kovski P et al. 2021). The replication strategy of CoVs can generate both single-stranded RNA (ssRNA) and double-stranded RNA (dsRNA) species, that may act as pathogen-associated molecular patterns (PAMPs) recognized by pattern recognition receptor (PRR) such as toll-like receptor 7 (TLR7) and TLR8, antiviral innate immune response receptor RIG-I (also known as DEAD box protein 58, DDX58) and interferon-induced helicase C domain-containing protein 1 (IFIH1, also known as MDA5) (Salvi V et al. 2021; Campbell GR et al. 2021; Rebendenne A et al. 2021). The activated PRRs trigger signaling pathways to produce type I and type III interferons IFNs and proinflammatory mediators that perform antiviral functions. This Reactome module describes the mechanisms underlying PRR-mediated sensing of the severe acute respiratory syndrome coronavirus type 2 (SARS-CoV-2) infection. First, endosomal recognition of viral ssRNA occurs by means of TLR7 and TLR8, which detect GU-rich ssRNA sequences (Salvi V et al. 2021; Campbell GR et al. 2021). Second, SARS-CoV-2 dsRNA replication intermediates can be recognized by cytoplasmic receptors DDX58 and IFIH1 which bind to mitochondrial antiviral-signaling protein (MAVS, IPS-1) to induce the IFN-mediated antiviral response (Rebendenne A et al. 2021; Yin X et al. 2021). In addition, SARS-CoV-2 E can be sensed by TLR2 (Zheng M et al. 2021). Further, cellular nucleic acid-binding protein (CNBP) and La-related protein 1 (LARP1) can directly bind SARS-CoV-2 gRNA to repress SARS-CoV-2 replication (Schmidt N et al. 2021). This module also describes several strategies developed by SARS-CoV-2 to evade or alter host immunity, including escaping innate immune

sensors, inhibiting IFN production and signaling, and evading antiviral function of IFN stimulated gene (ISG) products. For example, SARS-CoV-2 encodes nsp14 and nsp16 which possess guanine-N7-methyltransferase activity and 2’-O-methyl-transferase activity respectively (Ogando NS et al. 2020; Krafcikova P et al. 2020; Viswanatha T et al. 2020; Lin S et al. 2021; Yan L et al. 2021). In human coronaviruses nsp14 generates 5' cap-0 viral RNA (m7GpppN, guanine N7-methylated) and nsp16 further methylates cap-0 viral RNA. These viral RNA modifications mimic the 5'-cap structure of host mRNAs allowing the virus to efficiently evade recognition by cytosolic DDX58 and IFIH1 (Chen Y et al. 2009, 2011; Daffis S et al. 2010, shown for CoVs such as SARS-CoV-1 and MERS-CoV). Structural studies and computational analysis suggest that properties and biological functions of SARS-CoV-2 nsp14 and nsp16 could be very similar to these of SARS-CoV-1 (Rosas-Lemus M et al. 2020; Lin S et al. 2020; Viswanathan T et al. 2020; Krafcikova P et al. 2020; Jiang Y et al. 2020; Wilamowski M et al. 2021). Further, the uridylatespecific endoribonuclease (EndoU) activity of SARS-CoV-2 nsp15 degrades viral RNA to hide it from innate immune sensors (Frazier MN et al. 2021). Moreover, SARS-CoV-2 encodes several proteins that directly bind to host targets associated with SARSCoV2 infection and cytokine production (Shin D et al. 2020; Viswanathan T et al. 2020; Xia H et al. 2020; Matsuyama T et al. 2020; Yuen CK et al. 2020; reviewed by Park A & Iwasaki A 2020). This Reactome module describes several such binding events and their consequences. For example, as a deubiquitinating and deISGylating enzyme, viral nsp3 binds to and removes ISG15 from signaling proteins such as IRF3 and IFIH1 thereby modulating the formation of signaling complexes and the activation of IRF3/7 and NF-kappaB (Liu CQ et al. 2021). Binding of SARS-CoV-2 nsp6, nsp13 or membrane (M) protein to cytosolic TBK1 prevents IRF3/7 activation and inhibits IFN production downstream of DDX58, IFIH1, MAVS and STING signaling pathways (Xia H et al. 2020; Sui L et al. 2021). Next, M protein targets MAVS to prevent the formation of the MAVS signalosome complex and thereby inhibits downstream signaling pathways of DDX58 and IFIH1 (Fu YZ et al. 2021). Binding of SARS-CoV-2 nucleocapsid (N) protein to E3 ubiquitin ligase TRIM25 inhibits TRIM25-mediated DDX58 ubiquitination and the DDX58 signaling pathway (Gori SG et al. 2021). N interacts with NLRP3 to promote the assembly and activation of the NLRP3 inflammasome (Pan P et al. 2021). The interaction between viral N and MASP2 promotes MASP2-mediated cleavage of C4 (Ali YM et al. 2021) and C2 (Kang S et al. 2021) leading to the hyperactivation of the complement system. Besides, viral N promotes NF-kappaB activation by targeting signaling complexes of TAK1 and IKK (Wu Y et al. 2021). The ion channel activities of accessory protein ORF3a or 3a (open reading frame 3a) and SARSCoV2 envelope (E) protein contribute to activation of the NLRP3 inflammasome leading to highly inflammatory pyroptotic cell death (based on findings for SARS-CoV-1, Siu KL et al. 2019). SARS-CoV-2 nsp5 protease (3CLpro) cleaves TAB1, a component of the TAK1 complex, thus inhibiting NF-kappaB activation (Moustaqil M et al .2021). 3CLpro targets NLRP12 which modulates the expression of inflammatory cytokines through the regulation of the NFkappaB and MAPK pathways (Moustaqil M et al. 2021). SARS-CoV-2 6 (ORF6) interacts with importin KPNA2 and components of the nuclear pore complex, NUP98 and RAE1, to block nuclear translocation of IRF3, STAT1 and STAT2 (Xia H et al. 2020; Miorin L et al. 2020). SARS-CoV-2 9b (ORF9b) inhibits the MAVS-mediated production of type I IFNs by targeting TOMM70 on the mitochondria (Jiang HW et al. 2020). Binding of mitochondrial viral 9 to IKBKG prevents MAVS-dependent NF-kappaB activation (Wu J et al. 2021). Although the evasion mechanisms are mainly conserved between SARS-CoV-1 and SARS-CoV-2 (Gordon DE et al. 2020), studies identified SARS-CoV-2-specific modulations of host immune response that may contribute to pathophysiological determinants of COVID-19 (Gordon DE et al. 2020; Schiller HB et al. 2021). This Reactome module describes several virus-host interactions identified in cells during SARS-CoV-2, but not SARS-CoV-1, infection. For example, SARS-CoV-2 8 (ORF8) regulates the expression of class I MHC on the surface of the infected cells through

an autophagy-dependent lysosomal degradation of class I MHC (Zhang Y et al. 2021). At the plasma membrane, binding of secreted viral 8 to IL17RA activates IL17 signaling pathway leading to an increased secretion of cytokines/chemokines thus contributing to cytokine storm during SARS-CoV-2 infection (Lin X et al. 2021). Furthermore, SARS-CoV-2-host interactome and proteomics studies identified various human proteins that are targeted by SARS-CoV-2 proteins (Gordon DE et al. 2020a, b; Bojkova D et al. 2020; Stukalov A et al. 2021; Li J et al. 2021; Messina F et al. 2021). This Reactome module does not cover all identified SARS-CoV-2–human interactions; the module describes those associations that were functionally validated.

References

Bouayad A (2020). Innate immune evasion by SARS-CoV-2: Comparison with SARS-CoV. Rev Med Virol, 30, 1-9.

Sgarbanti M, Hiscott J, Di Carlo D & Palermo E (2021). Type I Interferons in COVID-19 Pathogenesis. Biology (Basel), 10.

Park A & Iwasaki A (2020). Type I and Type III Interferons - Induction, Signaling, Evasion, and Application to Combat COVID-19. Cell Host Microbe, 27, 870-878.

Lowery SA, Perlman S & Sariol A (2021). Innate immune and inflammatory responses to SARS-CoV-2: Implications for COVID-19. Cell Host Microbe, 29, 1052-1062.

Sandini S, Ricci D, Coccia EM, Etna MP, Rizzo F & Severa M (2021). Innate Immune Response to SARS-CoV-2 Infection: From Cells to Soluble Mediators. Int J Mol Sci, 22.

Edit history

Date	Action	Author
2020-10-28	Created	Shamovsky V
2021-05-03	Authored	Stephan R
2021-10-26	Authored	Shamovsky V
2022-02-17	Edited	Shamovsky V
2022-02-17	Reviewed	Messina F
2022-03-03	Modified	Shamovsky V

15 submitted entities found in this pathway, mapping to 51 Reactome entities

Input	UniProt Id	Input	UniProt Id	Input	UniProt Id
ENSG00000093000	Q9UKX7	ENSG00000112115	Q16552	ENSG00000112116	Q96PD4
ENSG00000113615	O95486	ENSG00000115267	Q9BYX4	ENSG00000115415	P42224, P42224-1, P42224-2
ENSG00000133661	P35247	ENSG00000134070	O43187	ENSG00000137462	O60603
ENSG00000167207	Q9HC29	ENSG00000170581	P52630	ENSG00000177889	P61088
ENSG00000182481	P52292	ENSG00000233816	P01562	ENSG00000234745	P01889, P03989, P10319, P18463, P18464, P18465, P30460, P30461, P30462, P30464, P30466, P30475, P30479, P30480, P30481, P30483, P30484, P30485, P30486, P30487, P30488, P30490, P30491, P30492, P30493, P30495, P30498, P30685, Q04826, Q29718, Q29836, Q29940, Q31610, Q31612, Q95365

15. Cell Cycle (R-HSA-1640170)

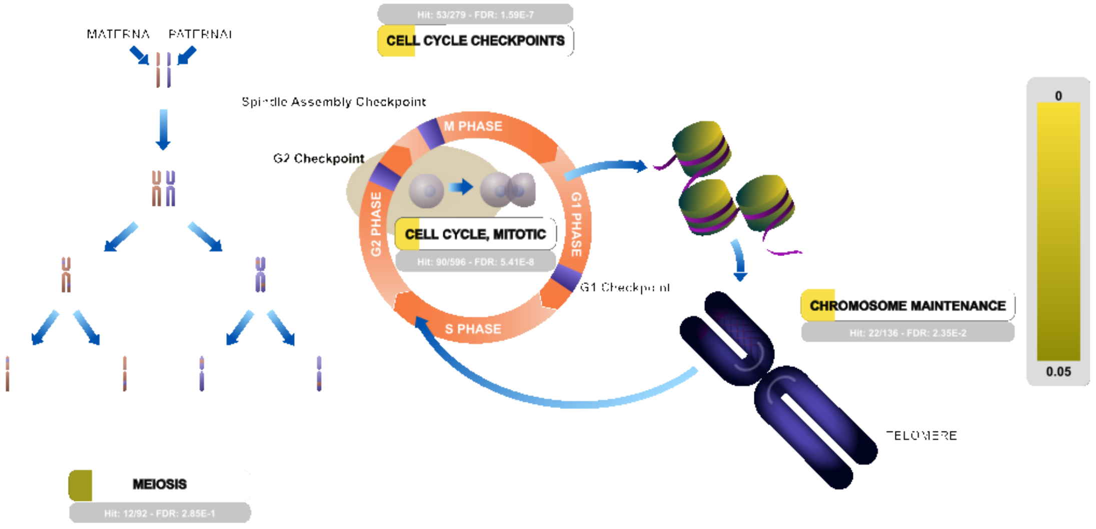

The replication of the genome and the subsequent segregation of chromosomes into daughter cells are controlled by a series of events collectively known as the **cell cycle**. DNA replication is carried out during a discrete temporal period known as the S (synthesis)-phase, and chromosome segregation occurs during a massive reorganization to cellular architecture at mitosis. Two gap-phases separate these major cell cycle events: G1 between mitosis and S-phase, and G2 between S-phase and mitosis. In the development of the human body, cells can exit the cell cycle for a period and enter a quiescent state known as G0, or terminally differentiate into cells that will not divide again, but undergo morphological development to carry out the wide variety of specialized functions of individual tissues.

A family of protein serine/threonine kinases known as the cyclin-dependent kinases (CDKs) controls progression through the cell cycle. As the name suggests, the activity of the catalytic subunit is dependent on binding to a cyclin partner. The human genome encodes several cyclins and several CDKs, with their names largely derived from the order in which they were identified. The oscillation of cyclin abundance is one important mechanism by which these enzymes phosphorylate key substrates to promote events at the relevant time and place. Additional post-translational modifications and interactions with regulatory proteins ensure that CDK activity is precisely regulated, frequently confined to a narrow window of activity.

In addition, genome integrity in the cell cycle is maintained by the action of a number of signal transduction pathways, known as **cell cycle checkpoints**, which monitor the accuracy and completeness of DNA replication during S phase and the orderly chromosomal condensation, pairing and partition into daughter cells during mitosis.

Replication of telomeric DNA at the ends of human chromosomes and packaging of their centromeres into chromatin are two aspects of **chromosome maintenance** that are integral parts of the cell cycle.

Meiosis is the specialized form of cell division that generates haploid gametes from diploid germ cells, associated with recombination (exchange of genetic material between chromosomal homologs).

References

Edit history

Date	Action	Author
2011-10-10	Edited	Matthews L
2011-10-10	Created	Matthews L
2022-03-23	Modified	Weiser JD

96 submitted entities found in this pathway, mapping to 111 Reactome entities

Input	UniProt Id	Input	UniProt Id	Input	UniProt Id
ENSG00000006634	Q9UBU7	ENSG00000007968	Q14209	ENSG00000051180	Q06609
ENSG00000072401	P51668	ENSG00000072571	O75330	ENSG00000072803	Q9UKB1
ENSG00000073111	P49736	ENSG00000087586	O14965	ENSG00000088986	P63167
ENSG00000089685	O15392	ENSG00000092010	Q06323	ENSG00000092853	Q9HAW4
ENSG00000093000	Q9UKX7	ENSG00000093009	O75419	ENSG00000094804	Q99741
ENSG00000100401	P46060	ENSG00000100479	P56282	ENSG00000100567	P25788
ENSG00000104147	O43482	ENSG00000105173	P24864	ENSG00000108671	O00231
ENSG00000108702	P51946	ENSG00000111206	Q08050	ENSG00000112242	O00716
ENSG00000113368	P20700	ENSG00000113575	P67775	ENSG00000113810	Q9NTJ3
ENSG00000117650	P51955	ENSG00000117724	P49454	ENSG00000120334	Q8N0S6
ENSG00000120539	Q96GX5	ENSG00000122952	O95229	ENSG00000124635	P06899
ENSG00000124762	P38936	ENSG00000126067	P49721	ENSG00000129810	Q5FBB7
ENSG00000132341	P62826	ENSG00000132646	P12004	ENSG00000134308	P27348
ENSG00000134690	Q53HL2	ENSG00000136492	Q9BX63	ENSG00000137807	Q02241
ENSG00000137812	Q8NG31	ENSG00000138346	P51530	ENSG00000139618	P51587
ENSG00000139687	P06400	ENSG00000140451	Q9H611	ENSG00000142507	P28072
ENSG00000142945	Q99661	ENSG00000143106	P28066	ENSG00000143156	Q9Y5B8
ENSG00000143228	Q9BZD4	ENSG00000145386	P20248	ENSG00000145604	Q13309
ENSG00000147889	P42771, P42772, Q8N726	ENSG00000152253	Q9HBM1	ENSG00000153147	O60264
ENSG00000158402	P30307	ENSG00000163535	Q562F6	ENSG00000163636	Q15008
ENSG00000163882	P52434	ENSG00000163918	P35249, P35250	ENSG00000163961	Q8IYW5
ENSG00000164032	P0C0S5	ENSG00000164045	P30304	ENSG00000164109	Q13257
ENSG00000164924	P63104	ENSG00000167553	Q9BQE3	ENSG00000167900	P04183
ENSG00000170312	P06493	ENSG00000172531	P62140	ENSG00000173692	Q99460
ENSG00000175643	Q96E14	ENSG00000175792	Q9Y265	ENSG00000175793	P31947
ENSG00000176014	Q9BUF5	ENSG00000176890	P04818	ENSG00000177889	P61088
ENSG00000181938	Q9BRX5	ENSG00000182628	Q8WVK7	ENSG00000186871	Q2NKX8
ENSG00000188486	P16104	ENSG00000196747	Q99878	ENSG00000197061	P62805
ENSG00000197238	P62805	ENSG00000203760	Q5EE01	ENSG00000228716	P00374
ENSG00000246705	Q9BTM1	ENSG00000254087	P07948	ENSG00000258366	Q9NZ71
ENSG00000258947	Q13509	ENSG00000261456	Q3ZCM7	ENSG00000274997	Q99878
ENSG00000277775	P68431	ENSG00000278463	P04908	ENSG00000278588	P62807

Input	Ensembl Id	Input	Ensembl Id	Input	Ensembl Id
ENSG00000093009	ENSG00000093009	ENSG00000094804	ENSG00000094804	ENSG00000105173	ENSG00000105173
ENSG00000117724	ENSG00000117724	ENSG00000124762	ENSG00000124762	ENSG00000132646	ENSG00000132646
ENSG00000145386	ENSG00000145386	ENSG00000164045	ENSG00000164045	ENSG00000167900	ENSG00000167900
ENSG00000170312	ENSG00000170312	ENSG00000176890	ENSG00000176890	ENSG00000228716	ENSG00000228716

16. Innate Immune System (R-HSA-168249)

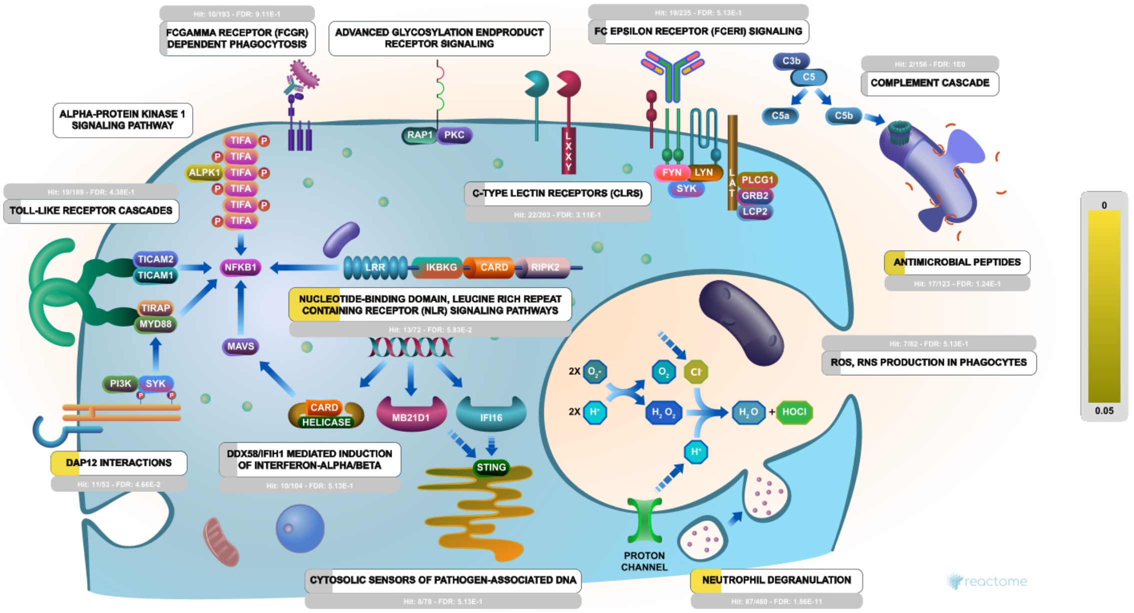

Innate immunity encompases the nonspecific part of immunity tha are part of an individual's natural biologic makeup

References

Edit history

Date	Action	Author
2005-11-11	Created	Gillespie ME
2022-03-23	Modified	Weiser JD

165 submitted entities found in this pathway, mapping to 172 Reactome entities

Input	UniProt Id	Input	UniProt Id	Input	UniProt Id
ENSG00000003400	Q92851	ENSG00000005844	P20701	ENSG00000007171	P35228
ENSG00000008516	Q9NPA2	ENSG00000021355	P30740	ENSG00000023445	Q13489, Q13490
ENSG00000057149	P29508	ENSG00000058063	Q9Y2G3	ENSG00000072401	P51668
ENSG00000072803	Q9UKB1	ENSG00000088986	P63167	ENSG00000090376	Q9Y616
ENSG00000090382	P61626	ENSG00000092010	Q06323	ENSG00000092929	Q70J99
ENSG00000100292	P09601	ENSG00000100453	P08311	ENSG00000100504	P06737
ENSG00000100554	Q9Y5K8	ENSG00000100567	P25788	ENSG00000103187	Q14019
ENSG00000103313	O15553	ENSG00000103415	P30519	ENSG00000103490	Q9ULZ3
ENSG00000108671	O00231	ENSG00000108771	Q96C10	ENSG00000109320	P19838
ENSG00000109971	P11142	ENSG00000110934	Q9UBW5	ENSG00000111229	O15145
ENSG00000111816	P42685	ENSG00000112299	O95497	ENSG00000113356	O15318
ENSG00000113575	P67775	ENSG00000114738	Q16644	ENSG00000115091	P61158
ENSG00000115267	Q9BYX4	ENSG00000115523	P22749	ENSG00000116260	O00391
ENSG00000116701	P19878	ENSG00000117971	P30926	ENSG00000117984	P07339
ENSG00000122861	P00749	ENSG00000124233	P04279	ENSG00000124256	Q9H171
ENSG00000124731	Q9NP99	ENSG00000126067	P49721	ENSG00000130303	Q10589
ENSG00000130429	O15143	ENSG00000132109	P19474	ENSG00000133661	P35247

Input	UniProt Id	Input	UniProt Id	Input	UniProt Id
ENSG00000134061	Q99467	ENSG00000134070	O43187	ENSG00000134539	Q13241
ENSG00000134545	P26717	ENSG00000134827	P20061	ENSG00000137462	O60603
ENSG00000137563	Q92820	ENSG00000137752	P29466	ENSG00000138646	Q9UII4
ENSG00000139572	Q9NQS5	ENSG00000140368	O43586	ENSG00000140678	P20702
ENSG00000142185	O94759	ENSG00000142507	P28072	ENSG00000142512	A6NMB1
ENSG00000142583	P22732	ENSG00000143106	P28066	ENSG00000143119	P19397
ENSG00000143226	P12318	ENSG00000143546	P05109	ENSG00000143556	P31151, Q86SG5
ENSG00000143621	Q12905	ENSG00000145113	Q99102	ENSG00000145287	Q9NZF1
ENSG00000147614	Q8N8Y2	ENSG00000147813	Q6XQN6	ENSG00000149925	P04075
ENSG00000150337	P12314	ENSG00000151418	Q96LB4	ENSG00000151651	P78325
ENSG00000153395	Q8NF37	ENSG00000156234	P02775	ENSG00000156575	Q9Y2Y8
ENSG00000156587	O14933	ENSG00000158517	P14598	ENSG00000159527	Q96LB9
ENSG00000160691	P29353-2	ENSG00000160703	Q86UT6	ENSG00000160796	Q6ZNJ1
ENSG00000160883	P52790	ENSG00000162747	O75015	ENSG00000163131	P25774
ENSG00000163563	P41218	ENSG00000163565	Q16666	ENSG00000163568	O14862
ENSG00000163636	Q15008	ENSG00000163661	P26022	ENSG00000163734	P09341
ENSG00000163736	P02775	ENSG00000163739	P09341	ENSG00000163882	P52434
ENSG00000163993	P25815	ENSG00000164047	P49913	ENSG00000164062	P13798
ENSG00000164086	Q16829	ENSG00000165025	P43405	ENSG00000165168	P04839
ENSG00000166226	P78371	ENSG00000166278	P06681	ENSG00000166523	Q9ULY5
ENSG00000166527	Q8WXI8	ENSG00000167207	Q9HC29	ENSG00000167984	Q7RTR2
ENSG00000168884	Q8NFZ5	ENSG00000169228	Q969Q5	ENSG00000169299	Q96G03
ENSG00000169385	P10153	ENSG00000170515	Q9UQ80	ENSG00000170956	P40198
ENSG00000171049	P21462, P25090	ENSG00000171051	P21462	ENSG00000172575	O95267
ENSG00000172757	P23528	ENSG00000173692	Q99460	ENSG00000173801	P14923
ENSG00000174775	P01112	ENSG00000175463	Q8IV04	ENSG00000176797	P81534
ENSG00000177105	P84095	ENSG00000177243	P81534	ENSG00000177257	O15263
ENSG00000177556	O00244	ENSG00000177889	P61088	ENSG00000178372	Q9NZT1
ENSG00000181467	P61225	ENSG00000183019	Q8IX19	ENSG00000186191	P59827
ENSG00000186407	Q496F6	ENSG00000186431	P24071	ENSG00000188404	P14151
ENSG00000196743	P17900	ENSG00000196776	Q08722	ENSG00000196954	P49662
ENSG00000197249	P01009	ENSG00000198286	Q9BXL7	ENSG00000198805	P00491
ENSG00000198821	P20963-1	ENSG00000203747	P08637	ENSG00000205420	P04264
ENSG00000205809	P26717	ENSG00000205846	Q6EIG7, Q8WTT0	ENSG00000205882	Q4QY38
ENSG00000213658	O43561-2	ENSG00000214643	Q30KQ1	ENSG00000228278	P19652, Q8N138
ENSG00000233816	P01562	ENSG00000234745	P01889, Q95365	ENSG00000239732	Q9NR96
ENSG00000239839	P59666	ENSG00000242550	P48595	ENSG00000243649	P00751
ENSG00000254087	P07948	ENSG00000254415	Q08ET2	ENSG00000257017	P00738

Input	Ensembl Id
ENSG00000233816	ENSG00000233816

17. Mitotic G1 phase and G1/S transition (R-HSA-453279)

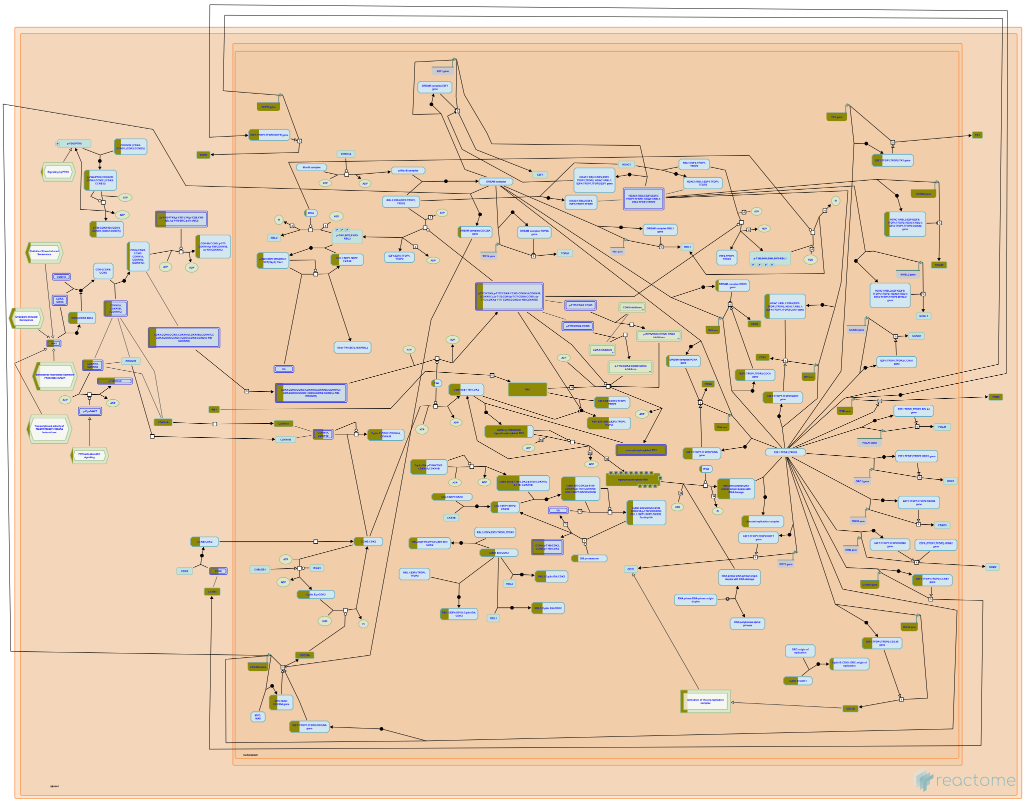

Mitotic G1-G1/S phase involves G1 phase of the mitotic interphase and G1/S transition, when a cell commits to DNA replication and divison genetic and cellular material to two daughter cells.

During early G1, cells can enter a quiescent G0 state. In quiescent cells, the evolutionarily conserved DREAM complex, consisting of the pocket protein family member p130 (RBL2), bound to E2F4 or E2F5, and the MuvB complex, represses transcription of cell cycle genes (reviewed by Sadasivam and DeCaprio 2013).

During early G1 phase in actively cycling cells, transcription of cell cycle genes is repressed by another pocket protein family member, p107 (RBL1), which forms a complex with E2F4 (Ferreira et al. 1998, Cobrinik 2005). RB1 tumor suppressor, the product of the retinoblastoma susceptibility gene, is the third member of the pocket protein family. RB1 binds to E2F transcription factors E2F1, E2F2 and E2F3 and inhibits their transcriptional activity, resulting in prevention of G1/S transition (Chellappan et al. 1991, Bagchi et al. 1991, Chittenden et al. 1991, Lees et al. 1993, Hiebert 1993, Wu et al. 2001). Once RB1 is phosphorylated on serine residue S795 by Cyclin D:CDK4/6 complexes, it can no longer associate with and inhibit E2F1-3. Thus, CDK4/6-mediated phosphorylation of RB1 leads to transcriptional activation of E2F1-3 target genes needed for the S phase of the cell cycle (Connell-Crowley et al. 1997). CDK2, in complex with cyclin E, contributes to RB1 inactivation and also activates proteins needed for the initiation of DNA replication (Zhang 2007). Expression of D type cyclins is regulated by extracellular mitogens (Cheng et al. 1998, Depoortere et al. 1998). Catalytic activities of CDK4/6 and CDK2 are controlled by CDK inhibitors of the INK4 family (Serrano et al. 1993, Hannon and Beach 1994, Guan et al. 1994, Guan et al. 1996, Parry et al. 1995) and the Cip/Kip family, respectively.

References

Zhang H (2007). Life without kinase: cyclin E promotes DNA replication licensing and beyond. Mol. Cell, 25, 175-6.

Robin P, Harel-Bellan A, Magnaghi-Jaulin L, Trouche D & Ferreira R (1998). The three members of the pocket proteins family share the ability to repress E2F activity through recruitment of a histone deacetylase. Proc Natl Acad Sci U S A, 95, 10493-8.

Chittenden T, Kaelin WG & Livingston DM (1991). The T/E1A-binding domain of the retinoblastoma product can interact selectively with a sequence-specific DNA-binding protein. Cell, 65, 1073-82.

Cobrinik D (2005). Pocket proteins and cell cycle control. Oncogene, 24, 2796-809.

Depoortere F, Dumont JE, Van Keymeulen A, Costagliola S, Dremier S, Roger PP, ... Bartek J (1998). A requirement for cyclin D3-cyclin-dependent kinase (cdk)-4 assembly in the cyclic adenosine monophosphate-dependent proliferation of thyrocytes. J. Cell Biol., 140, 1427-39.

Edit history

Date	Action	Author
2010-01-19	Edited	Matthews L
2010-01-20	Authored	Matthews L
2010-01-20	Created	Matthews L
2011-06-15	Reviewed	Grana X
2011-08-25	Reviewed	MacPherson D
2011-08-26	Revised	Orlic-Milacic M
2011-08-26	Authored	Orlic-Milacic M
2017-02-08	Edited	Orlic-Milacic M
2018-07-10	Reviewed	Manfredi JJ
2022-03-23	Modified	Weiser JD

30 submitted entities found in this pathway, mapping to 41 Reactome entities

Input	UniProt Id	Input	UniProt Id	Input	UniProt Id
ENSG00000006634	Q9UBU7	ENSG00000007968	Q14209	ENSG00000073111	P49736
ENSG00000092010	Q06323	ENSG00000093009	O75419	ENSG00000094804	Q99741
ENSG00000100479	P56282	ENSG00000100567	P25788	ENSG00000105173	P24864
ENSG00000108671	O00231	ENSG00000108702	P51946	ENSG00000112242	O00716
ENSG00000113575	P67775	ENSG00000124762	P38936	ENSG00000126067	P49721
ENSG00000132646	P12004	ENSG00000139687	P06400	ENSG00000142507	P28072
ENSG00000143106	P28066	ENSG00000145386	P20248	ENSG00000145604	Q13309
ENSG00000147889	P42771, P42772	ENSG00000163636	Q15008	ENSG00000164045	P30304
ENSG00000167900	P04183	ENSG00000170312	P06493	ENSG00000173692	Q99460
ENSG00000176890	P04818	ENSG00000228716	P00374	ENSG00000254087	P07948

Input	Ensembl Id	Input	Ensembl Id	Input	Ensembl Id
ENSG00000093009	ENSG00000093009	ENSG00000094804	ENSG00000094804	ENSG00000105173	ENSG00000105173
ENSG00000132646	ENSG00000132646	ENSG00000145386	ENSG00000145386	ENSG00000164045	ENSG00000164045
ENSG00000167900	ENSG00000167900	ENSG00000170312	ENSG00000170312	ENSG00000176890	ENSG00000176890

Input	Ensembl Id	Input	Ensembl Id	Input	Ensembl Id
ENSG00000228716	ENSG00000228716				

18. Cell Cycle, Mitotic (R-HSA-69278)

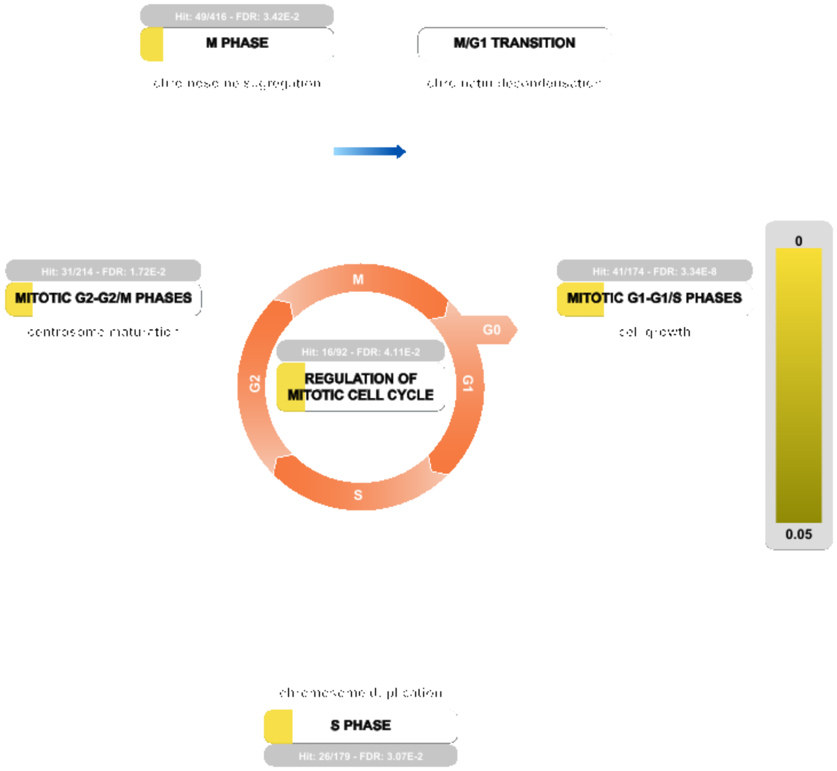

The events of replication of the genome and the subsequent segregation of chromosomes into daughter cells make up the cell cycle. DNA replication is carried out during a discrete temporal period known as the S (synthesis)-phase, and chromosome segregation occurs during a massive re-organization of cellular architecture at mitosis. Two gap-phases separate these cell cycle events: G1 between mitosis and S-phase, and G2 between S-phase and mitosis. Cells can exit the cell cycle for a period and enter a quiescent state known as G0, or terminally differentiate into cells that will not divide again, but undergo morphological development to carry out the wide variety of specialized functions of individual tissues.

A family of protein serine/threonine kinases known as the cyclin-dependent kinases (CDKs) controls progression through the cell cycle. As the name suggests, the kinase activity of the catalytic subunits is dependent on binding to cyclin partners, and control of cyclin abundance is one of several mechanisms by which CDK activity is regulated throughout the cell cycle.

A complex network of regulatory processes determines whether a quiescent cell (in G0 or early G1) will leave this state and initiate the processes to replicate its chromosomal DNA and divide. This regulation, during the **Mitotic G1-G1/S phases** of the cell cycle, centers on transcriptional regulation by the DREAM complex, with major roles for D and E type cyclin proteins.

Chromosomal DNA synthesis occurs in the **S phase**, or the synthesis phase, of the cell cycle. The cell duplicates its hereditary material, and two copies of each chromosome are formed. A key aspect of the **regulation of DNA** replication is the assembly and modification of a pre-replication complex assembled on ORC proteins.

Mitotic G2-G2/M phases encompass the interval between the completion of DNA synthesis and the beginning of mitosis. During G2, the cytoplasmic content of the cell increases. At G2/M transition, duplicated centrosomes mature and separate and CDK1:cyclin B complexes become active, setting the stage for spindle assembly and chromosome condensation at the start of mitotic **M phase**. Mitosis, or M phase, results in the generation of two daughter cells each with a complete diploid set of chromosomes. Events of the **M/G1 transition**, progression out of mitosis and division of the cell into two daughters (cytokinesis) are regulated by the Anaphase Promoting Complex.

The Anaphase Promoting Complex or Cyclosome (APC/C) plays additional roles in **regulation of the mitotic cell cycle**, insuring the appropriate length of the G1 phase. The APC/C itself is regulated by phosphorylation and interactions with checkpoint proteins.

References

Edit history

Date	Action	Author
2005-01-01	Authored	Walworth N, Bosco G, O'Donnell M
2005-01-01	Created	Walworth N, Bosco G, O'Donnell M
2010-01-19	Revised	Matthews L
2011-06-15	Reviewed	Grana X
2011-08-25	Reviewed	MacPherson D
2011-08-26	Revised	Orlic-Milacic M
2013-11-25	Edited	Matthews L, Gopinathrao G
2018-07-10	Reviewed	Manfredi JJ
2022-03-23	Modified	Weiser JD

79 submitted entities found in this pathway, mapping to 92 Reactome entities

Input	UniProt Id	Input	UniProt Id	Input	UniProt Id
ENSG00000006634	Q9UBU7	ENSG00000007968	Q14209	ENSG00000072401	P51668
ENSG00000072571	O75330	ENSG00000072803	Q9UKB1	ENSG00000073111	P49736
ENSG00000087586	O14965	ENSG00000088986	P63167	ENSG00000089685	O15392
ENSG00000092010	Q06323	ENSG00000093000	Q9UKX7	ENSG00000093009	O75419
ENSG00000094804	Q99741	ENSG00000100401	P46060	ENSG00000100479	P56282
ENSG00000100567	P25788	ENSG00000105173	P24864	ENSG00000108671	O00231
ENSG00000108702	P51946	ENSG00000111206	Q08050	ENSG00000112242	O00716
ENSG00000113368	P20700	ENSG00000113575	P67775	ENSG00000113810	Q9NTJ3
ENSG00000117650	P51955	ENSG00000117724	P49454	ENSG00000120334	Q8N0S6
ENSG00000120539	Q96GX5	ENSG00000122952	O95229	ENSG00000124635	P06899
ENSG00000124762	P38936	ENSG00000126067	P49721	ENSG00000129810	Q5FBB7
ENSG00000132341	P62826	ENSG00000132646	P12004	ENSG00000134690	Q53HL2
ENSG00000137807	Q02241	ENSG00000137812	Q8NG31	ENSG00000138346	P51530
ENSG00000139687	P06400	ENSG00000142507	P28072	ENSG00000142945	Q99661
ENSG00000143106	P28066	ENSG00000143156	Q9Y5B8	ENSG00000143228	Q9BZD4
ENSG00000145386	P20248	ENSG00000145604	Q13309	ENSG00000147889	P42771, P42772
ENSG00000152253	Q9HBM1	ENSG00000158402	P30307	ENSG00000163535	Q562F6

Input	UniProt Id	Input	UniProt Id	Input	UniProt Id
ENSG00000163636	Q15008	ENSG00000163918	P35249, P35250	ENSG00000164032	P0C0S5
ENSG00000164045	P30304	ENSG00000164109	Q13257	ENSG00000167553	Q9BQE3
ENSG00000167900	P04183	ENSG00000170312	P06493	ENSG00000172531	P62140
ENSG00000173692	Q99460	ENSG00000176014	Q9BUF5	ENSG00000176890	P04818
ENSG00000181938	Q9BRX5	ENSG00000182628	Q8WVK7	ENSG00000186871	Q2NKX8
ENSG00000188486	P16104	ENSG00000196747	Q99878	ENSG00000197061	P62805
ENSG00000197238	P62805	ENSG00000228716	P00374	ENSG00000246705	Q9BTM1
ENSG00000254087	P07948	ENSG00000258947	Q13509	ENSG00000261456	Q3ZCM7
ENSG00000274997	Q99878	ENSG00000277775	P68431	ENSG00000278463	P04908
ENSG00000278588	P62807				

Input	Ensembl Id	Input	Ensembl Id	Input	Ensembl Id
ENSG00000093009	ENSG00000093009	ENSG00000094804	ENSG00000094804	ENSG00000105173	ENSG00000105173
ENSG00000117724	ENSG00000117724	ENSG00000132646	ENSG00000132646	ENSG00000145386	ENSG00000145386
ENSG00000164045	ENSG00000164045	ENSG00000167900	ENSG00000167900	ENSG00000170312	ENSG00000170312
ENSG00000176890	ENSG00000176890	ENSG00000228716	ENSG00000228716		

19. Cell Cycle Checkpoints (R-HSA-69620)

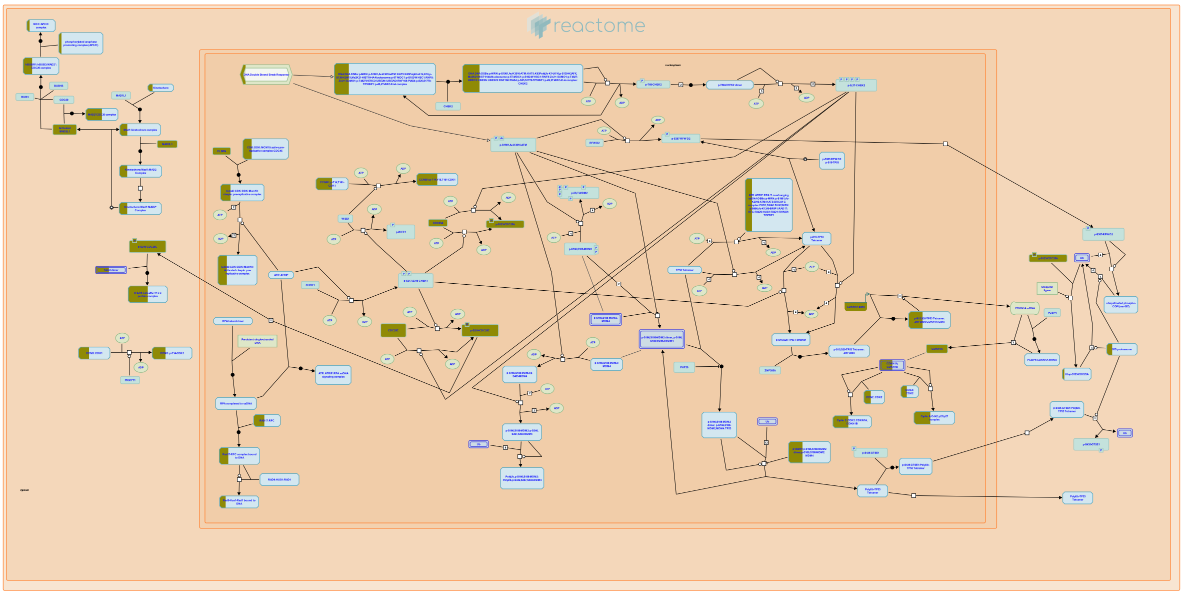

A hallmark of the human cell cycle in normal somatic cells is its precision. This remarkable fidelity is achieved by a number of signal transduction pathways, known as checkpoints, which monitor cell cycle progression ensuring an interdependency of S-phase and mitosis, the integrity of the genome and the fidelity of chromosome segregation.

Checkpoints are layers of control that act to delay CDK activation when defects in the division program occur. As the CDKs functioning at different points in the cell cycle are regulated by different means, the various checkpoints differ in the biochemical mechanisms by which they elicit their effect. However, all checkpoints share a common hierarchy of a sensor, signal transducers, and effectors that interact with the CDKs.

The stability of the genome in somatic cells contrasts to the almost universal genomic instability of tumor cells. There are a number of documented genetic lesions in checkpoint genes, or in cell cycle genes themselves, which result either directly in cancer or in a predisposition to certain cancer types. Indeed, restraint over cell cycle progression and failure to monitor genome integrity are likely prerequisites for the molecular evolution required for the development of a tumor. Perhaps most notable amongst these is the p53 tumor suppressor gene, which is mutated in >50% of human tumors. Thus, the importance of the checkpoint pathways to human biology is clear.

References

Edit history

Date	Action	Author
2005-01-01	Authored	Walworth N, Hoffmann I, Yen TJ, O'Donnell M, Khanna KK
2005-01-01	Created	Walworth N, Hoffmann I, Yen TJ, O'Donnell M, Khanna KK
2013-11-25	Edited	Matthews L
2022-03-10	Reviewed	Sanchez Y, Knudsen E, Hardwick KG
2022-03-23	Modified	Weiser JD

52 submitted entities found in this pathway, mapping to 54 Reactome entities

Input	UniProt Id	Input	UniProt Id	Input	UniProt Id
ENSG00000006634	Q9UBU7	ENSG00000072401	P51668	ENSG00000073111	P49736
ENSG00000088986	P63167	ENSG00000089685	O15392	ENSG00000092010	Q06323
ENSG00000092853	Q9HAW4	ENSG00000093009	O75419	ENSG00000094804	Q99741
ENSG00000100401	P46060	ENSG00000100567	P25788	ENSG00000105173	P24864
ENSG00000108671	O00231	ENSG00000113575	P67775	ENSG00000117724	P49454
ENSG00000120334	Q8N0S6	ENSG00000122952	O95229	ENSG00000124635	P06899
ENSG00000124762	P38936	ENSG00000126067	P49721	ENSG00000129810	Q5FBB7
ENSG00000134308	P27348	ENSG00000134690	Q53HL2	ENSG00000136492	Q9BX63
ENSG00000137812	Q8NG31	ENSG00000138346	P51530	ENSG00000142507	P28072
ENSG00000142945	Q99661	ENSG00000143106	P28066	ENSG00000143228	Q9BZD4
ENSG00000145386	P20248	ENSG00000147889	Q8N726	ENSG00000152253	Q9HBM1
ENSG00000158402	P30307	ENSG00000163535	Q562F6	ENSG00000163636	Q15008
ENSG00000163918	P35249, P35250	ENSG00000163961	Q8IYW5	ENSG00000164045	P30304
ENSG00000164109	Q13257	ENSG00000164924	P63104	ENSG00000170312	P06493
ENSG00000173692	Q99460	ENSG00000175643	Q96E14	ENSG00000175793	P31947
ENSG00000177889	P61088	ENSG00000182628	Q8WVK7	ENSG00000186871	Q2NKX8
ENSG00000188486	P16104	ENSG00000197061	P62805	ENSG00000197238	P62805
ENSG00000278588	P62807				

Input	Ensembl Id
ENSG00000124762	ENSG00000124762

20. Interleukin-10 signaling (R-HSA-6783783)

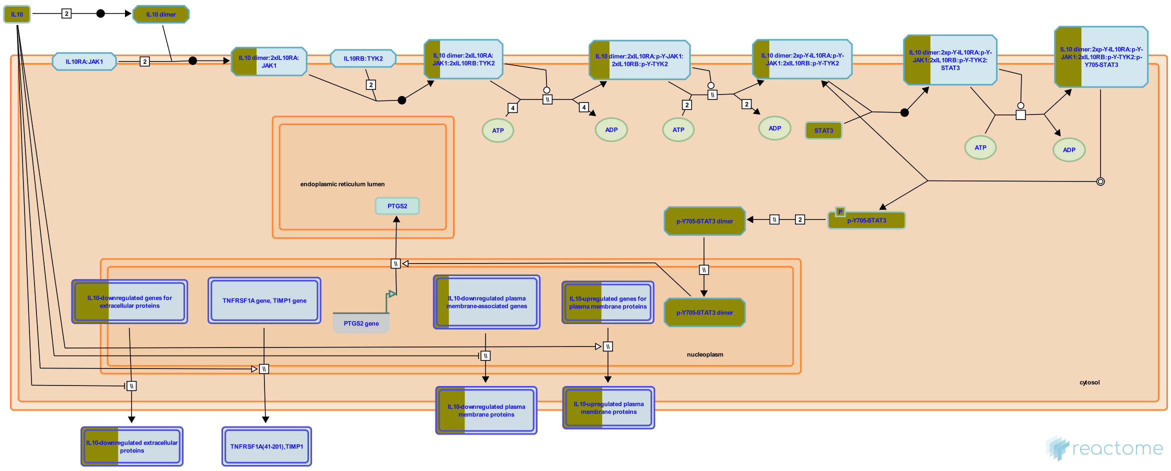

Interleukin-10 (IL10) was originally described as a factor named cytokine synthesis inhibitory factor that inhibited T-helper (Th) 1 activation and Th1 cytokine production (Fiorentino et al. 1989). It was found to be expressed by a variety of cell types including macrophages, dendritic cell subsets, B cells, several T-cell subpopulations including Th2 and T-regulatory cells (Tregs) and Natural Killer (NK) cells (Moore et al. 2001). It is now recognized that the biological effects of IL10 are directed at antigen-presenting cells (APCs) such as macrophages and dendritic cells (DCs), its effects on T-cell development and differentiation are largely indirect via inhibition of macrophage/dendritic cell activation and maturation (Pestka et al. 2004, Mocellin et al. 2004). T cells are thought to be the main source of IL10 (Hedrich & Bream 2010). IL10 inhibits a broad spectrum of activated macrophage/monocyte functions including monokine synthesis, NO production, and expression of class II MHC and costimulatory molecules such as IL12 and CD80/CD86 (de Waal Malefyt et al. 1991, Gazzinelli et al. 1992). Studies with recombinant cytokine and neutralizing antibodies revealed pleiotropic activities of IL10 on B, T, and mast cells (de Waal Malefyt et al. 1993, Rousset et al. 1992, Thompson-Snipes et al. 1991) and provided evidence for the in vivo significance of IL10 activities (Ishida et al. 1992, 1993). IL10 antagonizes the expression of MHC class II and the co-stimulatory molecules CD80/CD86 as well as the pro-inflammatory cytokines IL1Beta, IL6, IL8, TNFalpha and especially IL12 (Fiorentino et al. 1991, D'Andrea et al. 1993). The biological role of IL10 is not limited to inactivation of APCs, it also enhances B cell, granulocyte, mast cell, and keratinocyte growth/differentiation, as well as NK-cell and CD8+ cytotoxic T-cell activation (Moore et al. 2001, Hedrich & Bream 2010). IL10 also enhances NK-cell proliferation and/or production of IFN-gamma (Cai et al. 1999).

IL10-deficient mice exhibited inflammatory bowel disease (IBD) and other exaggerated inflammatory responses (Kuhn et al. 1993, Berg et al. 1995) indicating a critical role for IL10 in limiting inflammatory responses. Dysregulation of IL10 is linked with susceptibility to numerous infectious and autoimmune diseases in humans and mouse models (Hedrich & Bream 2010).

IL10 signaling is initiated by binding of homodimeric IL10 to the extracellular domains of two adjoining IL10RA molecules. This tetramer then binds two IL10RB chains. IL10RB cannot bind to IL10 unless bound to IL10RA (Ding et al. 2001, Yoon et al. 2006); binding of IL10 to IL10RA without the co-presence of IL10RB fails to initiate signal transduction (Kotenko et al. 1997).

IL10 binding activates the receptor-associated Janus tyrosine kinases, JAK1 and TYK2, which are constitutively bound to IL10R1 and IL10R2 respectively. In the classic model of receptor activation assembly of the receptor complex is believed to enable JAK1/TYK2 to phosphorylate and activate each other. Alternatively the binding of IL10 may cause conformational changes that allow the pseudokinase inhibitory domain of one JAK kinase to move away from the kinase domain of the other JAK within the receptor dimer-JAK complex, allowing the two kinase domains to interact and trans-activate (Waters & Brooks 2015).

The activated JAK kinases phosphorylate the intracellular domains of the IL10R1 chains on specific tyrosine residues. These phosphorylated tyrosine residues and their flanking peptide sequences serve as temporary docking sites for the latent, cytosolic, transcription factor, STAT3. STAT3 transiently docks on the IL10R1 chain via its SH2 domain, and is in turn tyrosine phosphorylated by the receptor-associated JAKs. Once activated, it dissociates from the receptor, dimerizes with other STAT3 molecules, and translocates to the nucleus where it binds with high affinity to STAT-binding elements (SBEs) in the promoters of IL-10-inducible genes (Donnelly et al. 1999).

References

Moore KW, O'Garra A, Coffman RL & de Waal Malefyt R (2001). Interleukin-10 and the interleukin-10 receptor. Annu. Rev. Immunol., 19, 683-765.

Edit history

Date	Action	Author
2015-06-17	Authored	Jupe S
2015-06-17	Created	Jupe S
2016-09-05	Reviewed	Meldal BH
2016-11-14	Edited	Jupe S
2022-03-30	Modified	Weiser JD

16 submitted entities found in this pathway, mapping to 29 Reactome entities

Input	UniProt Id	Input	UniProt Id	Input	UniProt Id
ENSG00000108688	P13500	ENSG00000108691	P13500	ENSG00000113302	P29460
ENSG00000114013	P33681, P42081	ENSG00000136634	P22301	ENSG00000136689	P18510
ENSG00000160791	P51681	ENSG00000163734	P09341	ENSG00000163739	P09341
ENSG00000168610	P40763	ENSG00000169245	P02778	ENSG00000171049	P21462
ENSG00000171051	P21462	ENSG00000172724	Q99731	ENSG00000271503	P13501
ENSG00000277632	P10147, P16619				

Input	Ensembl Id	Input	Ensembl Id	Input	Ensembl Id
ENSG00000108691	ENSG00000108691	ENSG00000113302	ENSG00000113302	ENSG00000114013	ENSG00000114013
ENSG00000136689	ENSG00000136689	ENSG00000160791	ENSG00000160791	ENSG00000163739	ENSG00000163739
ENSG00000169245	ENSG00000169245	ENSG00000171051	ENSG00000171051	ENSG00000172724	ENSG00000172724
ENSG00000271503	ENSG00000271503	ENSG00000277632	ENSG00000277632		

21. Chemokine receptors bind chemokines (R-HSA-380108)

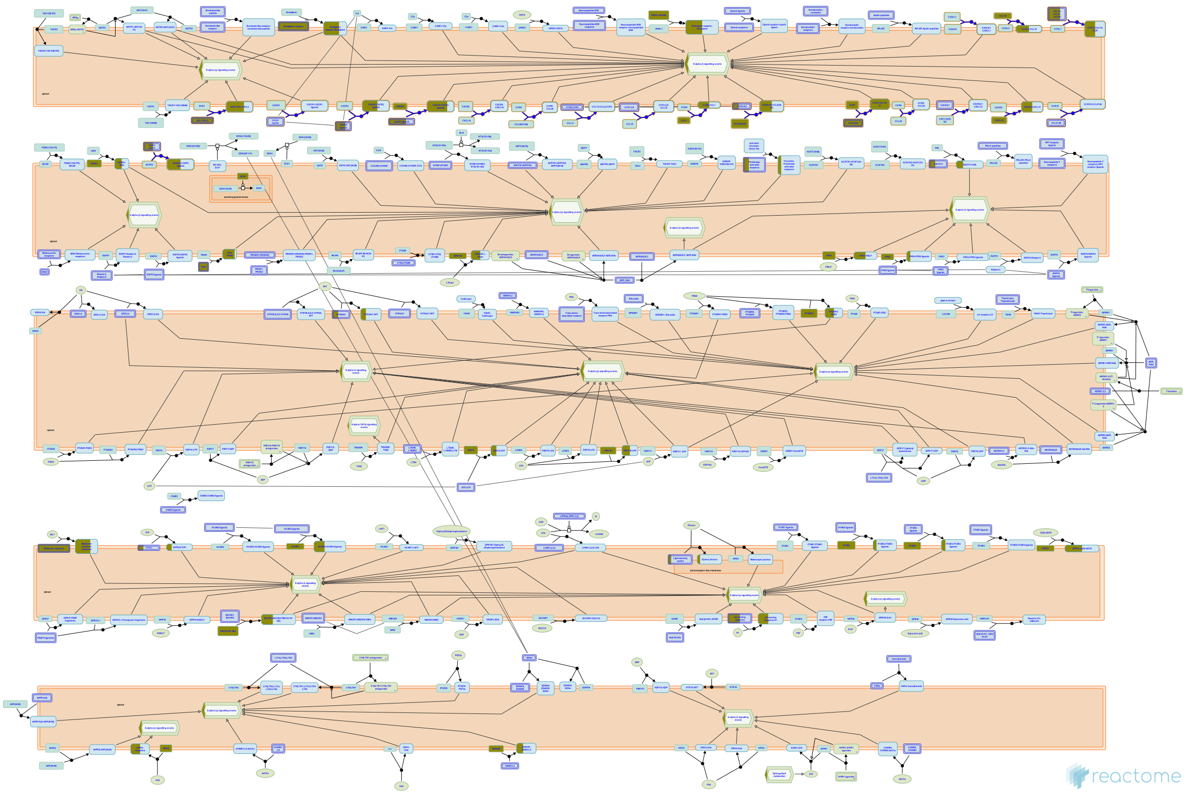

Chemokine receptors are cytokine receptors found on the surface of certain cells, which interact with a type of cytokine called a chemokine. Following interaction, these receptors trigger a flux of intracellular calcium which leads to chemotaxis. Chemokine receptors are divided into different families, CXC chemokine receptors, CC chemokine receptors, CX3C chemokine receptors and XC chemokine receptors that correspond to the 4 distinct subfamilies of chemokines they bind.

References

Finn A & Murdoch C (2000). Chemokine receptors and their role in inflammation and infectious diseases. Blood, 95, 3032-43.

Kim CH (2004). Chemokine-chemokine receptor network in immune cell trafficking. Curr Drug Targets Immune Endocr Metabol Disord, 4, 343-61.

Horuk R (2001). Chemokine receptors. Cytokine Growth Factor Rev, 12, 313-35.

Edit history

Date	Action	Author
2008-11-07	Authored	Jassal B
2008-11-07	Created	Jassal B
2022-03-23	Modified	Weiser JD

18 submitted entities found in this pathway, mapping to 25 Reactome entities

Input	UniProt Id	Input	UniProt Id	Input	UniProt Id
ENSG00000108688	P13500, P80098	ENSG00000108691	P13500	ENSG00000108702	O00585, P22362
ENSG00000126353	P32248	ENSG00000143184	P47992, Q9UBD3	ENSG00000143185	Q9UBD3

Input	UniProt Id	Input	UniProt Id	Input	UniProt Id
ENSG00000156234	O43927, P02775	ENSG00000160791	P51681	ENSG00000163734	P09341, P19876
ENSG00000163735	P42830	ENSG00000163736	P02775, P02776	ENSG00000163739	P09341
ENSG00000169245	P02778	ENSG00000169248	O14625	ENSG00000172724	Q99731
ENSG00000186810	P49682	ENSG00000271503	P13501	ENSG00000277632	P10147, P16619

22. SARS-CoV-2-host interactions (R-HSA-9705683)

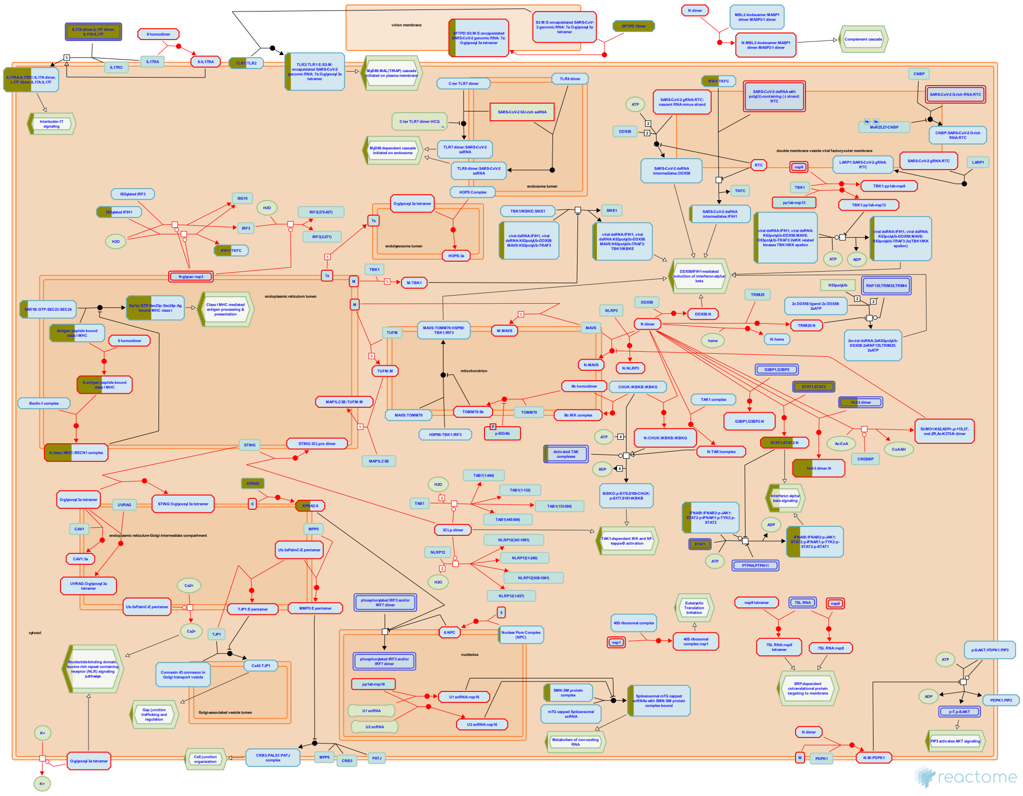

Diseases: COVID-19.

Coronaviruses are a group of enveloped viruses with singlestranded, positivesense RNA genomes. Each of the steps of viral replication - attachment and entry, translation of viral replicase, genome transcription and replication, translation of structural proteins, and virion assembly and release - involves host factors. These interactions can cause alterations in cellular structure and physiology, and activate host stress responses, autophagy, cell death, and processes of innate immunity (Fung TS & Liu DX 2019). This Reactome module describes molecular mechanisms by which severe acute respiratory syndrome coronavirus type 2 (SARS-CoV-2) modulates innate and adaptive immune responses, autophagy, host translation, intracellular signaling and regulatory pathways, and PDZ-mediated cell-cell junctions, mostly annotated from studies of cells infected with SARS-CoV-2.

References

Jansen S, Daelemans D, Vanstreels E & Baggen J (2021). Cellular host factors for SARS-CoV-2 infection. Nat Microbiol, 6, 1219-1232.

Agelidis A, Koganti R, Suryawanshi RK, Shukla D & Patil CD (2021). Dysregulation of Cell Signaling by SARS-CoV-2. Trends Microbiol, 29, 224-237.

Lowery SA, Perlman S & Sariol A (2021). Innate immune and inflammatory responses to SARS-CoV-2: Implications for COVID-19. Cell Host Microbe, 29, 1052-1062.

Forero A & Beyer DK (2021). Mechanisms of Antiviral Immune Evasion of SARS-CoV-2. J Mol Biol, 167265.

Fung TS & Liu DX (2019). Human Coronavirus: Host-Pathogen Interaction. Annu. Rev. Microbiol., 73, 529-557.

Edit history

Date	Action	Author
2020-10-28	Created	Shamovsky V
2021-05-03	Authored	Stephan R
2021-10-26	Authored	Shamovsky V
2022-02-17	Edited	Shamovsky V
2022-02-17	Reviewed	Messina F
2022-03-01	Reviewed	Gillespie ME
2022-03-16	Modified	Weiser JD

20 submitted entities found in this pathway, mapping to 56 Reactome entities

Input	UniProt Id	Input	UniProt Id	Input	UniProt Id
ENSG00000093000	Q9UKX7	ENSG00000112115	Q16552	ENSG00000112116	Q96PD4
ENSG00000113615	O95486	ENSG00000115267	Q9BYX4	ENSG00000115415	P42224, P42224-1, P42224-2
ENSG00000133661	P35247	ENSG00000134070	O43187	ENSG00000134308	P27348
ENSG00000137462	O60603	ENSG00000143977	P62308	ENSG00000164924	P63104
ENSG00000167088	P62314	ENSG00000167207	Q9HC29	ENSG00000170581	P52630
ENSG00000175793	P31947	ENSG00000177889	P61088	ENSG00000182481	P52292
ENSG00000233816	P01562	ENSG00000234745	P01889, P03989, P10319, P18463, P18464, P18465, P30460, P30461, P30462, P30464, P30466, P30475, P30479, P30480, P30481, P30483, P30484, P30485, P30486, P30487, P30488, P30490, P30491, P30492, P30493, P30495, P30498, P30685, Q04826, Q29718, Q29836, Q29940, Q31610, Q31612, Q95365		

23. G1/S Transition (R-HSA-69206)

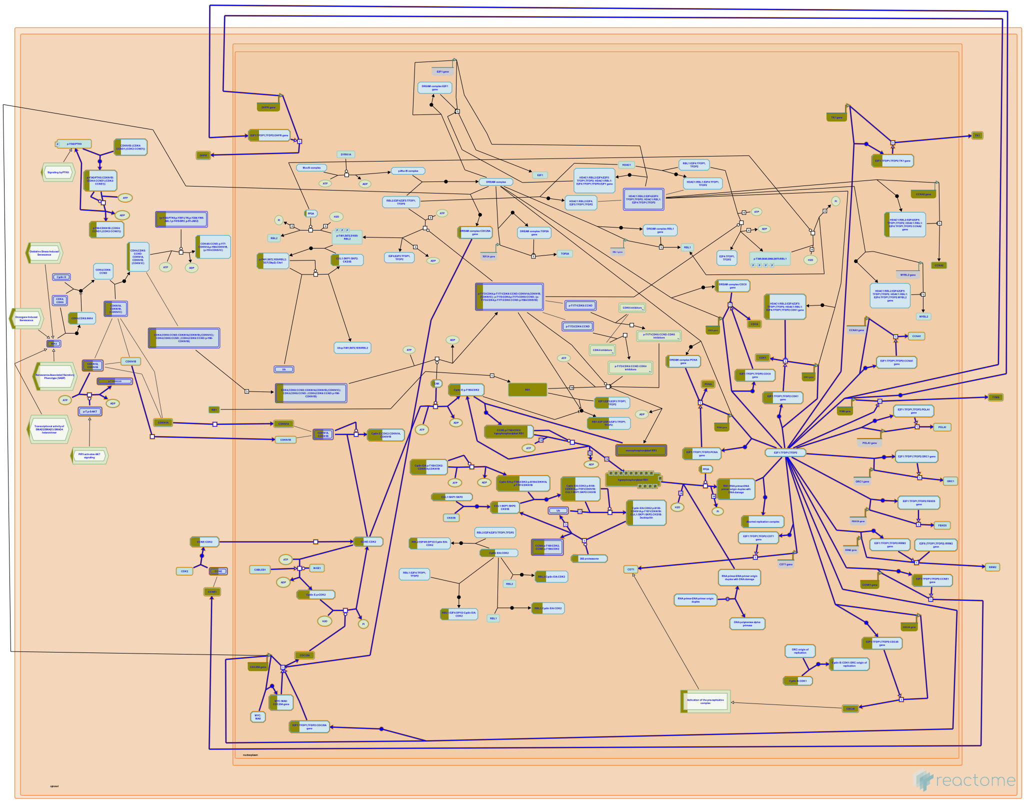

Cyclin E - Cdk2 complexes control the transition from G1 into S-phase. In this case, the binding of p21Cip1/Waf1 or p27kip1 is inhibitory. Important substrates for Cyclin E - Cdk2 complexes include proteins involved in the initiation of DNA replication. The two Cyclin E proteins are subjected to ubiquitin-dependent proteolysis, under the control of an E3 ubiquitin ligase known as the SCF. Cyclin A - Cdk2 complexes, which are also regulated by p21Cip1/Waf1 and p27kip1, are likely to be important for continued DNA synthesis, and progression into G2. An additional level of control of Cdk2 is reversible phosphorylation of Threonine-14 (T14) and Tyrosine-15 (Y15), catalyzed by the Wee1 and Myt1 kinases, and dephosphorylation by the three Cdc25 phosphatases, Cdc25A, B and C.

References

Edit history

Date	Action	Author
2003-06-05	Created	Walworth N, O'Donnell M
2022-03-23	Modified	Weiser JD

26 submitted entities found in this pathway, mapping to 35 Reactome entities

Input	UniProt Id	Input	UniProt Id	Input	UniProt Id
ENSG00000006634	Q9UBU7	ENSG00000073111	P49736	ENSG00000092010	Q06323
ENSG00000093009	O75419	ENSG00000094804	Q99741	ENSG00000100479	P56282
ENSG00000100567	P25788	ENSG00000105173	P24864	ENSG00000108671	O00231
ENSG00000108702	P51946	ENSG00000113575	P67775	ENSG00000124762	P38936
ENSG00000126067	P49721	ENSG00000132646	P12004	ENSG00000139687	P06400
ENSG00000142507	P28072	ENSG00000143106	P28066	ENSG00000145386	P20248
ENSG00000145604	Q13309	ENSG00000163636	Q15008	ENSG00000164045	P30304

Input	UniProt Id
ENSG00000167900	P04183
ENSG00000176890	P04818

Input	UniProt Id
ENSG00000170312	P06493
ENSG00000228716	P00374

Input	UniProt Id
ENSG00000173692	Q99460

Input	Ensembl Id
ENSG00000093009	ENSG00000093009
ENSG00000132646	ENSG00000132646
ENSG00000170312	ENSG00000170312

Input	Ensembl Id
ENSG00000094804	ENSG00000094804
ENSG00000164045	ENSG00000164045
ENSG00000176890	ENSG00000176890

Input	Ensembl Id
ENSG00000105173	ENSG00000105173
ENSG00000167900	ENSG00000167900
ENSG00000228716	ENSG00000228716

24. Formation of the cornified envelope (R-HSA-6809371)

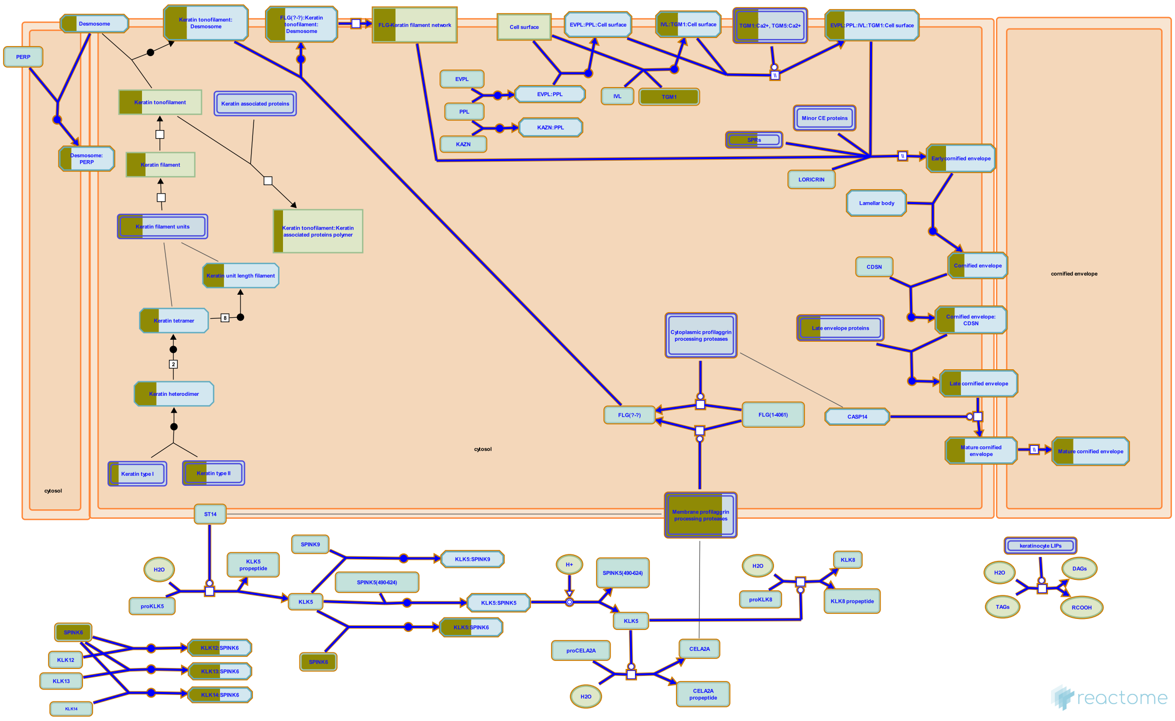

As keratinocytes progress towards the upper epidermis, they undergo a unique process of cell death termed cornification (Eckhart et al. 2013). This involves the crosslinking of keratinocyte proteins such as loricrin and involucrin by transglutaminases and the breakdown of the nucleus and other organelles by intracellular and secreted proteases (Eckhart et al. 2000, Denecker et al. 2008). This process is strictly regulated by the Ca2+ concentration gradient in the epidermis (Esholtz et al. 2014). Loricrin and involucrin are encoded in ‘Epidermal Differentiation Complex’ linked to a large number of genes encoding nonredundant components of the CE (Kypriotou et al. 2012, Niehues et al. 2016). Keratinocytes produce specialized proteins and lipids which are used to construct the cornified envelope (CE), a heavily crosslinked submembranous layer that confers rigidity to the upper epidermis, allows keratin filaments to attach to any location in the cell membrane (Kirfel et al. 2003) and acts as a water-impermeable barrier. The CE has two functional parts: covalently crosslinked proteins (10 nm thick) that comprise the backbone of the envelope and covalently linked lipids (5 nm thick) that coat the exterior (Eckert et al. 2005). Desmosomal components are crosslinked to the CE to form corneodesmosomes, which bind cornified cells together (Ishida-Yamamoto et al. 2011). Mature terminally differentiated cornified cells consist mostly of keratin filaments covalently attached to the CE embedded in lipid lamellae (Kalinin et al. 2002). The exact composition of the cornified envelope varies between epithelia (Steinert et al. 1998); the relative amino-acid composition of the proteins used may determine differential mechanical properties (Kartasova et al. 1996).

References

Candi E, Melino G & Schmidt R (2005). The cornified envelope: a model of cell death in the skin. Nat. Rev. Mol. Cell Biol., 6, 328-40.

Edit history

Date	Action	Author
2015-11-11	Created	Jupe S
2016-03-10	Authored	Jupe S
2016-08-10	Edited	Jupe S
2016-08-12	Reviewed	Blumenberg M
2022-03-23	Modified	Weiser JD

29 submitted entities found in this pathway, mapping to 35 Reactome entities

Input	UniProt Id	Input	UniProt Id	Input	UniProt Id
ENSG00000052344	Q16651	ENSG00000092295	P22735	ENSG00000134755	Q02487
ENSG00000134757	P32926	ENSG00000140479	P29122	ENSG00000140564	P09958
ENSG00000159516	Q9BYE4	ENSG00000167916	P08727, Q2M2I5	ENSG00000170423	Q8N1N4
ENSG00000170454	O95678	ENSG00000170465	P04259, P48668	ENSG00000170477	P19013
ENSG00000170486	Q14CN4	ENSG00000173801	P14923	ENSG00000178172	Q6UWN8
ENSG00000185069	Q01546	ENSG00000185962	Q5TA76	ENSG00000185966	Q5T5B0
ENSG00000186442	P12035	ENSG00000186832	P08779	ENSG00000186847	P02533, P08779, Q04695
ENSG00000187173	Q5TA79	ENSG00000187238	Q5TA77	ENSG00000196805	P35325
ENSG00000203785	P22531	ENSG00000205420	P02538, P04264, P48668	ENSG00000241794	P35326
ENSG00000244057	Q5T5A8	ENSG00000244094	Q96RM1		

25. G1/S-Specific Transcription (R-HSA-69205)

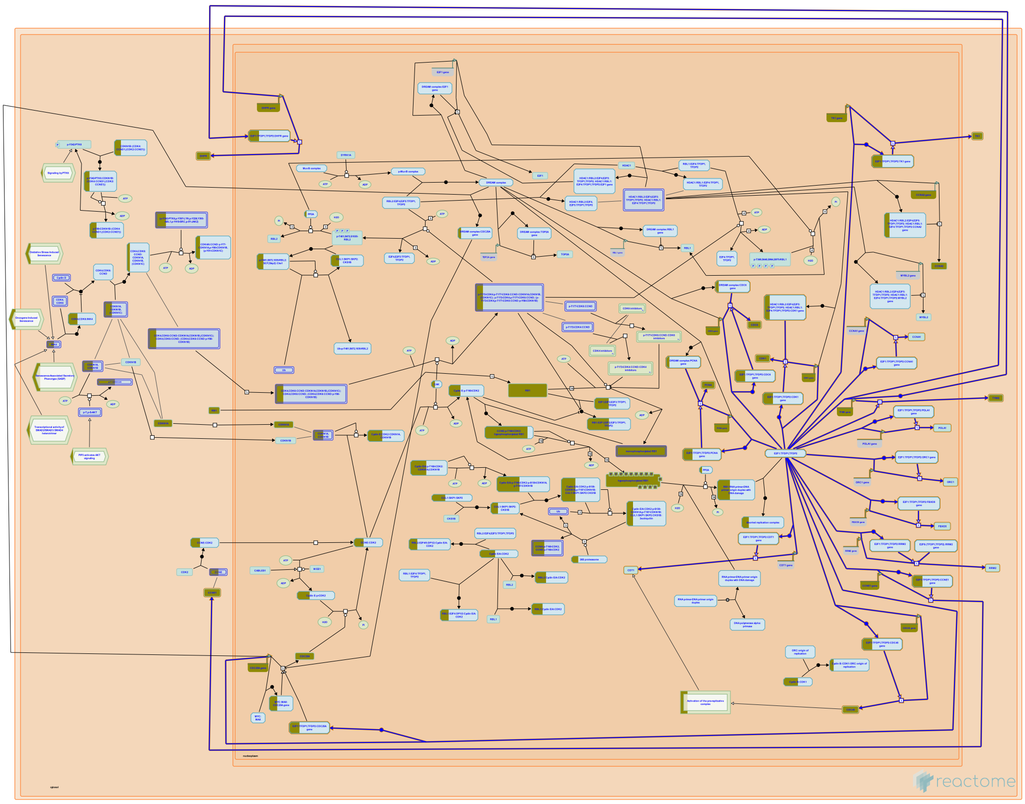

Cellular compartments: nucleoplasm.

The E2F family of transcription factors regulate the transition from the G1 to the S phase in the cell cycle. E2F activity is regulated by members of the retinoblastoma protein (pRb) family, resulting in the tight control of the expression of E2F-responsive genes. Phosphorylation of pRb by cyclin D:CDK complexes releases pRb from E2F, inducing E2F-targeted genes such as cyclin E.

E2F1 binds to E2F binding sites on the genome activating the synthesis of the target proteins. For annotation purposes, the reactions regulated by E2F1 are grouped under this pathway and information about the target genes alone are displayed for annotation purposes.

Cellular targets for activation by E2F1 include thymidylate synthase (TYMS) (DeGregori et al. 1995), Rir2 (RRM2) (DeGregori et al. 1995, Giangrande et al. 2004), Dihydrofolate reductase (DHFR) (DeGregori et al. 1995, Wells et al. 1997, Darbinian et al. 1999), Cdc2 (CDK1) (Furukawa et al. 1994, DeGregori et al. 1995, Zhu et al. 2004), Cyclin A1 (CCNA1) (DeGregori et al. 1995, Liu et al. 1998), CDC6 (DeGregori et al. 1995, Yan et al. 1998; Ohtani et al. 1998), CDT1 (Yoshida and Inoue 2004), CDC45 (Arata et al. 2000), Cyclin E (CCNE1) (Ohtani et al. 1995), Emi1 (FBXO5) (Hsu et al. 2002), and ORC1 (Ohtani et al. 1996, Ohtani et al. 1998). The activation of TK1 (Dnk1) (Dou et al. 1994, DeGregori et al. 1995, Giangrande et al. 2004) and CDC25A (DeGregori et al. 1995, Vigo et al. 1999) by E2F1 is conserved in Drosophila (Duronio and O'Farrell 1994, Reis and Edgar 2004).

RRM2 protein is involved in dNTP level regulation and activation of this enzyme results in higher levels of dNTPs in anticipation of S phase. E2F activation of RRM2 has been shown also in Drosophila by Duronio and O'Farrell (1994). E2F1 activation of CDC45 is shown in mouse cells by using human E2F1 construct (Arata et al. 2000). Cyclin E is also transcriptionally regulated by E2F1. Cyclin E protein plays important role in the transition of G1 in S phase by associating with CDK2 (Ohtani et al. 1996). E2F1-mediated activation of PCNA has been demonstrated in Drosophila (Duronio and O'Farrell 1994) and in some human cells by using recombinant adenovirus constructs (DeGregori et al. 1995). E2F1-mediated activation of the DNA polymerase alpha subunit p180 (POLA1) has been demonstrated in some human cells. It has also been demonstrated in Drosophila by Ohtani and Nevins (1994). It has been observed in Drosophila that E2F1 induced expression of Orc1 stimulates ORC1 6 complex formation and binding to the origin of replication (Asano and Wharton 1999). ORC1 6 recruit CDC6 and CDT1 that are required to recruit the MCM2 7 replication helicases. E2F1 regulation incorporates a feedback mechanism wherein Geminin (GMNN) can inhibit MCM2 7 recruitment of ORC1 6 complex by interacting with CDC6/CDT1. The activation of CDC25A and TK1 (Dnk1) by E2F1 has been inferred from similar events in Drosophila (Duronio RJ and O'Farrell 1994; Reis and Edgar 2004). E2F1 activates string (CDC25) that in turn activates the complex of Cyclin B and CDK1. A similar phenomenon has been observed in mouse NIH 3T3 cells and in Rat1 cells.

References

Ohtani K, Ikeda M, Nakamura M & Tsujimoto A (1998). Regulation of cell growth-dependent expression of mammalian CDC6 gene by the cell cycle transcription factor E2F. Oncogene, 17, 1777-85.

Wang J, Dou QP, Pardee AB, Zhao S, Helin K & Levin AH (1994). G1/S-regulated E2F-containing protein complexes bind to the mouse thymidine kinase gene promoter. J. Biol. Chem., 269, 1306-13.

Hateboer G, Vigo E, Helin K, Prosperini E, Cartwright P, Moroni MC & Muller H (1999). CDC25A phosphatase is a target of E2F and is required for efficient E2F-induced S phase. Mol Cell Biol, 19, 6379-95.

Tretiakova A, Kundu M, Gallia GL, Khalili K, Giordano A, Shcherbik N & Darbinian N (1999). Association of Pur alpha and E2F-1 suppresses transcriptional activity of E2F-1. Oncogene, 18, 6398-402.

Kijima S, Ohtani K, Fujita M, Arata Y & Kato JY (2000). Cdk2-dependent and -independent pathways in E2F-mediated S phase induction. J Biol Chem, 275, 6337-45.

Edit history

Date	Action	Author
2003-06-05	Created	Walworth N, O'Donnell M
2018-12-21	Modified	D'Eustachio P

9 submitted entities found in this pathway, mapping to 17 Reactome entities

Input	UniProt Id	Input	UniProt Id	Input	UniProt Id
ENSG00000093009	O75419	ENSG00000094804	Q99741	ENSG00000105173	P24864
ENSG00000132646	P12004	ENSG00000167900	P04183	ENSG00000170312	P06493
ENSG00000176890	P04818	ENSG00000228716	P00374		

Input	Ensembl Id	Input	Ensembl Id	Input	Ensembl Id
ENSG00000093009	ENSG00000093009	ENSG00000094804	ENSG00000094804	ENSG00000105173	ENSG00000105173
ENSG00000132646	ENSG00000132646	ENSG00000164045	ENSG00000164045	ENSG00000167900	ENSG00000167900
ENSG00000170312	ENSG00000170312	ENSG00000176890	ENSG00000176890	ENSG00000228716	ENSG00000228716

6. Identifiers found

Below is a list of the input identifiers that have been found or mapped to an equivalent element in Reactome, classified by resource.

903 of the submitted entities were found, mapping to 1128 Reactome entities

Input	UniProt Id	Input	UniProt Id	Input	UniProt Id
ENSG00000002587	O14792	ENSG00000003400	Q92851	ENSG00000004455	P54819
ENSG00000004468	P28907	ENSG00000004864	Q9UJS0	ENSG00000005022	P05141
ENSG00000005059	Q9NWR8	ENSG00000005469	Q9UKG9	ENSG00000005844	P20701
ENSG00000006327	Q9NP84	ENSG00000006625	O75223	ENSG00000006634	Q9UBU7
ENSG00000006695	Q12887	ENSG00000006831	Q86V24	ENSG00000007038	Q9Y6M0
ENSG00000007171	P35228	ENSG00000007908	P16581	ENSG00000007933	P31513
ENSG00000007968	Q14209	ENSG00000008311	Q9UDR5	ENSG00000008516	Q9NPA2
ENSG00000008517	P24001	ENSG00000010932	Q01740	ENSG00000011009	O95372
ENSG00000021355	P30740	ENSG00000023330	P13196	ENSG00000023445	Q13489, Q13490
ENSG00000023839	Q92887	ENSG00000037241	Q9UNX3	ENSG00000037897	Q9UBP6
ENSG00000047365	Q8WZ64	ENSG00000049249	Q07011	ENSG00000049768	Q9BZS1
ENSG00000050438	Q2Y0W8	ENSG00000050628	P43115	ENSG00000050730	Q96KP6
ENSG00000051128	Q9NSC5	ENSG00000051180	Q06609	ENSG00000051341	O75417
ENSG00000051596	Q96J01	ENSG00000052344	Q16651	ENSG00000055044	Q9Y2X3
ENSG00000055332	P19525	ENSG00000056558	Q13077	ENSG00000057149	P29508
ENSG00000057657	O75626	ENSG00000058063	Q9Y2G3	ENSG00000060339	Q8IX12
ENSG00000064102	Q9NVM9	ENSG00000070501	P06746	ENSG00000072041	Q9H2J7
ENSG00000072401	P51668	ENSG00000072571	O75330	ENSG00000072803	Q9UKB1
ENSG00000073111	P49736	ENSG00000078589	O00398	ENSG00000078900	O15350
ENSG00000079112	Q12864	ENSG00000083454	Q93086	ENSG00000087088	Q07812
ENSG00000087269	P78316	ENSG00000087586	O14965	ENSG00000088002	Q06520
ENSG00000088986	P63167	ENSG00000089195	Q9UJA5	ENSG00000089225	Q99593
ENSG00000089685	O15392	ENSG00000089692	P18627	ENSG00000089723	Q96DC9
ENSG00000089820	P98171	ENSG00000089902	Q9UKL0	ENSG00000090061	O75909
ENSG00000090104	Q08116	ENSG00000090376	Q9Y616	ENSG00000090382	P61626
ENSG00000090520	Q9UBS4	ENSG00000092010	Q06323	ENSG00000092295	P22735
ENSG00000092853	Q9HAW4	ENSG00000092929	Q70J99	ENSG00000093000	Q9UKX7
ENSG00000093009	O75419	ENSG00000093134	Q9NY84	ENSG00000094804	Q99741
ENSG00000094963	Q99518	ENSG00000095585	Q8WV28	ENSG00000095587	Q9Y6L7
ENSG00000095596	O43174	ENSG00000100228	O95755	ENSG00000100280	Q10567
ENSG00000100292	P09601	ENSG00000100342	O14791	ENSG00000100346	Q9P0X4
ENSG00000100368	P32927	ENSG00000100401	P46060	ENSG00000100433	P57789
ENSG00000100450	P20718	ENSG00000100453	P08311	ENSG00000100479	P56282
ENSG00000100504	P06737	ENSG00000100522	Q96EK6	ENSG00000100554	Q9Y5K8
ENSG00000100567	P25788	ENSG00000100596	O15270	ENSG00000100644	Q16665
ENSG00000101361	O00567	ENSG00000101670	Q9Y5X9	ENSG00000101811	P33240
ENSG00000101846	P08842	ENSG00000101850	P51810	ENSG00000101871	O15344
ENSG00000102144	P00558	ENSG00000103044	O00219	ENSG00000103056	Q9NY59
ENSG00000103067	Q9H6T0	ENSG00000103121	Q9NRP2	ENSG00000103187	Q14019
ENSG00000103222	P33527	ENSG00000103257	Q01650	ENSG00000103313	O15553

Input	UniProt Id	Input	UniProt Id	Input	UniProt Id
ENSG00000103326	O75808	ENSG00000103342	P15170	ENSG00000103415	P30519
ENSG00000103449	Q9NSC2	ENSG00000103490	Q9ULZ3	ENSG00000103522	Q9HBE5
ENSG00000103653	P41240	ENSG00000103888	Q8WUJ3	ENSG00000104131	O75822
ENSG00000104147	O43482	ENSG00000104356	Q99575	ENSG00000104885	Q8TEK3
ENSG00000104951	Q96RQ9	ENSG00000104972	Q8NHL6	ENSG00000104974	O75019
ENSG00000105173	P24864	ENSG00000105246	Q14213	ENSG00000105376	Q9UMF0
ENSG00000105388	P06731	ENSG00000105641	Q92911	ENSG00000105642	Q92952
ENSG00000105664	P49747	ENSG00000108242	P33261	ENSG00000108671	O00231
ENSG00000108679	Q08380	ENSG00000108688	P13500	ENSG00000108691	P13500
ENSG00000108702	P51946	ENSG00000108771	Q96C10	ENSG00000108786	P14061
ENSG00000108826	Q9P0M9	ENSG00000108950	Q96MK3	ENSG00000109079	Q13829
ENSG00000109158	P48169	ENSG00000109163	P30968	ENSG00000109193	P49888
ENSG00000109320	P19838	ENSG00000109321	P15514	ENSG00000109971	P11142
ENSG00000110680	P01258, P06881, P10092	ENSG00000110697	O00562	ENSG00000110876	Q14242
ENSG00000110934	Q9UBW5	ENSG00000110944	Q9NPF7	ENSG00000111012	O15528
ENSG00000111049	P13349	ENSG00000111206	Q08050	ENSG00000111229	O15145
ENSG00000111247	Q96B01	ENSG00000111275	P05091	ENSG00000111321	P36941
ENSG00000111331	Q9Y6K5	ENSG00000111335	P29728	ENSG00000111344	O95294
ENSG00000111639	Q4U2R6	ENSG00000111640	P04406	ENSG00000111641	P46087
ENSG00000111796	Q12918	ENSG00000111802	O95551	ENSG00000111816	P42685
ENSG00000112096	P04179	ENSG00000112115	Q16552	ENSG00000112116	Q96PD4
ENSG00000112146	Q9UK97	ENSG00000112195	Q5T2D2	ENSG00000112242	O00716
ENSG00000112249	Q8N3C0	ENSG00000112294	P51649	ENSG00000112299	O95497
ENSG00000112303	O95498	ENSG00000113300	Q9ULM6	ENSG00000113302	P29460
ENSG00000113356	O15318	ENSG00000113368	P20700	ENSG00000113396	Q9Y2P4
ENSG00000113407	P26639	ENSG00000113532	Q92187	ENSG00000113575	P67775
ENSG00000113615	O95486	ENSG00000113643	P54136	ENSG00000113712	P48729
ENSG00000113749	P25021	ENSG00000113763	Q6ZN44	ENSG00000113810	Q9NTJ3
ENSG00000114013	P33681, P42081	ENSG00000114251	P41221	ENSG00000114346	Q9H8V3
ENSG00000114354	Q92734	ENSG00000114738	Q16644	ENSG00000114767	O43818
ENSG00000115085	P43403	ENSG00000115091	P61158	ENSG00000115129	Q53FA7
ENSG00000115221	P18564	ENSG00000115232	P13612	ENSG00000115267	Q9BYX4
ENSG00000115392	Q9NW38	ENSG00000115415	P42224, P42224-1, P42224-2	ENSG00000115488	Q9Y3R4
ENSG00000115523	P22749	ENSG00000115541	P61604	ENSG00000115556	Q9BRC7
ENSG00000115607	O95256	ENSG00000115657	Q9NP58	ENSG00000115750	Q53T94
ENSG00000115808	O43815	ENSG00000116260	O00391	ENSG00000116663	Q9NRD1
ENSG00000116701	P19878	ENSG00000117632	P16949	ENSG00000117650	P51955
ENSG00000117724	P49454	ENSG00000117971	P30926	ENSG00000117984	P07339
ENSG00000118162	Q9Y664	ENSG00000118985	O00472	ENSG00000119508	Q92570
ENSG00000119535	Q99062	ENSG00000120217	Q9NZQ7	ENSG00000120254	Q6UB35
ENSG00000120334	Q8N0S6	ENSG00000120337	Q9UNG2	ENSG00000120436	O00270
ENSG00000120539	Q96GX5	ENSG00000120694	Q92598	ENSG00000120738	P18146
ENSG00000122223	Q9BZW8	ENSG00000122257	Q7Z6E9	ENSG00000122861	P00749
ENSG00000122862	P10124	ENSG00000122952	O95229	ENSG00000122966	O14578, O14578-3
ENSG00000123136	O00148	ENSG00000124233	P04279	ENSG00000124256	Q9H171

Input	UniProt Id	Input	UniProt Id	Input	UniProt Id
ENSG00000124275	Q9UBK8	ENSG00000124541	Q96EU6	ENSG00000124568	Q14916
ENSG00000124571	Q9HAV4	ENSG00000124635	P06899	ENSG00000124731	Q9NP99
ENSG00000124762	P38936	ENSG00000124780	Q96T54	ENSG00000124787	O75818
ENSG00000125430	Q9Y662	ENSG00000125454	Q9HC21	ENSG00000125657	P41273
ENSG00000125735	O43557	ENSG00000125740	P53539	ENSG00000125877	Q9BY32
ENSG00000125910	O95977	ENSG00000125968	P41134	ENSG00000125977	P20042
ENSG00000126067	P49721	ENSG00000126231	P22891	ENSG00000126262	O15552
ENSG00000126353	P32248	ENSG00000126522	P04424	ENSG00000126709	P09912
ENSG00000127588	Q9P2W3	ENSG00000127948	P16435	ENSG00000128016	P26651
ENSG00000129667	Q6PJF5	ENSG00000129673	Q16613	ENSG00000129749	Q9GZZ6
ENSG00000129810	Q5FBB7	ENSG00000130303	Q10589	ENSG00000130383	Q11128
ENSG00000130429	O15143	ENSG00000130508	Q92626	ENSG00000130589	Q9BYK8
ENSG00000130649	P05181	ENSG00000130766	P58004	ENSG00000130816	P26358
ENSG00000130829	Q99956	ENSG00000131979	P30793	ENSG00000132109	P19474
ENSG00000132341	P62826	ENSG00000132388	P62253	ENSG00000132646	P12004
ENSG00000133275	P78368	ENSG00000133313	Q96KP4	ENSG00000133619	A5PL33
ENSG00000133661	P35247	ENSG00000134061	Q99467	ENSG00000134070	O43187
ENSG00000134109	Q92611	ENSG00000134160	Q7Z4N2	ENSG00000134242	Q9Y2R2
ENSG00000134308	P27348	ENSG00000134375	Q99595	ENSG00000134504	Q719H9
ENSG00000134538	Q9Y6L6	ENSG00000134539	Q13241	ENSG00000134545	P26715, P26717
ENSG00000134640	P49286	ENSG00000134684	P54577	ENSG00000134690	Q53HL2
ENSG00000134755	Q02487	ENSG00000134757	P32926	ENSG00000134809	P62072
ENSG00000134827	P20061	ENSG00000136450	Q07955	ENSG00000136492	Q9BX63
ENSG00000136522	Q9HD33	ENSG00000136531	Q99250	ENSG00000136542	Q7Z7M9
ENSG00000136603	P12757	ENSG00000136634	P22301	ENSG00000136688	Q9NZH8
ENSG00000136689	P18510	ENSG00000136694	Q9UHA7	ENSG00000136695	Q9UBH0
ENSG00000136696	Q9NZH7	ENSG00000136698	P0CG37	ENSG00000137168	Q9Y3C6
ENSG00000137309	P17096	ENSG00000137440	Q14512	ENSG00000137462	O60603
ENSG00000137547	Q9P015	ENSG00000137563	Q92820	ENSG00000137692	Q9BTE7
ENSG00000137752	P29466	ENSG00000137757	P51878	ENSG00000137767	Q9Y6N5
ENSG00000137807	Q02241	ENSG00000137812	Q8NG31	ENSG00000138346	P51530
ENSG00000138363	P31939	ENSG00000138442	Q9GZL7	ENSG00000138496	Q8IXQ6
ENSG00000138594	Q9NYL9	ENSG00000138623	O75326	ENSG00000138642	Q8IVU3
ENSG00000138646	Q9UII4	ENSG00000139572	Q9NQS5	ENSG00000139618	P51587
ENSG00000139629	Q8NCL4	ENSG00000139687	P06400	ENSG00000139734	Q9NSV4
ENSG00000139988	Q96NR8	ENSG00000140022	Q8WXE9	ENSG00000140030	Q8IYL9
ENSG00000140105	P23381	ENSG00000140332	Q04726, Q04726-3	ENSG00000140368	O43586
ENSG00000140379	Q16548	ENSG00000140451	Q9H611	ENSG00000140464	P29590
ENSG00000140474	Q6PHR2	ENSG00000140479	P29122	ENSG00000140506	Q9HAT1
ENSG00000140519	Q9UBD6	ENSG00000140525	Q9NVI1	ENSG00000140557	Q92186
ENSG00000140564	P09958	ENSG00000140600	Q99963	ENSG00000140650	O15305
ENSG00000140678	P20702	ENSG00000141543	P38919	ENSG00000141682	Q13794
ENSG00000141738	Q14451	ENSG00000142089	Q01628	ENSG00000142185	O94759
ENSG00000142224	Q9UHD0	ENSG00000142507	P28072	ENSG00000142512	A6NMB1, Q96LC7
ENSG00000142583	P22732	ENSG00000142627	P29317	ENSG00000142634	Q96C19
ENSG00000142657	P52209	ENSG00000142945	Q99661	ENSG00000143106	P28066
ENSG00000143119	P19397	ENSG00000143153	P05026	ENSG00000143156	Q9Y5B8

Input	UniProt Id	Input	UniProt Id	Input	UniProt Id
ENSG00000143179	Q9BZX2	ENSG00000143184	P47992, Q9UBD3	ENSG00000143185	Q9UBD3
ENSG00000143226	P12318	ENSG00000143228	Q9BZD4	ENSG00000143257	Q14994-1, Q14994-2
ENSG00000143278	P05160	ENSG00000143319	Q9H9L3	ENSG00000143320	P29373
ENSG00000143321	P51858	ENSG00000143384	Q07820	ENSG00000143473	O95259
ENSG00000143476	Q9NZJ0	ENSG00000143546	P05109	ENSG00000143556	P31151, Q86SG5
ENSG00000143621	Q12905	ENSG00000143977	P62308	ENSG00000144118	P11234
ENSG00000144136	Q8WUM9	ENSG00000144837	Q53H76	ENSG00000144867	Q9Y5M8
ENSG00000145050	P55145	ENSG00000145113	Q99102	ENSG00000145194	P0DPD6
ENSG00000145246	Q9P241	ENSG00000145283	Q3KNW5	ENSG00000145287	Q9NZF1
ENSG00000145386	P20248	ENSG00000145604	Q13309	ENSG00000145623	Q99650
ENSG00000145632	Q9NYY3	ENSG00000147246	P28335	ENSG00000147614	Q8N8Y2
ENSG00000147813	Q6XQN6	ENSG00000147889	P42771, P42772, Q8N726	ENSG00000147912	Q9UK96
ENSG00000148136	Q8NGS5	ENSG00000148154	Q16739	ENSG00000148835	Q15542
ENSG00000148848	O43184	ENSG00000149054	Q9UL58	ENSG00000149923	P60510
ENSG00000149925	P04075	ENSG00000149948	P52926	ENSG00000149968	P08254
ENSG00000150244	Q8IWZ4	ENSG00000150337	P12314, Q92637	ENSG00000150551	Q8N2G4
ENSG00000150637	Q15762	ENSG00000150681	Q9NS28	ENSG00000150753	P48643
ENSG00000151012	Q9UPY5	ENSG00000151014	Q9UK39	ENSG00000151239	Q12792
ENSG00000151247	P06730	ENSG00000151388	P58397	ENSG00000151418	Q96LB4
ENSG00000151651	P78325	ENSG00000151689	P49441	ENSG00000151694	P78536
ENSG00000151790	P48775	ENSG00000152056	Q96PC3	ENSG00000152192	Q01851
ENSG00000152253	Q9HBM1	ENSG00000152266	P01270	ENSG00000152315	Q9HB14
ENSG00000152409	Q8N9B5	ENSG00000152413	Q86YM7	ENSG00000152455	Q9H5I1
ENSG00000152778	Q13325	ENSG00000153147	O60264	ENSG00000153283	P40200
ENSG00000153310	Q9NUQ9	ENSG00000153395	Q8NF37	ENSG00000153563	P01732
ENSG00000153898	Q8IZK6	ENSG00000153976	Q9Y663	ENSG00000154080	Q7L1S5-2
ENSG00000154096	P04216	ENSG00000154165	P49685	ENSG00000154227	Q8IU89
ENSG00000154380	Q8N8S7	ENSG00000155287	Q96A46	ENSG00000155368	P07108
ENSG00000155380	P53985	ENSG00000155846	Q86YN6	ENSG00000155918	Q5VY80
ENSG00000156017	Q8N4J0	ENSG00000156234	P02775	ENSG00000156282	P56750
ENSG00000156413	P51993, Q11128	ENSG00000156575	Q9Y2Y8	ENSG00000156587	O14933
ENSG00000156671	Q96LT4	ENSG00000156689	Q8WU03	ENSG00000156697	Q9BVJ6
ENSG00000158125	P47989	ENSG00000158220	A0FGR9	ENSG00000158402	P30307
ENSG00000158435	Q9UKZ1	ENSG00000158485	P29016	ENSG00000158517	P14598
ENSG00000158786	Q9BZM2	ENSG00000158859	O75173	ENSG00000159131	P22102
ENSG00000159224	P09681	ENSG00000159231	P16152	ENSG00000159337	Q86XP0
ENSG00000159399	P52789	ENSG00000159423	P30038	ENSG00000159479	Q96G25
ENSG00000159516	Q9BYE4	ENSG00000159527	Q96LB9	ENSG00000160179	P45844
ENSG00000160193	P57081	ENSG00000160691	P29353, P29353-2	ENSG00000160703	Q86UT6
ENSG00000160710	P55265	ENSG00000160791	P51681	ENSG00000160796	Q6ZNJ1
ENSG00000160883	P52790	ENSG00000161267	Q02338	ENSG00000161513	P22570
ENSG00000162063	P41002	ENSG00000162493	Q86YL7	ENSG00000162551	P05186
ENSG00000162572	P51172	ENSG00000162594	Q5VWK5	ENSG00000162639	Q5T8I9
ENSG00000162645	P32456	ENSG00000162654	Q96PP9	ENSG00000162695	Q8NEW0

Input	UniProt Id	Input	UniProt Id	Input	UniProt Id
ENSG00000162739	Q96DU3	ENSG00000162747	O75015	ENSG00000162777	Q9H6A0
ENSG00000162851	Q9H5Q4	ENSG00000162891	Q9NYY1	ENSG00000162892	Q13007
ENSG00000162924	Q04864	ENSG00000162959	Q9Y316	ENSG00000163131	P25774
ENSG00000163519	Q6PIZ9	ENSG00000163528	Q8N4Q1	ENSG00000163535	Q562F6
ENSG00000163563	P41218	ENSG00000163565	Q16666	ENSG00000163568	O14862
ENSG00000163584	Q6P5R6	ENSG00000163636	Q15008	ENSG00000163661	P26022
ENSG00000163734	P09341	ENSG00000163735	P42830	ENSG00000163736	P02775
ENSG00000163739	P09341	ENSG00000163808	Q9NS87	ENSG00000163882	P52434
ENSG00000163918	P35249, P35250	ENSG00000163961	Q8IYW5	ENSG00000163993	P25815
ENSG00000164024	P53582	ENSG00000164032	P0C0S5	ENSG00000164045	P30304
ENSG00000164047	P49913	ENSG00000164062	P13798	ENSG00000164070	O95757
ENSG00000164077	Q86VX9	ENSG00000164086	Q16829	ENSG00000164109	Q13257
ENSG00000164163	P61221	ENSG00000164181	A1L3X0	ENSG00000164182	Q8N183
ENSG00000164211	Q96DR4	ENSG00000164220	O00254	ENSG00000164284	Q8TAA5
ENSG00000164308	Q6P179	ENSG00000164904	P49419	ENSG00000164924	P63104
ENSG00000164934	Q9NV06	ENSG00000165025	P43405	ENSG00000165091	Q8TDI8
ENSG00000165168	P04839	ENSG00000165175	Q9NPA3	ENSG00000165195	P37287
ENSG00000165556	Q99626	ENSG00000165591	Q6GMR7	ENSG00000165732	Q9NR30
ENSG00000166123	Q8TD30-1	ENSG00000166183	Q86U10	ENSG00000166226	P78371
ENSG00000166278	P06681	ENSG00000166368	Q9H210	ENSG00000166394	Q6BCY4
ENSG00000166401	P50452	ENSG00000166523	Q9ULY5	ENSG00000166527	Q8WXI8
ENSG00000167088	P62314	ENSG00000167207	Q9HC29	ENSG00000167264	Q9NX74
ENSG00000167553	Q9BQE3	ENSG00000167600	Q96SQ9	ENSG00000167617	Q6NZY7
ENSG00000167618	Q6ISS4	ENSG00000167656	Q14210	ENSG00000167900	P04183
ENSG00000167916	P08727, Q2M2I5	ENSG00000167941	Q9BQB4	ENSG00000167984	Q7RTR2
ENSG00000168081	Q13519	ENSG00000168237	Q8IVS8	ENSG00000168259	Q99615
ENSG00000168275	Q5JTJ3	ENSG00000168393	P23919	ENSG00000168394	Q03518
ENSG00000168398	P30411, P46663	ENSG00000168404	Q8NB16	ENSG00000168412	P48039
ENSG00000168421	Q15669	ENSG00000168528	Q96SA4	ENSG00000168610	P40763
ENSG00000168685	P16871	ENSG00000168884	Q8NFZ5	ENSG00000168961	O00182
ENSG00000168995	Q9Y286	ENSG00000169035	P49862	ENSG00000169174	Q8NBP7
ENSG00000169220	O43566	ENSG00000169228	Q969Q5	ENSG00000169230	Q9Y255
ENSG00000169245	P02778	ENSG00000169248	O14625	ENSG00000169258	Q7Z2K8
ENSG00000169299	Q96G03	ENSG00000169385	P10153	ENSG00000170128	O00155
ENSG00000170231	P51161	ENSG00000170312	P06493	ENSG00000170423	Q8N1N4
ENSG00000170426	Q8NEX9	ENSG00000170454	O95678	ENSG00000170465	P04259, P48668
ENSG00000170477	P19013	ENSG00000170482	Q9UHI7	ENSG00000170486	Q14CN4
ENSG00000170515	Q9UQ80	ENSG00000170581	P52630	ENSG00000170606	P34932
ENSG00000170786	Q8N3Y7	ENSG00000170848	Q00889	ENSG00000170893	P20396
ENSG00000170899	O15217	ENSG00000170920	Q8NG95	ENSG00000170953	Q8NGG6
ENSG00000170956	P40198	ENSG00000171049	P21462, P25090	ENSG00000171051	P21462, P62942
ENSG00000171056	Q9BT81	ENSG00000172350	Q9H172	ENSG00000172432	Q9BX10
ENSG00000172464	Q8NGF4	ENSG00000172531	P62140	ENSG00000172548	Q0D2K0
ENSG00000172575	O95267	ENSG00000172590	Q86TS9	ENSG00000172602	Q92730
ENSG00000172724	Q99731	ENSG00000172757	P23528	ENSG00000173559	Q96AH0
ENSG00000173692	Q99460	ENSG00000173801	P14923	ENSG00000174502	Q7LBE3

Input	UniProt Id	Input	UniProt Id	Input	UniProt Id
ENSG00000174775	P01112	ENSG00000174837	Q14246	ENSG00000174842	Q92990
ENSG00000174951	P19526	ENSG00000175354	P17706-2	ENSG00000175426	P29120
ENSG00000175463	Q8IV04	ENSG00000175591	P41231	ENSG00000175592	P15407
ENSG00000175634	Q9UBS0	ENSG00000175643	Q96E14	ENSG00000175768	Q8N4H5
ENSG00000175792	Q9Y265	ENSG00000175793	P31947	ENSG00000175832	P43268
ENSG00000175893	Q8IVQ6	ENSG00000176014	Q9BUF5	ENSG00000176153	P18283
ENSG00000176290	Q96R72	ENSG00000176597	Q9BYG0	ENSG00000176749	Q15078
ENSG00000176797	P81534	ENSG00000176890	P04818	ENSG00000176900	Q8NGJ9
ENSG00000176920	Q10981	ENSG00000176922	Q8NGJ8	ENSG00000176925	Q8NH61
ENSG00000176928	Q9P109	ENSG00000177000	P42898	ENSG00000177058	Q8NBW4
ENSG00000177105	P84095	ENSG00000177192	Q9Y606-2	ENSG00000177201	Q8NG77
ENSG00000177202	Q8TDM5	ENSG00000177238	Q6ZMU5	ENSG00000177243	P81534
ENSG00000177257	O15263	ENSG00000177272	P22001	ENSG00000177535	Q5JQS5
ENSG00000177556	O00244	ENSG00000177889	P61088	ENSG00000178172	Q6UWN8
ENSG00000178295	Q17RS7	ENSG00000178372	Q9NZT1	ENSG00000178562	P10747
ENSG00000181449	P48431	ENSG00000181467	P61225	ENSG00000181544	Q8NB91
ENSG00000181625	Q9BQ83	ENSG00000181634	O95150	ENSG00000181652	Q674R7
ENSG00000181733	Q8NG97	ENSG00000181924	Q9NYJ1	ENSG00000181938	Q9BRX5
ENSG00000182199	P34897	ENSG00000182264	Q8IYV9	ENSG00000182389	O00305
ENSG00000182481	P52292	ENSG00000182566	Q6UXB4	ENSG00000182585	Q6UW88
ENSG00000182628	Q8WVK7	ENSG00000182687	O43603	ENSG00000182752	Q13219
ENSG00000182983	Q6ZS27	ENSG00000183018	Q8IVW8	ENSG00000183019	Q8IX19
ENSG00000183048	Q9UBX3	ENSG00000183336	Q9H3K6	ENSG00000183347	Q6ZN66
ENSG00000183395	P20382	ENSG00000183484	Q9UNW8	ENSG00000183486	P20592
ENSG00000183520	Q9Y3A2	ENSG00000183684	Q86V81	ENSG00000183696	Q16831
ENSG00000184967	Q9BVI4	ENSG00000184979	Q9UMW8	ENSG00000185052	Q9HC58
ENSG00000185069	Q01546	ENSG00000185745	P09914	ENSG00000185811	Q13422-1
ENSG00000185880	Q86WT6	ENSG00000185885	P13164	ENSG00000185897	O14843
ENSG00000185899	P59551	ENSG00000185947	Q14586	ENSG00000185962	Q5TA76
ENSG00000185966	Q5T5B0	ENSG00000186074	Q8TDQ1	ENSG00000186113	Q8NGL3
ENSG00000186153	Q9NZC7	ENSG00000186185	Q86Y91	ENSG00000186191	P59827
ENSG00000186197	Q8WWZ3	ENSG00000186265	Q7Z6A9	ENSG00000186306	Q8NGX3
ENSG00000186407	Q496F6	ENSG00000186431	P24071	ENSG00000186442	P12035
ENSG00000186716	P11274	ENSG00000186810	P49682	ENSG00000186818	Q8NHJ6
ENSG00000186827	P43489	ENSG00000186832	P08779	ENSG00000186847	P02533, P08779, Q04695
ENSG00000186871	Q2NKX8	ENSG00000187080	Q8NG84	ENSG00000187091	P51178
ENSG00000187116	A6NI73	ENSG00000187173	Q5TA79	ENSG00000187238	Q5TA77
ENSG00000187258	Q6W5P4	ENSG00000187801	Q9UJL9	ENSG00000187840	Q13541
ENSG00000187908	Q9UGM3	ENSG00000188037	P35523	ENSG00000188389	Q15116
ENSG00000188404	P14151	ENSG00000188486	P16104	ENSG00000188559	Q2PPJ7
ENSG00000188672	P18577	ENSG00000188676	Q6ZQW0	ENSG00000189013	Q99706
ENSG00000189181	A6ND48	ENSG00000196743	P17900	ENSG00000196747	Q99878
ENSG00000196776	Q08722	ENSG00000196805	P35325	ENSG00000196832	Q8NGC1
ENSG00000196954	P49662	ENSG00000197061	P62805	ENSG00000197233	Q8NGS2
ENSG00000197238	P62805	ENSG00000197241	Q6PXP3	ENSG00000197249	P01009
ENSG00000197780	Q15543	ENSG00000198018	Q9NQZ7	ENSG00000198019	Q92637
ENSG00000198283	A6NL26	ENSG00000198286	Q9BXL7	ENSG00000198400	P04629, P04629-1
ENSG00000198431	Q16881	ENSG00000198780	Q9Y6X4	ENSG00000198805	P00491

Input	UniProt Id	Input	UniProt Id	Input	UniProt Id
ENSG00000198814	P32189, Q14409, Q14410	ENSG00000198821	P20963-1	ENSG00000198826	Q6P4F7
ENSG00000198851	P07766	ENSG00000198890	Q96LA8	ENSG00000198900	P11387
ENSG00000198901	O43663	ENSG00000203722	Q6H3X3	ENSG00000203747	P08637
ENSG00000203760	Q5EE01	ENSG00000203785	P22531	ENSG00000204178	Q8N5G2
ENSG00000205420	P04264	ENSG00000205667	Q5FYA8	ENSG00000205678	Q5HYJ1
ENSG00000205755	Q9HC73	ENSG00000205809	P26717	ENSG00000205846	Q6EIG7, Q8WTT0
ENSG00000205882	Q4QY38	ENSG00000206384	A6NMZ7	ENSG00000213471	A6NNM8
ENSG00000213512	Q8N8V2	ENSG00000213585	P21796	ENSG00000213658	O43561, O43561-2
ENSG00000214643	Q30KQ1	ENSG00000221977	Q8NGC2	ENSG00000223572	P12532
ENSG00000226650	O95239, Q2VIQ3	ENSG00000227140	A8MUK1	ENSG00000227471	C9JRZ8
ENSG00000227507	Q06643	ENSG00000228278	P19652, Q8N138	ENSG00000228716	P00374
ENSG00000229894	Q14409	ENSG00000233816	P01562	ENSG00000234745	P01889, P03989, P10319, P18463, P18464, P18465, P30460, P30461, P30462, P30464, P30466, P30475, P30479, P30480, P30481, P30483, P30484, P30485, P30486, P30487, P30488, P30490, P30491, P30492, P30493, P30495, P30498, P30685, Q04826, Q29718, Q29836, Q29940, Q31610, Q31612, Q95365
ENSG00000236125	A6NCW7	ENSG00000237038	P0C7I0	ENSG00000237289	P12532
ENSG00000237541	P01906	ENSG00000237649	Q9BW19	ENSG00000238243	Q7Z3T1
ENSG00000239590	Q8NGS1	ENSG00000239672	P15531	ENSG00000239732	Q9NR96
ENSG00000239839	P59666	ENSG00000241356	P0C626	ENSG00000241794	P35326
ENSG00000242221	P11465	ENSG00000242366	Q9HAW9	ENSG00000242515	Q9HAW8
ENSG00000242550	P48595	ENSG00000243135	P35503	ENSG00000243509	O95407
ENSG00000243649	P00751	ENSG00000243708	P0C869	ENSG00000243989	Q03154
ENSG00000244057	Q5T5A8	ENSG00000244094	Q96RM1	ENSG00000244122	Q9HAW7
ENSG00000244474	P22310	ENSG00000244482	Q6PI73	ENSG00000246705	Q9BTM1
ENSG00000247077	Q96HS1-2	ENSG00000254087	P07948	ENSG00000254415	Q08ET2, Q96PQ1
ENSG00000254521	Q96PQ1	ENSG00000255095	P47884	ENSG00000255398	P49019
ENSG00000255582	Q8NGC3	ENSG00000256269	P08397	ENSG00000257017	P00738
ENSG00000258366	Q9NZ71	ENSG00000258947	Q13509	ENSG00000259494	Q9H2W6
ENSG00000261236	Q14137	ENSG00000261456	Q3ZCM7	ENSG00000262406	P39900
ENSG00000262628	P58170	ENSG00000268089	Q9UN88	ENSG00000268104	Q9UN76
ENSG00000268221	P04001	ENSG00000271503	P13501	ENSG00000274997	Q99878
ENSG00000276903	P0C0S8	ENSG00000277632	P10147, P16619	ENSG00000277775	P68431
ENSG00000278030	P04435	ENSG00000278053	Q9Y2R4	ENSG00000278463	P04908
ENSG00000278588	P62807				

Input	Ensembl Id	Input	Ensembl Id	Input	Ensembl Id
ENSG00000003400	ENSG00000003400	ENSG00000005844	ENSG00000005844	ENSG00000007171	ENSG00000007171
ENSG00000023330	ENSG00000023330	ENSG00000049768	ENSG00000049768	ENSG00000051180	ENSG00000051180
ENSG00000057657	ENSG00000057657	ENSG00000087088	ENSG00000087088	ENSG00000089685	ENSG00000089685
ENSG00000090061	ENSG00000090061	ENSG00000090520	ENSG00000090520	ENSG00000093009	ENSG00000093009
ENSG00000094804	ENSG00000094804	ENSG00000100292	ENSG00000100292	ENSG00000100453	ENSG00000100453
ENSG00000100644	ENSG00000100644	ENSG00000101871	ENSG00000101871	ENSG00000103449	ENSG00000103449

Input	Ensembl Id	Input	Ensembl Id	Input	Ensembl Id
ENSG00000105173	ENSG00000105173	ENSG00000105246	ENSG00000105246	ENSG00000108691	ENSG00000108691
ENSG00000109971	ENSG00000109971	ENSG00000111331	ENSG00000111331	ENSG00000111335	ENSG00000111335
ENSG00000111641	ENSG00000111641	ENSG00000112096	ENSG00000112096	ENSG00000112115	ENSG00000112115
ENSG00000112116	ENSG00000112116	ENSG00000113302	ENSG00000113302	ENSG00000113368	ENSG00000113368
ENSG00000114013	ENSG00000114013	ENSG00000115129	ENSG00000115129	ENSG00000115232	ENSG00000115232
ENSG00000115415	ENSG00000115415	ENSG00000117632	ENSG00000117632	ENSG00000117724	ENSG00000117724
ENSG00000117984	ENSG00000117984	ENSG00000119508	ENSG00000119508	ENSG00000119535	ENSG00000119535
ENSG00000120217	ENSG00000120217	ENSG00000120694	ENSG00000120694	ENSG00000120738	ENSG00000120738
ENSG00000124762	ENSG00000124762	ENSG00000125968	ENSG00000125968	ENSG00000126709	ENSG00000126709
ENSG00000130303	ENSG00000130303	ENSG00000130489	ENSG00000130489	ENSG00000130766	ENSG00000130766
ENSG00000132109	ENSG00000132109	ENSG00000132646	ENSG00000132646	ENSG00000133661	ENSG00000133661
ENSG00000134109	ENSG00000134109	ENSG00000136634	ENSG00000136634	ENSG00000136689	ENSG00000136689
ENSG00000137752	ENSG00000137752	ENSG00000140379	ENSG00000140379	ENSG00000140464	ENSG00000140464
ENSG00000140525	ENSG00000140525	ENSG00000140678	ENSG00000140678	ENSG00000141682	ENSG00000141682
ENSG00000142089	ENSG00000142089	ENSG00000142657	ENSG00000142657	ENSG00000143321	ENSG00000143321
ENSG00000143384	ENSG00000143384	ENSG00000144867	ENSG00000144867	ENSG00000145386	ENSG00000145386
ENSG00000145632	ENSG00000145632	ENSG00000147889	ENSG00000147889	ENSG00000148136	ENSG00000148136
ENSG00000149968	ENSG00000149968	ENSG00000150244	ENSG00000150244	ENSG00000150337	ENSG00000150337
ENSG00000151012	ENSG00000151012	ENSG00000151014	ENSG00000151014	ENSG00000152778	ENSG00000152778
ENSG00000158402	ENSG00000158402	ENSG00000159224	ENSG00000159224	ENSG00000160179	ENSG00000160179
ENSG00000160691	ENSG00000160691	ENSG00000160710	ENSG00000160710	ENSG00000160791	ENSG00000160791
ENSG00000162594	ENSG00000162594	ENSG00000162645	ENSG00000162645	ENSG00000162654	ENSG00000162654
ENSG00000162851	ENSG00000162851	ENSG00000163508	ENSG00000163508	ENSG00000163739	ENSG00000163739
ENSG00000164045	ENSG00000164045	ENSG00000165556	ENSG00000165556	ENSG00000166368	ENSG00000166368
ENSG00000167900	ENSG00000167900	ENSG00000168610	ENSG00000168610	ENSG00000169245	ENSG00000169245
ENSG00000169258	ENSG00000169258	ENSG00000170231	ENSG00000170231	ENSG00000170312	ENSG00000170312
ENSG00000170920	ENSG00000170920	ENSG00000170953	ENSG00000170953	ENSG00000171051	ENSG00000171051
ENSG00000172464	ENSG00000172464	ENSG00000172724	ENSG00000172724	ENSG00000172757	ENSG00000172757
ENSG00000175354	ENSG00000175354	ENSG00000175793	ENSG00000175793	ENSG00000176290	ENSG00000176290
ENSG00000176749	ENSG00000176749	ENSG00000176890	ENSG00000176890	ENSG00000176900	ENSG00000176900
ENSG00000176922	ENSG00000176922	ENSG00000176925	ENSG00000176925	ENSG00000177201	ENSG00000177201
ENSG00000177535	ENSG00000177535	ENSG00000181449	ENSG00000181449	ENSG00000181733	ENSG00000181733
ENSG00000182481	ENSG00000182481	ENSG00000183336	ENSG00000183336	ENSG00000183347	ENSG00000183347
ENSG00000183486	ENSG00000183486	ENSG00000185745	ENSG00000185745	ENSG00000185885	ENSG00000185885
ENSG00000186113	ENSG00000186113	ENSG00000186306	ENSG00000186306	ENSG00000187080	ENSG00000187080
ENSG00000188672	ENSG00000188672	ENSG00000189181	ENSG00000189181	ENSG00000196832	ENSG00000196832
ENSG00000197233	ENSG00000197233	ENSG00000198019	ENSG00000198019	ENSG00000198283	ENSG00000198283
ENSG00000198431	ENSG00000198431	ENSG00000213512	ENSG00000213512	ENSG00000228716	ENSG00000228716
ENSG00000233816	ENSG00000233816	ENSG00000234745	ENSG00000234745	ENSG00000237541	ENSG00000237541
ENSG00000238243	ENSG00000238243	ENSG00000239590	ENSG00000239590	ENSG00000241356	ENSG00000241356
ENSG00000243135	ENSG00000243135	ENSG00000243708	ENSG00000243708	ENSG00000255095	ENSG00000255095
ENSG00000255582	ENSG00000255582	ENSG00000262628	ENSG00000262628	ENSG00000271503	ENSG00000271503
ENSG00000277632	ENSG00000277632				

7. Identifiers not found

These 1 identifiers were not found neither mapped to any entity in Reactome.

ENSG00000207782
