# Supplementary material for: Biology-inspired graph neural network encodes reactome and reveals biochemical reactions of disease
Source: Patterns (N Y). 2023 May 22;4(7):100758. doi: 10.1016/j.patter.2023.100758 (PMC10382942; doi:10.1016/j.patter.2023.100758)
Supplement: Document S2. Article plus supplemental information [file mmc14.pdf]

Patterns

Article

Biology-inspired graph neural network encodes reactome and reveals biochemical reactions of disease

Graphical abstract

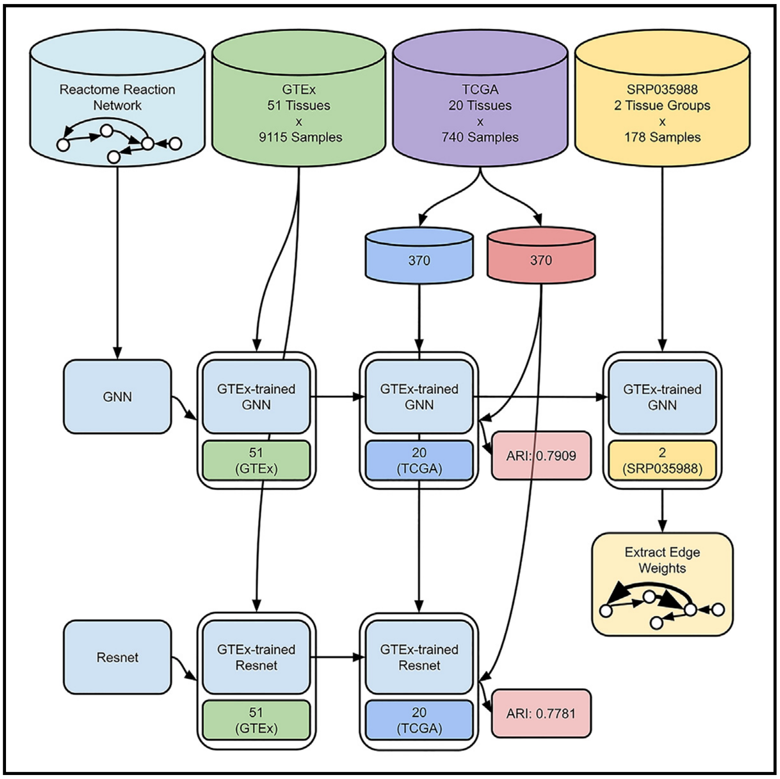

Highlights

- Biochemical reaction states are approximated with PCA-transformed RNA-seq count data
- Our graph neural network outperforms both random rewired and deep learning controls
- Analysis of our model reveals reactions associated with psoriasis in prior studies
- Traditional analysis approaches fail to discover the reactions found with our method

Authors

Joshua G. Burkhart, Guanming Wu, Xubo Song, Francesco Raimondi, Shannon McWeeney, Melissa H. Wong, Youping Deng

Correspondence

jgburk@hawaii.edu (J.G.B.),
dengy@hawaii.edu (Y.D.)

In brief

Identification of dysregulated biochemical reactions is critical to comprehending molecular mechanisms of disease. In this work, we leverage a graph neural network based on the Reactome reaction network to reveal biochemical reactions associated with disease and supported by prior literature. We evaluate a principal-component data transformation process and find that our model outperforms both random rewired and deep learning controls. Further, we show that the biochemical reactions our model discovers remain hidden by traditional differential gene expression and hypergeometric enrichment analyses.

Burkhart et al., 2023, Patterns *4*, 100758
July 14, 2023 © 2023 The Author(s).
https://doi.org/10.1016/j.patter.2023.100758

Patterns

CellPress
OPEN ACCESS

Article

Biology-inspired graph neural network encodes reactome and reveals biochemical reactions of disease

Joshua G. Burkhart,[1,6,*] Guanming Wu,[2] Xubo Song,[3] Francesco Raimondi,[4] Shannon McWeeney,[2] Melissa H. Wong,[5] and Youping Deng[1,*]

[1]Department of Quantitative Health Sciences, University of Hawaii John A. Burns School of Medicine, Honolulu, HI 96813, USA
[2]Division of Bioinformatics and Computational Biology, Department of Medical Informatics and Clinical Epidemiology, Oregon Health & Science University, Portland, OR 97239, USA
[3]Department of Computer Science and Electrical Engineering, Oregon Health & Science University, Portland, OR 97239, USA
[4]BIO@SNS, Scuola Normale Superiore di Pisa, 56126 Pisa, Italy
[5]Department of Cell, Developmental, and Cancer Biology, Oregon Health & Science University, Portland, OR 97201, USA
[6]Lead contact
*Correspondence: jgburk@hawaii.edu (J.G.B.), dengy@hawaii.edu (Y.D.)
https://doi.org/10.1016/j.patter.2023.100758

THE BIGGER PICTURE The Human Genome Project unlocked the door to a vast but unannotated collection of genes. In the following decades, annotations in the form of biochemical reaction graphs were painstakingly curated via experimental studies. Though gene set enrichment analysis considers groups within these annotation graphs, it disregards group dependencies. Here, we utilize these dependencies by generating a graph neural network based on the Reactome reaction network and show how integrating the curated relationships from this graph with gene expression values from other studies can be used to identify biochemical reactions associated with tissue-specific disease. In the future, similar approaches could enable fruitful reanalyses of prior work, highlighting influential relationships and pinpointing biochemical reactions. As more experimental research databases become available, we envision extensions of our work predicting effects of rare or indistinct genetic variations and guiding precision medicine.

1 2 3 4 5 **Proof-of-Concept:** Data science output has been formulated, implemented, and tested for one domain/problem

SUMMARY

Functional heterogeneity of healthy human tissues complicates interpretation of molecular studies, impeding precision therapeutic target identification and treatment. Considering this, we generated a graph neural network with Reactome-based architecture and trained it using 9,115 samples from Genotype-Tissue Expression (GTEx). Our graph neural network (GNN) achieves adjusted Rand index (ARI) = 0.7909, while a Resnet18 control model achieves ARI = 0.7781, on 370 held-out healthy human tissue samples from The Cancer Genome Atlas (TCGA), despite the Resnet18 using over 600 times the parameters. Our GNN also succeeds in separating 83 healthy skin samples from 95 lesional psoriasis samples, revealing that upregulation of 26S- and NUB1-mediated degradation of NEDD8, UBD, and their conjugates is central to the largest perturbed reaction network component in psoriasis. We show that our results are not discoverable using traditional differential expression and hypergeometric pathway enrichment analyses yet are supported by separate human multi-omics and small-molecule mouse studies, suggesting future molecular disease studies may benefit from similar GNN analytical approaches.

This is an open access article under the CC BY license (http://creativecommons.org/licenses/by/4.0/).

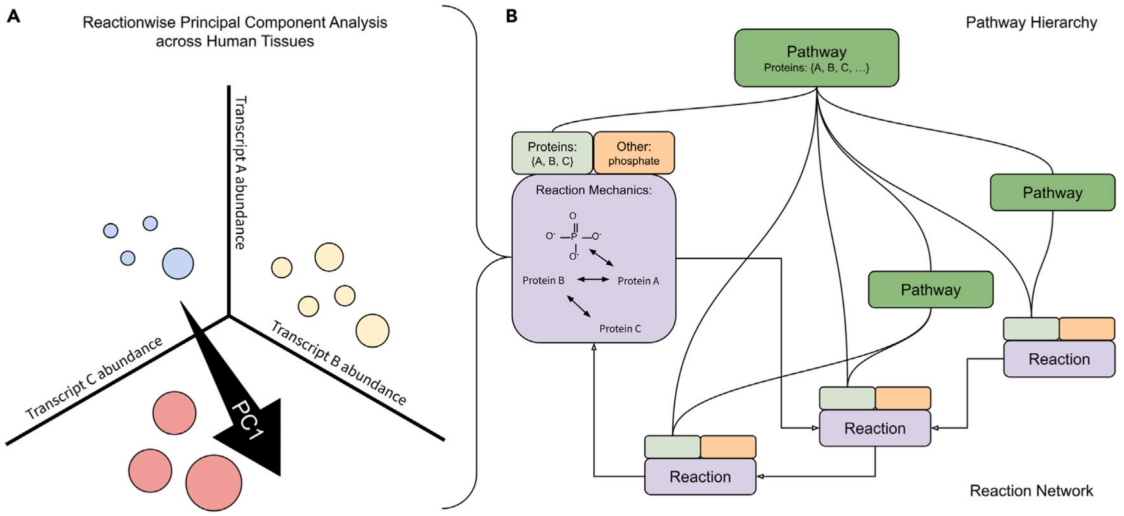

Figure 1. Transcript aggregation within a reaction and across reaction network

(A) A first principal component (PC1) drawn within a space representing a reaction to which three proteins are annotated. Axes represent three transcript abundance levels corresponding to the reaction's three proteins. Circles represent individual tissue samples. Circle color represents sample tissue type. Circle size is varied to suggest image depth.

(B) Reactions composed as three information sets: proteins (light green boxes), other reaction components (orange boxes), and reaction mechanics (purple boxes). Protein sets are shown to be connected to a pathway hierarchy (dark green boxes) by curved connectors. Reaction mechanics are shown to be connected to each other by arrows.

INTRODUCTION

Variation of mRNA abundance across tissue results from complex phenomena

Across human tissues, mRNA abundance varies,[1–3] and consequent differential protein expression is likewise observed,[4,5] affecting both which and to what degree biochemical reactions take place.[6–8] While it is understood that human tissues harbor characteristic gene expression patterns,[9] gene products exhibit complex relationships with cellular behaviors as a result of RNA and protein modification, variation in small-molecule abundances, differential cell compartment morphology, and, presumably, other phenomena that preclude straightforward extrapolation from gene expression values to the states of biochemical reactions carrying out tissue functions.[10–15] Despite considerable advances of contemporary omics-based biological studies, this complexity remains largely hidden from us. Achieving high confidence regarding which reactions are likely to occur or assume different states within particular tissue contexts is paramount in order to understand the biochemical mechanisms of tissue development and tissue-specific functions and the etiology of tissue-specific disease.

Protein expression is primarily determined by mRNA expression

Using RNA sequencing,[16] experimentalists are able to approximate the abundance of mRNA present in a cell or group of cells. Though many biochemical reactions involve protein-protein interactions and not interactions of mRNA, specific mRNA synthesis is required for specific protein synthesis, and thus mRNA patterns are associated with protein-regulated phenotypes and cell states. Total mRNA abundance is positively correlated with protein abundance (R^2 = 0.41 on log-log scale, and R^2 = 0.44 following nonlinear transformation[17]), and, when approximate steady-state conditions are met, protein abundance has been shown to be primarily determined by mRNA abundance,[18] with between 56% and 84% of variation in protein abundance shown to be explained by mRNA abundance alone.[19]

Biochemical reactions may be reasonably characterized by mRNA expression alone

In order for any particular reaction to occur, its necessary reactants must be present; however, there may not be direct correspondence between a protein's measured abundance and its availability to participate in a biochemical reaction due to competitive occupancy among binding partners or post-translational modifications.[10,11,13–15,20,21] Furthermore, some reaction components, such as small molecules, small interfering RNA (siRNA), metal ions, and other organic and inorganic compounds, are currently unquantified in a tissue-specific manner and remain unavailable for consideration in cross-tissue analyses. However, many biochemical reactions are mediated by enzymes resulting from translation of mRNA, which influence the reaction rate, and their expression may act as proxies for specific reaction states.[22–30] Thus, biochemical reaction metrics characteristic to particular tissues may be inferred by identifying patterns repeated across multiple samples among the mRNA transcripts coding for proteins that participate in specific biochemical reactions. Such patterns of mRNA transcript abundance that associate with particular tissues imply patterns of protein abundance[17–19] and—by syllogism—the biochemical reaction states characteristic of those tissues. Within a tissue, proteins and other components participate in interactions and react with each other to form a network.[31–35] Tissues may thus plausibly be characterized by the combined states of their biochemical reaction networks using mRNA transcript abundance values alone.

CellPress
OPEN ACCESS

Table 1. Tissue sample counts of GTEx training dataset

GTEx tissue label	Number of samples
Adipose – subcutaneous	386
Adipose – visceral (omentum)	234
Adrenal gland	159
Artery – aorta	247
Artery – coronary	140
Artery – tibial	363
Bladder	11
Brain – amygdala	81
Brain – Ant. cin. cortex (BA24)	99
Brain – caudate (basal ganglia)	134
Brain – cerebellar hemisphere	118
Brain – cerebellum	145
Brain – cortex	132
Brain – frontal cortex (BA9)	120
Brain – hippocampus	103
Brain – hypothalamus	104
Brain – Nuc. acc. (basal ganglia)	123
Brain – putamen (basal ganglia)	103
Brain – spinal cord (cervical c-1)	76
Brain – substantia nigra	71
Breast – mammary tissue	218
Cervix – ectocervix	6
Cervix – endocervix	5
Colon – sigmoid	173
Colon – transverse	203
Esophagus – gastro. junction	176
Esophagus – mucosa	331
Esophagus – muscularis	283
Fallopian tube	7
Heart – atrial appendage	218
Heart – left ventricle	271
Kidney – cortex	36
Liver	136
Lung	374
Minor salivary gland	70
Muscle – skeletal	475
Nerve – tibial	335
Ovary	108
Pancreas	197
Pituitary	124
Prostate	119
Skin – not sun exposed (suprapubic)	271
Skin – sun exposed (lower leg)	397
Small intestine – terminal ileum	104
Spleen	118
Stomach	204
Testis	203
Thyroid	361
Uterus	90
Vagina	97
Whole blood	456

GTEx tissue labels and RNA-seq sample counts were downloaded from Recount2 and used for the training procedure.

Reaction characterization and connections are determined and validated in this study by public datasources

To characterize biochemical reaction network states across tissue, we leveraged the graph neural network paradigm, which has been demonstrated to scale to large graphs.[36] We combined gene expression values from Genotype-Tissue Expression (GTEx),[9] the largest source of human tissue-specific RNA sequencing (RNA-seq) data with the biochemical reaction network annotations from Reactome,[37] the most comprehensive pathway database. Reactome is the largest biological pathway database and is unique in its repertoire and representation of biochemical reactions. Each reaction annotated in Reactome is approved by human experts and is traceable to its source literature. Considering a reaction's state is approximated by its constituent protein participants' transcript abundances, we generated values for each reaction by applying principal-component analysis (PCA)[38] across transcript sets annotated for each reaction as a conceptually simple, computationally efficient, and outcome-naive aggregation strategy (Figure 1). We then created a graph neural network architecture[39] using the Reactome reaction network and trained it to classify 51 tissue types using the transformed GTEx reaction data (Figure 7), embedding tissue-specific reaction network states as weights in our trained model. This graph structure is critical to the overall workflow in obtaining biologically significant results because the graph structure specifies reaction interdependency. By including this graph, the graph neural network (GNN) is able to consider information from multiple interdependent reactions we can extract after training. In contrast, other deep neural network models assume architectures not representative of biological systems. We validated our trained model with a transfer learning approach (results) where we show that our GNN classifies held-out healthy tissue samples from The Cancer Genome Atlas (TCGA)[40] as well as a conventional deep learning model[41] trained on the same data, despite the conventional deep learning model using more than 600 times the parameters. Furthermore, we show that by applying our trained model to a study comparing healthy skin tissue with lesional psoriasis samples,[42] our GNN recovers biochemical reactions shown to promote psoriasis in both a human multi-omics integrative genomics study[43] and a separate study using a mouse model,[44] yet these are not discoverable by traditional differential gene expression or pathway enrichment analyses.

RESULTS

Training data

Our model is trained with data from GTEx, the largest healthy human dataset available

The GTEx Project and TCGA are two of the largest-scale human tissue-specific RNA-seq studies conducted over the preceding

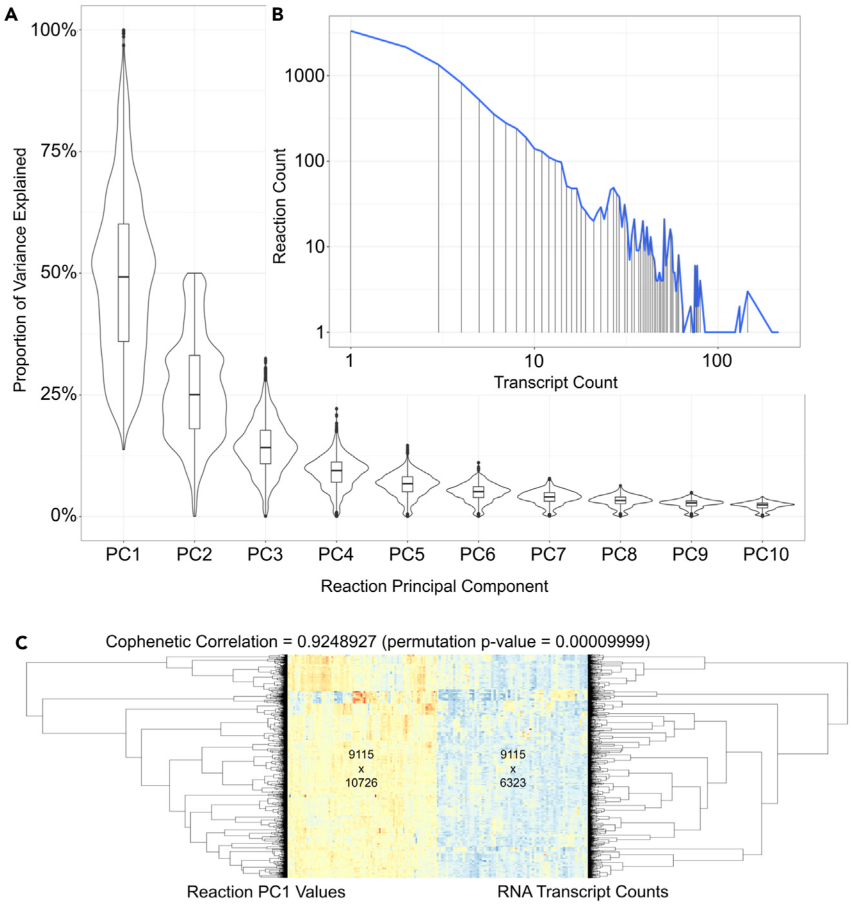

Figure 2. Reaction PCA summary statistics

(A) Principal-component distributions of proportions of variance explained across the first ten principal components.

(B) A log-log plot of reaction and transcript counts.

(C) Tissue sample dendrograms from both reaction PC1 (left) and transcript counts (right) are significantly correlated (Cophenetic correlation permutation p-value = 0.00009999). Heatmap columns shown are downsampled from the underlying matrices for visualization purposes.

decade; the data generated by each have been made largely publicly available and are commonly subject to reanalyses. Each of these studies' RNA-seq samples as well as many others hosted by the Sequence Read Archive (SRA) have been reprocessed in a uniform way with a single pipeline by the Recount2 project,[45] which provides sample phenotype data and gene transcript count data through their online portal at https://jhubiostatistics.shinyapps.io/recount/. We opted to use this Recount(-ed) GTEx data to train our GNN because it represented the most samples (9, 115) and reserved Recount(-ed) TCGA and other data for downstream validation.

Our data-transformation procedure maps mRNA expression values to reaction-specific values using PCA

We calculated reaction-specific values to train a GNN to classify reaction graphs. GTEx gene transcript data were downloaded and grouped in a many-to-many fashion according to the Reactome reactions in which their protein products participated. Tissue sample counts ranged from 5 (endocervix) to 475 (skeletal muscle), as indicated in Table 1. We considered several methods to reduce dimensionality from RNA-seq transcript counts to reaction-specific values including t-distributed stochastic neighbor embedding (t-SNE),[46] uniform manifold approximation and projection (UMAP),[47] and potential of heat diffusion for affinity-based transition embedding (PHATE).[48] PCA was selected due to concerns for both performance and simplicity. Reactionwise PCA was conducted using the *prcomp()* function from the R *stats* package,[49] and resulting principal-component result objects were stored. The distribution of the proportion of variance explained by the first ten principal components across all reactions is plotted in Figure 2A, where the median of the first principal components explains 50% of the variance, the median of the second principal components explains 25% of the variance, and the subsequent principal components tend to explain less variance, as would be expected. The first principal component value from each reaction was recorded for each sample, forming a samplewise PC1 matrix. This routine transformed a matrix representing 6,323 gene transcripts representing 6,323 unique genes across 9,115 tissue samples to a matrix representing 10,726 reactions across those same 9,115 samples. Reaction transcript counts ranged from 1 (multiple reactions) to 214 for "olfactory receptor-G protein olfactory trimer complex formation" (Reactome:R-HSA-381750), with the log reaction-log transcript count distribution shown in Figure 2B.

Sample structure maintenance

The sample structure of our training data is maintained by our data transformation procedure

A common method to assess gene expression information across samples is to perform hierarchical clustering, which may reveal the structure among samples by considering overall samplewise similarity. In order to determine whether calculating reactionwise principal-component summarization significantly degraded or otherwise influenced this structure, we calculated the Euclidean distance between samples and performed agglomerative hierarchical clustering using Ward's D[50,51] on both reaction principal-component coordinates and transcript counts across all samples and compared the resulting dendrograms. Cophenetic correlation[52,53] is a method used to calculate the similarity between dendrograms based on sample distance. The cophenetic correlation coefficient was calculated to be 0.9248 (Figure 2C) and was shown to be significant by permuting over the reaction dendrogram labels as specified in the documentation for the *cor_cophenetic()* function from the R *dendextend* package[54] 10,000 times ($p < 1E-5$). This demonstrates that the reactionwise first principal-component coordinate

CellPress
OPEN ACCESS

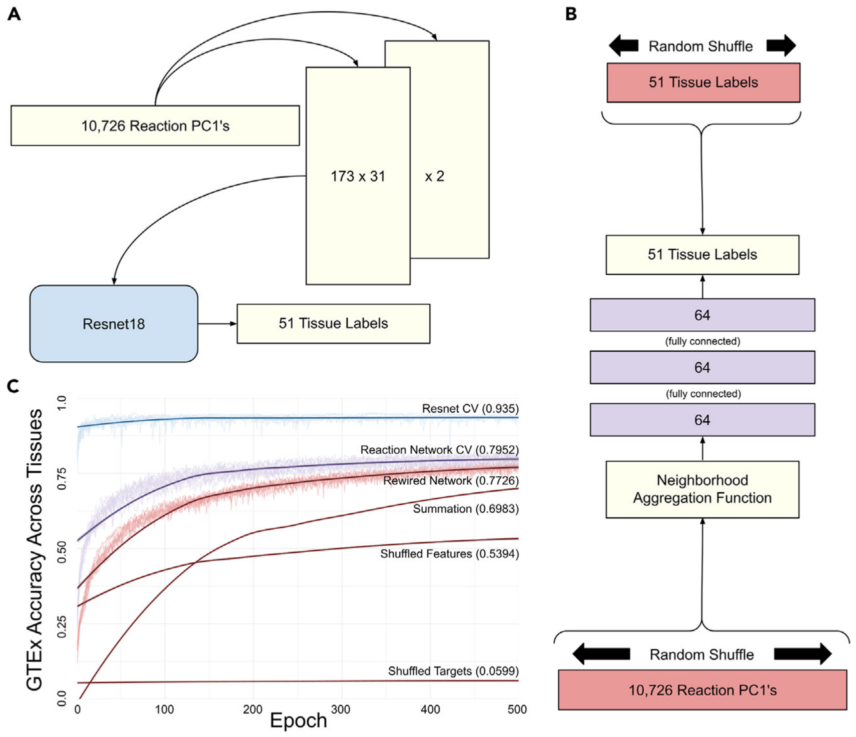

Figure 3. Training results with positive and negative controls

(A) Reaction PC1 values reshaped into image-like tensors for Resnet18, used to classify 51 tissues as a positive control.

(B) Both randomly shuffled reaction PC1 values and randomly shuffled tissue labels are used as features and targets, respectively, as negative controls.

(C) Classification accuracy of held-out samples using K = 10-fold cross-validation and overlaid loess curves for both our graph neural network and Resnet and all training samples for 10 "Rewired Network," "Shuffled Features," and "Shuffled Targets" control models, as well as our "Summation model," which we used to determine the contribution of PCA.

values group tissue samples similarly to transcript counts, justifying our feature matrix transformation. The advantage of expressing this information as reactionwise values, rather than transcript counts, is that reactions form a network distinct from other networks formed by transcripts alone. The transformation of transcript count information into reactionwise information offers the opportunity to view gene expression information through the lens of the human biochemical reaction network.

Training performance

We use Resnet, a conventional deep learning model, shuffled and summation data as controls

To approximate an upper bound on the degree to which our reaction PC1 values explain the tissue types in our training data, we used Resnet18,[41] a deep learning model recommended as a default selection.[55] Resnet18 was developed by Microsoft to classify images with 18 convolutional layers. Because Resnet18 is an image classifier, we reshaped our samplewise reaction PC1 values from a 1-dimensional vector of length 10,726 to two 2-dimensional vectors of height 173 and width 31 using the PyTorch[56] *reshape()* function after redefining the first layer as a 2 channel, rather than the default Resnet18 3 channel used for RGB images. We also modified the final layer to output tissue labels (Figure 3A). After ten executions of 500 epochs, this Resnet18 architecture achieved an accuracy of 93.5% using K = 10-fold cross-validation, where the accuracy was calculated as the mean proportion of samples in the held-out folds correctly classified (Figure 3C). Convolutional neural networks (CNNs) use hidden layers, which consider adjacent values together. Though CNNs perform well on image classification—partly because adjacent pixel relationships are meaningful for images—these neural network models contain no inherent representation of interdependent reactions, unlike our GNN. We considered the possibility that additional information about biological network structure may be subtly encoded in this input data by positioning biologically related reaction PC1 values near each other in the resulting 2-channel matrix. To test whether this was the case, we shuffled the reaction PC1 values and retrained our Resnet18 model; however, these results were indistinguishable from one another, suggesting that such additional information does not contribute to our Resnet18 model performance. Our GNN was trained using the same routine and achieved a mean accuracy of 79.52%. The observed 13.98% accuracy difference of the Resnet18 architecture vanishes when tested against held-out validation data (model validation), suggesting that the apparent performance advantage is merely due to the Resnet18 overfitting on the GTEx training data. To establish lower bounds on our training performance, we used our GNN with four control datasets: a degree-preserving randomly rewired[57] reaction graph, "Rewired Network"; randomly shuffled reaction PC1 values within samples, "Shuffled Features"; randomly shuffled tissue labels across samples, "Shuffled Targets"; and summed expression values, rather than PC1 values, "Summation" (Figure 3). We trained each of our controls using the full GTEx dataset and reported training accuracy after 500 epochs (Figure 3C). Notably, we observed that our "Rewired Network" control model achieves a mean accuracy of 77.26% across 10 randomly rewired graphs. Despite this representing training—rather than held-out fold—error, we recognize this as relatively high accuracy and hypothesize it is an artifact of the redundant structure in the human reaction network itself.[58] We hypothesize that the unexpectedly high "Shuffled Features" control model accuracy of 53.94% is the result of cases where the sum of reaction PC1 values for one or more samples for some tissues is high and the sum of reaction PC1 values for other tissues is low. Considering the variation observed across tissue sample size (Table 1), one would not expect a random classifier to perform quite as poorly as 1/51 (<2%). We hypothesize that the 5.99% accuracy of our "Shuffled Targets" control model is a reflection of this sample size imbalance. To determine the contribution of PCA to our analysis, we

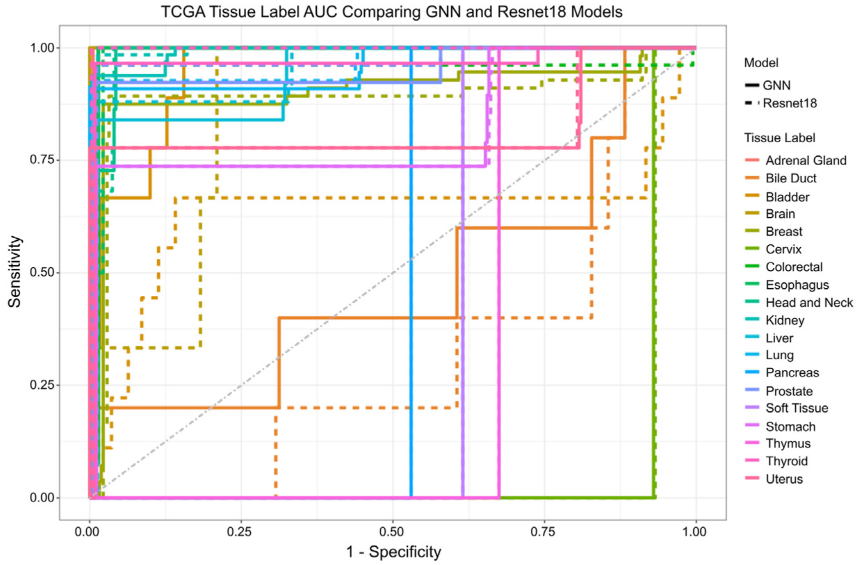

Figure 4. TCGA test set area under the curve (AUC) across tissue labels

MultiROC plot showing TCGA tissue label one-vs.-rest AUC comparing performance of the graph neural network (GNN; solid lines) and Resnet18 (dashed lines). Both models exhibit excellent AUC, defined as AUC >0.8, for adrenal gland, brain, and all TCGA tissue labels with 5 or more samples in the tuning set, except for bladder. The GNN showed a higher AUC than the Resnet18 model for 10 tissues, while the Resnet18 model showed a higher AUC for 6 (Table 2). Though the models' differences in AUC for most tissue labels are small, these results demonstrate that the apparent disadvantage of the GNN relative to the Resnet18 model observed during training does not extend to test data.

also trained a "Summation" control model using reaction-specific values calculated by taking the sum of expression values corresponding to each reaction (Figure 3C). Using the full GTEx training data, this model achieves an accuracy of 69.83%, showing it fits better than either model trained using shuffled data but worse than models using unshuffled PC1 values. Following this cross-validation assessment, Resnet and GNN models were trained using the full GTEx dataset for downstream validation.

Model validation

Fitting GTEx-trained GNN for comparison to GTEx-trained Resnet18, "Rewired Network," and "Summation" model performance via transfer learning

Our GNN outperforms the control models at classifying TCGA tissue samples. Neural networks have been shown to represent generalizable features in their early layers and dataset-specific features in later ones.[59] Considering this, to validate our GNN, we used an approach that held all except the final classification layer weights fixed. We demonstrated performance via transfer learning on a separate dataset, showing that our GNN outperforms the GTEx-trained Resnet18 conventional deep learning architecture despite it using over 600 times the parameters (18,004 for our GNN compared with 11,183,636 for Resnet18). We use 740 healthy tissue samples from TCGA, representing 20 tissue types. We divide these samples with a 50:50 split into tissue type-balanced tuning and test datasets, each composed of 370 unique samples (Table 2). Because both our GTEx-trained Resnet18 and GNN models were constructed with final layers mapping to the 51 tissue types GTEx data represented, these layers were replaced with linear layers mapping to the 20 tissues represented in our TCGA validation dataset. The tuning dataset was used to adjust the new final layers of both our GNN (where the new final layer represents 1,300 parameters) and the Resnet18 (where the new final layer represents 10,260 parameters), with prior layer weights frozen in place, as they were following the tuning procedure using the full GTEx dataset. Using this approach, both our GTEx-trained GNN and the GTEx-trained Resnet18 converge and achieve 100% accuracy on the tuning set in 487 and 214 epochs, respectively; however, our tuned GNN outperforms the tuned Resnet18 on TCGA test dataset, where our GNN achieves an adjusted Rand index[60] (ARI) = 0.7909 and the Resnet18 achieves an ARI = 0.7781. This demonstrates that the apparent advantage of the Resnet18 observed through 10-fold cross-validation accuracy during the GTEx-training routine relied upon overfitting to GTEx-specific features—not features generalizable to unseen data. The 10 "Rewired Network" models and the "Summation" model used as controls in our training procedure achieve mean ARIs = 0.6568 and 0.4541, respectively, on TCGA test dataset after the same layer replacement and tuning. This shows that both the real Reactome network structure and PCA contribute to our model, outperforming respective control models by 16.96% and 42.58%. Notably, misclassified test samples from real Reactome and Resnet models were highly disjointed (Figures S1 and S2; Table S1); though only 282/370 (76.22%) of the test samples were correctly classified by both models, 348/370 (94.05%) of the test samples were correctly classified by at least one of the models, suggesting the possibility of fruitful results from future ensembles of GNN-based learning and conventional deep learning approaches. See Data S1, S8, S9, S10, S11, and S12 for the full list of real Reactome and Resnet model classification results and full TCGA execution records and data, respectively.

Fitting GTEx-trained GNN for comparison to traditional expression and enrichment analyses

Our GNN separates healthy skin from psoriatic lesional skin samples. Recently, GNNs have been reported to perform well at anomaly detection tasks due to their ability to exploit underlying relationship structures.[61–63] To test our model's ability to identify subnetworks of the reactome dysregulated by disease and compare our results with those of traditional differential gene expression analyses, we applied our GNN to a study of healthy and psoriatic skin. Briefly, psoriasis is a complex, currently uncured chronic relapsing inflammatory skin disorder characterized by painful rashes of scaly skin[64] that is estimated

Table 2. Tissue sample counts of TCGA datasets and test set AUC

TCGA tissue label	Tuning set	Test set	Resnet AUC	GNN AUC
Adrenal gland	2	1	1*	0.994
Bile duct	4	5	0.304	0.472*
Bladder	10	9	0.635	0.951*
Brain	2	3	0.869	1*
Breast	56	56	0.885	0.901*
Cervix	2	1	0.067	0.07*
Colorectal	25	26	0.958	0.986*
Esophagus	7	6	0.989	0.995*
Head and neck	22	22	0.985	0.988*
Kidney	64	65	0.984	0.988*
Liver	25	25	0.955*	0.944
Lung	55	55	0.968*	0.959
Pancreas	2	2	0.47	0.47
Prostate	26	26	0.966*	0.95
Skin	1	0	–	–
Soft tissue	1	1	0.384	0.384
Stomach	18	19	0.826*	0.819
Thymus	1	1	0.325	0.325
Thyroid	30	29	0.997*	0.966
Uterus	17	18	0.816	0.82*

TCGA tissue label counts for tuning and held-out test sets with their corresponding AUC for both the Resnet and GNN models are shown.[1]
[1]*Higher Tissue-specific AUC

to affect 3.2% of US adults.[65] Several clinical variants are described; however, pathogenesis generally consists of dysregulated keratinocyte proliferation and differentiation concomitant with neovascularization.[66] In moderate to severe cases, long-term therapy options include fewer than 20 small molecules and biologics, many of which target the same tumor necrosis factor α (TNF-α) signaling and interleukin pathways.[66] To potentiate enumeration of additional targets, we selected the largest psoriasis dataset whose RNA-seq data were processed using the same Recount2 pipeline as the GTEx (and TCGA) samples that our GNN was trained (and validated) with: SRA: SRP035988,[42] containing 178 samples (83 healthy skin and 95 lesional psoriatic skin). To investigate this dataset, we replaced the final layer of our GNN with a linear layer mapping to two conditions, representing healthy and diseased skin tissue, and held all other layer weights in place, as with TCGA tuning procedure, while we fit the final layer of the model using all the available data. Fitting our GNN in this way, our GNN converges in 300 epochs.

Our GNN reveals that a large reaction network component is perturbed in psoriatic tissue and that a drug target in the central reaction has been previously identified in a separate mouse study. Following this fitting procedure, we calculated the reaction connection weight using the 83 healthy skin samples to arrive at 40,032 edge weights for the reaction network, representing the reaction connection importance to the GNN for classifying healthy skin samples from the SRA:SRP035988 dataset (Figure 5). Briefly, edge weights were arrived upon by averaging gradients from the output labels backward through the GNN to input features using the integrated gradients technique described by Sundararajan et al.[67] and implemented in the *Captum*[68] PyTorch library. To analyze components of the resulting weighted reaction network, we first used three thresholds: the top 1% of edges, representing the 400 most important reaction pairs; the top 25% of cumulative reaction network edge weights, representing the 225 most important reaction pairs; and the top 100 edges, representing the 100 most important reaction pairs. However, using each of these thresholds, the largest network component extracted from our GNN was centered about the same network hub reaction Reactome:R-HSA-8956184: 26S- and NUB1-mediated degradation of NEDD8, UBD, and their conjugates (https://reactome.org/content/detail/R-HSA-8956184), which is a central component of the neddylation pathway linked to psoriasis in the literature. This reaction is preceded only by its requisite binding reaction Reactome:R-HSA-8956140: NEDD8 and UBD bind NUB1 and the 26S proteasome (https://reactome.org/content/detail/R-HSA-8956140), to which all the same gene products are annotated. A small-molecule drug, MLN4924, targets NEDD8 via inhibition of its activating enzyme NAE. Initially developed as a cancer therapy to arrest the cell cycle,[69] it has been demonstrated *in vivo* in a separate study that MLN4924 administration promotes psoriasis in mice.[44] Reactome:R-HSA-8956184 is represented by 21 transcripts detected in the SRA:SRP035988 study representing 21 unique genes (Table 3).

The central reaction of the perturbed network component remains hidden using traditional gene expression analysis techniques. In an attempt to reproduce this finding with traditional differential gene expression and pathway enrichment analysis, we used the same SRA:SRP035988 gene transcript count data we used to fit our GNN along with the R *EnhancedVolcano* package,[70] a software package that applies the *lfcShrink()* function[71] and calculates s-values[72] for differential expression analysis. To ensure that this package yielded appropriate results for the SRA:SRP035988 dataset, we first used its default parameters to find significantly differentially expressed genes reported in prior literature as associated with psoriasis (Table S2; Figure S3). We then considered differential gene expression at three significance thresholds: strict, defined by a $\log_2$ fold change ($\log_2$FC) >2 and an s-value <10e−32; default, defined by a $\log_2$(FC) >2 and a p <10e−6 (the EnhancedVolcano default); and relaxed, defined by a $\log_2$(FC) >0.5 and an s-value <0.05. Using the strict, default, and relaxed thresholds, 117, 135, and 904 gene transcripts are considered differentially expressed in SRA:SRP035988, respectively. See Data S2 for the complete differentially expressed (DE) transcript list and corresponding FC and significance values. Despite the central nature of this reaction in our weighted reaction network, none of its constituent transcripts are found to be DE between healthy skin and lesional psoriatic tissue using either the strict or default thresholds in the EnhancedVolcano package. Using relaxed thresholds, 8 of the 21 transcripts are calculated to be DE (Table 3; Figure 6A), and following multiple hypothesis correction using a Benjimini-Hochberg procedure, Reactome:R-HSA-8956184 is enriched with a nonsignificant adjusted p value of 0.58727. See Data S3 for the full list of reaction enrichment results.

Original differential gene expression and pathway enrichment results. Li et al.[42] conducted differential expression and pathway

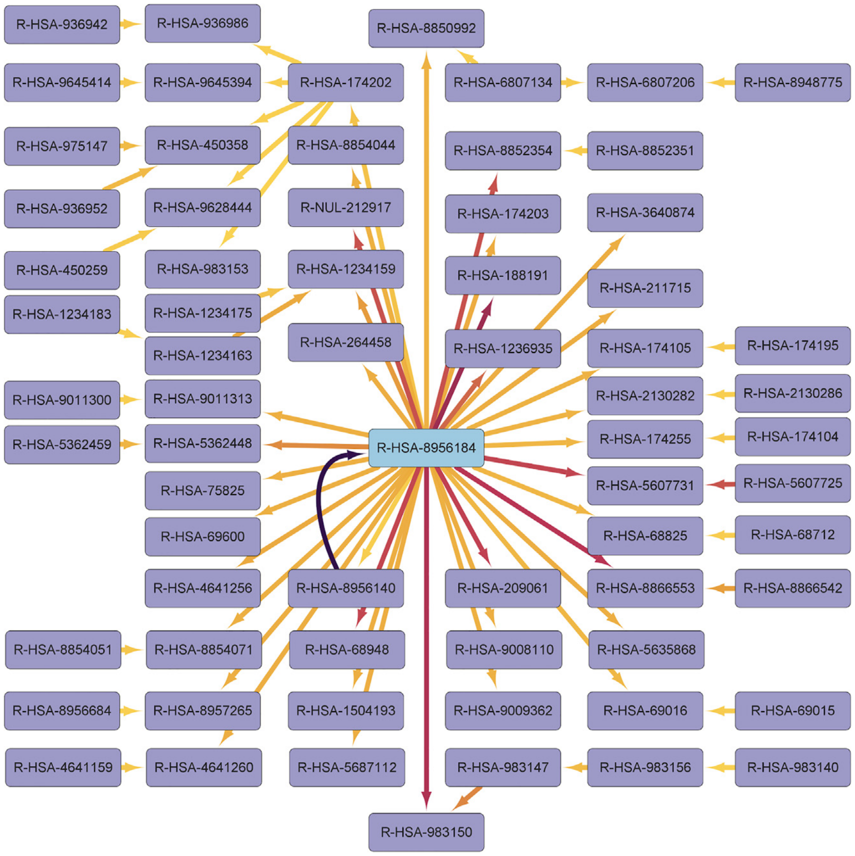

Figure 5. Neddylation is central in largest differential network component of psoriatic skin

The largest network component among the top 25% of total edge weight in our GNN applied to SRA:SRP035988 samples is centered about Reactome:R-HSA-8956184: 26S- and NUB1-mediated degradation of NEDD8, UBD, and their conjugates. Nodes (light purple) are labeled with Reactome's stable identifiers, and edges are colored by weight (yellow = low and dark purple = high). In this diagram, edge weight represents importance for separation of normal skin from psoriatic lesional skin samples; edge direction represents the preceding/following reaction network relationship, where preceding reactions' products are reactants, catalysts, or regulators of following reactions. We identify reaction Reactome:R-HSA-8956184 (light blue) as a hub in this network component and note that 37 of its 52 total connections in the original reaction network—1 preceding reaction and 36 following reactions—are included within the top 25% of total edge weight.

enrichment analyses, finding significant enrichment of genes overexpressed in psoriatic lesions involved in inflammatory responses, cytokine-receptor interactions, cell division, and keratinization.[42] These pathways are represented in the authors' dataset by 268, 275, 305, and 41 genes, respectively.[42] These pathways are largely recapitulated in our traditional enrichment reanalyses at each of the three thresholds performed using Reactome pathways; however, pathways referring specifically to *neddylation* or NEDD8 itself are absent. See Data S5, S6, and S7for the enrichment reports. Together, these findings demonstrate the contribution of our GNN as a combination of both capable of discovering genotype-phenotype relationships that are nonsignificant by traditional statistical methods as well as reaction connection-aware learning, capable of localizing those discoveries to specific biochemical reactions deep within the pathway hierarchy.

Hypergeometric enrichment analysis fails to detect edges. If the relaxed thresholds described above (*The central reaction of the perturbed network component remains hidden using traditional gene expression analysis techniques*) were used for differential gene expression to conduct Reactome edge (reaction-pair) enrichment analysis, 41%, 29%, and 16% of the edges in the top 1%, top 25% of cumulative edge weights, and top 100 edges, respectively, would be undiscoverable using hypergeometric enrichment analysis, even in the $b = 0$ setting, where all of their transcripts are considered DE, as those edges are represented in SRA:SRP035988 by fewer than 8 transcripts (experimental procedures). This demonstrates the bias of hypergeometric enrichment analysis toward large pathways and its inability to detect positively associated smaller ones.

The connections of the central reaction in the perturbed network component remain hidden using traditional gene expression analysis techniques. The 52 edges representing reactions preceding and following Reactome:R-HSA-8956184 were also considered using the union of each reaction pair's transcripts, and none of the edges were found to be significantly enriched using strict, default, or relaxed thresholds. The most significantly enriched edge connected to Reactome:R-HSA-8956184 using the relaxed threshold, 26S- and NUB1-mediated degradation of NEDD8, UBD, and their conjugates precedes proteasomal degradation of K48polyUb-TRAF3 (https://reactome.org/content/detail/R-HSA-5668481), was found within the top 1% of edges in our analysis and calculated to be enriched with a nonsignificant Benjimini-Hochberg adjusted p value of 0.05986. See Data S4 for the full list of edge enrichment results.

The central reaction of the perturbed network component reveals two distinct expression clusters. Genes in one of these clusters have been characterized as key drivers of psoriasis in a separate human multi-omics study. Notably, Pearson correlation of expression values for these eight genes found to be significantly DE using the relaxed threshold results in the formation of two distinct clusters (Figure S4). Cluster 1 consists of PSMD11, PSMA3, PSMD1, and PSMD6, and cluster 2 consists of PSMA5, PSME1, PSMB2, and PSMB6. Two members of the first cluster, PSMD1 and PSMD6, have previously been reported as key drivers of psoriasis in a human multi-omics study.[43] These clusters do not appear to be associated by shared loci, promoters/enhancers (Table S3), or 20S complex assembly interdependency.[73] The two least-correlated genes are PSMA3 and PSMB6 (Pearson $r = 0.365$). Despite their shared overexpression in psoriasis (Figures 6B and 6D), these genes exhibit both natural and psoriatic variation, with diverging expression

CellPress
OPEN ACCESS

Table 3. Differential gene expression of a central reaction in psoriasis

Gene name	Ensembl ID	log2(FC)	s-value	Threshold
PSMD11	Ensembl:ENSG00000108671	0.612	2.95E−87	relaxed
PSMA5	Ensembl:ENSG00000143106	0.677	1.08E−66	relaxed
PSME1	Ensembl:ENSG00000092010	0.549	4.35E−57	relaxed
PSMB2	Ensembl:ENSG00000126067	0.633	1.09E−55	relaxed
PSMA3	Ensembl:ENSG00000100567	0.711	2.51E−50	relaxed
PSMD1	Ensembl:ENSG00000173692	0.515	3.94E−49	relaxed
PSMD6	Ensembl:ENSG00000163636	0.501	1.58E−43	relaxed
PSMB6	Ensembl:ENSG00000142507	0.538	6.69E−22	relaxed
PSMD14	Ensembl:ENSG00000115233	0.455	1.13E−25	(none)
PSMB3	Ensembl:ENSG00000277791	0.485	2.91E−20	(none)
PSMD7	Ensembl:ENSG00000103035	0.233	1.47E−12	(none)
PSMC6	Ensembl:ENSG00000100519	0.308	1.25E−10	(none)
PSMB1	Ensembl:ENSG00000008018	0.22	2.11E−09	(none)
SEM1	Ensembl:ENSG00000127922	0.265	3.30E−08	(none)
PSMD9	Ensembl:ENSG00000110801	0.25	4.27E−06	(none)
PSMB4	Ensembl:ENSG00000159377	0.11	0.005	(none)
PSMD5	Ensembl:ENSG00000095261	0.099	0.035	(none)
PSMD4	Ensembl:ENSG00000159352	0.081	0.098	(none)
PSMF1	Ensembl:ENSG00000125818	0.042	0.207	(none)
NEDD8	Ensembl:ENSG00000129559	−0.043	0.323	(none)
PSMD10	Ensembl:ENSG00000101843	0.023	0.47	(none)

Transcripts representing Reactome:R-HSA-8956184 in the SRA:SRP035988 study. None of these transcripts are significantly differentially expressed using the strict or default thresholds. Using relaxed thresholds, 8 of these transcripts are differentially expressed. However, Reactome:R-HSA-8956184 itself is not considered significantly enriched at any of the thresholds.

gradients observed across each condition (Figures 6C and 6E). These genes form the α3 and β6 subunits of the human constitutive 20S proteasome,[74] respectively. The constitutive 20S proteasome is a core component of the 26S proteasome,[75,76] suggesting that 26S proteasome overexpression resulting in upregulated NEDD8 degradation occurs through Reactome:R-HSA-8956184 by two separate expression avenues, corresponding to each of the two clusters.

DISCUSSION

Our GNN models healthy human tissues and enables tissue-specific disease analysis

This study sought to characterize the differential biochemical reactions of healthy human tissue using mRNA expression data and use resulting information to analyze disease. To accomplish this, we transformed healthy human tissue-specific RNA-seq data from GTEx, mapping gene transcript counts to annotated biochemical reactions using Reactome reaction annotations, and performed dimensionality reduction with PCA. Using the reactionwise PCA-transformed data and constraining potential relationships to those found within the Reactome reaction network, we generated a GNN, aggregating layers using the *GraphConv()* function[39] with nodes representing reactions and edges representing preceding (product)-following (reactant) relationships. We assessed our method by including positive and negative performance controls in our training procedure as well as showing a quantitative performance advantage relative to randomly rewired controls as well as a conventional deep learning model on held-out validation data. We demonstrated a qualitative advantage by extracting reaction network edge weights and revealing an expression-phenotype association not discoverable by conventional expression analysis techniques yet supported by results of both a human multi-omics study[43] and a separate small-molecule study using a mouse model.[44] Individually, each element of this workflow has been previously described; however, we contend that the unique composition of our biology-inspired design choices demonstrates the advantage and potential of this and similar interdisciplinary work.

Similar models have recently been shown to reflect biological systems

Inherent in our modeling technique is the implication that biochemical networks process information, a theory proposed in the literature more than a century ago.[77] However, only more recently has it been shown that chemical networks exhibit features capable of explaining subtle life sciences phenomena[78] and that, in addition to traditional ordinary differential equation (ODE) and state-based modeling techniques,[79–81] neural network models with appropriate architecture reflect the behavior of biological systems.[82–85]

Our GNN differs from and extends existing models

Considering prior neural network models,[82–84] our work differs in three important ways. First, we used data from human bulk RNA-seq rather than gene knockout or binary gene mutation

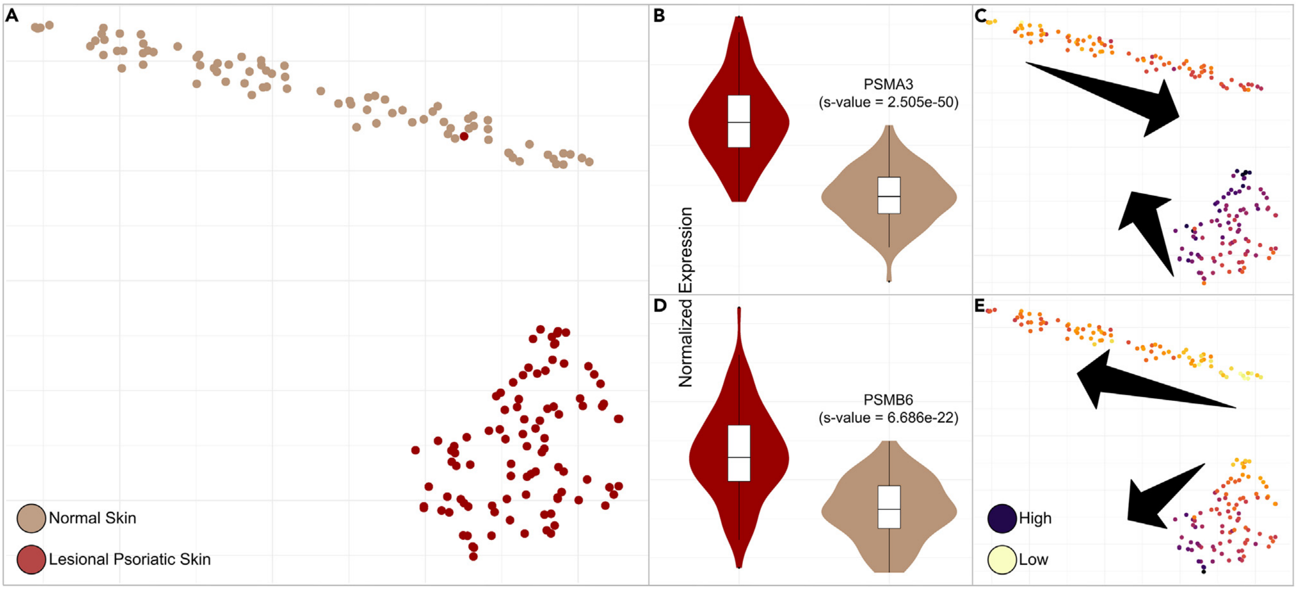

Figure 6. Nonuniform overexpression of 20S subunit genes PSMA3 and PSMB6 in psoriasis
(A) A t-SNE plot[46] of healthy (beige) and lesional psoriatic skin (crimson) samples calculated using the normalized expression values of all genes.
(B and D) The expression distributions of the two least correlated of the significantly differentially expressed genes (PSMA3 and PSMB6) representing the hub reaction Reactome:R-HSA-8956184.
(C and E) The expression values of PSMA3 and PSMB6 in the t-SNE context (yellow = low and dark purple = high).
Interestingly, despite these genes' similar differential expression distributions when considered across conditions (B and D), the t-SNE context reveals diverging gradients within each condition (C and E).

data from *S. cerevisiae*, *E. coli*, or transformed cell lines in order to interrogate differentiated *ex vivo* human tissue in place of single cells or model organisms. This choice was a compromise in that it reduces the number of samples available for training and validation but enables direct investigation of reaction subnetworks underlying medical conditions using widely available bulk RNA-seq sample data. Second, the Reactome network from which we derived our architecture represents biochemical reactions specific to *H. sapiens* and is manually curated by experts rather than a representation of ontological relations with varying degrees of granularity and evidence codes[86,87] or automatically generated using databases of experimental results.[88] This difference constrains our GNN to set weights for edges representing individually reviewed reaction connections, rather than inferred or ontological relations, which is important because highly parameterized neural networks have been shown to offer myopic solutions,[89] potentiating overfitting and misinterpretation. Finally, prior work was assessed quantitatively by showing comparable performance relative to a similarly sized, fully connected neural network positive control.[82] We opted not to restrict the parameter space or architecture of our positive control in these ways, showing that our model performs comparably to a conventional Resnet18 deep learning architecture rather than one designed following the evaluation of our GNN.

Our GNN method complements existing analytical techniques

The modest isoform coverage and consequent incompleteness of Reactome[37] limit possible insights into mechanism; thus, differential expression analysis remains advantageous for discovery of novel associations at transcript and gene scales. Moreover, pathway enrichment analysis is sufficient to find associations at broad scales; however, we demonstrate our method as complementary to both differential expression and enrichment approaches due to its ability to reveal associations using expression data at the mesoscale of biochemical reactions.

Our study was limited by data-inherent characteristics and methodological compromises

This project was limited by several factors and compromises that should be taken in concert with our results. Firstly, the data we perform our analysis with exhibit several issues. Our training set size of 9,115 represents an infinitesimal fraction of the human population and is smaller than prior demonstrations of biology-inspired neural networks,[82–84] which were trained on circa 500,000 samples. We chose to use this sample size after careful review of available data, finding no additional data suitable for our purposes at the time of writing, aside from healthy TCGA samples, which we opted to hold out and use as a validation dataset. These data themselves have several limitations. Consider, we use training samples collected from cadaver tissue, indicating that all donors experienced death due to some cause. The tissues from which the samples were collected may have appeared healthy; however, a donor may have suffered an unidentified illness, such as metastatic cancer, or experienced some temperature fluctuation or other environmental exposure perhaps leading to misrepresented healthy tissue gene expression patterns. Furthermore, these samples

CellPress
OPEN ACCESS

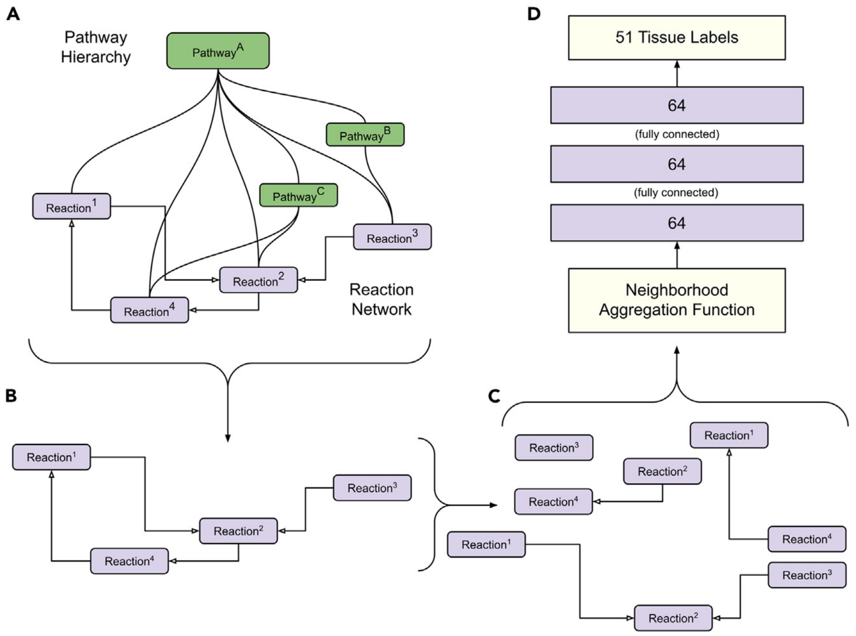

Figure 7. Reaction network decomposition and model input

(A) The reactome pathway hierarchy and reaction network.
(B) The reaction network.
(C) The reaction network decomposition for the reaction network deep learning architecture.
(D) The reaction network architecture with the 1-GNN *GraphConv()* aggregator function, passed through three layers of size 64 and trained to classify 51 tissue types.

were collected using bulk RNA-seq, which provides only an average value of gene expression for each tissue. We understand that tissues are host to many cell types of varying lineage, and it may be the case that only the primary cell types in these tissue samples are represented in our input data. As large-scale single-cell databases come online, network analytical techniques such as ours may prove to better characterize reaction networks *in situ*. We use RNA-seq data themselves to approximate states of biochemical reactions, though we know that transcript abundance has been shown only to correlate with protein abundance and has not been shown to correlate directly with the reaction state itself. In the future, large-scale, tissue-specific phosphoproteomic datasets may be able to address this shortcoming. Next, the biochemical networks described by Reactome are understood to be incomplete. We trained our model using only 6,323 transcripts for each sample that experts have annotated as participating in reactions, but the human genome now contains 19,901 to 21,306 genes annotated as protein coding.[90] Reactome is increasing coverage over time but does not yet completely cover the human transcriptome. Finally, our methods lose some information. We chose PCA as a method for transcript-to-reaction dimensionality reduction because it was a simple and performant method, making no assumptions about tissue label and allowing us to maintain maximum naivete. Increasingly sophisticated methods are developed for studying RNA transcript values, utilization of which may explain more than a median of 50% of the variance our first principal components did. And parameters for our GNN architectures were left unaltered from the literature in which they were described. This choice likely translated into underperformance but allowed us to compare our results conservatively with positive and negative controls in an unbiased way. Tuning our GNN parameters based on our training data could have introduced undue bias in our first demonstration of this approach and brought results from our controls into question. However, extensions of this work could focus more specifically on generating GNN models optimized for both performance and information retention.

EXPERIMENTAL PROCEDURES

Resource availability

Lead contact

Further information and requests for resources and reagents should be directed to and will be fulfilled by the lead contact, Joshua G. Burkhart (jgburk@hawaii.edu).

Materials availability

This study did not generate new unique reagents.

Data and Code availability

- Data from GTEx, TCGA, and SRA:SRP035988 used to train and validate our GNN are available from the Recount2 data download portal at https://jhubiostatistics.shinyapps.io/recount/.
- The Reactome MySQL database is freely available for download from the Reactome webpage at https://reactome.org/download-data.
- Intermediate data files generated by our work are available as supplemental information.
- The source code accompanying this work is publicly available at https://github.com/joshuaburkhart/reticula. The source code has been released as v.1.0.2 to reflect the repository version at publication time. This version is available at https://zenodo.org/record/7811429, and the corresponding DOI is 10.5281/zenodo.7811429.

Model generation

GNN architecture

In order to infer tissue-specific biochemical networks, we used manually curated reaction network annotations from Reactome to generate a GNN architecture with corresponding features (Figure 7A). The architecture relies upon the 1-GNN described by Morris et al.[39] and implemented in the Pytorch Geometric[91] *GraphConv()* function, using a summing aggregator and three fully connected layers of size 64 as described in the original article. This architecture was benchmarked using an independent dataset, the TU-molecular dataset,[92] showing 64 as the most performant batch size, which we considered a default value and used for our GNN as well. We use a cross-entropy loss function and report training accuracy as a proportion of correct tissue classifications.

Graph data loading

The Reactome reaction network contains directed edges connecting pairs of reactions, and the network is presented to our GNN in a reaction-node manner whereby the network is decomposed into 10,726 subnetworks where each component is represented by a following reaction and all of its preceding reactions (Figures 7B and 7C). The preceding reactions are considered the following reaction's neighborhood, and their values are combined with the neighborhood aggregation function described in the original manuscript[39]:

$$x_v^{(l+1)} = W_1^{(l+1)} x_v^{(l)} + W_2^{(l+1)} \sum_{w \in N(v)} x_w^{(l)},$$

where l is the time-step, x is the value of the vertex, v is the current vertex, $N(v)$ is the neighborhood—preceding reactions—of v, and W_1 and W_2 are weights learned during the training procedure for the current vertex and neighborhood, respectively.

Graph visualization

To visualize the graph resulting from our analysis (Figure 5), we used Cytoscape,[93] v.3.9.1.

RNA-seq data processing

GTEx RNA-seq data files were downloaded from the Recount2 project website at https://jhubiostatistics.shinyapps.io/recount/, and records annotated with healthy tissue labels were retained. Samples removed included those annotated as "cells - transformed fibroblasts" (306), "cells - leukemia cell line (CML)" (102), and "cells - EBV-transformed lymphocytes" (139) and those with no tissue label (5). Transcripts whose identifiers were not annotated as participating in Reactome reactions were removed. Values were scaled up to the machine maximum integer value −1 and added to 1, producing transcript pseudocounts,[71,94] and processed using the variance stabilizing transform[95] provided by DESeq2.[96]

Traditional differential gene expression and pathway enrichment analysis

Reactome.org pathway enrichment analysis of SRA:SRP035988

Enrichment was performed using three differential expression thresholds described in the main text: strict, default, and relaxed. DE transcripts were uploaded to the Reactome.org analysis web tool (=https://reactome.org/PathwayBrowser/#TOOL=AT). Pathway enrichment reports were generated and downloaded from Reactome.org and are available. See Data S5, S6, and S7 for the enrichment reports.

Hypergeometric enrichment analysis edge rejection

Transcripts found to be DE = 904.

Transcripts in SRA:SRP035988 annotated in Reactome reaction network = 5,401.

Edges in reaction network = 40,032.

We set discoverable edge significance considering a Bonferroni-adjusted maximum p value of 0.05:

$$\frac{0.05}{40032} \approx 1.249 \times 10^{-6}.$$

Using Fisher's formula for hypergeometric enrichment,

$$p = \frac{\binom{a+b}{a}\binom{c+d}{c}}{\binom{n}{a+c}} = \frac{\binom{a+b}{b}\binom{c+d}{d}}{\binom{n}{b+d}} = \frac{(a+b)!(c+d)!(a+c)!(b+d)!}{a!b!c!d!n!},$$

where a is the number of DE transcripts representing the edge, b is the number of transcripts representing the edge that are not DE, c is the number of DE transcripts that are not representing that the edge d is the number of DE transcripts, and n is the number of transcripts. We set $b = 0$ and see that edges represented by fewer than 8 transcripts cannot be discovered as significant:

$$p = \frac{a!(c+d)!(a+c)!d!}{a!c!d!n!} = \frac{(c+d)!(a+c)!}{c!n!} = \frac{((904-7)+(5401-904))!(7+(904-7))!}{(904-7)!5401!} \approx 3.609 \times 10^{-6} > 1.249 \times 10^{-6}.$$

SUPPLEMENTAL INFORMATION

Supplemental information can be found online at https://doi.org/10.1016/j.patter.2023.100758.

ACKNOWLEDGMENTS

This work was partially supported by the NIH grants P20GM103466, U54MD007601, P30GM114737, P20GM139753, U54GM138062, P30CA071789, and U41HG003751, as well as the National Library of Medicine Informatics Training Grant T15LM007088-29. The technical support and advanced computing resources from University of Hawaii Information Technology Services – Cyberinfrastructure were funded in part by the National Science Foundation MRI award #1920304.

AUTHOR CONTRIBUTIONS

Conceptualization, J.G.B.; methodology, J.G.B., G.W., X.S., S.M., and M.H.W.; software, J.G.B.; validation, J.G.B.; formal analysis, J.G.B.; investigation, J.G.B.; writing – original draft, J.G.B., G.W., X.S., S.M., and M.H.W.; writing – review & editing, J.G.B., G.W., X.S., F.R., S.M., M.H.W., and Y.D.; visualization, J.G.B.; supervision, G.W., X.S., S.M., M.H.W., and Y.D.; funding acquisition, J.G.B., G.W., and Y.D.

DECLARATION OF INTERESTS

The authors declare no competing interests.

Received: September 23, 2022
Revised: November 20, 2022
Accepted: May 1, 2023
Published: May 22, 2023

REFERENCES

1. Melé, M., Ferreira, P.G., Reverter, F., DeLuca, D.S., Monlong, J., Sammeth, M., Young, T.R., Goldmann, J.M., Pervouchine, D.D., Sullivan, T.J., and Johnson, R. (2015). Human genomics. The human transcriptome across tissues and individuals. Science *348*, 660–665.
2. Suntsova, M., Gaifullin, N., Allina, D., Reshetun, A., Li, X., Mendeleeva, L., Surin, V., Sergeeva, A., Spirin, P., Prassolov, V., et al. (2019). Atlas of RNA sequencing profiles for normal human tissues. Sci. Data *6*, 36–39.
3. Wang, D., Eraslan, B., Wieland, T., Hallström, B., Hopf, T., Zolg, D.P., Zecha, J., Asplund, A., Li, L.H., Meng, C., et al. (2019 Feb). A deep proteome and transcriptome abundance atlas of 29 healthy human tissues. Mol. Syst. Biol. *15*, e8503.
4. Wilhelm, M., Schlegl, J., Hahne, H., Gholami, A.M., Lieberenz, M., Savitski, M.M., Ziegler, E., Butzmann, L., Gessulat, S., Marx, H., et al. (2014). Mass-spectrometry-based draft of the human proteome. Nature *509*, 582–587.
5. Aebersold, R., and Mann, M. (2016). Mass-spectrometric exploration of proteome structure and function. Nature *537*, 347–355.
6. Blagoev, B., Kratchmarova, I., Ong, S.E., Nielsen, M., Foster, L.J., and Mann, M. (2003). A proteomics strategy to elucidate functional protein-protein interactions applied to EGF signaling. Nat. Biotechnol. *21*, 315–318.
7. Meisinger, C., Sickmann, A., and Pfanner, N. (2008). The mitochondrial proteome: from inventory to function. Cell *134*, 22–24.
8. Lundberg, E., Fagerberg, L., Klevebring, D., Matic, I., Geiger, T., Cox, J., Algenäs, C., Lundeberg, J., Mann, M., and Uhlen, M. (2010). Defining the transcriptome and proteome in three functionally different human cell lines. Mol. Syst. Biol. *6*, 450.
9. Lonsdale, J., Thomas, J., Salvatore, M., Phillips, R., Lo, E., Shad, S., Hasz, R., Walters, G., Garcia, F., Young, N., et al. (2013). The genotype-tissue expression (GTEx) project. Nat. Genet. *45*, 580–585.
10. Krishna, R.G., and Wold, F. (1993). Post-translational modifications of proteins. Methods in protein sequence analysis, 167–172.
11. Mann, M., and Jensen, O.N. (2003). Proteomic analysis of post-translational modifications. Nat. Biotechnol. *21*, 255–261.
12. Minton, A.P. (2006). How can biochemical reactions within cells differ from those in test tubes? J. Cell Sci. *119*, 2863–2869.

13. Chen, B.J., Lam, T.C., Liu, L.Q., and To, C.H. (2017 1). Post-translational modifications and their applications in eye research. Mol. Med. Rep. *15*, 3923–3935.
14. Ramazi, S., and Zahiri, J. (2021). Post-translational modifications in proteins: resources, tools and prediction methods. Database *2021*, baab012.
15. Li, W., Li, F., Zhang, X., Lin, H.K., and Xu, C. (2021). Insights into the post-translational modification and its emerging role in shaping the tumor microenvironment. Signal Transduct. Targeted Ther. *6*, 422–430.
16. Mortazavi, A., Williams, B.A., McCue, K., Schaeffer, L., and Wold, B. (2008). Mapping and quantifying mammalian transcriptomes by RNA-Seq. Nat. Methods *5*, 621–628.
17. Schwanhäusser, B., Busse, D., Li, N., Dittmar, G., Schuchhardt, J., Wolf, J., Chen, W., and Selbach, M. (2011). Global quantification of mammalian gene expression control. Nature *473*, 337–342.
18. Liu, Y., Beyer, A., and Aebersold, R. (2016). On the dependency of cellular protein levels on mRNA abundance. Cell *165*, 535–550.
19. Li, J.J., Bickel, P.J., and Biggin, M.D. (2014). System wide analyses have underestimated protein abundances and the importance of transcription in mammals. PeerJ *2*, e270. (a).
20. Lash, L.H., Putt, D.A., and Cai, H. (2008). Drug metabolism enzyme expression and activity in primary cultures of human proximal tubular cells. Toxicology *244*, 56–65.
21. Jens, M., and Rajewsky, N. (2015). Competition between target sites of regulators shapes post-transcriptional gene regulation. Nat. Rev. Genet. *16*, 113–126.
22. Rieder, M.J., Carmona, R., Krieger, J.E., Pritchard, K.A., Jr., and Greene, A.S. (1997). Suppression of angiotensin-converting enzyme expression and activity by shear stress. Circ. Res. *80*, 312–319.
23. Wassmann, S., Wassmann, K., and Nickenig, G. (2004). Modulation of oxidant and antioxidant enzyme expression and function in vascular cells. Hypertension *44*, 381–386.
24. Johnston, J.A., Liu, W.W., Todd, S.A., Coulson, D., Murphy, S., Irvine, G.B., and Passmore, A.P. (2005). Expression and activity of β-site amyloid precursor protein cleaving enzyme in Alzheimer's disease. Biochem. Soc. Trans. *33*, 1096–1100.
25. García-López, D., Häkkinen, K., Cuevas, M.J., Lima, E., Kauhanen, A., Mattila, M., Sillanpää, E., Ahtiainen, J.P., Karavirta, L., Almar, M., and González-Gallego, J. (2007). Effects of strength and endurance training on antioxidant enzyme gene expression and activity in middle-aged men. Scand. J. Med. Sci. Sports *17*, 595–604.
26. Mauriz, J.L., Molpeceres, V., García-Mediavilla, M.V., González, P., Barrio, J.P., and González-Gallego, J. (2007). Melatonin prevents oxidative stress and changes in antioxidant enzyme expression and activity in the liver of aging rats. J. Pineal Res. *42*, 222–230.
27. Ronis, M.J.J. (2016). Effects of soy containing diet and isoflavones on cytochrome P450 enzyme expression and activity. Drug Metab. Rev. *48*, 331–341.
28. Xie, C., Yan, L., Gong, W., Zhu, Z., Tan, S., Chen, D., Hu, Z., and Peng, Y. (2016). Effects of different substrates on lignocellulosic enzyme expression, enzyme activity, substrate utilization and biological efficiency of Pleurotus eryngii. Cell. Physiol. Biochem. *39*, 1479–1494.
29. Matsumoto, M., Matsuzaki, F., Oshikawa, K., Goshima, N., Mori, M., Kawamura, Y., Ogawa, K., Fukuda, E., Nakatsumi, H., Natsume, T., et al. (2017). A large-scale targeted proteomics assay resource based on an in vitro human proteome. Nat. Methods *14*, 251–258.
30. Dammann, C., Stapelfeld, C., and Maser, E. (2019). Expression and activity of the cortisol-activating enzyme 11β-hydroxysteroid dehydrogenase type 1 is tissue and species-specific. Chem. Biol. Interact. *303*, 57–61.
31. Stelzl, U., Worm, U., Lalowski, M., Haenig, C., Brembeck, F.H., Goehler, H., Stroedicke, M., Zenkner, M., Schoenherr, A., Koeppen, S., et al. (2005). A human protein-protein interaction network: a resource for annotating the proteome. Cell *122*, 957–968.
32. Price, N.D., and Shmulevich, I. (2007). Biochemical and statistical network models for systems biology. Curr. Opin. Biotechnol. *18*, 365–370.
33. Bossi, A., and Lehner, B. (2009). Tissue specificity and the human protein interaction network. Mol. Syst. Biol. *5*, 260.
34. Wu, G., Feng, X., and Stein, L. (2010). A human functional protein interaction network and its application to cancer data analysis. Genome Biol. *11*. R53-R23.
35. Wu, G., and Haw, R. (2017). Functional interaction network construction and analysis for disease discovery. In Protein bioinformatics (Humana Press, New York, NY), pp. 235–253.
36. Hamilton, W., Ying, Z., and Leskovec, J. (2017). Inductive representation learning on large graphs. In Advances in Neural Information Processing Systems, *30*.
37. Gillespie, M., Jassal, B., Stephan, R., Milacic, M., Rothfels, K., Senff-Ribeiro, A., Griss, J., Sevilla, C., Matthews, L., Gong, C., et al. (2022). The reactome pathway knowledgebase 2022. Nucleic Acids Res. *50*, D687–D692.
38. Pearson, K. (1901). LIII. On lines and planes of closest fit to systems of points in space. London, Edinburgh Dublin Phil. Mag. J. Sci. *2*, 559–572.
39. Morris, C., Ritzert, M., Fey, M., Hamilton, W.L., Lenssen, J.E., Rattan, G., and Grohe, M. (2019). Weisfeiler and leman go neural: higher-order graph neural networks. In Proceedings of the AAAI Conference on Artificial Intelligence, *33*, pp. 4602–4609.
40. Cancer Genome Atlas Research Network, Weinstein, J.N., Collisson, E.A., Mills, G.B., Shaw, K.R.M., Ozenberger, B.A., Ellrott, K., Shmulevich, I., Sander, C., and Stuart, J.M. (2013). The cancer genome atlas pan-cancer analysis project. Nat. Genet. *45*, 1113–1120.
41. He, K., Zhang, X., Ren, S., and Sun, J. (2016). Deep residual learning for image recognition. In Proceedings of the IEEE conference on computer vision and pattern recognition, pp. 770–778.
42. Li, B., Tsoi, L.C., Swindell, W.R., Gudjonsson, J.E., Tejasvi, T., Johnston, A., Ding, J., Stuart, P.E., Xing, X., Kochkodan, J.J., et al. (2014). Transcriptome analysis of psoriasis in a large case–control sample: RNA-seq provides insights into disease mechanisms. J. Invest. Dermatol. *134*, 1828–1838.
43. Zhao, Y., Jhamb, D., Shu, L., Arneson, D., Rajpal, D.K., and Yang, X. (2019). Multi-omics integration reveals molecular networks and regulators of psoriasis. BMC Syst. Biol. *13*. 8-4.
44. Huang, T., Gao, Z., Zhang, Y., Fan, K., Wang, F., Li, Y., Zhong, J., Fan, H.Y., Cao, Q., Zhou, J., et al. (2018). CRL4DCAF2 negatively regulates IL-23 production in dendritic cells and limits the development of psoriasis. J. Exp. Med. *215*, 1999–2017.
45. Collado-Torres, L., Nellore, A., Kammers, K., Ellis, S.E., Taub, M.A., Hansen, K.D., Jaffe, A.E., Langmead, B., and Leek, J.T. (2017). Reproducible RNA-seq analysis using recount2. Nat. Biotechnol. *35*, 319–321.
46. Van der Maaten, L., and Hinton, G. (2008). Visualizing data using t-SNE. J. Mach. Learn. Res. *9*.
47. McInnes, L., Healy, J., and Melville, J. (2018). Umap: uniform manifold approximation and projection for dimension reduction. Preprint at arXiv. https://doi.org/10.48550/arXiv.1802.03426.
48. Moon, K.R., van Dijk, D., Wang, Z., Gigante, S., Burkhardt, D.B., Chen, W.S., Yim, K., Elzen, A.v.d., Hirn, M.J., Coifman, R.R., et al. (2019). Visualizing structure and transitions in high-dimensional biological data. Nat. Biotechnol. *37*, 1482–1492.
49. R Core Team (2022). R: A Language and Environment for Statistical Computing (Vienna, Austria: R Foundation for Statistical Computing). https://www.R-project.org/.
50. Ward, J.H., Jr. (1963). Hierarchical grouping to optimize an objective function. J. Am. Stat. Assoc. *58*, 236–244.
51. Murtagh, F., and Legendre, P. (2014). Ward's hierarchical agglomerative clustering method: which algorithms implement Ward's criterion? J. Classif. *31*, 274–295.
52. Sokal, R.R., and Rohlf, F.J. (1962). The comparison of dendrograms by objective methods. Taxon *11*, 33–40.
53. Sneath, P.H. (1973). Taxonomic structure. Numerical taxonomy, 230–234.

54. Galili, T. (2015). dendextend: an R package for visualizing, adjusting and comparing trees of hierarchical clustering. Bioinformatics *31*, 3718–3720.
55. Pointer, I. (2019). Programming PyTorch for Deep Learning: Creating and Deploying Deep Learning Applications (O'Reilly Media, Inc.).
56. Paszke, A., Gross, S., Chintala, S., Chanan, G., Yang, E., DeVito, Z., Lin, Z., Desmaison, A., Antiga, L., and Lerer, A. (2017). Automatic Differentiation in Pytorch.
57. Ray, J., Pinar, A., and Seshadhri, C. (2012). Are we there yet? When to stop akov chain while generating random graphs. InInternational Workshop on Algorithms and Models for the Web-Graph (Berlin, Heidelberg: Springer), pp. 153–164.
58. Kitami, T., and Nadeau, J.H. (2002). Biochemical networking contributes more to genetic buffering in human and mouse metabolic pathways than does gene duplication. Nat. Genet. *32*, 191–194.
59. Yosinski, J., Clune, J., Bengio, Y., and Lipson, H. (2014). How transferable are features in deep neural networks? Adv. Neural Inf. Process. Syst. *27*.
60. Hubert, L., and Arabie, P. (1985). Comparing partitions. J. Classif. *2*, 193–218.
61. Atkinson, O., Bhardwaj, A., Englert, C., Ngairangbam, V.S., and Spannowsky, M. (2021). Anomaly detection with convolutional graph neural networks. J. High Energy Phys. *2021*, 80–89.
62. Deng, A., and Hooi, B. (2021). Graph neural network-based anomaly detection in multivariate time series. In Proceedings of the AAAI Conference on Artificial Intelligence, *35*, pp. 4027–4035. No. 5.
63. Wu, Y., Dai, H.N., and Tang, H. (2022). Graph neural networks for anomaly detection in industrial internet of things. IEEE Internet Things J. *9*, 9214–9231.
64. o Clinic. Psoriasis: symptoms & causes. n.d. https://www.mayoclinic.org/diseases-conditions/psoriasis/symptoms-causes/syc-20355840
65. Rachakonda, T.D., Schupp, C.W., and Armstrong, A.W. (2014). Psoriasis prevalence among adults in the United States. J. Am. Acad. Dermatol. *70*, 512–516.
66. Rendon, A., and Schäkel, K. (2019). Psoriasis pathogenesis and treatment. Int. J. Mol. Sci. *20*, 1475.
67. Sundararajan, M., Taly, A., and Yan, Q. (2017). Axiomatic attribution for deep networks. In International Conference on Machine Learning (PMLR), pp. 3319–3328.
68. Kokhlikyan, N., Miglani, V., tin, M., Wang, E., Alsallakh, B., Reynolds, J., Melnikov, A., Kliushkina, N., Araya, C., Yan, S., and Reblitz-Richardson, O. (2020). Captum: a unified and generic model interpretability library for pytorch. Preprint at arXiv. https://doi.org/10.48550/arXiv.2009.07896.
69. Soucy, T.A., Dick, L.R., Smith, P.G., Milhollen, M.A., and Brownell, J.E. (2010). The NEDD8 conjugation pathway and its relevance in cancer biology and therapy. Genes Cancer *1*, 708–716.
70. Blighe, K., Rana, S., and Lewis, M. (2021). EnhancedVolcano: publication-ready volcano plots with enhanced colouring and labeling. Available at: https://github.com/kevinblighe/EnhancedVolcano
71. Zhu, A., Ibrahim, J.G., and Love, M.I. (2019). Heavy-tailed prior distributions for sequence count data: removing the noise and preserving large differences. Bioinformatics *35*, 2084–2092.
72. Stephens, M. (2017). False discovery rates: a new deal. Biostatistics *18*, 275–294.
73. Kunjappu, M.J., and Hochstrasser, M. (2014). Assembly of the 20S proteasome. Biochim. Biophys. Acta Mol. Cell Res. *1843*, 2–12.
74. Harshbarger, W., Miller, C., Diedrich, C., and Sacchettini, J. (2015). Crystal structure of the human 20S proteasome in complex with carfilzomib. Structure *23*, 418–424.
75. Tanaka, K. (2009). The proteasome: overview of structure and functions. Proc. Jpn. Acad. Ser. B Phys. Biol. Sci. *85*, 12–36.
76. Livneh, I., Cohen-Kaplan, V., Cohen-Rosenzweig, C., Avni, N., and Ciechanover, A. (2016). The life cycle of the 26S proteasome: from birth, through regulation and function, and onto its death. Cell Res. *26*, 869–885.
77. Sidis, W.J. (1925). The Animate and the Inanimate (RG Badger).
78. Otero-Muras, I., Yordanov, P., and Stelling, J. (2017). Chemical reaction network theory elucidates sources of multistability in interferon signaling. PLoS Comput. Biol. *13*, e1005454.
79. Cortassa, S., Aon, M.A., and Thomas, D. (1990). Thermodynamic and kinetic studies of a stoichiometric model of energetic metabolism under starvation conditions. FEMS Microbiol. Lett. *66*, 249–255.
80. Tyson, J.J., Laomettachit, T., and Kraikivski, P. (2019). Modeling the dynamic behavior of biochemical regulatory networks. J. Theor. Biol. *462*, 514–527.
81. Sanwal, U., Hoang, T.S., Petre, L., and Petre, I. (2022). Scalable reaction network modeling with automatic validation of consistency in Event-B. Sci. Rep. *12*, 1287–1289.
82. Ma, J., Yu, M.K., Fong, S., Ono, K., Sage, E., Demchak, B., Sharan, R., and Ideker, T. (2018). Using deep learning to model the hierarchical structure and function of a cell. Nat. Methods *15*, 290–298.
83. Yang, J.H., Wright, S.N., Hamblin, M., McCloskey, D., Alcantar, M.A., Schrübbers, L., Lopatkin, A.J., Satish, S., Nili, A., Palsson, B.O., et al. (2019). A white-box machine learning approach for revealing antibiotic mechanisms of action. Cell *177*, 1649–1661.e9.
84. Kuenzi, B.M., Park, J., Fong, S.H., Sanchez, K.S., Lee, J., Kreisberg, J.F., Ma, J., and Ideker, T. (2020). Predicting drug response and synergy using a deep learning model of human cancer cells. Cancer Cell *38*, 672–684.e6.
85. Anderson, D.F., Joshi, B., and Deshpande, A. (2021). On reaction network implementations of neural networks. J. R. Soc. Interface *18*, 20210031.
86. Ashburner, M., Ball, C.A., Blake, J.A., Botstein, D., Butler, H., Cherry, J.M., Davis, A.P., Dolinski, K., Dwight, S.S., Eppig, J.T., et al. (2000). Gene ontology: tool for the unification of biology. Nat. Genet. *25*, 25–29.
87. Blake, J.A. (2013). Ten quick tips for using the gene ontology. PLoS Comput. Biol. *9*, e1003343.
88. Kramer, M., Dutkowski, J., Yu, M., Bafna, V., and Ideker, T. (2014). Inferring gene ontologies from pairwise similarity data. Bioinformatics *30*, i34–i42.
89. Madala, V.C., and Chandrasekaran, S. (2022). CNNs are Myopic. Preprint at arXiv. https://doi.org/10.48550/arXiv.2205.10760.
90. Salzberg, S.L. (2018). Open questions: how many genes do we have? BMC Biol. *16*, 1–3.
91. Fey, M., and Lenssen, J.E. (2019). Fast graph representation learning with PyTorch Geometric. Preprint at arXiv. https://doi.org/10.48550/arXiv.1903.02428.
92. Morris, C., Kriege, N.M., Bause, F., Kersting, K., Mutzel, P., and Neumann, M. (2020). Tudataset: a collection of benchmark datasets for learning with graphs. Preprint at arXiv. https://doi.org/10.48550/arXiv.2007.08663.
93. Shannon, P., kiel, A., Ozier, O., Baliga, N.S., Wang, J.T., Ramage, D., Amin, N., Schwikowski, B., and Ideker, T. (2003). Cytoscape: a software environment for integrated models of biomolecular interaction networks. Genome Res. *13*, 2498–2504.
94. Love, M.I., Anders, S., Kim, V., and Huber, W. (2015). RNA-Seq workflow: gene-level exploratory analysis and differential expression. F1000Res. *4*, 1070.
95. Anders, S., and Huber, W. (2010). Differential expression analysis for sequence count data. Genome Biol. *11*, R106.
96. Love, M.I., Huber, W., and Anders, S. (2014). Moderated estimation of fold change and dispersion for RNA-seq data with DESeq2. Genome Biol. *15*, 1–21.

Patterns, Volume *4*

Supplemental information

Biology-inspired graph neural network encodes reactome and reveals biochemical reactions of disease

Joshua G. Burkhart, Guanming Wu, Xubo Song, Francesco Raimondi, Shannon McWeeney, Melissa H. Wong, and Youping Deng

Supplementary Figures & Figure Legends

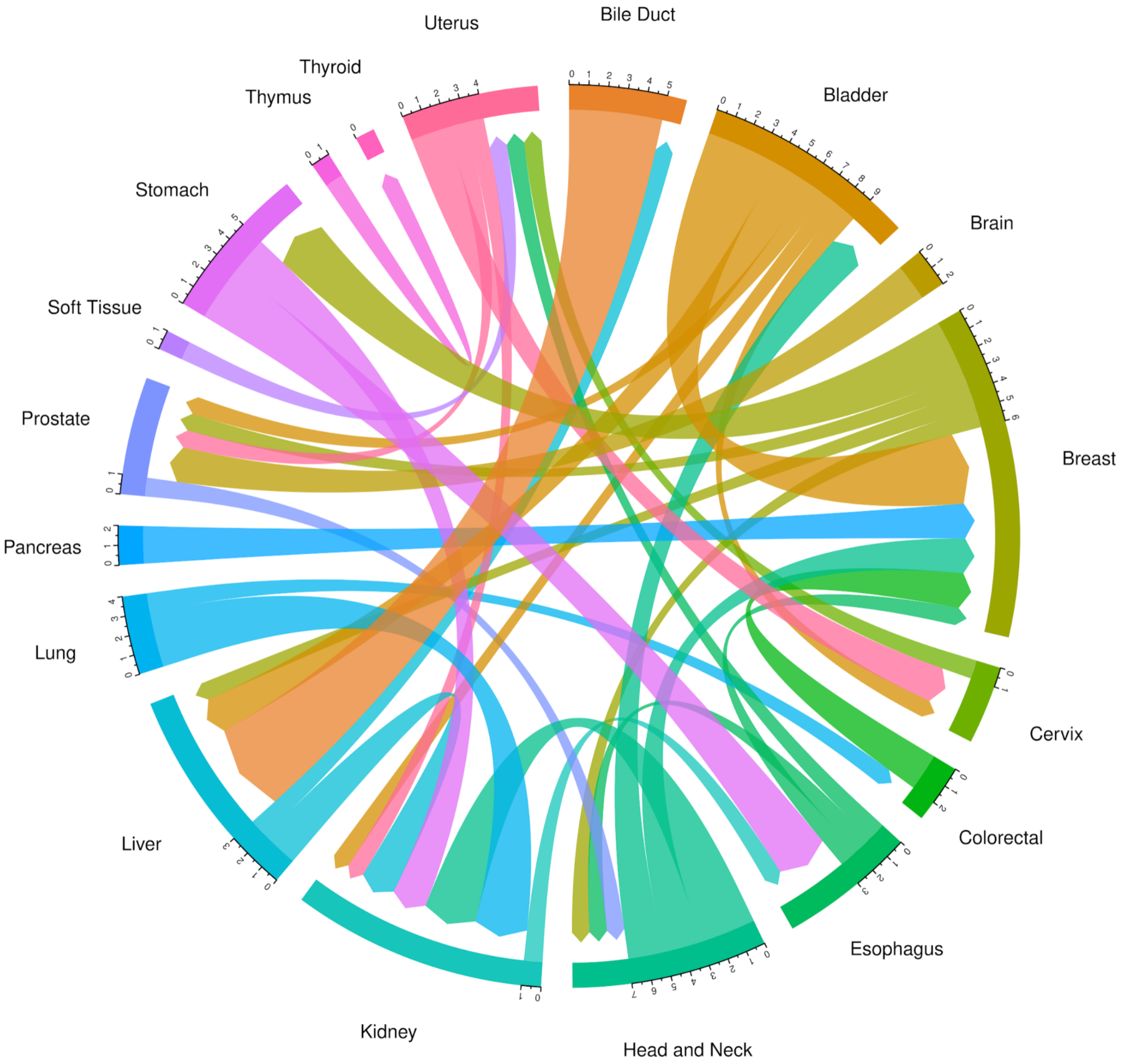

Supplementary Figure 1: Resnet TCGA Tissue Sample Misclassifications

Chord diagram showing Resnet TCGA tissue misclassifications from the held-out dataset of 370 samples. True sample labels are numbered on the edge of the diagram and their misclassified label is depicted as the target of the corresponding ribbon. For example, the Resnet model misclassified 1 cervix sample as uterus and it misclassified 2 uterus samples and 1 bladder sample as cervix.

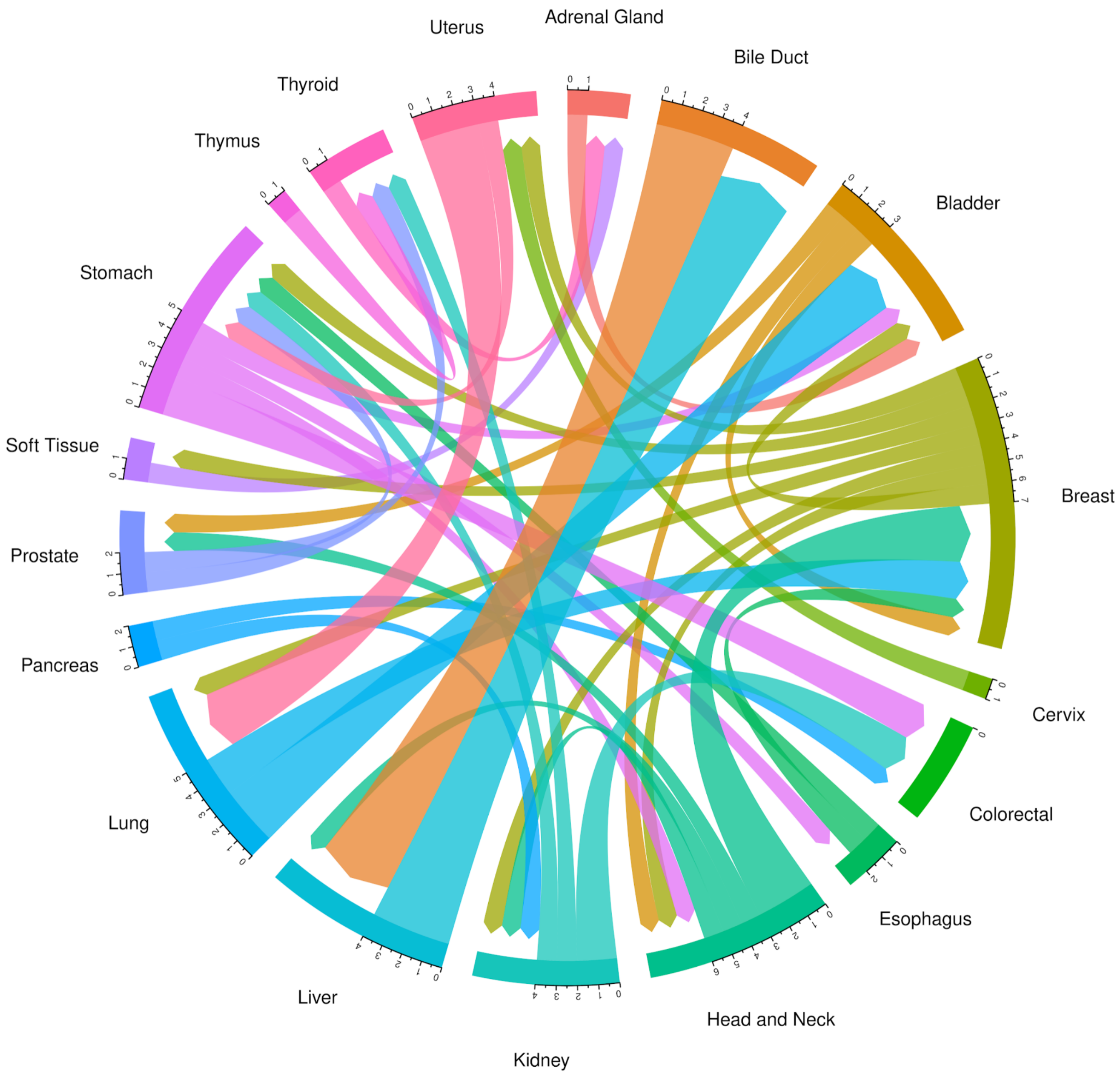

Supplementary Figure 2: GNN TCGA Tissue Sample Misclassifications

Chord diagram showing GNN TCGA tissue misclassifications from the held-out dataset of 370 samples. True sample labels are numbered on the edge of the diagram and their misclassified label is depicted as the target of the corresponding ribbon. For example, the GNN model misclassified 0 colorectal samples; however, it misclassified 2 stomach samples, 2 kidney samples and 1 pancreas sample as colorectal.

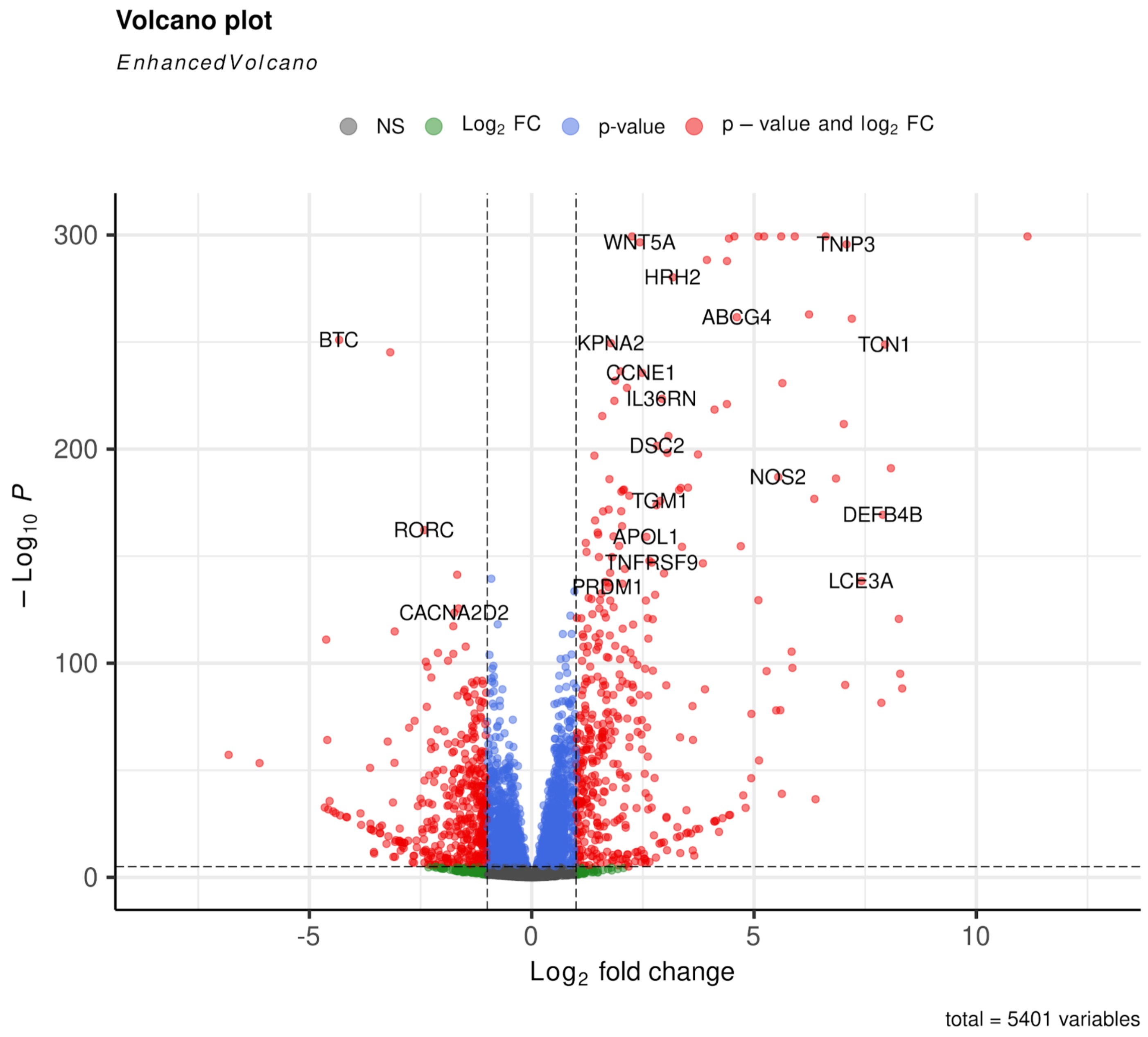

Supplementary Figure 3: EnhancedVolcano Plot for SRP035988 using Default Parameters

Volcano plot generated using the EnhancedVolcano package showing significantly differentially expressed genes found in the SRP035988 dataset using default parameters. Genes reported in prior literature as associated with psoriasis (Table S2) are labeled.

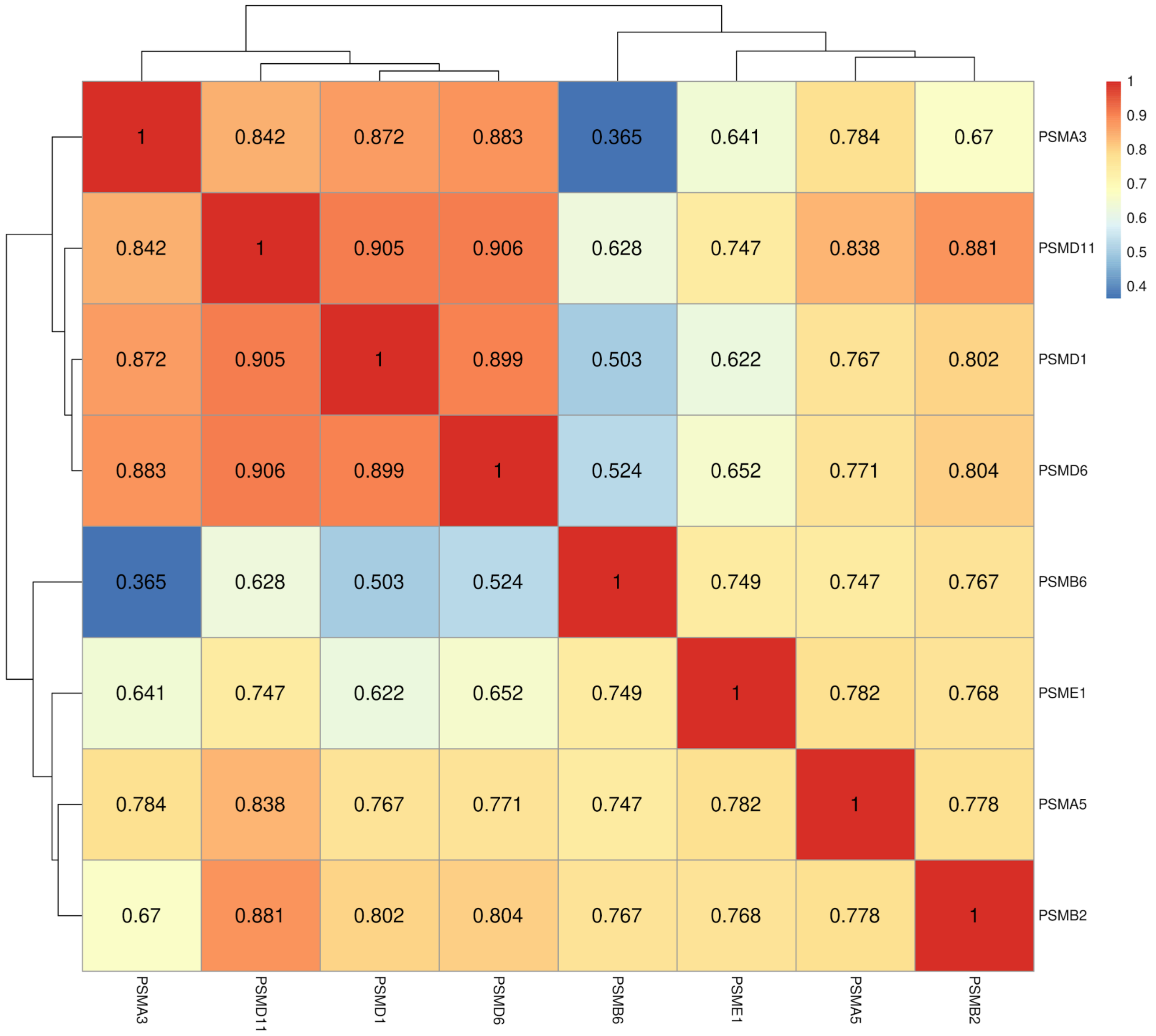

Supplementary Figure 4: R-HSA-8956184 Differentially Expressed Gene Correlation

Pearson correlation coefficients of significantly differentially expressed genes of R-HSA-8956184 form two distinct clusters.

Supplementary Tables

Supplementary Table 1: TCGA Misclassification Sample Counts

Table showing TCGA tissue misclassification counts from the held-out dataset of 370 samples for each model.

TCGA Tissue Label	# Miscalled by both models	# Miscalled by Resnet only	# Miscalled by GNN only
Adrenal Gland	0	0	1
Bile Duct	4	1	0

Bladder	3	6	0
Brain	0	2	0
Breast	2	4	5
Cervix	1	0	0
Colorectal	0	2	0
Esophagus	1	2	1
Head and Neck	3	4	3
Kidney	1	0	3
Liver	0	3	4
Lung	0	4	5
Pancreas	2	0	0
Prostate	0	1	2
Soft Tissue	1	0	0
Stomach	2	3	3
Thymus	1	0	0
Thyroid	0	0	1
Uterus	1	3	3
(Total)	22	35	31

Supplementary Table 2: Significant Differentially Expressed Genes of EnhancedVolcano

Significantly differentially expressed genes found in the SRP035988 dataset using default parameters of EnhancedVolcano reported in prior literature as associated with psoriasis.

Gene Name	Ensembl ID	Overexpression Group	Supporting Literature PMID
WNT5A	ENSG00000114251	Lesional psoriatic skin	31313518
TNIP3	ENSG00000050730	Lesional psoriatic skin	25521225
HRH2	ENSG00000113749	Lesional psoriatic skin	29317264
ABCG4	ENSG00000172350	Lesional psoriatic skin	25505559
BTC	ENSG00000174808	Normal skin	20220767
KPNA2	ENSG00000182481	Lesional psoriatic skin	24098495
TCN1	ENSG00000134827	Lesional psoriatic skin	23915137
CCNE1	ENSG00000105173	Lesional psoriatic skin	21711342
IL36RN	ENSG00000136695	Lesional psoriatic skin	24129779
DSC2	ENSG00000134755	Lesional psoriatic skin	33995349
NOS2	ENSG00000007171	Lesional psoriatic skin	25539641
TGM1	ENSG00000092295	Lesional psoriatic skin	32106600
RORC	ENSG00000143365	Normal skin	26149470
APOL1	ENSG00000100342	Lesional psoriatic skin	29204449
DEFB4B	ENSG00000177257	Lesional psoriatic skin	32484435
TNFRSF9	ENSG00000049249	Lesional psoriatic skin	24885462
PRDM1	ENSG00000057657	Lesional psoriatic skin	23576729
LCE3A	ENSG00000185962	Lesional psoriatic skin	28634035
CACNA2D2	ENSG00000007402	Normal skin	31611939

Supplementary Table 3: Loci and TFs of Differentially Expressed Genes in R-HSA-8956184

HGNC (Tweedie et al. 2021) bands and top five promoters/enhancers by GeneHancer Score (Fishilevich et al. 2017) of significantly differentially expressed genes of R-HSA-8956184.

Gene Name	Ensembl ID	HGNC Band	Cluster	Promoter/Enhancer List
PSMD11	ENSG00000108671	17q11.2	1	GH17J032348, GH17J032485, GH17J032443, GH17J031548, GH17J032302
PSMA5	ENSG00000143106	1p13.3	2	GH01J109389, GH01J109423, GH01J109438, GH01J109441, GH01J109466
PSME1	ENSG00000092010	14q12	2	GH14J024422, GH14J024240, GH14J024270, GH14J024139, GH14J024135
PSMB2	ENSG00000126067	1p34.3	2	GH01J035717, GH01J035636, GH01J035707, GH01J035715, GH01J035577
PSMA3	ENSG00000100567	14q23.1	1	GH14J058242, GH14J058199, GH14J058238, GH14J058271, GH14J058280
PSMD1	ENSG00000173692	2q37.1	1	GH02J230863, GH02J230711, GH02J231056, GH02J231407, GH02J231052
PSMD6	ENSG00000163636	3p14.1	1	GH03J063910, GH03J064021, GH03J064219, GH03J063966, GH03J064264
PSMB6	ENSG00000142507	17p13.2	2	GH17J004795, GH17J004843, GH17J004842, GH17J004791, GH17J004801

Ancillary R Package References

1. Azzalini A, Menardi G. Clustering via nonparametric density estimation: The R package pdfCluster. arXiv preprint arXiv:1301.6559. 2013 Jan 28. doi: https://doi.org/10.48550/arXiv.1301.6559
2. Bache SM and Wickham H, (2022). magrittr: A Forward-Pipe Operator for R. R package version 2.0.2. https://CRAN.R-project.org/package=magrittr
3. Chen H, (2022). VennDiagram: Generate High-Resolution Venn and Euler Plots. R package version 1.7.3. https://CRAN.R-project.org/package=VennDiagram
4. Dewey M, (2022). metap: meta-analysis of significance values. R package version 1.8.
5. Donaldson J, (2022). tsne: T-Distributed Stochastic Neighbor Embedding for R (t-SNE). R package version 0.1-3.1. https://CRAN.R-project.org/package=tsne
6. Durinck S, Spellman PT, Birney E, Huber W. Mapping identifiers for the integration of genomic datasets with the R/Bioconductor package biomaRt. Nature protocols. 2009 Aug;4(8):1184-91.
7. Eckert A, (2022). parallelDist: Parallel Distance Matrix Computation using Multiple Threads. R package version 0.2.6. https://CRAN.R-project.org/package=parallelDist
8. Fishilevich S, Nudel R, Rappaport N, Hadar R, Plaschkes I, Iny Stein T, Rosen N, Kohn A, Twik M, Safran M, Lancet D. GeneHancer: genome-wide integration of enhancers and target genes in GeneCards. Database. 2017 Jan 1;2017.
9. Flor M, (2022). chorddiag: Interactive Chord Diagrams. R package version 0.1.3. https://github.com/mattflor/chorddiag/
10. Garnier S, Ross N, Rudis R, Camargo AP, Sciaini M, and Scherer C, (2021). Rvision - Colorblind-Friendly Color Maps for R. R package version 0.6.2
11. Gohel D and Skintzos P, (2022). ggiraph: Make 'ggplot2' Graphics Interactive. R package version 0.8.3. https://CRAN.R-project.org/package=ggiraph
12. Gu Z, Gu L, Eils R, Schlesner M, Brors B. Circlize implements and enhances circular visualization in R. Bioinformatics. 2014 Oct 1;30(19):2811-2.

13. Hatje K, Rahman RU, Vidal RO, Simm D, Hammesfahr B, Bansal V, Rajput A, Mickael ME, Sun T, Bonn S, Kollmar M. The landscape of human mutually exclusive splicing. Molecular systems biology. 2017 Dec;13(12):959.
14. Hosmer DW, Lemeshow S. Applied Logistic Regression, 2nd Ed. Chapter 5. New York, NY: John Wiley and Sons, 2000. Pp. 160 –164.
15. Hočevar T, Demšar J. Computation of graphlet orbits for nodes and edges in sparse graphs. Journal of Statistical Software. 2016 Jul 28;71:1-24.
16. Huber W, Carey VJ, Gentleman R, Anders S, Carlson M, Carvalho BS, Bravo HC, Davis S, Gatto L, Girke T, Gottardo R. Orchestrating high-throughput genomic analysis with Bioconductor. Nature methods. 2015 Feb;12(2):115-21.
17. Kassambara A and Mundt F, (2020). factoextra: Extract and Visualize the Results of Multivariate Data Analyses. R package version 1.0.7. https://CRAN.R-project.org/package=factoextra
18. Kolde R, (2019). pheatmap: Pretty Heatmaps. R package version 1.0.12. https://CRAN.R-project.org/package=pheatmap
19. Konopka T, (2022). umap: Uniform Manifold Approximation and Projection. R package version 0.2.9.0. https://CRAN.R-project.org/package=umap
20. Kuhn M. Caret: classification and regression training. Astrophysics Source Code Library. 2015 May:ascl-1505.
21. Mandrekar JN. Receiver operating characteristic curve in diagnostic test assessment. Journal of Thoracic Oncology. 2010 Sep 1;5(9):1315-6.
22. Mecham BH, Nelson PS, Storey JD. Supervised normalization of microarrays. Bioinformatics. 2010 May 15;26(10):1308-15.
23. Microsoft and Weston S, (2022). foreach: Provides Foreach Looping Construct. R package version 1.5.2. https://CRAN.R-project.org/package=foreach
24. Microsoft Corporation and Weston S, (2022). doParallel: Foreach Parallel Adaptor for the 'parallel' Package. R package version 1.0.17. https://CRAN.R-project.org/package=doParallel
25. Morgan M, Obenchain V, Hester J and Pagès H, (2021). SummarizedExperiment: SummarizedExperiment container. R package version 1.24.0. https://bioconductor.org/packages/SummarizedExperiment
26. Neuwirth E, (2022). RColorBrewer: ColorBrewer Palettes. R package version 1.1-3. https://CRAN.R-project.org/package=RColorBrewer
27. Pedersen TL, (2022). patchwork: The Composer of Plots. R package version 1.1.2. https://CRAN.R-project.org/package=patchwork
28. Ritchie ME, Phipson B, Wu DI, Hu Y, Law CW, Shi W, Smyth GK. limma powers differential expression analyses for RNA-sequencing and microarray studies. Nucleic acids research. 2015 Apr 20;43(7):e47-.
29. Rudis B, (2020). hrbrthemes: Additional Themes, Theme Components and Utilities for 'ggplot2'. R package version 0.8.0. https://CRAN.R-project.org/package=hrbrthemes
30. Sievert C. Interactive web-based data visualization with R, plotly, and shiny. CRC Press; 2020 Jan 30.
31. Signorell A, Aho K, Alfons A, Anderegg N, Aragon T, Arppe A, Baddeley A, Barton K, Bolker B, Borchers HW. DescTools: Tools for descriptive statistics. R package version 0.99. 2019;28:17.
32. Tang Y, Horikoshi M, Li W. ggfortify: unified interface to visualize statistical results of popular R packages. R J.. 2016 Dec 1;8(2):474.
33. Tweedie S, Braschi B, Gray K, Jones TE, Seal RL, Yates B, Bruford EA. Genenames. org: the HGNC and VGNC resources in 2021. Nucleic acids research. 2021 Jan 8;49(D1):D939-46.
34. Venables WN, Ripley BD, (2002). Modern Applied Statistics with S, Fourth edition. Springer, New York. ISBN 0-387-95457-0, https://www.stats.ox.ac.uk/pub/MASS4/.
35. Wei R and Wang J, (2018). multiROC: Calculating and Visualizing ROC and PR Curves Across Multi-Class Classifications. R package version 1.1.1. https://CRAN.R-project.org/package=multiROC
36. Wickham H, (2022). stringr: Simple, Consistent Wrappers for Common String Operations. R package version 1.4.1. https://CRAN.R-project.org/package=stringr

37. Wickham H, Averick M, Bryan J, Chang W, McGowan LD, François R, Grolemund G, Hayes A, Henry L, Hester J, Kuhn M. Welcome to the Tidyverse. Journal of open source software. 2019 Nov 21;4(43):1686.
38. Wickham H, François R, Henry L and Müller K, (2022). dplyr: A Grammar of Data Manipulation. R package version 1.0.10. https://CRAN.R-project.org/package=dplyr
39. Wickham H. Package 'ggplot2': elegant graphics for data analysis. Springer-Verlag New York. doi. 2016;10:978-0.
40. Wickham H. Reshaping data with the reshape package. Journal of statistical software. 2007 Nov 13;21:1-20.
41. Wickham H. The split-apply-combine strategy for data analysis. Journal of statistical software. 2011 Apr 7;40:1-29.
42. Wickham H and Girlich M, (2022). tidyr: Tidy Messy Data. R package version 1.2.1. https://CRAN.R-project.org/package=tidyr
43. Wickham H and Seidel D, (2022). scales: Scale Functions for Visualization. R package version 1.2.1. https://CRAN.R-project.org/package=scales
